# Oxidative Rearomatization of Tetrahydroisoquinolines Promoted by Pyridine-*N*-oxide

**DOI:** 10.1021/acs.orglett.4c03186

**Published:** 2024-09-23

**Authors:** Timothy C. Jenkins, Darren L. Poole, Timothy J. Donohoe

**Affiliations:** † Chemistry Research Laboratory, Department of Chemistry, University of Oxford, Mansfield Road, Oxford OX1 3TA, United Kingdom; ‡ Molecular Modalities Discovery, GSK Medicines Research Centre, Stevenage SG1 2NY, United Kingdom

## Abstract

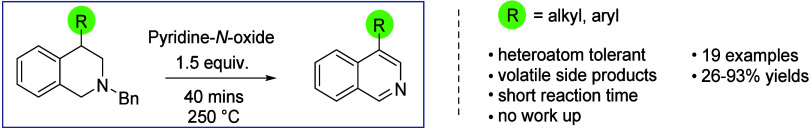

Isoquinolines are ubiquitous arenes found in many biologically useful molecules. While direct substitution at the heterocyclic ring is uncommon, reductive functionalization to form tetrahydroisoquinolines (THIQs) is straightforward. Herein, we describe a facile method for producing C4-functionalized isoquinolines from a readily available parent THIQ. This high-temperature transformation utilizes pyridine-*N*-oxide as an oxidant generating only volatile side products and is functional-group-tolerant.

Isoquinolines and their derivatives are common motifs in a range of pharmaceuticals, agrochemicals, and natural products.^1,2^ Novel methods for synthesizing functionalized isoquinolines from accessible starting materials are, therefore, particularly important. Previously reported methods include arene *de novo* syntheses, which require functionality to be built into a molecule early on and can limit its usefulness in library building. Methods for arene derivatization include CH functionalization, which can require harsh reagents or toxic and expensive metals or have limited scope.^3,4^ The rearomatization of saturated versions of heterocyclic compounds can also be performed using a variety of methods, many of which have issues, such as the need for sacrificial alkenes as oxidants or expensive and toxic metal catalysts.^5−14^

Huang and co-workers reported the use of an iridium catalyst to facilitate transfer hydrogenation from saturated heterocycles, including tetrahydroisoquinoline, to give oxidized arene products; this report contains one example of a tetrahydroisoquinoline (THIQ) to isoquinoline oxidation (Scheme 1a).^15^ Some time ago, Vasil’ev and co-workers reported pyridine-*N*-oxide (PNO) as an inexpensive oxidant in a related reaction utilizing *N*-methylpiperidines as substrates (Scheme 1b).^16^

**Scheme 1:**
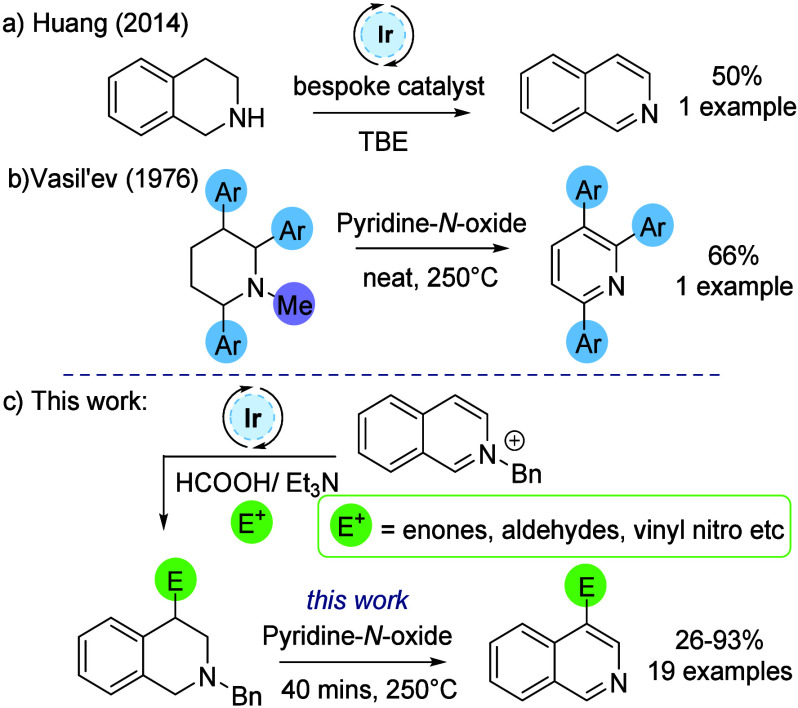
Rearomatization of *N*-Heterocycles

Previous work in the group has focused on the catalytic reductive functionalization of isoquinoliniums to give THIQs using mildly acidic reducing conditions.^17^ The scope of this transformation is broad, and it provides a route from isoquinolines to highly functionalized THIQs with a substituent added to the heterocycle during the reduction (Scheme 1c).

Given the utility of this method, we sought an oxidative rearomatization to expand it, facilitating the transformation of β-functionalized THIQs into their respective β-functionalized isoquinolines. This sequence has potential in biological applications because planarity can be returned post-functionalization, affording a “2D version” of a desired molecule. It is worth noting that we have also worked in the area of temporary dearomatization to functionalize pyridines and isoquinolines *in situ*.^18,19^

We decided to examine PNO as an oxidant for the rearomatization of THIQs, given its low cost and wide availability. However, application of the neat reaction conditions described by Vasil’ev and co-workers afforded a poor yield of product **2** (entry 1 in Table 1). Several parameters were explored to try to improve the yield (Table 1). Sealing the reaction under air in a microwave vial produced the most reproduceable results (see the Supporting Information). Use of camphor as a high-boiling solvent gave a substantial increase in the yield (entry 2 in Table 1); increasing the temperature produced another marked increase, giving an optimum temperature of 250 °C (entries 3 and 4 in Table 1). Investigating the reaction time revealed that the transformation was predominantly finished after 30 min, with an optimum time of 40 min (entries 5 and 6 in Table 1).

**Table 1:**
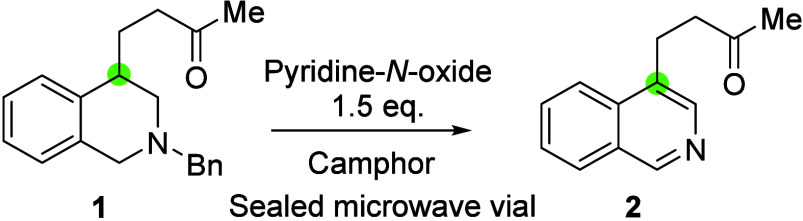
Optimization of Rearomatization of THIQ **1** with PNO^a^

entry	temperature (°C)	PNO (equiv)	time (min)	yield of product **2** (%)^b^
1	200	5.0	40	6^c^^,^^d^
2	200	5.0	40	52
3	250	5.0	40	66^d^
4	270	5.0	40	37^e^
5	250	5.0	30	63
6	250	5.0	60	66^e^
7	250	1.0	40	57
8	250	2.0	40	73
9	250	1.5	40	69

a Reactions were performed on a 0.125 mmol scale.

b Isolated yield.

c The reaction was performed without a solvent.

d Quantitative nuclear magnetic resonance (qNMR) yield using 0.33 equiv of trimethoxybenzene as the internal standard.

e Reverse-phase high-performance liquid chromatography (HPLC) yields.

Decreasing the equivalents of PNO did not significantly change the yield; however, a small decrease was observed when 1.0 equiv was used, giving an optimum amount of 1.5 equiv (entries 7–9 in Table 1). PNO is a hygroscopic solid; we suggest that 1.5 equiv ensures that at least 1.0 equiv of reagent is present in the reaction. Although a slightly higher yield is reported in Table 1 with 2.0 equiv of PNO, repetition of entries 8 and 9 produced multiple results within ±5% of the yields detailed below and gave no advantage. Thus, we believe that 1.5 equiv is sufficient to provide the 1.0 equiv of reagent necessary to effect the transformation.

Using the optimized conditions, the reaction scope was investigated with pleasing yields and functional group tolerances (Scheme 2A). Alkyl chains, alkenes, and rings were tolerated, producing products **4a**, **4k**, and **4f**, in yields of 64, 70, and 61% respectively. Simple aryl rings also performed well with benzyl and naphthyl substituents (see products **4e** and **4h** in yields of 70 and 66%, respectively). Pleasingly, when scaled up to 1 mmol, product **4e** was isolated in 93% yield, a significant increase on the 0.25 mmol scale.

**Scheme 2:**
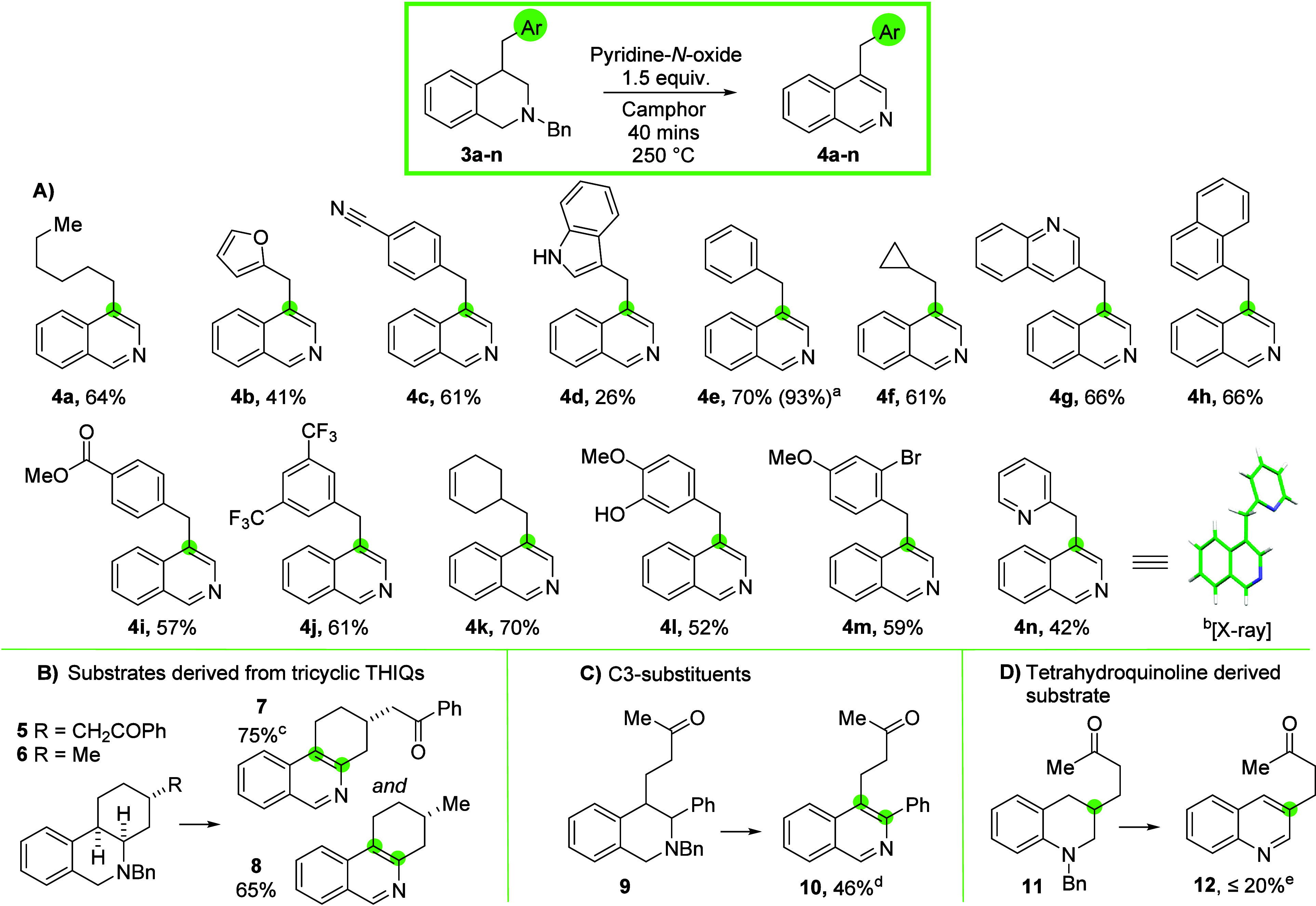
Scope of Substrates Derived from THIQs The reaction was performed on a 1.0 mmol scale. Determined by single-crystal X-ray diffraction.^20^ The reaction was performed on a 0.090 mmol scale. The reaction was performed on a 0.18 mmol scale. The reaction was performed on a 0.050 mmol scale. Reactions were performed on a 0.25 mmol scale sealed under air, unless otherwise stated, with the isolated yields.

Substrates containing electron-deficient aryl rings, such as esters, **4i** (57%), bis-*meta*-CF_3_ groups, **4j** (61%), and nitriles, **4c** (61%), gave reasonable yields. Aryl rings with electron-donating groups performed slightly less well. Compound **4l** with hydroxyl and ether groups gave a yield of 52%, but the related product **4m** with halogen and ether substituents performed marginally better, affording a 59% yield. Substrates with heterocyclic substituents produced mixed results, with good yields for a quinoline substituent, **4g** (66%) and moderate yields for pyridine and furan substituents, **4n** and **4b** (42 and 41%), respectively. An indole substituent fared poorly, affording 26% of product **4d**. No workup is required for these reactions, and upon cooling, the crude mixture can be dry loaded onto SiO_2_ and purified by flash column chromatography.

The rearomatization of tricyclic THIQs was probed with two annulated substrates (Scheme 2B). Pleasingly, rearomatized isoquinolines **7** and **8** were returned in good yields. A C3-aryl, C4-alkyl isoquinoline substrate was also synthesized and produced a reasonable yield of isoquinoline **10** (Scheme 2C). Finally, tetrahydroquinoline was trialed, which afforded product **12** in a disappointing yield (Scheme 2D), and this class of substrates was not pursued further.

The mechanism of the rearomatization of compound **1** was investigated (Table 2). A control experiment with PNO under an inert atmosphere (entry 2 in Table 2) reduced the yield to 52%, indicating that oxygen was, in part, necessary. Accordingly, omitting pyridine-*N*-oxide from the reaction still afforded some product, but in 21% yield (entry 3 in Table 2). This demonstrates that a background autoxidation reaction is present. Excluding both oxygen and pyridine-*N*-oxide from the reaction shut down reactivity (3%), which is consistent with this hypothesis (entry 4 inTable 2). Entry 5 demonstrates that atmospheric oxygen is sufficient for the transformation.

**Table 2:**
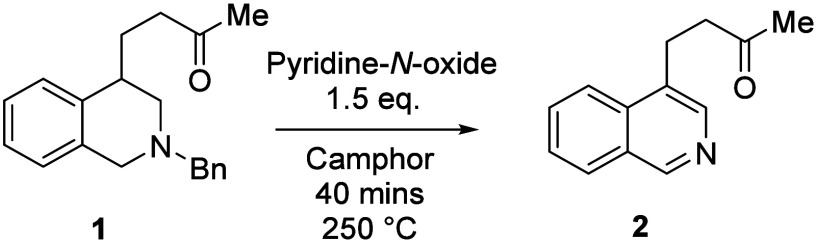
Effect of the Reaction Headspace^a^

entry	atmosphere	PNO (equiv)	yield of product **2** (%)^b^
1	air	1.5	69
2	argon	1.5	52
3	air	0.0	21
4	argon	0.0	3
5	oxygen	1.5	68

a Reactions were performed on a 0.25 mmol scale.

b Isolated yields.

Next, we wished to investigate the byproduct(s) produced from pyridine-*N*-oxide, which was challenging because of volatility considerations. Therefore, changing the oxidant to 4-phenyl pyridine-*N*-oxide (PPNO) allowed us to isolate a non-volatile byproduct, 4-phenyl pyridine, **13**, from the reaction mixture in a quantitative yield (Scheme 3). This observation shows the likely fate of PNO (pyridine) as it donates its oxygen. While a slightly elevated yield of product **2** (85%) was isolated when using PPNO, we found that widespread use of this reagent was less consistent than PNO with repeats under identical conditions.

**Scheme 3:**
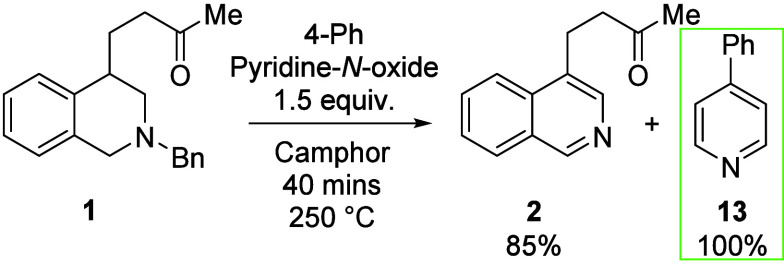
Investigation into the Fate of *N*-Oxide Reactions were performed on a 0.25 mmol scale sealed under air.

We attempted to determine the fate of the benzyl group lost upon aromatization. Two analogues of compound **1** with varying N-protecting groups were subjected to the aromatization sequence (Scheme 4). We found that altering the benzyl group decreased or maintained the yield of the reaction; however, in no case could any N-protecting group decomposition products be isolated or observed. Interestingly, the *N*-Me analogue of compound **1** did not perform well under these conditions, delivering the product in 19% yield (see the Supporting Information). In an attempt to probe the recipient of the PNO oxygen atom, the *N*-oxide derivative of compound **1** (**16**, a putative intermediate) was synthesized (see the Supporting Information). However, the use of compound **16** as a substrate provided a low amount (33%) of product **2**, implying that it is not a major intermediate in the reaction.

**Scheme 4:**
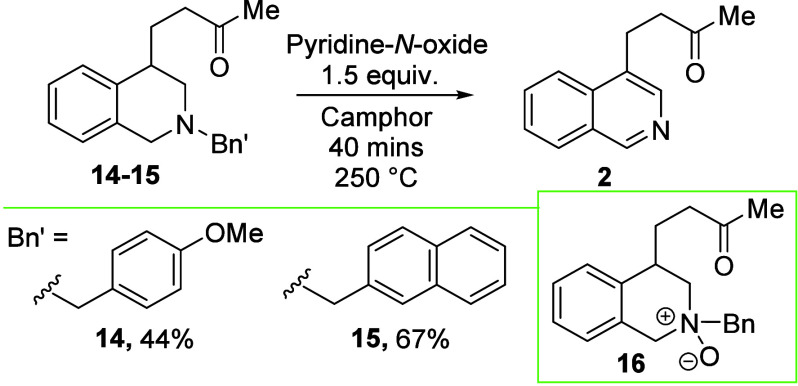
Investigations into Modification of the N Group Reactions were performed on a 0.25 mmol scale sealed under air.

Next, the aromatization of compound **1** was spiked with additives conventionally used as radical traps [(2,2,6,6-tetramethylpiperidin-1-yl)oxyl (TEMPO), 1,1-diphenylethylene, amylene, and butylated hydroxytoluene (BHT)], but none of them affected the yield significantly (see the Supporting Information).^21−23^

For further mechanistic work, we subjected other potential intermediates to the reaction both with and without PNO present. However, the synthesis of these substituted intermediates proved challenging, and a simpler model system was introduced without a side chain at C4 (Scheme 5). Initially, compound **17**, a model for compound **1**, was subjected to the key reaction and afforded a 76% yield of isoquinoline **23** with PNO present and a 31% yield without PNO. These values are close to those obtained with the substituted system of compound **1** (69 and 21%) and gave us confidence to continue with other derivatives of compound **17**.

**Scheme 5:**
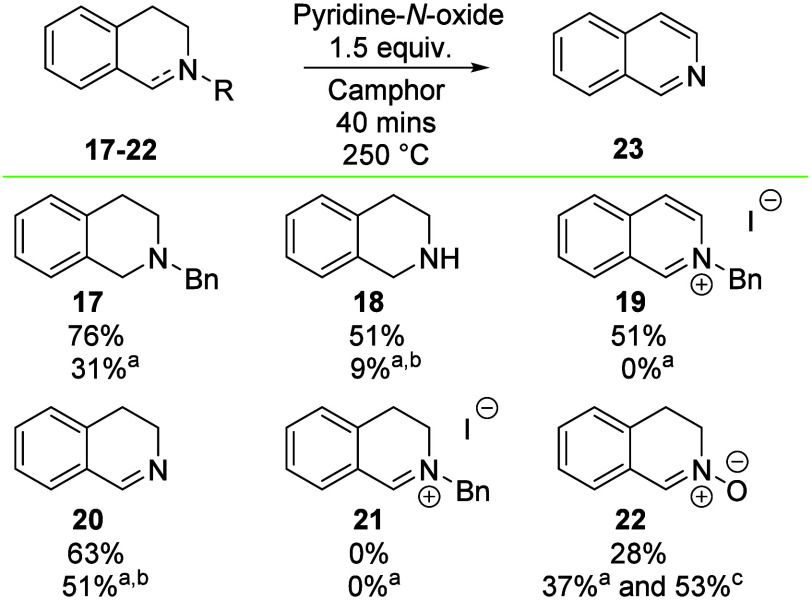
Testing of Further Putative Intermediates The reaction was performed in the absence of PNO. qNMR yield using 0.33 equiv of trimethoxybenzene as the internal standard. In the absence of PNO but with 1.5 equiv of pyridine. Reactions were performed on a 0.25 mmol scale sealed under air, with isolated yields, unless otherwise specified.

To probe the timing of benzyl group removal, debenzylated THIQ **18** was tested and afforded a 51% yield of product **23**. Interestingly, fully aromatized *N*-benzyl isoquinolinium salt **19** also afforded a 51% yield of product **23**.

Both of these results represent >20% reduction in the yield compared to *N*-benzyl substrate **17** (Scheme 5). Substrates **18** and **19** give conflicting views of the timing of Bn group removal versus heterocycle oxidation, yet both still give the product in a reasonable but reduced yield.

Next, we found that subjection of imine **20** to the reaction conditions afforded product **23** in a good yield (63%), which suggests that compound **20** is a realistic intermediate in the reaction. Intriguingly, *N*-benzyl iminium **21** gave no isoquinoline formation when subjected to the reaction conditions (with or without PNO). This strongly suggests that iminium **21** is not an intermediate in the reaction. Given the viability of compound **20**, we considered the mechanism by which it might be oxidized to isoquinoline **23**, and we suggest nitrone **22** as a possible derivative that then could go on to form an isoquinoline. However, when nitrone **22** was subjected to the reaction conditions (with or without PNO), the reaction outcome was disappointing. Given that the transformation of compound **22** to compound **23** is an elimination of water and that PNO is reduced to pyridine *in situ* (Scheme 3), we repeated this reaction with 1.5 equiv of pyridine and were pleased to see the yield increase to 53%.

These studies show it is likely that there is more than one productive pathway for aromatization under high reaction temperatures. Moreover, many of the key intermediates shown give reasonable amounts of product by autoxidation in the absence of PNO. The data in Table 2 suggest to us that there is a major (PNO) pathway and a supplementary air-promoted pathway operative. In Scheme 6, we propose one possible mechanism that is consistent with our results: that the first oxidation to an imine and benzyl removal happens as a concerted process, forming imine **20** with the elimination of toluene (possibly via a retro-ene process).^24,25^ This would explain why the NMe analogue of compound **1** is poorly yielding under the main reaction conditions, as it is unable to undergo this reaction. We note that a benzylic radical at C1, formed by autoxidation, could also fragment to form the same imine and a stabilized benzyl radical.

**Scheme 6:**
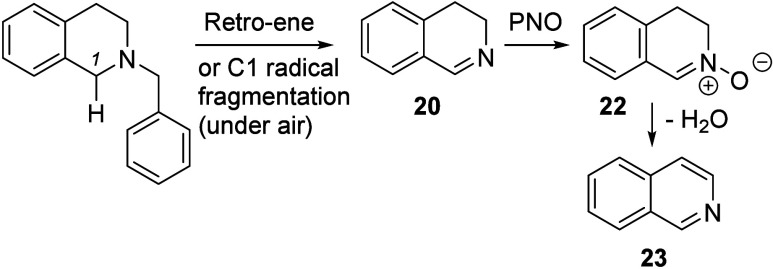
Proposed Mechanism for the Aromatization

During the methodology development, we found that substrates with substituents at C1 did not perform well under the reaction conditions, producing trace products (see the Supporting Information), which points to key reactivity occurring at the C1 position, which is consistent with our proposition.

We propose that imine **20** is a reaction intermediate and is oxidized to product **23** with or without PNO. While it is unclear how this happens, we suggest the PNO-promoted conversion of imine **20** into nitrone **22** (forming pyridine). While the subjection of compound **22** to the reaction conditions afforded a disappointing yield of product **23**, the addition of pyridine gave a 53% yield, which is evidence for compound **22** to be considered a legitimate intermediate (Scheme 6). Here, the base (pyridine)-promoted elimination of water from compound **22** would form the isoquinoline.^26^ We speculate that the mechanism of this step might involve tautomerization of compound **22** into a quinodimethane intermediate.

In conclusion, we described an oxidative transformation for synthesizing C4-substituted isoquinolines from readily available THIQ precursors. The reaction is versatile, tolerating a wide range of functionality, and allows access to complex unsaturated aromatic tricyclic scaffolds. We explored the reaction through putative intermediates and suggested a mechanism.

## Data Availability

The data underlying this study are available in the published article and its Supporting Information.

## Supporting Information Available

Experimental details and analytical data on starting materials and products, copies of NMR spectra, an extended optimization table, and X-ray diffraction data (PDF)
