# Supplementary material for: Oxidative Rearomatization of Tetrahydroisoquinolines Promoted by Pyridine-N-oxide
Source: Org Lett. 2024 Sep 23;26(39):8377–81. doi: 10.1021/acs.orglett.4c03186 (PMC11459517; doi:10.1021/acs.orglett.4c03186)
Supplement: Supplementary file 1 — ol4c03186_si_001.pdf [file ol4c03186_si_001.pdf]

Supporting Information for

Oxidative Rearomatization of Tetrahydroisoquinolines promoted by Pyridine-*N*-Oxide

Timothy C. Jenkins,[a] Darren L. Poole,[b] Timothy J. Donohoe[a]*

[a] Department of Chemistry, University of Oxford, Chemistry Research Laboratory, Mansfield Road, Oxford, OX1 3TA, UK.

*E-mail: timothy.donohoe@chem.ox.ac.uk

[b] Molecular Modalities Discovery, GlaxoSmithKline Medicines Research Centre, Stevenage, SG1 2NY

Contents:

1. **General Experimental Information** **S2**
2. **Experimental Procedures** **S3**
   - **2.1 General Procedures** **S3**
   - **2.2 Synthesis of Tetrahydroisoquinolines** **S4**
   - **2.3 Rearomatization Products** **S17**
   - **2.4 Synthesis of Compounds for Mechanistic Studies** **S29**
   - **2.5 Synthesis of Starting Material Salts and Electrophiles** **S35**
3. **Extended Optimisation Table and Failed Substrates** **S39**
4. **NMR Spectra** **S45**
5. **Single Crystal X-Ray Data** **S99**
6. **References** **S107**

1. General Experimental Information

Chemicals were purchased from commercial suppliers (Sigma Aldrich, Fluorochem and Alfa Aesar) and used without further purification. Compounds **13**, **18** and **20** are commercially available and were purchased from the above suppliers. Solvents were dried either by filtration through activated alumina purification columns or over pre-activated 4 Å molecular sieves. TLC analysis was performed using pre-coated silica gel aluminium sheets (Merck TLC Silica Gel 60 F_{254}), with spots visualised either under UV light (λ = 254 nm) or by staining with a solution of $KMnO_4$. Flash column chromatography was performed using Merck Geduran® Silica Gel 60 (40-63 μm). Deactivated Silica refers to pre-basified silica: silica (400 g), pentane (250 mL), Et_2O (250 mL) and NEt_3 (5 mL) were combined in a 1 L beaker and stirred for 15 mins. The solvent was allowed to evaporate overnight and the resulting silica could be stored in a sealed bottle for a few weeks.[15] Reactions were carried out in standard glassware under an atmosphere of air unless stated otherwise. Room temperature (rt) refers to 20-25 °C. Temperatures of 0 °C were obtained using an ice/water bath. Temperatures elevated above r.t. were achieved using a heating block mounted on an IKA basic stirrer hotplate. Where reactions were performed in a round bottomed flask, the block took the form of a commercial Asynt DrySyn® Multi-E kit heating block. Reactions performed in a microwave vial were heated using a cuboidal aluminium block manufactured in-house for Biotage® 5 mL glass microwave vials. The block has dimensions of 47.5 by 75.0 by 50.0 mm, with 6 bores of 17.0 mm diameter, and depth 30.0 mm.

NMR spectroscopy was carried out using a Bruker 400, 500 or 600 MHz spectrometer in the deuterated solvent stated, using the residual non-deuterated solvent signal as an internal reference. Chemical shifts, δ, are recorded to the nearest 0.01 ppm (^{1}H NMR) or 0.1 ppm (^{13}C NMR) and referenced to residual solvent peaks. Splitting patterns are classified as a singlet (s), broad singlet (br s), doublet (d), triplet (t), quartet (q), hextet (h), heptet (hept) multiplet (m) or combinations thereof. Chemical shifts and splitting patterns were recorded as observed. ^{1}H NMR yields were calculated by using trimethoxy benzene as an internal standard, added to the crude reaction mixture after work up and concentration *in vacuo*. Quantitative ^{1}H NMR spectra were recorded using a Bruker AVIII HD 400 Spectrometer with a 25 s relaxation time and samples measured in $CDCl_3$.

Reverse phase HPLC yields were obtained using a Dionex UltiMate 3000 system equipped with UV-Vis variable wavelength detector, fitted with an Agilent InfinityLab Poroshell 120 EC-C18 column (0.46 cm x 150 mm, 4 µm pore size)

LRMS were obtained using an Agilent 6120 Quadrupole LC-MS spectrometer with electro spray ionisation conditions (ESI). Electrospray ionisation (ESI) HRMS were recorded on a Thermo Exactive orbitrap spectrometer equipped with a Waters Equity LC system, with a flow rate of 0.2 mL/min using water:methanol:formic acid (10:89.9:0.1) as eluent. The system uses a heated electrospray ionisation (HESI-II) probe for ESI^+ and has a resolution of 50,000 FWHM under conditions for maximum sensitivity, with an accuracy of better than 5 ppm for 24 h following external calibration on the day of analysis. The mass reported is that containing the most abundant isotopes, with each value rounded to 4 decimal places and within 5 ppm of the calculated mass.

Single crystal X-ray data collection and structure determination were performed by Timothy C. Jenkins in the Chemistry Research Laboratory, University of Oxford. Crystals were mounted on MiTeGen loops using perfluoropolyether oil and rapidly transferred to a goniometer head on a diffractometer fitted with an Oxford CryoSystems CryoStream open-flow nitrogen cooling device[1]. Data collections were carried out at 150 K using an (Rigaku) Oxford Diffraction Supernova A diffractometer using mirror-monochromated Cu Kα radiation (λ = 1.54184 Å) and data were processed using CryAlisPro. The structure was solved using charge-flipping algorithm SUPERFLIP[2] and refined by full-matrix least squares using CRYSTALS[3 4].

Melting points were measured using a Gallenkamp Griffin melting point apparatus. Fourier Transform Infrared (FTIR) spectra were recorded using a Bruker Tensor 27 spectrometer with absorption maxima quoted in wavenumbers (cm^{-1}). Chiral compounds and images depicting chiral compounds refer to racemic mixtures unless stated otherwise. Compounds were named using Perkin Elmer ChemDraw software. All chiral molecules depicted are racemic.

2.1 General Procedures

General procedure A, Reductive functionalisation of isoquinolinium iodide:

Tetrahydroisoquinoline (THIQ) starting materials for our transformations were synthesised according to a procedure first reported by Kischkewitz and co-workers: *N*-benzyl isoquinolinium iodide (**19**) (170 mg, 0.50 mmol, 1.0 equiv.) was dissolved in MeCN (0.40 mL)

with 5:2 HCO_2H:NEt_3 (0.17 mL, 2.0 mmol, 4.0 equiv.) and an electrophile (0.50 mmol, 1.0 equiv.). The solution was heated to 80 °C for 18 h, cooled to r.t., diluted with CH_2Cl_2, and washed with sat. aq. Na_2CO_3. The layers were partitioned, the aqueous layer washed twice more with CH_2Cl_2, then the organic layers combined, dried over $MgSO_4$, filtered under gravity and concentrated *in vacuo*.[5]

General Procedure B, THIQ oxidation by Pyridine-*N*-oxide:

Tetrahydroisoquinoline (0.25 mmol, 1.0 equiv.) was placed in a microwave vial with pyridine-*N*-oxide (36 mg, 0.38 mmol, 1.5 equiv.) and camphor (0.20 g), sealed with a crimped cap, heated to 250 °C for 40 min then cooled to r.t.

2.2 Synthesis of Tetrahydroisoquinolines

4-(2-Benzyl-1,2,3,4-tetrahydroisoquinolin-4-yl)butan-2-one (1):

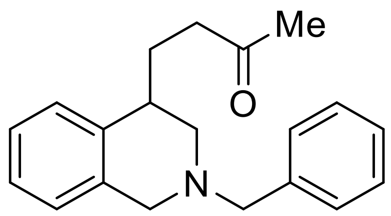

The title compound was prepared according to **General Procedure A** using MVK (41 µL, 0.500 mmol, 1.00 equiv.) as the electrophile. Purification by flash column chromatography (10% EtOAc in pentane) afforded **1** as a pale-yellow oil (85 mg, 58%).

^{1}H NMR (600 MHz, $CDCl_3$): δ_H = 7.39 (2H, d, *J* = 6.8 Hz), 7.34 (2H, t, *J* = 7.6 Hz), 7.30 – 7.26 (1H, m), 7.21 – 7.14 (2H, m), 7.12 (1H, td, *J* = 7.3, 1.8 Hz), 7.00 (1H, d, *J* = 7.5 Hz), 3.81 (1H, d, *J* = 14.8 Hz), 3.75 (1H, d, *J* = 13.0 Hz), 3.56 (1H, d, *J* = 13.0 Hz), 3.48 (1H, d, *J* = 14.8 Hz), 2.80 (1H, dq, *J* = 8.5, 4.1 Hz), 2.72 – 2.66 (1H, m), 2.54 (1H, dd, *J* = 11.6, 4.2 Hz), 2.39 (1H, ddd, *J* = 16.2, 9.4, 6.4 Hz), 2.28 (1H, ddd, *J* = 16.7, 9.5, 5.4 Hz), 2.07 (3H, s), 2.06 – 1.95 (2H, m); **^{13}C NMR** (151 MHz, $CDCl_3$): δ_C = 209.1, 138.8, 138.4, 135.1, 129.2, 128.5, 128.4, 127.3, 126.6, 126.4, 126.0, 62.9, 56.9, 53.9, 41.4, 38.0, 30.0, 30.0. Spectroscopic data were consistent with the literature data for this compound.[5a]

2-Benzyl-4-hexyl-1,2,3,4-tetrahydroisoquinoline (3a):

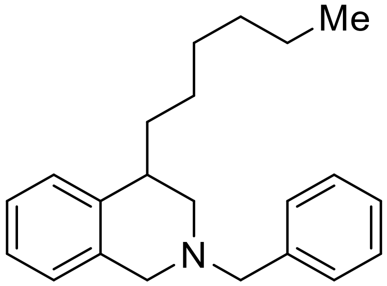

The title compound was prepared according to **General Procedure A** using hexanal (60.0 µL, 0.500 mmol, 1.00 equiv.) as the electrophile. Purification with flash column chromatography (3% EtOAc in Pentane) afforded **3a** as a pale orange oil (115 mg, 75%).

^{1}H NMR (600 MHz, $CDCl_3$): δ_H = 7.40 (1H, d, *J* = 7.5 Hz), 7.33 (2H, t, *J* = 7.5 Hz), 7.30 – 7.27 (1H, m), 7.20 – 7.12 (2H, m), 7.10 (1H, t, *J* = 7.4 Hz), 6.98 (1H, d, *J* = 7.6 Hz), 3.79 – 3.73 (2H, m), 3.58 (1H, d, *J* = 13.2 Hz), 3.51 (1H. d, *J* = 14.8 Hz), 2.78 (1H, s), 2.73 – 2.67 (1H, m), 2.66 – 2.59 (1H, m), 1.77 (1H, dq, *J* = 17.4, 5.9 Hz), 1.67 (1H, dt, *J* = 15.0, 7.3 Hz), 1.34 – 1.18 (8H, m), 0.88 (3H, t, *J* = 6.9 Hz); **^{13}C NMR** (151 MHz, $CDCl_3$): δ_C = 139.5, 138.8, 134.9, 129.0, 128.2 (2C), 127.1, 126.4, 126.1, 125.5, 62.9, 56.8, 54.2, 38.7, 36.0, 31.8, 29.5, 27.4, 22.7, 14.1. Spectroscopic data were consistent with the literature data for this compound.[5a]

2-Benzyl-4-(furan-2-ylmethyl)-1,2,3,4-tetrahydroisoquinoline (3b):

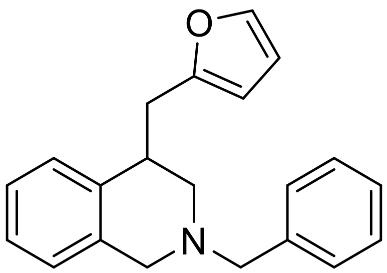

The title compound was prepared according to **General Procedure A** using furfural (42.0 µL, 0.500 mmol, 1.00 equiv.) as the electrophile. Purification with flash column chromatography (3% EtOAc in Pentane) afforded **3b** as a pale orange oil (136 mg, 90%).

^{1}H NMR (600 MHz, $CDCl_3$): δ_H = 7.41 (2H, d, *J* = 7.5 Hz), 7.35 (2H, t, *J* = 7.6 Hz), 7.31 – 7.27 (2H, m), 7.18 – 7.08 (3H, m), 7.01 (1H, dd, *J* = 7.0, 1.9 Hz), 6.21 (1H, dd, *J* = 3.1, 1.9 Hz), 5.69 (1H, d, *J* = 3.1 Hz), 3.85 (1H, d, *J* = 14.8 Hz), 3.73 (1H, d, *J* = 13.0 Hz), 3.57 (1H, d, *J* = 12.9 Hz), 3.45 (1H, d, *J* = 14.8 Hz), 3.16 – 3.06 (2H, m), 2.99 – 2.92 (1H, m), 2.79 (1H,

d, J = 11.5 Hz), 2.50 – 2.45 (1H, m); **^{13}C NMR** (151 MHz, $CDCl_3$): δ_C = 154.8, 141.1, 138.9, 138.1, 135.3, 129.4, 128.8, 128.4, 127.2, 126.6, 126.3, 126.1, 110.2, 106.7, 63.0, 56.8, 53.9, 38.6, 35.1. Spectroscopic data were consistent with the literature data for this compound.[5a]

4-((2-Benzyl-1,2,3,4-tetrahydroisoquinolin-4-yl)methyl)benzonitrile (3c):

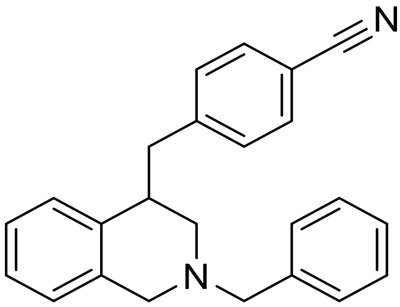

The title compound was prepared according to **General Procedure A** using 4-CN benzaldehyde (66 mg, 0.50 mmol, 1.0 equiv.) as the electrophile. Purification with flash column chromatography (7% EtOAc in Pentane) afforded **3c** as a colourless solid (87 mg, 51%).

^{1}H NMR (600 MHz, $CDCl_3$): δ_H = 7.46 – 7.42 (2H, m), 7.42 – 7.34 (5H, m), 7.22 – 7.15 (2H, m), 7.14 (1H, d, J = 2.8 Hz), 7.06 (1H, dd, J = 6.6, 2.3 Hz), 6.98 (2H, d, J = 7.9 Hz), 3.96 (1H, d, J = 14.8 Hz), 3.79 (1H, d, J = 12.7 Hz), 3.49 (1H, d, J = 14.9 Hz), 3.45 (1H, d, J = 12.7 Hz), 3.06 (1H, dd, J = 12.8, 10.2 Hz), 3.00 – 2.92 (2H, m), 2.69 (1H, dt, J = 11.5, 1.9 Hz), 2.32 (1H, dd, J = 11.6, 3.7 Hz); **^{13}C NMR** (151 MHz, $CDCl_3$): δ_C = 146.7, 138.5, 137.8, 135.1, 132.0, 130.1, 129.7, 128.7, 128.4, 127.3, 126.6, 126.3, 126.2, 119.2, 109.7, 62.8, 57.1, 52.1, 42.9, 41.3. Spectroscopic data were consistent with the literature data for this compound.[5a]

4-((1H-Indol-3-yl)methyl)-2-benzyl-1,2,3,4-tetrahydroisoquinoline (3d):

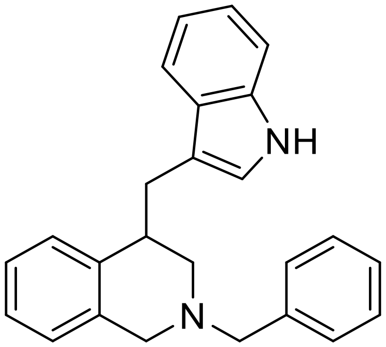

The title compound was prepared according to **General Procedure A** using indole-3-carbaldehyde (73.0 mg, 0.500 mmol, 1.00 equiv.) as the electrophile. Purification with flash

column chromatography (5% EtOAc in pentane) afforded **3d** as a fluffy, colourless solid (112 mg, 64%).

^{1}H NMR (600 MHz, $CDCl_3$): δ_H = 7.86 (1H, s), 7.78 (1H, d, *J* = 7.8 Hz), 7.48 (2H, d, *J* = 7.0 Hz), 7.43 – 7.38 (3H, m), 7.38 – 7.34 (2H, m), 7.29 – 7.16 (4H, m), 7.09 (1H, d, *J* = 7.6 Hz), 6.44 (1H, d, *J* = 2.2 Hz), 3.95 (1H, d, *J* = 14.7 Hz), 3.79 (1H, d, *J* = 12.9 Hz), 3.58 – 3.49 (2H, m), 3.23 (1H, dd, *J* = 15.2, 11.9 Hz), 3.19 – 3.12 (2H, m), 2.90 (1H, dt, *J* = 11.5, 1.9 Hz), 2.41 (1H, dd, *J* = 11.4, 3.8 Hz); **^{13}C NMR** (151 MHz, $CDCl_3$): δ_C = 139.2, 139.1, 136.4, 135.2, 129.6, 129.0, 128.4, 127.7, 127.2, 126.5, 126.3, 125.9, 122.8, 121.9, 119.3, 119.0, 115.0, 111.2, 63.0, 57.1, 53.3, 39.8, 32.3; **IR** (neat) cm^{-1}: 3422, 1493, 1455, 1342, 1093, 740, 700; **HRMS** (ESI^+) m/z: $[M+H]^+$ calcd for $C_{25}H_{25}N_2$ 353.2012; found at 353.2007; **m.p.** = 46-47 °C.

2,4-Dibenzyl-1,2,3,4-tetrahydroisoquinoline (3e):

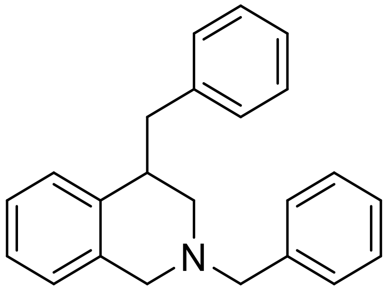

The title compound was prepared according to **General Procedure A** but at the 1.0 mmol scale, using benzaldehyde (100 µL, 1.00 mmol, 1.00 equiv.) as the electrophile. Purification with flash column chromatography (3% EtOAc in Pentane) afforded **3e** as a colourless solid (232 mg, 74%).

^{1}H NMR (600 MHz, $CDCl_3$): δ_H = 7.46 – 7.41 (2H, m), 7.40 – 7.35 (2H, m), 7.33 (1H, dt, *J* = 5.8, 2.0 Hz), 7.25 – 7.11 (6H, m), 7.05 – 6.99 (3H, m), 3.88 (1H, d, *J* = 14.8 Hz), 3.72 (1H, d, *J* = 12.9 Hz), 3.57 – 3.52 (1H, m), 3.45 (1H, d, *J* = 14.9 Hz), 3.06 – 2.96 (3H, m), 2.78 (1H, d, *J* = 11.5 Hz), 2.38 (1H, d, *J* = 11.3 Hz); **^{13}C NMR** (151 MHz, $CDCl_3$): δ_C = 141.2, 138.8 (2C), 135.2, 129.6, 129.5, 128.9, 128.5, 128.4, 127.3, 126.6, 126.3, 126.0 (2C), 63.1, 56.9, 53.1, 42.9, 41.6. Spectroscopic data were consistent with the literature data for this compound.[5a,b]

2-Benzyl-4-(cyclopropylmethyl)-1,2,3,4-tetrahydroisoquinoline (3f):

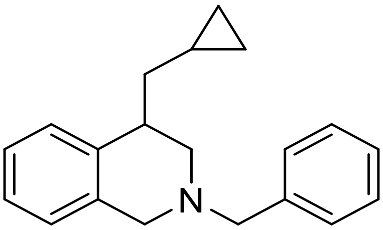

The title compound was prepared according to **General Procedure A** using cyclopropane carbaldehyde (35.0 µL, 0.500 mmol, 1.00 equiv.) as the electrophile. Purification with flash column chromatography (5% EtOAc in Pentane) afforded **3f** as a pale orange oil (103 mg, 74%).

^{1}H NMR (600 MHz, $CDCl_3$): δ_H = 7.42 (2H, d, *J* = 6.9 Hz), 7.40 – 7.31 (2H, m), 7.31 – 7.26 (1H, m), 7.19 (1H, dd, *J* = 7.7, 1.5 Hz), 7.15 (1H, td, *J* = 7.4, 1.4 Hz), 7.10 (1H, td, *J* = 7.4, 1.5 Hz), 7.01 – 6.96 (1H, m), 3.80 – 3.71 (2H, m), 3.64 (1H, d, *J* = 13.0 Hz), 3.48 (1H, d, *J* = 14.8 Hz), 2.97 – 2.89 (2H, m), 2.74 – 2.68 (1H, m), 1.73 (1H, ddd, *J* = 13.7, 9.4, 6.9 Hz), 1.58 (1H, ddd, *J* = 13.8, 7.0, 4.2 Hz), 0.72 – 0.62 (1H, m), 0.46 – 0.37 (2H, m), 0.05 – 0.00 (2H, m); **^{13}C NMR** (151 MHz, $CDCl_3$): δ_C = 139.3, 138.9, 135.0, 129.2, 128.5, 128.4, 127.2, 126.5, 126.1, 125.6, 63.2, 56.8, 54.8, 41.3, 39.7, 9.6, 5.5, 4.3. Spectroscopic data were consistent with the literature data for this compound.[5a]

3-((2-Benzyl-1,2,3,4-tetrahydroisoquinolin-4-yl)methyl)quinoline (3g):

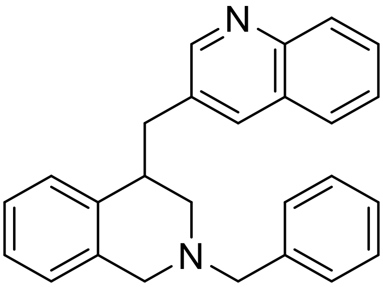

The title compound was prepared according to **General Procedure A** using quinoline-3-carbaldehyde (79.0 mg, 0.500 mmol, 1.00 equiv.) as the electrophile. Purification with flash column chromatography (20% EtOAc in pentane) afforded **3g** as a pale orange oil (146 mg, 80%).

^{1}H NMR (600 MHz, $CDCl_3$): δ_H = 8.76 (1H, d, *J* = 2.2 Hz), 8.07 (1H, d, *J* = 8.4 Hz), 7.70 – 7.59 (2H, m), 7.54 – 7.48 (2H, m), 7.45 – 7.41 (2H, m), 7.40 – 7.34 (3H, m), 7.24 – 7.13 (3H, m), 7.08 – 7.03 (1H, m), 3.94 (1H, d, *J* = 14.8 Hz), 3.76 (1H, d, *J* = 12.9 Hz), 3.54 – 3.45 (2H,

m), 3.25 – 3.18 (1H, m), 3.14 (1H, dd, *J* = 13.7, 4.3 Hz), 3.10 – 3.04 (1H, m), 2.79 (1H, dt, *J* = 11.7, 1.9 Hz), 2.39 (1H, dd, *J* = 11.6, 3.9 Hz); **^{13}C NMR** (151 MHz, $CDCl_3$): δ_C = 152.6, 146.9, 138.8, 138.0, 135.5, 135.2, 133.8, 129.6, 129.3, 128.9, 128.8, 128.5, 128.1, 127.6, 127.5, 126.7, 126.6, 126.4, 126.3, 63.0, 57.0, 52.8, 41.3, 40.3; **IR** (neat) cm^{-1}: 1494, 1454, 911, 793, 745, 701; **HRMS** (ESI^+) m/z: $[M+H]^+$ calcd for $C_{26}H_{25}N_2$ 365.2012; found at 365.2003.

2-Benzyl-4-(naphthalen-1-ylmethyl)-1,2,3,4-tetrahydroisoquinoline (3h):

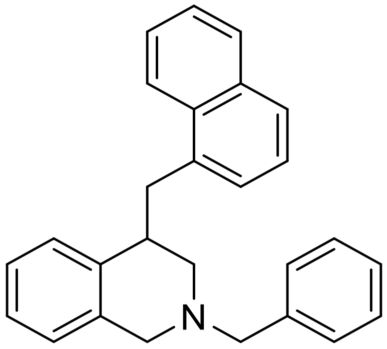

The title compound was prepared according to **General Procedure A** using 1-naphthaldehyde (78.0 mg, 0.500 mmol, 1.00 equiv.) as the electrophile. Purification with flash column chromatography (1% EtOAc in pentane) afforded **3h** as a pale orange solid (124 mg, 68%).

^{1}H NMR (600 MHz, $CDCl_3$): δ_H = 8.37 – 8.31 (1H, m), 7.92 – 7.86 (1H, m), 7.73 (1H, t, *J* = 6.3 Hz), 7.60 – 7.53 (1H, m), 7.53 – 7.46 (3H, m), 7.45 – 7.39 (2H, m), 7.39 – 7.34 (2H, m), 7.28 (1H, ddd, *J* = 8.6, 5.3, 1.8 Hz), 7.24 (1H, d, *J* = 6.8 Hz), 7.20 (1H, t, *J* = 7.5 Hz), 7.07 (1H, t, *J* = 6.1 Hz), 7.03 – 6.96 (1H, m), 4.00 – 3.93 (1H, m), 3.72 – 3.58 (2H, m), 3.56 – 3.47 (2H, m), 3.46 – 3.37 (1H, m), 3.22 (1H, s), 2.88 – 2.82 (1H, m), 2.38 – 2.33 (1H, m); **^{13}C NMR** (151 MHz, $CDCl_3$): δ_C = 139.0, 138.8, 137.0, 135.2, 134.1, 132.4, 129.5, 129.0, 128.8, 128.5, 128.0, 127.3, 126.9, 126.7, 126.4, 126.1, 125.9, 125.6, 125.5, 124.2, 63.2, 56.8, 53.4, 40.4, 39.9; **IR** (neat) cm^{-1}: 3062, 1493, 1454, 1395, 1367, 1344, 1262, 1237, 1198, 1139, 1088, 1059, 1026, 963, 949, 920, 869, 857, 818, 799, 791, 781, 758, 747, 731, 702; **HRMS** (ESI^+) m/z: $[M+H]^+$ calcd for $C_{27}H_{26}N$ 364.2060; found at 364.2067; **m.p.** = 79-81 °C.

Methyl 4-((2-benzyl-1,2,3,4-tetrahydroisoquinolin-4-yl)methyl)benzoate (3i):

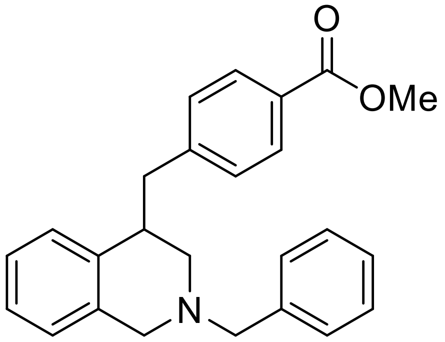

The title compound was prepared according to **General Procedure A** using methyl terephthalaldehyde (74 mg, 0.50 mmol, 1.0 equiv.) as the electrophile. Purification with flash column chromatography (3% EtOAc in Pentane) afforded **3i** as a colourless solid (99 mg, 53%).

^{1}H NMR (600 MHz, $CDCl_3$): δ_H = 7.92 – 7.87 (2H, m), 7.45 – 7.42 (2H, m), 7.42 – 7.34 (3H, m), 7.22 – 7.14 (3H, m), 7.08 – 7.02 (3H, m), 3.96 – 3.91 (4H, m), 3.76 (1H, d, J = 12.8 Hz), 3.50 (2H, dd, J = 18.3, 13.8 Hz), 3.09 (1H, dd, J = 13.8, 11.2 Hz), 3.05 – 2.98 (2H, m), 2.75 (1H, dt, J = 11.6, 1.9 Hz), 2.36 (1H, dd, J = 11.6, 3.7 Hz); **^{13}C NMR** (151 MHz, $CDCl_3$): δ_C = 167.2, 146.7, 138.7, 138.3, 135.2, 129.7, 129.6, 129.5, 128.8, 128.5, 127.9, 127.4, 126.6, 126.3, 126.1, 63.0, 57.0, 52.8, 52.1, 42.9, 41.4; Spectroscopic data were consistent with the literature data for this compound.[5a]

2-Benzyl-4-((3,5-bis(trifluoromethyl)cyclohexyl)methyl)-1,2,3,4-tetrahydroisoquinoline (3j):

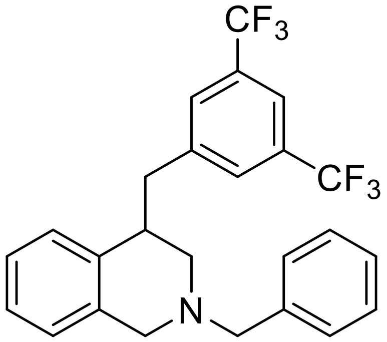

The title compound was prepared according to **General Procedure A** using 3,5-bis CF_3 benzaldehyde (121 mg, 0.500 mmol, 1.00 equiv.) as the electrophile. Purification with flash column chromatography (10% EtOAc in pentane) afforded **3j** as a pale orange oil (130 mg, 57%).

^{1}H NMR (600 MHz, $CDCl_3$): δ_H = 7.71 (1H, d, *J* = 2.0 Hz), 7.51 – 7.48 (2H, m), 7.41 – 7.38 (2H, m), 7.38 – 7.33 (2H, m), 7.32 – 7.28 (1H, m), 7.20 – 7.13 (2H, m), 7.08 – 7.01 (2H, m), 3.91 (1H, d, *J* = 14.9 Hz), 3.72 (1H, d, *J* = 12.9 Hz), 3.56 (1H, d, *J* = 12.9 Hz), 3.46 (1H, d, *J* = 14.9 Hz), 3.19 (1H, dd, *J* = 13.7, 9.8 Hz), 3.11 (1H, dd, *J* = 13.7, 4.8 Hz), 3.06 – 2.99 (1H, m), 2.70 (1H, ddd, *J* = 11.7, 2.6, 1.4 Hz), 2.45 (1H, dd, *J* = 11.6, 3.8 Hz); **^{13}C NMR** (151 MHz, $CDCl_3$): δ_C = 143.6, 138.3, 137.4, 135.2, 131.6 (q, *J* = 33.0 Hz), 129.6 (q, *J* = 3.6 Hz), 129.2, 128.8, 128.5, 127.6, 126.8, 126.5, 126.4, 123.5 (q, *J* = 272.7 Hz), 120.2 (hept, *J* = 3.9 Hz), 63.0, 56.7, 53.3, 42.6, 41.2; **^{19}F NMR** (377 MHz, $CDCl_3$): δ_F = -62.68; **IR** (neat) cm^{-1}: 1378, 1279, 1132, 1109, 778, 748, 723, 702, 683; **HRMS** (ESI$^+$) m/z: $[M+H]^+$ calcd for $C_{25}H_{22}F_6N$ 450.1651; found at 450.1653.

2-Benzyl-4-(cyclohex-3-en-1-ylmethyl)-1,2,3,4-tetrahydroisoquinoline (3k):

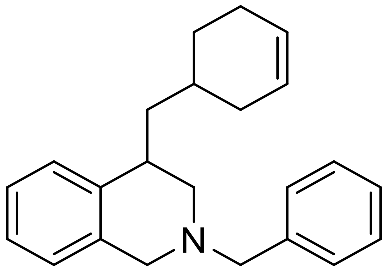

The title compound was prepared according to **General Procedure A** using 3-cyclohexene-1-carbaldehyde (55.0 mg, 0.500 mmol, 1.00 equiv.) as the electrophile. Purification with flash column chromatography (2% EtOAc in pentane) afforded **3k** as a 1:1 mixture of diastereomers as a pale orange oil (72.2 mg, 46%).

^{1}H NMR (600 MHz, $CDCl_3$): δ_H = 7.40 (2H, dd, *J* = 7.6, 3.0 Hz), 7.33 (2H, t, *J* = 7.5 Hz), 7.27 (1H, t, *J* = 7.3 Hz), 7.18 – 7.12 (2H, m), 7.12 – 7.08 (1H, m), 6.99 (1H, dd, *J* = 7.7, 3.3 Hz), 5.65 (2H, d, *J* = 3.2 Hz), 3.80 (2H, dd, *J* = 20.1, 13.1 Hz), 3.60 – 3.47 (2H, m), 2.89 (1H, ddd, *J* = 25.1, 10.0, 4.3 Hz), 2.74 (1H, ddd, *J* = 21.0, 11.5, 3.7 Hz), 2.61 – 2.50 (1H, m), 2.21 – 2.13 (1H, m), 2.11 – 1.92 (3H, m), 1.84 (2H, dddd, *J* = 31.5, 13.5, 10.3, 3.7 Hz), 1.76 – 1.61 (2H, m), 1.61 – 1.40 (2H, m), 1.24 (2H, dddd, *J* = 15.8, 12.4, 10.6, 5.7 Hz); **^{13}C NMR** (151 MHz, $CDCl_3$): δ_C = 140.0, 138.9, 135.0, 129.1, 129.1, 128.5, 128.5, 128.4, 127.4, 127.2, 127.1, 126.8, 126.6, 126.5, 126.4, 126.3, 125.6, 63.0, 62.8, 57.0, 56.9, 54.4, 53.8, 43.9, 43.2, 36.0, 35.8, 33.0, 31.4, 31.1, 30.9, 30.2, 28.1, 25.4, 25.2; **IR** (neat) cm^{-1}: 2914, 1702, 1494, 1454, 1144, 1095, 914, 743, 699, 654; **HRMS** (ESI$^+$) m/z: $[M+H]^+$ calcd for $C_{23}H_{28}N$ 318.2216; found at 318.2219.

5-((2-Benzyl-1,2,3,4-tetrahydroisoquinolin-4-yl)methyl)-2-methoxyphenol (3l):

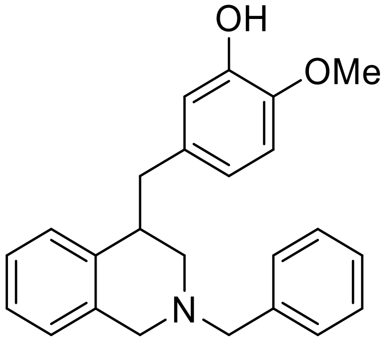

The title compound was prepared according to **General Procedure A** using isovanillin (76.0 mg, 0.500 mmol, 1.00 equiv.) as the electrophile. Purification with flash column chromatography (10% EtOAc in pentane) afforded **3l** as a pale orange oil (88.9 mg, 50%).

^{1}H NMR (600 MHz, $CDCl_3$): δ_H = 7.44 – 7.39 (2H, m), 7.38 – 7.34 (2H, m), 7.32 – 7.28 (1H, m), 7.19 (1H, dd, *J* = 7.6, 1.6 Hz), 7.18 – 7.14 (1H, m), 7.12 (1H, td, *J* = 7.3, 1.6 Hz), 7.00 (1H, dd, *J* = 7.4, 1.5 Hz), 6.71 (1H, d, *J* = 2.1 Hz), 6.67 (1H, d, *J* = 8.1 Hz), 6.45 (1H, dd, *J* = 8.1, 2.1 Hz), 5.52 (1H, br s), 3.86 (3H, s), 3.83 (1H, d, *J* = 14.7 Hz), 3.69 (1H, d, *J* = 12.9 Hz), 3.56 (1H, d, *J* = 12.9 Hz), 3.41 (1H, d, *J* = 14.8 Hz), 3.00 – 2.94 (1H, m), 2.90 (2H, dd, *J* = 7.4, 3.3 Hz), 2.77 (1H, ddd, *J* = 11.6, 2.9, 1.4 Hz), 2.39 (1H, dd, *J* = 11.5, 3.9 Hz); **^{13}C NMR** (151 MHz, $CDCl_3$): δ_C = 145.5, 145.0, 138.9, 138.8, 135.2, 134.5, 129.5, 128.8, 128.4, 127.2, 126.6, 126.3, 125.9, 121.0, 115.4, 110.6, 63.2, 56.8, 56.1, 53.4, 42.3, 41.5; **IR** (neat) cm^{-1}: 1591, 1510, 1454, 1274, 1238, 1130, 1029, 910, 784, 734, 701; **HRMS** (ESI^+) m/z: $[M+H]^+$ calcd for $C_{24}H_{26}NO_2$ 360.1958; found at 360.1949.

2-Benzyl-4-(2-bromo-4-methoxybenzyl)-1,2,3,4-tetrahydroisoquinoline (3m):

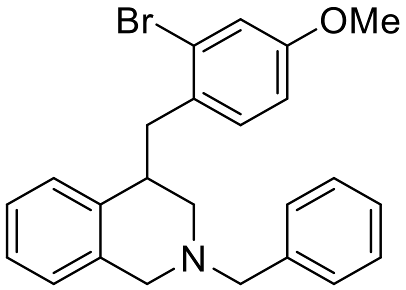

The title compound was prepared according to General Procedure C using 4-methoxy-2-bromo benzaldehyde (108 mg, 0.500 mmol, 1.00 equiv.) as the electrophile. Purification with flash column chromatography (2% EtOAc in pentane) afforded **3m** as an orange solid (150 mg, 71%).

^{1}H NMR (500 MHz, $CDCl_3$): δ_H = 7.47 – 7.42 (2H, m), 7.42 – 7.37 (3H, m), 7.37 – 7.33 (1H, m), 7.19 (1H, td, *J* = 7.5, 1.5 Hz), 7.15 (1H, td, *J* = 7.4, 1.5 Hz), 7.10 (1H, d, *J* = 2.6 Hz), 7.03 (1H, dd, *J* = 7.5, 1.5 Hz), 6.53 (1H, dd, *J* = 8.4, 2.6 Hz), 6.45 (1H, d, *J* = 8.5 Hz), 3.94 (1H, d, *J* = 14.8 Hz), 3.83 – 3.69 (4H, m), 3.52 (1H, d, *J* = 12.7 Hz), 3.45 (1H, d, *J* = 14.8 Hz), 3.15 – 3.06 (1H, m), 3.06 – 2.96 (2H, m), 2.84 – 2.76 (1H, m), 2.30 (1H, dd, *J* = 11.8, 3.8 Hz); **^{13}C NMR** (126 MHz, $CDCl_3$): δ_C = 158.5, 138.8, 135.1, 132.5, 132.2, 129.8, 129.1, 129.0, 128.5, 127.3, 126.4, 126.4, 126.0, 125.0, 118.0, 113.4, 63.1, 57.2, 55.6, 52.3, 42.2, 39.0; **IR** (neat) cm^{-1}: 1653, 1493, 1454, 1440, 1281, 1242, 1093, 1030, 911, 864, 842, 796, 751, 701, 639; **HRMS** (ESI^+) m/z: $[M+H]^+$ calcd for $C_{24}H_{25}{}^{79}BrNO$ 422.1114; found at 422.1108; **m.p.** = 56-58 °C.

2-Benzyl-4-(pyridin-2-ylmethyl)-1,2,3,4-tetrahydroisoquinoline (3n):

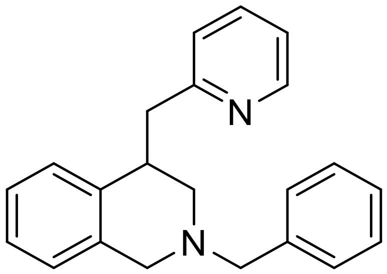

The title compound was prepared according to **General Procedure A** using 2-pyridinecarbaldehyde (48.0 µL, 0.500 mmol, 1.00 equiv.) as the electrophile. Purification with flash column chromatography (20% EtOAc in Pentane) afforded **3n** as a pale-yellow oil (215 mg, 68%).

^{1}H NMR (400 MHz, $CDCl_3$): δ_H = 8.56 (1H, ddd, *J* = 4.9, 1.9, 0.9 Hz), 7.43 – 7.38 (3H, m), 7.38 – 7.27 (3H, m), 7.23 – 7.10 (3H, m), 7.10 – 7.00 (2H, m), 6.68 (1H, d, *J* = 7.7 Hz), 3.91 (1H, d, *J* = 14.9 Hz), 3.73 (1H, d, *J* = 12.8 Hz), 3.48 (2H, dd, *J* = 14.0, 12.3 Hz), 3.42 – 3.32 (1H, m), 3.24 – 3.08 (3H, m), 2.70 (1H, ddd, *J* = 11.6, 2.7, 1.4 Hz), 2.41 (1H, dd, *J* = 11.6, 4.0 Hz); **^{13}C NMR** (101 MHz, $CDCl_3$): δ_C = 160.6, 149.5, 138.8, 138.6, 136.1, 135.1, 129.5, 128.9, 128.4, 127.2, 126.5, 126.3, 126.0, 124.4, 121.1, 63.0, 57.0, 53.4, 45.2, 39.6; Spectroscopic data were consistent with the literature data for this compound.[5a]

2-(*N*-Benzyl-1,2,3,4,4a,5,6,10b-octahydrophenanthridin-3-yl)-1-phenylethan-1-one (5):

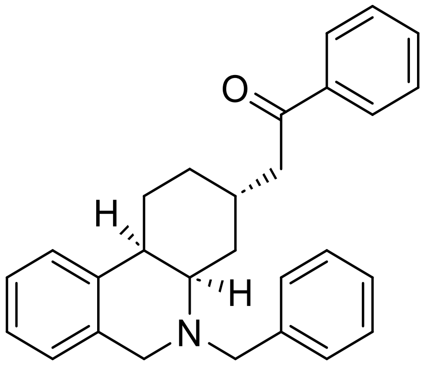

The title compound was prepared according to a literature procedure first reported by Kischkewitz and co-workers: 2-Benzyl-3-methylisoquinolin-2-ium iodide (**S5**) (180 mg, 0.50 mmol, 1.00 equiv.) was placed in a microwave vial with (E)-1-phenylpenta-2,4-dien-1-one (**S3**) (80 mg, 0.50 mmol, 1.0 equiv.), formic acid triethylamine complex 5:2 (168 μL, 2.00 mmol, 4.00 equiv.), and an aliquot of a premade solution of $(RhCp^*Cl_2)_2$ in MeCN (0.400 mL, 0.0100 mol%, 3.86 mg in 50.0 mL). The mixture was stirred at 80 $^{\circ}$C for 18 h, then cooled to r.t. and diluted with CH_2Cl_2. The solution was washed with sat. aq. Na_2CO_3, the layers partitioned and the aqueous phase extracted twice more with CH_2Cl_2. The combined organic layers were dried over $MgSO_4$, filtered under gravity and concentrated *in vacuo*. Purification by flash column chromatography (2% EtOAc in Pentane) afforded **5** as a pale orange solid (39 mg, 20%).

^{1}H NMR (500 MHz, $CDCl_3$): δ_H = 8.00 – 7.93 (2H, m), 7.57 – 7.50 (1H, m), 7.48 – 7.41 (4H, m), 7.37 – 7.32 (2H, m), 7.29 (1H, ddt, *J* = 7.4, 6.2, 1.5 Hz), 7.19 – 7.06 (3H, m), 6.90 (1H, d, *J* = 7.5 Hz), 4.50 (1H, d, *J* = 12.7 Hz), 3.83 (1H, d, *J* = 15.4 Hz), 3.27 (1H, d, *J* = 15.4 Hz), 3.06 (1H, d, *J* = 12.7 Hz), 2.99 (1H, dd, *J* = 16.2, 5.5 Hz), 2.95 – 2.85 (2H, m), 2.79 – 2.72 (1H, m), 2.58 (1H, ddtq, *J* = 11.6, 8.5, 5.9, 3.2 Hz), 2.51 – 2.44 (1H, m), 2.25 (1H, qd, *J* = 13.0, 3.4 Hz), 2.01 – 1.92 (1H, m), 1.82 (1H, dq, *J* = 13.4, 3.7 Hz), 1.48 – 1.34 (2H, m); **^{13}C NMR** (126 MHz, $CDCl_3$): δ_C = 199.9, 140.4, 140.2, 137.3, 134.6, 133.0, 128.9, 128.7, 128.3, 128.1, 128.0, 126.8, 126.2, 126.0, 125.7, 58.0, 56.8, 55.9, 45.9, 42.5, 35.3, 33.7, 32.2, 27.8. Spectroscopic data were consistent with the literature data for this compound.[5a]

_N_-Benzyl-3-methyl-1,2,3,4,4a,5,6,10b-octahydrophenanthridine (6):

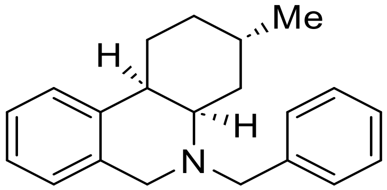

The title compound was prepared according to a literature procedure first reported by Kischkewitz and co-workers: 2-Benzyl-3-methylisoquinolin-2-ium iodide (**S5**) (180 mg, 0.50 mmol, 1.00 equiv.) was placed in a microwave vial with MVK (41.0 μL, 0.50 mmol, 1.0 equiv.), formic acid triethylamine complex 5:2 (168 μL, 2.00 mmol, 4.00 equiv.), and an aliquot of a premade solution of $(RhCp^*Cl_2)_2$ in MeCN (0.400 mL, 0.0100 mol%, 3.86 mg in 50.0 mL). The mixture was stirred at 80 °C for 18 h, then cooled to r.t. and diluted with CH_2Cl_2. The solution was washed with sat. aq. Na_2CO_3, the layers partitioned and the aqueous phase extracted twice more with CH_2Cl_2. The combined organic layers were dried over $MgSO_4$, filtered under gravity and concentrated *in vacuo*. Purification by flash column chromatography (2% EtOAc in pentane) afforded **6** as a colourless oil (61.3 mg, 42%, 96% pure by ^{1}H NMR).

^{1}H NMR (600 MHz, $CDCl_3$): δ_H = 7.48 (2H, dd, *J* = 7.1, 1.9 Hz), 7.38 (2H, td, *J* = 7.7, 2.5 Hz), 7.33 – 7.28 (1H, m), 7.19 – 7.12 (2H, m), 7.10 (1H, td, *J* = 7.4, 3.9 Hz), 6.91 (1H, d, *J* = 7.6 Hz), 4.38 (1H, dd, *J* = 13.0, 2.5 Hz), 3.85 (1H, dd, *J* = 15.5, 2.6 Hz), 3.28 (1H, dd, *J* = 15.4, 2.4 Hz), 3.11 (1H, dd, *J* = 13.1, 2.3 Hz), 2.88 (1H, q, *J* = 3.1 Hz), 2.72 (1H, dt, *J* = 11.8, 3.4 Hz), 2.31 (1H, dq, *J* = 14.7, 2.9 Hz), 2.20 (1H, dddd, *J* = 16.2, 13.0, 8.2, 3.4 Hz), 1.96 – 1.84 (2H, m), 1.79 (1H, dp, *J* = 13.5, 3.4 Hz), 1.34 (1H, ddt, *J* = 14.8, 11.9, 2.7 Hz), 1.28 – 1.18 (1H, m), 0.96 (3H, d, *J* = 6.6 Hz); **^{13}C NMR** (151 MHz, $CDCl_3$): δ_C = 140.8, 140.4, 134.6, 128.7, 128.4, 128.1, 126.9, 126.1, 126.0, 125.6, 58.3, 56.8, 56.1, 42.5, 37.5, 35.6, 32.4, 26.0, 22.7. Spectroscopic data were consistent with the literature data for this compound.[5a]

4-(2-Benzyl-3-phenyl-1,2,3,4-tetrahydroisoquinolin-4-yl)butan-2-one (9):

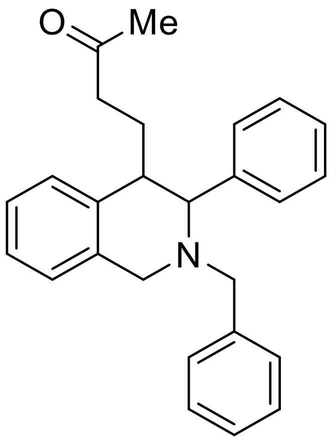

2-Benzyl-3-phenylisoquinolin-2-ium iodide (**S4**) (210 mg, 0.50 mmol, 1.0 equiv.) was dissolved in MeCN (0.40 mL) with MVK (42 µL, 0.50 mmol, 1.0 equiv.) and 5:2 HCO_2H:NEt_3 (0.17 mL, 2.0 mmol, 4.0 equiv.) then stirred at 80 °C for 18 h. The crude mixture was cooled to r.t., diluted with sat. aq. Na_2CO_3 then extracted three times with CH_2Cl_2. The combined organic extracts were dried over $MgSO_4$, filtered under gravity and concentrated *in vacuo*. Flask column chromatography (3-10% EtOAc in pentane) afforded **9** as a single diastereomer as a pale orange oil (67 mg, 36%). The relative stereochemistry was unassigned.

^{1}H NMR (500 MHz, $CDCl_3$): δ_H = 7.43 (2H, d, *J* = 7.6 Hz), 7.37 (2H, td, *J* = 7.6, 1.8 Hz), 7.34 – 7.15 (9H, m), 7.03 (1H, d, *J* = 7.3 Hz), 4.09 (1H, d, *J* = 4.6 Hz), 3.87 – 3.78 (2H, m), 3.68 (1H, d, *J* = 15.8 Hz), 3.48 (1H, d, *J* = 13.9 Hz), 3.24 (1H, q, *J* = 6.4 Hz), 2.44 (1H, ddd, *J* = 14.5, 9.7, 4.8 Hz), 2.33 – 2.19 (2H, m), 1.99 (3H, s), 1.75 – 1.63 (1H, m); **^{13}C NMR** (126 MHz, $CDCl_3$): δ_C = 208.8, 139.3, 138.1, 137.7, 135.3, 129.4, 128.6, 128.5, 128.2, 127.4, 127.0, 126.7, 126.4, 126.4, 126.1, 65.7, 59.9, 52.4, 42.7, 41.6, 29.9, 23.9; **IR** (neat) cm^{-1}: 1715, 1494, 1454, 1361, 911, 735, 702; **HRMS** (ESI$^+$) m/z: [M+H]$^+$ calcd for $C_{26}H_{28}NO$ 370.2165; found at 370.2181.

4-(1-Benzyl-1,2,3,4-tetrahydroquinolin-3-yl)butan-2-one (11):

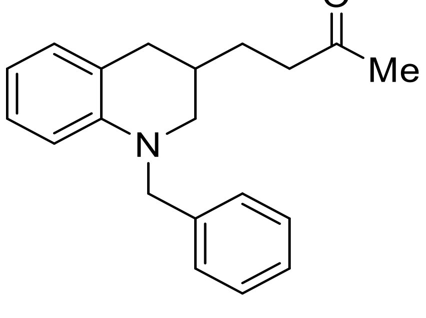

The title compound was prepared according to **General Procedure A** using 1-benzylquinolin-1-ium iodide (347 mg, 1.00 mmol, 1.00 equiv.) and MVK (82.0 µL, 1.00 mmol, 1.00 equiv.) as the electrophile. Purification with flash column chromatography (10-50% EtOAc in pentane) afforded **11** as a pale-yellow oil (12.0 mg, 4%).

^{1}H NMR (600 MHz, $CDCl_3$): δ_H = 7.34 – 7.29 (2H, m), 7.27 – 7.22 (3H, m), 7.01 – 6.95 (2H, m), 6.59 (1H, td, *J* = 7.3, 1.1 Hz), 6.55 – 6.50 (1H, m), 4.52 – 4.42 (2H, m), 3.32 (1H, ddd, *J* = 11.3, 3.9, 2.0 Hz), 3.07 (1H, dd, *J* = 11.3, 9.2 Hz), 2.92 – 2.84 (1H, m), 2.59 – 2.44 (3H, m), 2.14 (3H, s), 2.06 – 1.96 (1H, m), 1.73 – 1.57 (2H, m); **^{13}C NMR** (151 MHz, $CDCl_3$): δ_C = 208.7, 145.4, 139.0, 129.4, 128.7, 127.4, 127.0, 126.8, 121.3, 116.2, 111.0, 55.3, 55.0, 41.2, 34.4, 32.1, 30.1, 27.4; **IR** (neat) cm^{-1}: 2922, 1715, 1603, 1507, 1452, 1357, 1294, 1245, 1169, 745, 698; **HRMS** Compound could not be observed by ESI MS.

2.3 Rearomatization Products

4-(Isoquinolin-4-yl)butan-2-one (2):

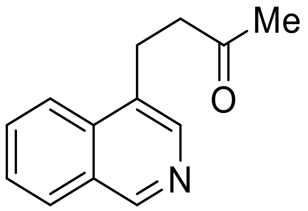

4-(2-Benzyl-1,2,3,4-tetrahydroisoquinolin-4-yl)butan-2-one **1** (37.0 mg, 0.125 mmol, 1.00 equiv.) was placed in a microwave vial with pyridine-*N*-oxide (18 mg, 0.19 mmol, 1.5 equiv.) and camphor (0.20 g), sealed with a crimped cap, heated to 250 °C for 40 min then cooled to r.t. Purification by flash column chromatography (60-80% EtOAc in pentane) afforded **2** as a pale-yellow oil (17 mg, 69%).

Mechanistic studies For experiments conducted with a different headspace, the microwave vial was charged with reactants, sealed with a crimped cap, then attached to a vacuum manifold *via* a needle. The reaction vial was evacuated and backfilled with the replacement gas three times. After the last backfill the needle was removed and the reacted then heated, cooled and purified as above.

4-(2-Benzyl-1,2,3,4-tetrahydroisoquinolin-4-yl)butan-2-one **1** (37.0 mg, 0.125 mmol, 1.00 equiv.) was placed in a microwave vial with 4-phenyl pyridine-*N*-oxide (32.0 mg, 0.188 mmol, 1.50 equiv.) and camphor (0.20 g), sealed with a crimped cap, heated to 250 °C for 40 min then cooled to r.t. Purification by flash column chromatography (60-80% EtOAc in

pentane) afforded **2** as a pale-yellow oil (21 mg, 85%) and 4-phenyl pyridine, **13**, as a pale brown solid (29 mg, quant.). The spectroscopic data of compound **13** was identical to that of commercial material.

4-(2-(4-Methoxybenzyl)-1,2,3,4-tetrahydroisoquinolin-4-yl)butan-2-one (**14**) (81 mg, 0.25 mmol, 1.0 equiv.) was added to a microwave vial with pyridine-*N*-oxide (36 mg, 0.38 mmol, 1.5 equiv.) and camphor (0.2 g), sealed with a crimped cap and heated to 250 °C for 40 mins. After cooling to r.t. purification by flash column chromatography (60-80% EtOAc in pentane) afforded **2** as a pale-yellow oil (22 mg, 44%).

4-(2-(Naphthalen-2-ylmethyl)-1,2,3,4-tetrahydroisoquinolin-4-yl)butan-2-one (**15**) (86 mg, 0.25 mmol, 1.0 equiv.) was added to a microwave vial with pyridine-*N*-oxide (36 mg, 0.38 mmol, 1.5 equiv.) and camphor (0.2 g), sealed with a crimped cap and heated to 250 °C for 40 mins. After cooling to r.t. purification by flash column chromatography (60-80% EtOAc in pentane) afforded **2** as a pale-yellow oil (33 mg, 67%).

2-Benzyl-4-(3-oxobutyl)-1,2,3,4-tetrahydroisoquinoline 2-oxide (**16**) (77 mg, 0.25 mmol, 1.0 equiv.) was added to a microwave vial with pyridine-*N*-oxide (36 mg, 0.38 mmol, 1.5 equiv.) and camphor (0.2 g), sealed with a crimped cap and heated to 250 °C for 40 mins. After cooling to r.t. purification by flash column chromatography (60-80% EtOAc in pentane) afforded **2** as a pale-yellow oil (16 mg, 33%).

Analytical data for **2**:

^{1}H NMR (600 MHz, $CDCl_3$): δ_H = 9.12 (1H, s), 8.37 (1H, s), 7.99 – 7.91 (2H, m), 7.72 (1H, dddt, *J* = 8.2, 6.9, 2.5, 1.3 Hz), 7.59 (1H, dddt, *J* = 8.1, 6.9, 2.4, 1.2 Hz), 3.28 (2H, td, *J* = 7.7, 3.3 Hz), 2.87 (2H, td, *J* = 7.8, 2.9 Hz), 2.16 (3H, s); **^{13}C NMR** (151 MHz, $CDCl_3$): δ_C = 207.2, 151.6, 142.5, 134.4, 130.5, 130.2, 128.5, 127.0, 122.6, 44.0, 30.1, 23.7. Spectroscopic data were consistent with the literature data for this compound.[6]

4-Hexylisoquinoline (4a):

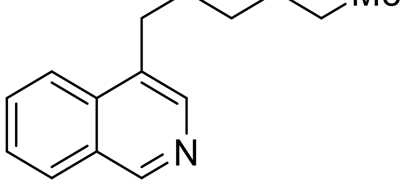

The title compound was synthesised using **General Procedure B** with 2-benzyl-4-hexyl-1,2,3,4-tetrahydroisoquinoline (**3a**) (77 mg, 0.25 mmol, 1.0 equiv.). Flash column chromatography (10% EtOAc in pentane) afforded **4a** as an orange oil (34 mg, 64%).

^{1}H NMR (400 MHz, $CDCl_3$): δ_H = 9.11 (1H, s), 8.37 (1H, s), 8.03 – 7.93 (2H, m), 7.72 (1H, ddd, J = 8.4, 6.8, 1.4 Hz), 7.59 (1H, ddd, J = 8.0, 6.8, 1.1 Hz), 3.05 – 2.96 (2H, m), 1.80 – 1.68 (2H, m), 1.49 – 1.39 (2H, m), 1.38 – 1.27 (4H, m), 0.95 – 0.82 (3H, m); **^{13}C NMR** (101 MHz, $CDCl_3$): δ_C = 151.2, 142.7, 134.8, 131.9, 130.2, 128.6, 128.4, 126.8, 123.1, 31.8, 30.8, 30.3, 29.5, 22.8, 14.2. Spectroscopic data were consistent with the literature data for this compound.[7]

4-(Furan-2-ylmethyl)isoquinoline (4b):

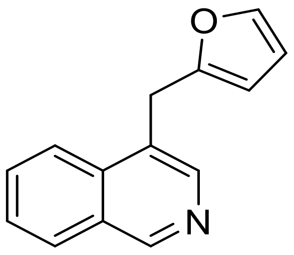

The title compound was synthesised using **General Procedure B** with 2-benzyl-4-(furan-2-ylmethyl)-1,2,3,4-tetrahydroisoquinoline (**3b**) (76 mg, 0.25 mmol, 1.0 equiv.). Flash column chromatography (15% EtOAc in pentane) afforded **4b** as an orange oil (21 mg, 41%).

^{1}H NMR (400 MHz, $CDCl_3$): δ_H = 9.19 (1H, s), 8.42 (1H, s), 7.96 – 7.87 (2H, m), 7.71 (1H, ddd, J = 8.3, 6.9, 1.4 Hz), 7.61 (1H, ddd, J = 8.1, 6.9, 1.1 Hz), 7.33 (1H, dd, J = 1.9, 0.8 Hz), 6.26 (1H, dd, J = 3.3, 1.9 Hz), 5.93 (1H, dq, J = 3.1, 1.0 Hz), 4.36 (2H, s); **^{13}C NMR** (101 MHz, $CDCl_3$): δ_C = 153.4, 152.3, 143.5, 141.6, 134.9, 130.6, 128.6, 128.4, 127.5, 127.2, 123.3, 110.6, 106.9, 29.3. Spectroscopic data were consistent with the literature data for this compound.[8]

4-(Isoquinolin-4-ylmethyl)benzonitrile (4c):

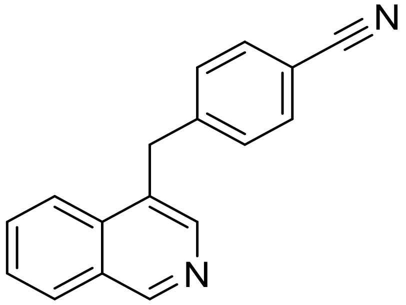

The title compound was synthesised using **General Procedure B** with 4-((2-benzyl-1,2,3,4-tetrahydroisoquinolin-4-yl)methyl)benzonitrile (**3c**) (84 mg, 0.25 mmol, 1.0 equiv.). Flash column chromatography (30-40% EtOAc in pentane) afforded **4c** as a pale orange powder (37 mg, 61%).

^{1}H NMR (400 MHz, $CDCl_3$): δ_H = 9.21 (1H, s), 8.41 (1H, s), 8.01 (1H, ddd, *J* = 8.0, 1.5, 0.8 Hz), 7.77 (1H, dq, *J* = 8.4, 0.9 Hz), 7.66 (1H, ddd, *J* = 8.4, 6.9, 1.5 Hz), 7.61 (1H, ddd, *J* = 8.1, 6.8, 1.3 Hz), 7.57 – 7.52 (2H, m), 7.32 – 7.27 (2H, m), 4.44 (2H, s); **^{13}C NMR** (101 MHz, $CDCl_3$): δ_C = 152.7, 145.5, 144.0, 134.7, 132.6, 130.9, 129.4, 128.8, 128.6, 128.2, 127.4, 123.1, 118.9, 110.5, 36.5. Spectroscopic data were consistent with the literature data for this compound.[9]

4-((1H-Indol-3-yl)methyl)isoquinoline (4d):

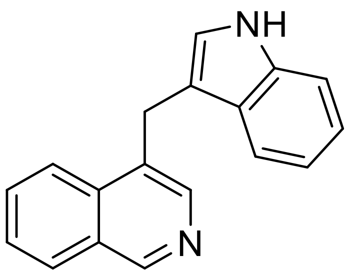

The title compound was synthesised using **General Procedure B** with 4-((1H-indol-3-yl)methyl)-2-benzyl-1,2,3,4-tetrahydroisoquinoline (**3d**) (88 mg, 0.25 mmol, 1.0 equiv.). Flash column chromatography (20% EtOAc in pentane) afforded **4d** as a brown solid (17 mg, 26%).

^{1}H NMR (600 MHz, $CDCl_3$): δ_H = 9.18 (1H, s), 8.47 (1H, s), 8.23 (1H, s), 8.04 (1H, d, *J* = 8.4 Hz), 7.99 (1H, d, *J* = 8.1 Hz), 7.65 (2H, td, *J* = 6.4, 1.5 Hz), 7.60 (1H, t, *J* = 7.5 Hz), 7.36 (1H, d, *J* = 8.1 Hz), 7.24 – 7.18 (1H, m), 7.14 (1H, t, *J* = 7.5 Hz), 6.69 – 6.66 (1H, m), 4.50 (2H, s); **^{13}C NMR** (151 MHz, $CDCl_3$): δ_C = 151.3, 142.6, 136.5, 135.3, 130.7, 130.5, 128.6, 128.4, 127.3, 127.2, 123.8, 122.9, 122.4, 119.7, 118.9, 114.5, 111.4, 26.4; **IR** (neat) cm^{-1}: 1623, 1457, 1228, 909, 739; **HRMS** (ESI^{+}) m/z: $[M+2H]^{2+}$ calcd for $C_{18}H_{16}N_2$ 260.1263; found at 260.1259; **m.p.** = 125-129 °C.

4-Benzylisoquinoline (4e):

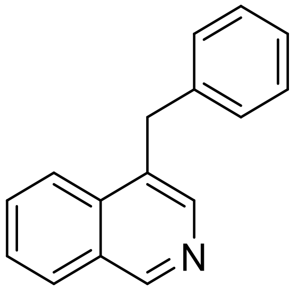

The title compound was synthesised using **General Procedure B** with 2,4-dibenzyl-1,2,3,4-tetrahydroisoquinoline (**3e**) (78 mg, 0.25 mmol, 1.0 equiv.). Flash column chromatography (10-15% EtOAc in pentane) afforded **4e** as a colourless solid (39 mg, 70%).

The title compound was synthesised using **General Procedure B** but on the 1 mmol scale, with 2,4-dibenzyl-1,2,3,4-tetrahydroisoquinoline (**3e**) (313 mg, 1.0 mmol, 1.0 equiv.). Flash column chromatography (10-15% EtOAc in pentane) afforded **4e** as a colourless solid (203 mg, 93%).

^{1}H NMR (400 MHz, $CDCl_3$): δ_H = 9.24 (1H, s), 8.48 (1H, s), 8.02 (1H, dt, *J* = 8.0, 1.1 Hz), 7.96 (1H, dt, *J* = 8.4, 1.0 Hz), 7.68 (1H, ddd, *J* = 8.4, 6.9, 1.4 Hz), 7.61 (1H, ddd, *J* = 8.0, 6.9, 1.2 Hz), 7.38 – 7.28 (2H, m), 7.28 – 7.20 (3H, m), 4.43 (2H, s); **^{13}C NMR** (101 MHz, $CDCl_3$): δ_C = 152.0, 143.9, 139.8, 134.9, 130.5, 129.7, 128.7 (2C), 128.6, 128.3, 127.0, 126.4, 123.6, 36.3. Spectroscopic data were consistent with the literature data for this compound.[10]

4-(Cyclopropylmethyl)isoquinoline (4f):

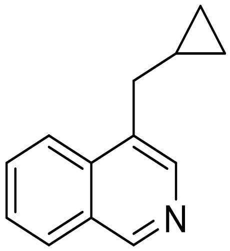

The title compound was synthesised using **General Procedure B** with 2-benzyl-4-(cyclopropylmethyl)-1,2,3,4-tetrahydroisoquinoline (**3f**) (69 mg, 0.25 mmol, 1.0 equiv.). Flash column chromatography (15% EtOAc in pentane) afforded **4f** as an orange oil (28 mg, 61%).

^{1}H NMR (400 MHz, $CDCl_3$): δ_H = 9.13 (1H, s), 8.47 (1H, s), 8.02 (1H, dq, *J* = 8.5, 1.0 Hz), 7.96 (1H, dt, *J* = 8.1, 1.1 Hz), 7.71 (1H, ddd, *J* = 8.4, 6.9, 1.4 Hz), 7.58 (1H, ddd, *J* = 8.0, 6.8, 1.1 Hz), 2.93 (2H, d, *J* = 6.7 Hz), 1.15 (1H, ttt, *J* = 8.0, 6.7, 4.9 Hz), 0.63 – 0.51 (2H, m), 0.27

(2H, dt, J = 6.0, 4.6 Hz); **^{13}C NMR** (101 MHz, $CDCl_3$): δ_C = 151.3, 142.4, 134.9, 131.0, 130.2, 128.4, 128.3, 126.8, 123.1, 34.5, 11.0, 5.3 (2C); **IR** (neat) cm^{-1}: 1623, 1583, 1504, 1390, 1046, 932, 900, 828, 798, 779, 750; **HRMS** (ESI^+) m/z: $[M+H]^+$ calcd for $C_{13}H_{14}N$ 184.1121; found at 184.1117.

3-(Isoquinolin-4-ylmethyl)quinoline (4g):

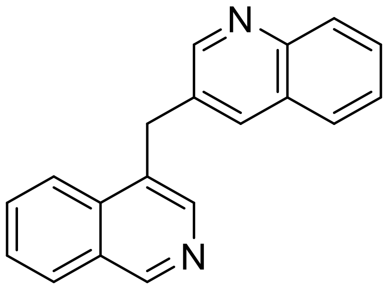

The title compound was synthesised using **General Procedure B** with 3-((2-Benzyl-1,2,3,4-tetrahydroisoquinolin-4-yl)methyl)quinoline (**3g**) (91 mg, 0.25 mmol, 1.0 equiv.). Flash column chromatography (80% - 100% EtOAc in pentane) afforded **4g** as a beige solid (39 mg, 66%).

^{1}H NMR (500 MHz, $CDCl_3$): δ_H = 9.25 (1H, s), 8.93 (1H, d, J = 2.2 Hz), 8.50 (1H, s), 8.08 (1H, d, J = 8.4 Hz), 8.03 (1H, dt, J = 8.1, 1.1 Hz), 7.88 (1H, d, J = 8.4 Hz), 7.81 – 7.77 (1H, m), 7.68 – 7.63 (3H, m), 7.60 (1H, ddd, J = 8.1, 7.0, 1.2 Hz), 7.48 (1H, ddd, J = 8.1, 6.9, 1.2 Hz), 4.57 (2H, s); **^{13}C NMR** (126 MHz, $CDCl_3$): δ_C = 152.4, 151.4, 147.0, 143.6, 135.0, 134.8, 132.6, 131.1, 129.3, 129.2, 128.8, 128.7 (2C), 128.1, 127.6, 127.5, 127.0, 123.3, 33.8; **IR** (neat) cm^{-1}: 1653, 1496, 1437, 1420, 1232, 911, 865, 791, 752, 733; **HRMS** (ESI^+) m/z: $[M+H]^+$ calcd for $C_{19}H_{15}N_2$ 271.1230; found at 271.1223; **m.p.** = 133-134 °C.

4-(Naphthalen-1-ylmethyl)isoquinoline (4h):

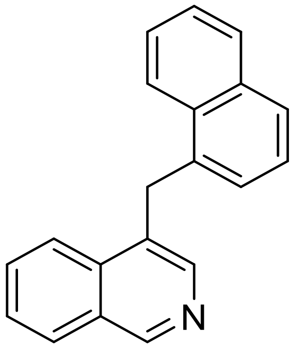

The title compound was synthesised using **General Procedure B** with 2-benzyl-4-(naphthalen-1-ylmethyl)-1,2,3,4-tetrahydroisoquinoline (**3h**) (91 mg, 0.25 mmol, 1.0 equiv.). Flash column chromatography (20% EtOAc in pentane) afforded **4h** as an orange solid (44 mg, 66%).

^{1}H NMR (600 MHz, $CDCl_3$): δ_H = 9.23 (1H, s), 8.28 (1H, s), 8.14 – 8.08 (1H, m), 8.05 – 8.00 (1H, m), 7.95 – 7.89 (2H, m), 7.77 (1H, d, *J* = 8.2 Hz), 7.64 (1H, ddd, *J* = 8.4, 6.9, 1.5 Hz), 7.61 (1H, ddd, *J* = 8.1, 6.8, 1.3 Hz), 7.57 – 7.51 (2H, m), 7.31 (1H, dd, *J* = 8.2, 7.1 Hz), 7.01 (1H, dt, *J* = 7.1, 1.1 Hz), 4.82 (2H, s); **^{13}C NMR** (151 MHz, $CDCl_3$): δ_C = 151.9, 144.0, 135.2, 135.1, 133.9, 132.0, 130.6, 129.3, 129.0, 128.5, 128.4, 127.4, 127.1, 126.8, 126.4, 125.9, 125.6, 123.6, 123.3, 33.0. Spectroscopic data were consistent with the literature data for this compound.[11]

Methyl 4-(isoquinolin-4-ylmethyl)benzoate (4i):

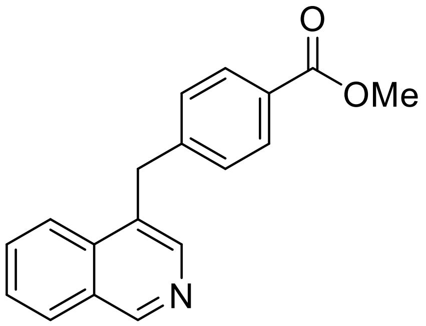

The title compound was synthesised using **General Procedure B** with methyl 4-((2-benzyl-1,2,3,4-tetrahydroisoquinolin-4-yl)methyl)benzoate (**3i**) (93 mg, 0.25 mmol, 1.0 equiv.). Flash column chromatography (30% EtOAc in pentane) afforded **4i** as a pale brown solid (40 mg, 57%).

^{1}H NMR (500 MHz, $CDCl_3$): δ_H = 9.22 (1H, s), 8.42 (1H, s), 8.02 (1H, dt, *J* = 8.0, 1.1 Hz), 7.96 – 7.91 (2H, m), 7.87 – 7.82 (1H, m), 7.66 (1H, ddd, *J* = 8.4, 6.9, 1.4 Hz), 7.61 (1H, ddd, *J* = 8.0, 6.8, 1.2 Hz), 7.26 (2H, d, *J* = 8.3 Hz), 4.44 (2H, s), 3.88 (3H, s); **^{13}C NMR** (126 MHz, $CDCl_3$): δ_C = 166.9, 151.9, 145.0, 143.2, 134.9, 130.9, 130.0, 129.2, 128.6, 128.5, 128.5, 127.3, 123.3, 52.1, 36.4. Spectroscopic data were consistent with the literature data for this compound.[12]

4-(3,5-Bis(trifluoromethyl)benzyl)isoquinoline (4j):

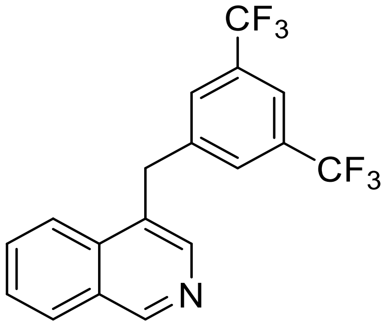

The title compound was synthesised using **General Procedure B** with 2-benzyl-4-((3,5-bis(trifluoromethyl)cyclohexyl)methyl)-1,2,3,4-tetrahydroisoquinoline (**3j**) (110 mg, 0.25 mmol, 1.0 equiv.). Flash column chromatography (20% EtOAc in pentane) afforded **4j** as an off white solid (54 mg, 61%).

^{1}H NMR (500 MHz, $CDCl_3$): δ_H = 9.24 (1H, s), 8.41 (1H, s), 8.02 (1H, dd, *J* = 8.1, 1.2 Hz), 7.80 (1H, d, *J* = 8.4 Hz), 7.73 (1H, s), 7.70 (1H, ddd, *J* = 8.4, 6.8, 1.4 Hz), 7.65 (2H, s), 7.62 (1H, ddd, *J* = 8.1, 6.9, 1.1 Hz), 4.50 (2H, s); **^{13}C NMR** (126 MHz, $CDCl_3$): δ_C = 152.9, 143.9, 142.3, 134.5, 132.1 (q, *J* = 33.2 Hz), 131.1, 128.8 – 128.6 (3C, m), 127.7, 127.5, 124.4 (q, *J* = 273.8 Hz), 122.8, 120.8 (hept, *J* = 3.8 Hz), 36.0; **^{19}F NMR** (377 MHz, $CDCl_3$): δ_F = -62.87; **IR** (neat) cm^{-1}: 1623, 1376, 1279, 1172, 1129, 906, 886, 843, 788, 750, 731, 706, 683; **HRMS** (ESI^{+}) m/z: $[M+H]^{+}$ calcd for $C_{18}H_{12}F_6N$ 356.0868; found at 356.0863; **m.p.** = 90-92 °C.

4-(Cyclohex-3-en-1-ylmethyl)isoquinoline (4k):

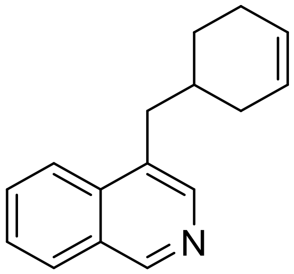

The title compound was synthesised using **General Procedure B** with 2-benzyl-4-(cyclohex-3-en-1-ylmethyl)-1,2,3,4-tetrahydroisoquinoline (**3k**) (79 mg, 0.25 mmol, 1.0 equiv.). Flash column chromatography (20% EtOAc in pentane) afforded **4k** as a pale yellow oil (39 mg, 70%).

^{1}H NMR (600 MHz, $CDCl_3$): δ_H = 9.12 (1H, s), 8.35 (1H, s), 7.98 (1H, d, *J* = 8.5 Hz), 7.96 (1H, d, *J* = 8.2 Hz), 7.70 (1H, ddd, *J* = 8.3, 6.8, 1.3 Hz), 7.58 (1H, t, *J* = 7.5 Hz), 5.68 – 5.59

(2H, m), 3.01 – 2.89 (2H, m), 2.12 – 1.91 (4H, m), 1.88 – 1.75 (2H, m), 1.38 (1H, dtd, *J* = 12.9, 10.5, 5.7 Hz); **^{13}C NMR** (151 MHz, $CDCl_3$): δ_C = 151.3, 143.5, 135.1, 130.2, 130.1, 128.6, 128.4, 127.2, 126.8, 126.1, 123.3, 36.9, 34.7, 32.0, 28.9, 25.1; **IR** (neat) cm^{-1}: 3022, 2916, 1622, 1582, 1502, 1452, 1335, 1390, 1225, 1042, 1020, 962, 896, 861, 797, 782, 751, 715, 700, 656, 619; **HRMS** (ESI^+) m/z: $[M+H]^+$ calcd for $C_{16}H_{18}N$ 224.1434; found at 224.1424.

5-(Isoquinolin-4-ylmethyl)-2-methoxyphenol (4l):

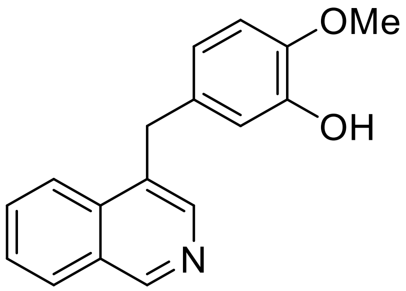

The title compound was synthesised using **General Procedure B** with 5-((2-benzyl-1,2,3,4-tetrahydroisoquinolin-4-yl)methyl)-2-methoxyphenol (**3l**) (78 mg, 0.25 mmol, 1.0 equiv.). Flash column chromatography (40% - 60% EtOAc in pentane) afforded **4l** as a beige solid (31 mg, 52%).

^{1}H NMR (500 MHz, $CDCl_3$): δ_H = 9.12 (1H, s), 8.37 (1H, s), 7.99 – 7.89 (2H, m), 7.66 (1H, ddd, *J* = 8.4, 6.9, 1.4 Hz), 7.58 (1H, ddd, *J* = 8.1, 6.8, 1.1 Hz), 6.76 (1H, d, *J* = 6.3 Hz), 6.75 (1H, s), 6.68 (1H, dd, *J* = 8.1, 2.1 Hz), 4.29 (2H, s), 3.84 (3H, s); **^{13}C NMR** (126 MHz, $CDCl_3$): δ_C = 151.4, 146.1, 145.5, 142.8, 135.2, 132.9, 130.9, 130.6, 128.7, 128.5, 127.3, 123.8, 119.9, 115.1, 111.0, 56.1, 35.8; **IR** (neat) cm^{-1}: 2931, 1625, 1531, 1434, 1391, 1283, 1268, 1247, 1224, 1133, 1109, 1033, 864, 787, 750, 714; **HRMS** (ESI^+) m/z: $[M+H]^+$ calcd for $C_{17}H_{16}NO_2$ 266.1176; found at 266.1172; **m.p.** = 108-109 °C.

4-(2-Bromo-4-methoxybenzyl)isoquinoline (4m):

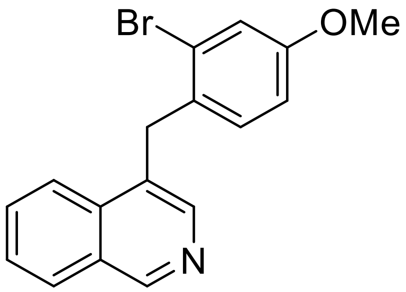

The title compound was synthesised using General Procedure B with 2-benzyl-4-(2-bromo-4-methoxybenzyl)-1,2,3,4-tetrahydroisoquinoline (**3m**) (106 mg, 0.250 mmol, 1.00 equiv.).

Flash column chromatography (30% EtOAc in pentane) afforded **4m** as a viscous brown oil (48.6 mg, 59%).

^{1}H NMR (600 MHz, $CDCl_3$): δ_H = 9.19 (1H, s), 8.28 (1H, s), 7.99 (1H, dd, *J* = 8.1, 1.3 Hz), 7.84 (1H, d, *J* = 8.5 Hz), 7.67 (1H, ddd, *J* = 8.3, 6.8, 1.3 Hz), 7.59 (1H, ddd, *J* = 8.0, 6.8, 1.1 Hz), 7.19 (1H, d, *J* = 2.6 Hz), 6.76 (1H, d, *J* = 8.6 Hz), 6.67 (1H, dd, *J* = 8.6, 2.6 Hz), 4.40 (2H, s), 3.75 (3H, s); **^{13}C NMR** (151 MHz, $CDCl_3$): δ_C = 158.8, 152.0, 143.7, 134.9, 130.8, 130.8, 130.7, 129.1, 128.6, 128.4, 127.2, 124.8, 123.4, 118.2, 113.7, 55.6, 35.4; **IR** (neat) cm^{-1}: 1605, 1568, 1493, 1461, 1439, 1390, 1317, 1285, 1235, 1184, 1148, 1037, 908, 861, 842, 791, 779, 747, 647, 629; **HRMS** (ESI^+) m/z: $[M+H]^+$ calcd for $C_{17}H_{15}{}^{79}BrNO$ 328.0332; found at 328.0338;

4-(Pyridin-2-ylmethyl)isoquinoline (4n):

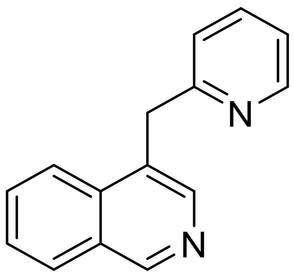

The title compound was synthesised using **General Procedure B** with 2-benzyl-4-(pyridin-2-ylmethyl)-1,2,3,4-tetrahydroisoquinoline (**3n**) (79 mg, 0.25 mmol, 1.0 equiv.). Flash column chromatography (20% EtOAc in pentane) afforded **4n** as a dark brown solid (23 mg, 42%).

^{1}H NMR (600 MHz, $CDCl_3$): δ_H = 9.18 (1H, s), 8.58 – 8.54 (1H, m), 8.48 (1H, s), 8.00 (1H, d, *J* = 8.5 Hz), 7.96 (1H, ddd, *J* = 8.2, 2.1, 1.1 Hz), 7.64 (1H, ddt, *J* = 8.3, 7.1, 1.3 Hz), 7.57 (1H, ddt, *J* = 8.2, 6.9, 1.3 Hz), 7.49 (1H, td, *J* = 7.7, 2.0 Hz), 7.12 – 7.07 (1H, m), 7.00 (1H, dd, *J* = 7.9, 1.4 Hz), 4.55 (2H, s); **^{13}C NMR** (151 MHz, $CDCl_3$): δ_C = 160.0, 152.2, 149.4, 143.7, 136.7, 135.1, 130.7, 128.9, 128.7, 128.3, 127.2, 123.9, 123.0, 121.6, 39.5; **IR** (neat) cm^{-1}: 1622, 1591, 1569, 1503, 1474, 1433, 1229, 1193, 1050, 994, 903, 797, 785, 776, 751; **HRMS** (ESI^+) m/z: $[M+H]^+$ calcd for $C_{15}H_{13}N_2$ 221.1073; found at 221.1070; **m.p.** = 70-74 °C.

1-Phenyl-2-(1,2,3,4-tetrahydrophenanthridin-3-yl)ethan-1-one (7):

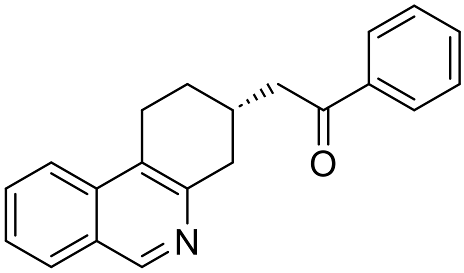

The title compound was synthesised using **General Procedure B** but on the 0.090 mmol scale with 2-(*N*-benzyl-1,2,3,4,4a,5,6,10b-octahydrophenanthridin-3-yl)-1-phenylethan-1-one (**5**) (36 mg, 0.09 mmol, 1.0 equiv.). Flash column chromatography (40% EtOAc in pentane) afforded **7** as a pale orange solid (21 mg, 75%).

^{1}H NMR (600 MHz, $CDCl_3$): δ_H = 9.06 (1H, s), 8.04 – 7.98 (1H, m), 7.92 (1H, dd, *J* = 8.1, 1.2 Hz), 7.90 (1H, dd, *J* = 8.5, 1.2 Hz), 7.70 (1H, ddd, *J* = 8.4, 6.9, 1.3 Hz), 7.59 – 7.52 (2H, m), 7.47 (1H, dd, *J* = 8.3, 7.1 Hz), 3.32 – 3.20 (2H, m), 3.17 (1H, dd, *J* = 16.2, 5.8 Hz), 3.12 – 3.03 (2H, m), 2.89 (1H, ddt, *J* = 17.0, 10.0, 1.7 Hz), 2.73 – 2.61 (1H, m), 2.23 (1H, dtt, *J* = 12.6, 5.8, 2.4 Hz), 1.65 (1H, dtd, *J* = 13.0, 10.7, 5.6 Hz); **^{13}C NMR** (151 MHz, $CDCl_3$): δ_C = 199.5, 150.3, 148.8, 137.4, 135.4, 133.2, 130.6, 128.8, 128.3, 128.3, 127.1, 126.2, 124.5, 122.2, 44.6, 39.2, 30.7, 28.6, 24.3; **IR** (neat) cm^{-1}: 2922, 1684, 1623, 1597, 1581, 1449, 1431, 1358, 1303, 1261, 1212, 1001, 775, 753, 738, 691; **HRMS** (ESI$^+$) m/z: $[M+H]^+$ calcd for $C_{21}H_{20}NO$ 302.1539; found at 302.1530; **m.p.** = 133-135 °C.

3-Methyl-1,2,3,4-tetrahydrophenanthridine (8):

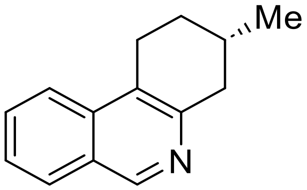

The title compound was synthesised using **General Procedure B** with *N*-benzyl-3-methyl-1,2,3,4,4a,5,6,10b-octahydrophenanthridine (**6**) (78 mg, 0.25 mmol, 1.0 equiv.). Flash column chromatography (20% EtOAc in pentane) afforded **8** as a brown oil (32 mg, 65%, 96% pure by ^{1}H NMR). Compound **8** contains a small impurity which could not be removed by chromatography; this is thought to originate from the starting material **6.**

^{1}H NMR (500 MHz, $CDCl_3$): δ_H = 9.04 (1H, s), 7.91 – 7.84 (2H, m), 7.66 (1H, ddt, *J* = 8.2, 6.9, 1.3 Hz), 7.50 (1H, ddd, *J* = 8.2, 6.9, 1.2 Hz), 3.25 – 3.10 (2H, m), 3.03 – 2.91 (1H, m),

2.70 (1H, ddt, J = 17.2, 10.0, 1.8 Hz), 2.11 – 1.94 (2H, m), 1.50 (1H, dddd, J = 12.7, 9.6, 6.1, 4.9 Hz), 1.14 (3H, d, J = 6.5 Hz); ^{13}**C NMR** (126 MHz, $CDCl_3$): δ_C = 150.2, 149.8, 135.3, 130.2, 128.2, 127.0, 125.9, 124.2, 122.1, 41.2, 30.8, 29.2, 24.6, 21.7; **IR** (neat) cm^{-1}: 2950, 2922, 1623, 1582, 1500, 14554, 1430, 1381, 1361, 1228, 1155, 774, 751; **HRMS** (ESI^+) m/z: $[M+H]^+$ calcd for $C_{14}H_{16}N$ 198.1277; found at 198.1273.

4-(3-Phenylisoquinolin-4-yl)butan-2-one (10):

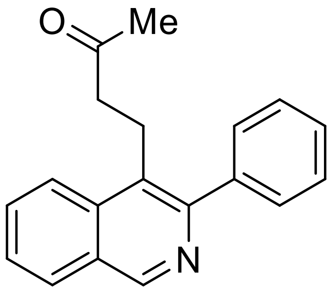

4-(2-Benzyl-3-phenyl-1,2,3,4-tetrahydroisoquinolin-4-yl)butan-2-one (**9**) (67 mg, 0.18 mmol, 1.0 equiv.) was placed in a microwave vial with pyridine-*N*-oxide (26 mg, 0.27 mmol, 1.5 equiv.) and camphor (0.20 g), then heated to 250 °C for 40 mins. Purification by flash column chromatography (20-40% EtOAc in pentane) afforded **20** as a pale brown oil (23 mg, 46%).

^{1}**H NMR** (600 MHz, $CDCl_3$): δ_H = 9.20 (1H, s), 8.04 – 8.01 (1H, m), 8.00 (1H, dd, J = 8.6, 1.0 Hz), 7.77 (1H, ddd, J = 8.4, 6.9, 1.3 Hz), 7.63 (1H, ddd, J = 8.0, 6.8, 1.0 Hz), 7.50 – 7.45 (4H, m), 7.44 – 7.39 (1H, m), 3.37 – 3.31 (2H, m), 2.73 – 2.67 (2H, m), 2.07 (3H, s); ^{13}**C NMR** (151 MHz, $CDCl_3$): δ_C = 207.4, 152.4, 150.6, 141.1, 135.2, 131.1, 129.2, 128.7, 128.5, 128.0, 127.9, 127.6, 127.0, 123.2, 44.5, 29.9, 22.5; **IR** (neat) cm^{-1}: 3434, 1776, 1713, 1620, 1575, 1498, 1446, 1422, 1364, 1235, 1164, 768, 703; **HRMS** (ESI^+) m/z: $[M+H]^+$ calcd for $C_{19}H_{18}NO$ 276.1383; found at 276.1388.

4-(Quinolin-3-yl)butan-2-one (12):

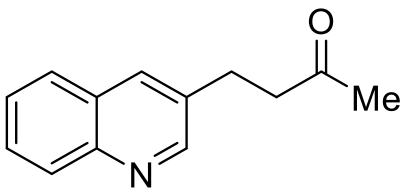

The title compound was synthesised using **General Procedure B** with 4-(1-benzyl-1,2,3,4-tetrahydroquinolin-3-yl)butan-2-one (**11**) (14 mg, 0.05 mmol, 1.0 equiv.). Flash column

chromatography (60% EtOAc in pentane) afforded **12** as a dark brown oil (2.0 mg, 20%, 90% purity by ^{1}H NMR).

^{1}H NMR (600 MHz, $CDCl_3$): δ_H = 8.82 (1H, d, *J* = 2.2 Hz), 8.22 (1H, d, *J* = 8.5 Hz), 8.11 (1H, d, *J* = 2.2 Hz), 7.82 (1H. dd, *J* = 8.2, 1.4 Hz), 7.73 (1H, ddd, *J* = 8.5, 6.9, 1.4 Hz), 7.59 (1H, ddd, *J* = 8.2, 6.8, 1.1 Hz), 3.12 (2H, t, *J* = 7.2 Hz), 2.90 (2H, t, *J* = 7.3 Hz), 2.17 (3H, s); **^{13}C NMR** (151 MHz, $CDCl_3$): δ_C = 206.9, 150.2, 145.0, 136.8, 134.2, 130.1, 128.3, 127.8, 127.6, 127.6, 44.4, 30.2, 26.9. Spectroscopic data were consistent with the literature data for this compound.[6]

2.4 Synthesis of Compounds for Mechanistic Studies

4-(2-(4-Methoxybenzyl)-1,2,3,4-tetrahydroisoquinolin-4-yl)butan-2-one (14):

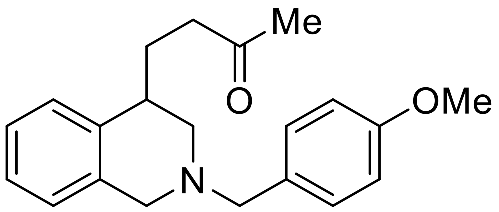

The title compound was prepared according to **General Procedure A** using 2-(4-methoxybenzyl)isoquinolin-2-ium bromide (**S1**) (1.13 g, 3.00 mmol, 1.00 equiv.) and MVK (252 µL, 3.00 mmol, 1.00 equiv.) as the electrophile. Purification with flash column chromatography (15-50% EtOAc in pentane) afforded **14** as a pale-yellow oil (275 mg, 28%).

^{1}H NMR (600 MHz, $CDCl_3$): δ_H = 7.32 – 7.28 (2H, m), 7.18 (1H, dd, *J* = 7.7, 1.8 Hz), 7.17 – 7.14 (1H, m), 7.11 (1H, td, *J* = 7.3, 1.8 Hz), 7.03 – 6.97 (1H, m), 6.91 – 6.84 (2H, m), 3.81 (3H, s), 3.80 – 3.75 (1H, m), 3.69 (1H, d, *J* = 12.8 Hz), 3.48 (2H, m), 2.81 (1H, dt, *J* = 8.6, 4.1 Hz), 2.71 – 2.65 (1H, m), 2.53 (1H, dd, *J* = 11.7, 4.3 Hz), 2.39 (1H, ddd, *J* = 16.2, 9.3, 6.5 Hz), 2.28 (1H, ddd, *J* = 16.8, 9.4, 5.6 Hz), 2.08 (3H, s), 2.05 – 1.94 (2H, m); **^{13}C NMR** (151 MHz, $CDCl_3$): δ_C = 209.1, 159.0, 138.4, 135.1, 130.6, 130.4, 128.5, 126.6, 126.4, 126.0, 113.8, 62.1, 56.7, 55.4, 53.8, 41.4, 37.8, 30.0, 29.9; **IR** (neat) cm^{-1}: 2932, 2796, 1714, 1612, 1513, 1464, 1454, 1367, 1302, 1248, 1172, 1094, 1036, 835, 739, 646, 633; **HRMS** (ESI^+) m/z: $[M+H]^+$ calcd for $C_{21}H_{26}NO_2$ 324.1958; found at 324.1973.

4-(2-(Naphthalen-2-ylmethyl)-1,2,3,4-tetrahydroisoquinolin-4-yl)butan-2-one (15):

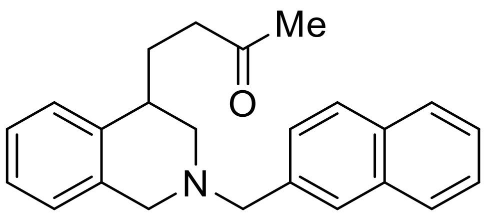

The title compound was prepared according to **General Procedure A** using 2-(naphthalen-2-ylmethyl)isoquinolin-2-ium bromide (**S2**) (352 mg, 1.00 mmol, 1.00 equiv.) and MVK (84.0 µL, 1.00 mmol, 1.00 equiv.) as the electrophile. Purification with flash column chromatography (5-10% acetone in pentane) afforded **15** as a pale-yellow oil (188 mg, 55%).

^{1}H NMR (600 MHz, $CDCl_3$): δ_H = 7.86 – 7.80 (4H, m), 7.58 (1H, dd, *J* = 8.4, 1.6 Hz), 7.54 – 7.43 (2H, m), 7.20 (1H, dd, *J* = 7.8, 1.6 Hz), 7.19 – 7.15 (1H, m), 7.13 (1H, td, *J* = 7.3, 1.8 Hz), 7.00 (1H, dd, *J* = 7.4, 1.4 Hz), 3.93 (1H, d, *J* = 13.0 Hz), 3.87 (1H, d, *J* = 14.8 Hz), 3.73 (1H, d, *J* = 13.0 Hz), 3.56 (1H, d, *J* = 14.9 Hz), 2.83 (1H, dq, *J* = 8.7, 4.2 Hz), 2.75 (1H, dd, *J* = 11.7, 3.9 Hz), 2.62 (1H, dd, *J* = 12.0, 4.1 Hz), 2.37 (1H, ddd, *J* = 16.2, 9.5, 6.3 Hz), 2.25 (1H, ddd, *J* = 16.9, 9.6, 5.4 Hz), 2.11 – 2.00 (2H, m), 2.00 (3H, s); **^{13}C NMR** (151 MHz, $CDCl_3$): δ_C = 209.0, 138.3, 136.1, 134.9, 133.5, 133.0, 128.5, 128.1, 127.8, 127.8, 127.8, 127.4, 126.6, 126.5, 126.2, 126.0, 125.8, 62.9, 56.8, 53.9, 41.4, 37.8, 30.0, 29.8; **IR** (neat) cm^{-1}: 3059, 1713, 1450, 1368, 1160, 1095, 911, 858, 821, 742; **HRMS** (ESI^+) m/z: $[M+H]^+$ calcd for $C_{24}H_{26}NO$ 344.2009; found at 344.2013.

2-Benzyl-4-(3-oxobutyl)-1,2,3,4-tetrahydroisoquinoline 2-oxide (16):

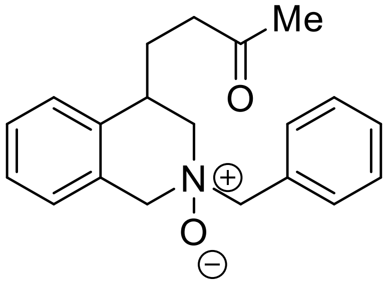

4-(2-Benzyl-1,2,3,4-tetrahydroisoquinolin-4-yl)butan-2-one (**1**) (100 mg, 0.34 mmol, 1.0 equiv.) was dissolved in CH_2Cl_2 (5.0 mL) and cooled to -78 °C with stirring. 3-Chloroperoxybenzoic acid (65 mg, 0.38 mmol, 1.1 equiv.) was added portionwise and the solution allowed to warm slowly to rt overnight. Sat. aq. $Na_2S_2O_3$ was added and the mixture was diluted with CH_2Cl_2. The layers were partitioned and the aqueous layer extracted twice more with CH_2Cl_2. The combined organic layers were washed with 2M aq. NaOH, dried over

$MgSO_4$, filtered under gravity and concentrated *in vacuo*. Purification by flash column chromatography (80-100% EtOAc in pentane) afforded **16** as an orange oil (94 mg, 89%).

^{1}H NMR (600 MHz, $CDCl_3$): δ_H = 7.47 (2H, s), 7.36 – 7.27 (3H, m), 7.22 (1H, d, *J* = 7.8 Hz), 7.11 (1H, t, *J* = 7.6 Hz), 7.02 (1H, t, *J* = 7.5 Hz), 6.79 (1H, d, *J* = 7.7 Hz), 4.48 – 4.38 (3H, m), 3.93 (1H, d, *J* = 14.5 Hz), 3.68 (1H, qd, *J* = 9.8, 6.3 Hz), 3.34 (1H, ddd, *J* = 11.5, 5.9, 2.5 Hz), 3.02 (1H, t, *J* = 11.4 Hz), 2.33 (2H, t, *J* = 7.5 Hz), 2.13 (1H, dtd, *J* = 15.2, 7.6, 3.4 Hz), 1.99 (3H, s), 1.85 (1H, dq, *J* = 15.4, 7.7 Hz); **^{13}C NMR** (151 MHz, $CDCl_3$): δ_C = 207.5, 134.6, 132.4, 129.6, 129.5, 128.9, 128.5, 127.5, 127.1, 126.4, 125.8, 76.4, 65.6, 65.5, 40.0, 32.8, 29.9, 24.2; **IR** (neat) cm^{-1}:1713, 1497, 1456, 911, 731, 702; **HRMS** (ESI^+) m/z: $[M+H]^+$ calcd for $C_{20}H_{24}NO_2$ 310.1802; found at 310.1801.

2-Benzyl-1,2,3,4-tetrahydroisoquinoline (17):

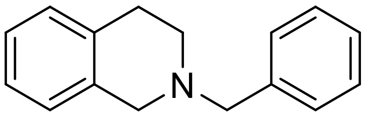

1,2,3,4-Tetrahydroisoquinoline (1.25 mL, 10.0 mmol, 1.00 equiv.) was dissolved in CH_2Cl_2 (20 mL) with NEt_3 (4.2 mL, 30 mmol, 3.0 equiv.). BnBr (1.78 mL, 15.0 mmol, 1.50 equiv.) was added dropwise, then the solution was stirred at r.t. overnight. The reaction was quenched with H_2O, diluted with CH_2Cl_2, shaken and the layers partitioned. The aqueous phase was extracted twice more with CH_2Cl_2, then the combined organic layers were washed once with brine, dried over $MgSO_4$, filtered under gravity and concentrated *in vacuo*. The residue was passed through a short silica plug to afford **17** as a pale-yellow oil (1.63 g, 73%).

^{1}H NMR (600 MHz, $CDCl_3$): δ_H = 7.43 – 7.39 (2H, m), 7.38 – 7.32 (2H, m), 7.30 – 7.26 (1H, m), 7.16 – 7.07 (3H, m), 7.01 – 6.97 (1H, m), 3.70 (2H, s), 3.65 (2H, s), 2.91 (2H, t, *J* = 6.0 Hz), 2.76 (2H, t, *J* = 5.9 Hz); **^{13}C NMR** (151 MHz, $CDCl_3$): δ_C = 138.6, 135.1, 134.6, 129.2, 128.8, 128.4, 127.2, 126.7, 126.2, 125.7, 63.0, 56.3, 50.8, 29.3. Spectroscopic data were consistent with the literature data for this compound.[14]

2-Benzylisoquinolin-2-ium iodide (19):

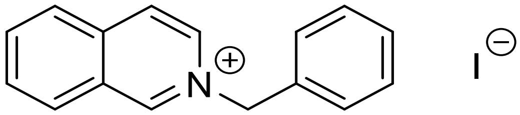

Isoquinoline (0.74 g, 5.8 mmol, 1.0 equiv.) and benzyl iodide (3.8 g, 17 mmol, 3.0 equiv.) were placed in a large microwave vial with a stirrer bar, sealed and heated at 80 °C with stirring for 48 h. The reaction mixture was cooled and triturated with diethyl ether. The resultant precipitate was collected by vacuum filtration to give **19** as a bright yellow solid (1.6 g, 82%).

^{1}H NMR (600 MHz, $CDCl_3$): δ_H = 10.95 (1H, s), 8.82 (1H, dd, *J* = 6.8, 1.5 Hz), 8.60 (1H, d, *J* = 8.4 Hz), 8.29 (1H, d, *J* = 6.8 Hz), 8.08 (1H, d, *J* = 8.3 Hz), 8.03 (1H, ddd, *J* = 8.2, 6.9, 1.2 Hz), 7.85 (1H, ddd, *J* = 8.2, 7.0, 1.2 Hz), 7.74 – 7.68 (2H, m), 7.33 – 7.26 (3H, m), 6.27 (2H, s); **^{13}C NMR** (151 MHz, $CDCl_3$): δ_c = 149.4, 137.5, 137.3, 134.4, 132.9, 131.4, 131.0, 130.0, 129.7, 129.6, 127.6, 127.2, 126.4, 63.6. Spectroscopic data were consistent with the literature data for this compound.[5a]

2-Benzyl-3,4-dihydroisoquinolin-2-ium iodide (21):

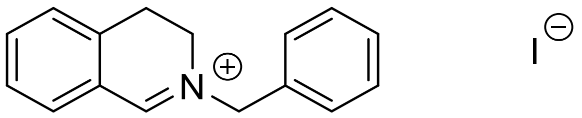

3,4-Dihydroisoquinoline (262 mg, 2.00 mmol, 1.00 equiv.) was dissolved in acetone (5.0 mL) with BnI (523 mg, 2.40 mmol, 1.20 equiv.) and stirred at r.t. overnight, then triturated with Et_2O. The precipitate was filtered under vacuum to afford **21** as a pale-yellow solid (657 mg, 94%).

^{1}H NMR (600 MHz, $CDCl_3$): δ_H = 10.33 (1H, s), 8.09 (1H, d, *J* = 7.7 Hz), 7.65 (1H, t, *J* = 7.6 Hz), 7.62 (2H, dd, *J* = 6.6, 2.9 Hz), 7.43 – 7.36 (4H, m), 7.30 (1H, d, *J* = 7.6 Hz), 5.54 (2H, s), 4.00 (2H, t, *J* = 8.1 Hz), 3.25 (2H, t, *J* = 8.1 Hz); **^{13}C NMR** (151 MHz, $CDCl_3$): δ_C = 166.1, 138.3, 136.1, 135.0, 130.6, 130.1, 130.0, 129.7, 128.8, 128.3, 124.6, 64.1, 48.5, 25.6; **IR** (neat) cm^{-1}: 3006 (br), 1654, 1603, 1572, 1491, 1445, 1415, 1361, 1312, 1286, 1227, 1152, 1134, 1087, 1051, 1003, 783, 773, 717, 702; **HRMS** (ESI^+) m/z: $[M]^+$ calcd for $C_{16}H_{16}N$ 222.1277; found at 222.1282; **LRMS** (ESI^+) m/z: $[I]^-$ calcd for I 126.9; found at 126.9.

3,4-Dihydroisoquinoline 2-oxide (22):

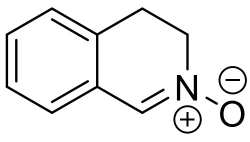

1,2,3,4-Tetrahydroisoquinoline (1.3 g, 10 mmol, 1.0 equiv.) was dissolved in MeOH (30 mL) with H_2O_2 (4.1 mL, 40 mmol, 4.0 equiv. 34.5-36.5% w/w) and heated to 50 °C overnight. The

solution was then cooled to r.t. and diluted with H_2O. The mixture was extracted three times with CH_2Cl_2, the organic layers combined, dried over $MgSO_4$, filtered under gravity and concentrated *in vacuo*. Purification by flash column chromatography on deactivated silica (5-10% EtOH in EtOAc) afforded **22** as a pale orange oil (260 mg, 18%).

^{1}H NMR (600 MHz, $CDCl_3$): δ_H = 7.74 (1H, s), 7.29 – 7.23 (2H, m), 7.23 – 7.17 (1H, m), 7.13 – 7.07 (1H, m), 4.09 (2H, td, *J* = 7.9, 2.5 Hz), 3.17 (2H, td, *J* = 7.8, 2.7 Hz); **^{13}C NMR** (151 MHz, $CDCl_3$): δ_C = 134.2, 130.2, 129.5, 128.5, 127.8, 127.4, 125.6, 58.1, 27.9. Spectroscopic data were consistent with the literature data for this compound.[15]

Mechanism studies in Scheme 5

Isoquinoline (23):

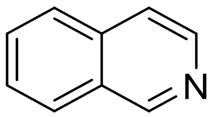

The title compound was synthesised using **General Procedure B** with *N*-benzyl tetrahydroisoquinoline (**17**) (56 mg, 0.25 mmol, 1.0 equiv.). Flash column chromatography (20% EtOAc in pentane) afforded **23** as a brown solid (25 mg, 76%).

N-benzyl tetrahydroisoquinoline (**17**) (56 mg, 0.25 mmol, 1.0 equiv.) was heated to 250 °C in a microwave vial for 40 mins with camphor (0.20 g), sealed with a crimped cap. Flash column chromatography (20% EtOAc in pentane) afforded **23** as a brown solid (10 mg, 31%).

The title compound was synthesised using **General Procedure B** with 1,2,3,4-tetrahydroisoquinoline (**18**) (33 mg, 0.25 mmol, 1.0 equiv.). Flash column chromatography (20% EtOAc in pentane) afforded **23** as a brown solid (16 mg, 51%).

1,2,3,4-Tetrahydroisoquinoline (**18**) (33 mg, 0.25 mmol, 1.0 equiv.) was heated to 250 °C in a microwave vial for 40 mins with camphor (0.20 g), sealed with a crimped cap. The residue was dissolved in $CDCl_3$ (0.5 mL) and trimethoxy benzene was added as an internal standard (14 mg, 0.083 mmol, 0.33 equiv.). Analysis by quantitative ^{1}H NMR showed a yield of 9%.

The title compound was synthesised using **General Procedure B** with 2-benzylisoquinolin-2-ium iodide (**19**) (87 mg, 0.25 mmol, 1.0 equiv.). Flash column chromatography (20% EtOAc in pentane) afforded **23** as a brown solid (16 mg, 51%).

2-Benzylisoquinolin-2-ium iodide (**19**) (87 mg, 0.25 mmol, 1.0 equiv.) was heated to 250 °C in a microwave vial for 40 mins with camphor (0.20 g), sealed with a crimped cap. Flash column chromatography (20% EtOAc in pentane) failed to afford any product.

The title compound was synthesised using **General Procedure B** with 3,4-dihydroisoquinoline (**20**) (33 mg, 0.25 mmol, 1.0 equiv.). Flash column chromatography (20% EtOAc in pentane) afforded **23** as a brown solid (20 mg, 63%).

3,4-Dihydroisoquinoline (**20**) (33 mg, 0.25 mmol, 1.0 equiv.) was heated to 250 °C in a microwave vial for 40 mins with camphor (0.20 g), sealed with a crimped cap. The residue was dissolved in $CDCl_3$ (0.5 mL) and trimethoxy benzene was added as an internal standard (14 mg, 0.083 mmol, 0.33 equiv.). Analysis by quantitative ^{1}H NMR showed a yield of 51%.

2-Benzyl-3,4-dihydroisoquinolin-2-ium iodide (**21**) (87 mg, 0.25 mmol, 1.0 equiv.) was placed in a microwave vial with pyridine-*N*-oxide (36 mg, 0.38 mmol, 1.5 equiv.) and camphor (0.2 g), then heated to 250 °C in a microwave vial sealed with a crimped cap for 40 mins. Purification by flash column chromatography (20% EtOAc in pentane) failed to afford any product.

2-Benzyl-3,4-dihydroisoquinolin-2-ium iodide (**21**) (87 mg, 0.25 mmol, 1.0 equiv.) was placed in a microwave vial with camphor (0.2 g), then heated to 250 °C in a microwave vial sealed with a crimped cap for 40 mins. Purification by flash column chromatography (20% EtOAc in pentane) failed to afford any product.

The title compound was synthesised using **General Procedure B** with 3,4-dihydroisoquinoline 2-oxide (**22**) (37 mg, 0.25 mmol, 1.0 equiv.). Flash column chromatography (20% EtOAc in pentane) afforded **23** as a brown solid (9.0 mg, 28%).

3,4-Dihydroisoquinoline 2-oxide (**22**) (37 mg, 0.25 mmol, 1.0 equiv.) was heated to 250 °C in a microwave vial for 40 mins with camphor (0.20 g), sealed with a crimped cap. Flash column chromatography (20% EtOAc in pentane) afforded **23** as a brown solid (12 mg, 37%).

3,4-Dihydroisoquinoline 2-oxide (**22**) (37 mg, 0.25 mmol, 1.0 equiv.) was heated to 250 °C in a microwave vial for 40 mins with camphor (0.20 g), and pyridine (30.0 µL, 0.375 mmol, 1.50 equiv.) sealed with a crimped cap. Flash column chromatography (20% EtOAc in pentane) afforded **23** as a brown solid (17 mg, 53%).

^{1}H NMR (400 MHz, $CDCl_3$): δ_H = 9.24 (1H, s), 8.51 (1H, d, *J* = 5.8 Hz), 7.94 (1H, dd, *J* = 8.1, 1.1 Hz), 7.79 (1H, dd, *J* = 8.2, 1.2 Hz), 7.71 – 7.51 (3H, m); **^{13}C NMR** (101 MHz, $CDCl_3$): δ_C

= 152.6, 143.0, 135.8, 130.4, 128.7, 127.7, 127.3, 126.5, 120.5. Spectroscopic data were consistent with the literature data for this compound.[13]

2.5 Synthesis of Starting Material Salts and Electrophiles

2-(4-Methoxybenzyl)isoquinolin-2-ium iodide (S1):

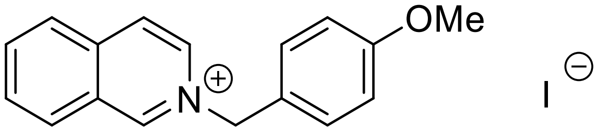

Isoquinoline (1.29 g, 10.0 mmol, 1.00 equiv.) was dissolved in acetone (40.0 mL) with 4-methoxybenzyl chloride (1.88 g, 12.0 mmol, 1.20 equiv.) and KI (3.32 g, 20.0 mmol, 2.00 equiv.) then stirred at r.t. for 4h. The resultant precipitate was filtered off , washed with H_2O and acetone, then dried *in vacuo* to give **S1** as a pale yellow solid (3.27 g, 87%).

^{1}H NMR (600 MHz, DMSO): δ_H = 10.33 (1H, s), 8.84 (1H, dt, *J* = 6.9, 1.3 Hz), 8.60 (1H, d, *J* = 6.8 Hz), 8.53 (1H, dd, *J* = 8.4, 1.2 Hz), 8.35 (1H, dd, *J* = 8.4, 1.1 Hz), 8.26 (1H, ddd, *J* = 8.3, 7.0, 1.2 Hz), 8.08 (1H, ddd, *J* = 8.2, 6.9, 1.1 Hz), 7.64 – 7.58 (2H, m), 7.03 – 6.97 (2H, m), 5.92 (2H, s), 3.75 (3H, s); **^{13}C NMR** (151 MHz, DMSO): δ_C = 160.0, 149.7, 137.0, 137.0, 134.5, 131.3, 130.7, 130.5, 127.3, 127.2, 126.2, 126.0, 114.5, 62.9, 55.3. Spectroscopic data were consistent with the literature data for this compound.[17]

2-(Naphthalen-2-ylmethyl)isoquinolin-2-ium bromide (S2):

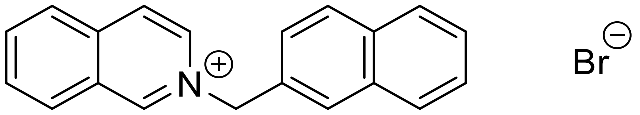

Isoquinoline (0.65 g, 5.0 mmol, 1.0 equiv.) was dissolved in acetone (25 mL) along with 2-bromomethylnaphthalene (1.4 g, 6.0 mmol, 1.2 equiv.) and stirred at r.t. for 3h. The resultant precipitate was filtered *in vacuo* to afford **S2** as a pale yellow solid (0.67 g, 38%).

^{1}H NMR (600 MHz, $CDCl_3$): δ_H = 11.19 (1H, d, *J* = 1.4 Hz), 8.94 (1H, dd, *J* = 6.8, 1.4 Hz), 8.54 (1H, dt, *J* = 8.5, 1.0 Hz), 8.27 (1H, d, *J* = 1.8 Hz), 8.13 (1H, d, *J* = 6.8 Hz), 7.93 – 7.87 (2H, m), 7.78 – 7.69 (3H, m), 7.68 – 7.62 (2H, m), 7.41 – 7.33 (2H, m), 6.54 (2H, s); **^{13}C NMR** (151 MHz, $CDCl_3$): δ_C = 150.2, 137.2, 137.0, 134.5, 133.4, 133.1, 131.2, 131.1, 130.7, 129.7, 129.6, 128.4, 127.7, 127.7, 127.2, 127.0, 126.8, 126.2, 126.1, 63.8. Spectroscopic data were consistent with the literature data for this compound.[18]

(E)-1-Phenylpenta-2,4-dien-1-one (S3):

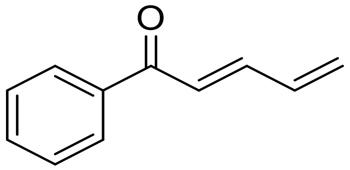

The title compound was synthesised according to a procedure first published by Jørgensen and co-workers: Fresh LDA was produced by adding nBuLi (11.2 mL, 2.50 M in hexanes, 28.0 mmol, 1.10 equiv.) dropwise so a stirred solution of diisopropylamine (4.05 mL, 28.0 mmol, 1.10 equiv.) in anhydrous THF (30.0 mL) at -78 °C. Acetophenone (2.92 mL, 25.0 mmol, 1.00 equiv.) dissolved in anhydrous THF (5.00 mL) was added dropwise, and the solution stirred for 1 h. Acrolein (1.87 mL, 28.0 mmol, 1.10 equiv.) dissolved in anhydrous THF (5.00 mL) was added dropwise and the solution stirred for 5 mins, after which the reaction was quenched with sat. aq. NH_4Cl, poured onto Et_2O and the layers partitioned. The aqueous layer was extracted twice more with Et_2O, the combined organic layers washed with brine, dried over $MgSO_4$, filtered under gravity and concentrated *in vacuo*. The crude mixture was dissolved in CH_2Cl_2 (25 mL) and TsOH (2.40 g, 14.2 mmol, 0.570 equiv.) was added, after which the mixture was refluxed for 3 h. The reaction was cooled to r.t., diluted with CH_2Cl_2, washed with water and the layers partitioned. The aqueous layer was extracted twice more with CH_2Cl_2 then the combined organic layers were dried over $MgSO_4$, filtered under gravity and concentrated *in vacuo*. Flash column chromatography (1-5% EtOAc in pentane) afforded **S3** as a pale-yellow oil (1.05 g, 27%).

^{1}H NMR (400 MHz, $CDCl_3$): δ_H = 7.97 – 7.93 (2H, m), 7.61 – 7.52 (1H, m), 7.51 – 7.45 (2H, m), 7.40 (1H, ddt, *J* = 15.2, 11.0, 0.8 Hz), 6.99 (1H, dd, *J* = 15.1, 0.8 Hz), 6.60 (1H, dddd, *J* = 16.9, 10.8, 10.0, 0.7 Hz), 5.72 (1H, ddt, *J* = 16.9, 1.5, 0.8 Hz), 5.59 (1H, ddt, *J* = 10.0, 1.4, 0.7 Hz); **^{13}C NMR** (101 MHz, $CDCl_3$): δ_C = 190.9, 144.9, 138.1, 135.5, 132.9, 128.7, 128.6, 127.0, 126.4. Spectroscopic data were consistent with the literature data for this compound.[19]

2-Benzyl-3-phenylisoquinolin-2-ium iodide (S4):

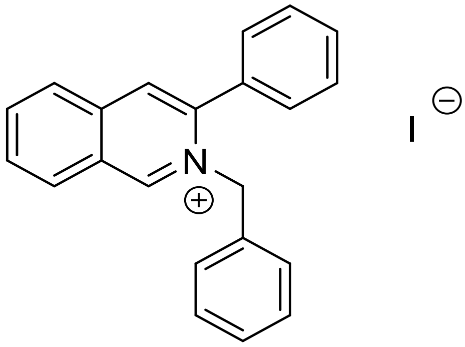

3-Phenyl isoquinoline (226 mg, 1.10 mmol, 1.00 equiv.) was dissolved in acetone (20.0 mL) with benzyl iodide (288 mg, 1.30 mmol, 1.20 equiv.) and stirred at 60 °C overnight. Precipitate was filtered *in vacuo*, washed with cold Et_2O and dried to afford **S4** as a pale-yellow powder (250 mg, 54%).

^{1}H NMR (600 MHz, $CDCl_3$): δ_H = 11.17 (1H, s), 8.81 (1H, dt, *J* = 8.3, 1.0 Hz), 8.14 (1H, ddd, *J* = 8.2, 7.0, 1.2 Hz), 8.09 – 8.04 (2H, m), 7.96 (1H, ddd, *J* = 8.2, 7.0, 1.1 Hz), 7.64 – 7.58 (1H, m), 7.57 – 7.51 (2H, m), 7.46 – 7.41 (2H, m), 7.29 – 7.25 (1H, m), 7.24 – 7.20 (2H, m), 7.04 – 6.99 (2H, m), 6.15 (2H, s); **^{13}C NMR** (151 MHz, $CDCl_3$): δ_C = 152.2, 146.1, 138.0, 137.8, 133.2, 131.8, 131.7, 131.4, 131.1, 130.0, 129.5 (2C), 129.3, 128.6, 127.4, 127.3, 126.8, 61.9; **IR** (neat) cm^{-1}: 1641, 1495, 918, 764, 734, 703; **HRMS** (ESI$^+$) m/z: $[M]^+$ calcd for $C_{22}H_{18}N$ 296.1434; found at 296.1444; **m.p.** = 150 °C.

2-Benzyl-3-methylisoquinolin-2-ium iodide (S5):

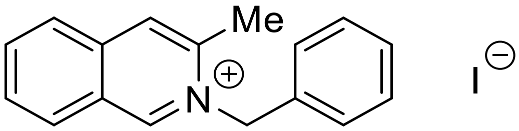

3-Methyl isoquinoline (0.50 g, 3.5 mmol, 1.0 equiv.) and benzyl iodide (0.92 g, 4.2 mmol, 1.2 equiv.) were dissolved in acetone (10 mL) in a 25 mL microwave vial with a stirrer bar, sealed and stirred at 60 °C overnight. The reaction mixture was cooled and triturated with diethyl ether. The resultant precipitate was collected by vacuum filtration to give **S5** as a pale-yellow solid (1.1 g, 85%).

^{1}H NMR (400 MHz, $CDCl_3$): δ_H = 10.94 (1H, s), 8.61 (1H, dd, *J* = 8.5, 1.2 Hz), 8.24 (1H, s), 8.09 – 7.98 (2H, m), 7.81 (1H, ddd, *J* = 8.2, 6.4, 1.6 Hz), 7.34 – 7.23 (5H, m), 6.27 (2H, s), 2.87 (3H, s); **^{13}C NMR** (101 MHz, $CDCl_3$): δ_C = 151.1, 144.2, 138.6, 137.4, 132.2, 130.7,

130.6, 129.6, 129.4, 128.0, 127.1, 126.7, 126.3, 61.5, 20.7; Spectroscopic data were consistent with the literature data for this compound.[5a]

4-(2-Methyl-1,2,3,4-tetrahydroisoquinolin-4-yl)butan-2-one (S6):

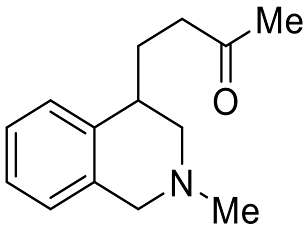

2-Methylisoquinolin-2-ium iodide (1.4 g, 5.0 mmol, 1.0 equiv.) was dissolved in MeCN (4.0 mL) along with MVK (0.41 mL, 5.0 mmol, 1.0 equiv.) and 5:2 HCO_2H:NEt_3 (1.2 mL, 20 mmol, 4.0 equiv.). The solution was heated to 80 °C for 18 h, then cooled, diluted with CH_2Cl_2 and quenched with sat. aq. Na_2CO_3. The layers were partitioned and the aqueous layer was extracted twice more with CH_2Cl_2. The combined organic layers were dried over $MgSO_4$, filtered under gravity and concentrated *in vacuo*. Column chromatography (100% EtOAc) afforded **S6** as a pale orange oil (0.43 g, 40%).

^{1}H NMR (600 MHz, $CDCl_3$): δ_H = 7.20 (1H, dd, *J* = 7.7, 1.6 Hz), 7.16 (1H, td, *J* = 7.4, 1.5 Hz), 7.12 (1H, td, *J* = 7.3, 1.6 Hz), 7.04 – 6.99 (1H, m), 3.67 (1H, d, *J* = 14.8 Hz), 3.39 (1H, d, *J* = 14.8 Hz), 2.90 (1H, dq, *J* = 9.0, 4.6 Hz), 2.57 (2H, t, *J* = 4.1 Hz), 2.55 – 2.49 (1H, m), 2.46 – 2.39 (1H, m), 2.38 (3H, s), 2.12 (4H, s), 1.96 (1H, dtd, *J* = 14.2, 8.4, 5.8 Hz); **^{13}C NMR** (151 MHz, $CDCl_3$): δ_C = 208.8, 137.5, 135.2, 128.3, 126.4 (2C), 126.0, 58.5, 57.4, 46.3, 41.2, 37.7, 30.1, 29.7; **IR** (neat) cm^{-1}: 2781, 1715, 1493, 1451, 1380, 1357, 1253, 1161, 1146, 1129, 1104, 1038, 1003, 769, 742; **HRMS** (ESI$^+$) m/z: [M]$^+$ calcd for $C_{14}H_{20}NO$ 218.1539; found at 218.1536.

3. Extended optimisation table

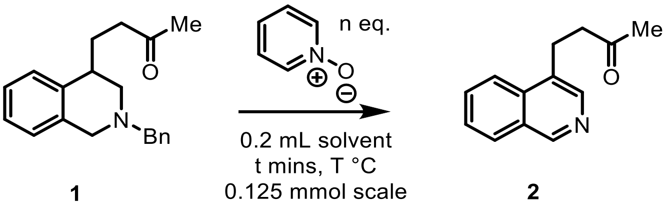

#	°C	Reagent	Reagent eq.	Time (mins)	Solvent	Additive	Balloon	RP HPLC Yield (%)	Isolated Yield (%)
**1**	200	Pyridine-*N*-oxide	5.0	40	-	-	Sealed	6[a]	-
**2**	200	Pyridine-*N*-oxide	5.0	40	Toluene	-	Sealed	-	32
**3**	200	Pyridine-*N*-oxide	5.0	40	Camphor	-	Sealed	-	52
**4**	200	Pyridine-*N*-oxide	5.0	40	Nitrobenzene	-	Sealed	-	40
**5**	200	Pyridine-*N*-oxide	5.0	40	MeCN	-	Sealed	-	41
**6**	200	Pyridine-*N*-oxide	5.0	40	Sulpholane	-	Sealed	28	26
**7**	200	Pyridine-*N*-oxide	5.0	40	Biphenyl	-	Sealed	25	21
**8**	200	Pyridine-*N*-oxide	5.0	40	DMA	-	Sealed	26	-
**9**	200	Pyridine-*N*-oxide	5.0	40	1-Me-2-pyrrolidinone	-	Sealed	18	-
**10**	200	Pyridine-*N*-oxide	5.0	40	DMSO	-	Sealed	14	-
**11**	100	Pyridine-*N*-oxide	5.0	40	MeCN	-	Sealed	0	-
**12**	150	Pyridine-*N*-oxide	5.0	40	MeCN	-	Sealed	0	-
**13**	200	NMO	5.0	40	MeCN	-	Sealed	3	-
**14**	200	4-NO_2 Pyridine-*N*-oxide	5.0	40	MeCN	-	Sealed	22	23
**15**	200	Pyridine-*N*-oxide	5.0	40	MeCN[b]	-	Sealed	-	19
**16**	200	Pyridine-*N*-oxide	1.0	40	MeCN	-	Sealed	30	27
**17**	200	Pyridine-*N*-oxide	5.0	40	Camphor[c]	-	Sealed	24	27
**18**	200	Pyridine-*N*-oxide	5.0	20	Camphor	-	Sealed	-	27
**19**	200	Pyridine-*N*-oxide	5.0	60	Camphor	-	Sealed	-	31

**20**	200	Pyridine-*N*-oxide	5.0	40	Camphor	SiO_2 2.0 eq.	N_2 balloon	45	-
**21**	200	Pyridine-*N*-oxide	5.0	40	Camphor	Na_2SO_4 2.0 eq.	N_2 balloon	48	53
**22**	200	Pyridine-*N*-oxide	5.0	40	Camphor	$CaCl_2$ 2.0 eq.	N_2 balloon	51	44
**23**	200	Pyridine-*N*-oxide	5.0	40	Camphor	P_2O_5 2.0 eq.	N_2 balloon	-	Complex mixture
**24**	200	Pyridine-*N*-oxide	5.0	40	Camphor	H_2O 2.0 eq.	Sealed	36	-
**25**	200	-	-	40	Camphor	-	Sealed	17	-
**26**	200	-	-	40	Camphor	-	N_2 balloon	12	-
**27**	200	TMAO	5.0	40	Camphor	-	Sealed	5	-
**28**	200	Methyl pyridinium iodide	5.0	40	Camphor	-	Sealed	0	-
**29**	200	Pyridine	5.0	40	Camphor	-	Sealed	-	31
**30**	200	NaI	5.0	40	Camphor	-	Sealed	34	-
**31**	200	Imidazole	5.0	40	Camphor	-	N_2 balloon	5	-
**32**	200	DMAP	5.0	40	Camphor	-	N_2 balloon	8	-
**33**	200	KSCN	5.0	40	Camphor	-	N_2 balloon	17	-
**34**	200	BnSH	5.0	40	Camphor	-	N_2 balloon	18	-
**35**	200	H_2O	5.0	40	Camphor	-	N_2 balloon	17	-
**36**	200	Phenol	5.0	40	Camphor	-	N_2 balloon	7	-
**37**	200	BzOH	5.0	40	Camphor	-	N_2 balloon	11	-
**38**	200	Ph_3P	5.0	40	Camphor	-	N_2 balloon	1	-
**39**	200	NaBr	5.0	40	Camphor	-	N_2 balloon	15	-
**40**	230	Pyridine-*N*-oxide	5.0	40	Camphor	-	N_2 balloon	66	66
**41**	250	Pyridine-*N*-oxide	5.0	40	Camphor	-	N_2 balloon	80	62
**42**	250	Pyridine-*N*-oxide	5.0	40	Camphor	-	Sealed	66[a]	-
**43**	270	Pyridine-*N*-oxide	5.0	40	Camphor	-	Sealed	37	-
**44**	250	Pyridine	5.0	40	Camphor	-	N_2 balloon	6	-
**45**	250	Pyridine-*N*-oxide	5.0	40	Camphor	-	O_2 balloon	64	51

**46**	250	-	-	40	Camphor	-	O_2 balloon	28	-
**47**	250	Pyridine-*N*-oxide	5.0	40	Camphor	-	Ar balloon	75	65
**48**	250	-	-	40	Camphor	-	Ar balloon	9	-
**49**	250	Pyridine-*N*-oxide	5.0	20	Camphor	-	Sealed	48	-
**50**	250	Pyridine-*N*-oxide	5.0	30	Camphor	-	Sealed	76	63
**51**	250	Pyridine-*N*-oxide	5.0	40	Camphor	-	Sealed	74	-
**52**	250	Pyridine-*N*-oxide	5.0	50	Camphor	-	Sealed	34	-
**53**	250	Pyridine-*N*-oxide	5.0	60	Camphor	-	Sealed	66	-
**54**	250	Pyridine-*N*-oxide	5.0	40	Camphor	$CaCl_2$ 5.0 eq.	Sealed	28	-
**55**	250	Pyridine-*N*-oxide	5.0	40	Camphor	Na_2SO_4 5.0 eq.	Sealed	66	-
**56**	250	Pyridine-*N*-oxide	5.0	40	Camphor	-	N_2 balloon	80	88
**57**	250	Pyridine-*N*-oxide	5.0	40	Camphor	-	N_2 balloon	31	-
**58**	250	Pyridine-*N*-oxide	5.0	40	Camphor	-	N_2 balloon	31	-
**59**	250	Pyridine-*N*-oxide	1.0	40	Camphor	-	N_2 balloon	73	80
**60**	250	Pyridine-*N*-oxide	3.0	40	Camphor	-	N_2 balloon	60	-
**61**	250	Pyridine-*N*-oxide	10	40	Camphor	-	N_2 balloon	78	71
**62**	250	Pyridine-*N*-oxide	1.0	40	Camphor	-	N_2 balloon	73	58
**63**	250	Pyridine-*N*-oxide	1.0	40	Camphor	-	N_2 balloon	74	66
**64**	250	Pyridine-*N*-oxide	1.0	40	Camphor	-	N_2 balloon	62	-
**65**	250	Pyridine-*N*-oxide	1.0	40	Camphor	-	Sealed	70	57
**66**	250	Pyridine-*N*-oxide	1.0	40	Camphor	-	Sealed	63	-
**67**	250	Pyridine-*N*-oxide	1.0	40	Camphor	-	Sealed	68	-
**68**	250	Pyridine-*N*-oxide	1.5	40	Camphor	-	Sealed	-	65
**69**	250	Pyridine-*N*-oxide	1.5	40	Camphor	-	Sealed	-	69
**70**	250	Pyridine-*N*-oxide	2.0	40	Camphor	-	Sealed	-	73

**71**	250	Pyridine-*N*-oxide	2.0	40	Camphor	-	Ar	-	63

Sealed indicates that the reaction was sealed with a microwave vial cap under atmospheric conditions. [a] ^{1}H NMR yield with trimethoxy benzene as an internal standard. [b]0.1 mL MeCN used as solvent. [c]0.5 g camphor used as solvent.

Note on the use of balloons. The use of balloons on the reaction vial occasionally produced a higher yield than our final optimized yield (eg entry 56), however this factor created reproducibility issues due to needles periodically blocking with solidified camphor and some high yields could not be easily repeated.

Further method elucidation experiments:

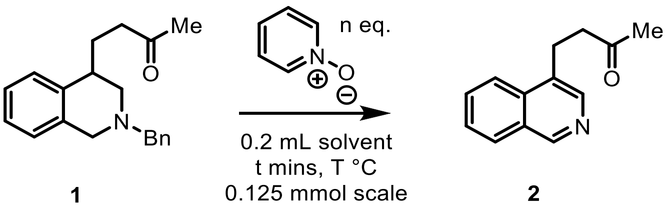

#	°C	Reagent	Reagent eq.	Time (mins)	Solvent	Additive	Headspace	Isolated Yield (%)
**1**	250	Pyridine-*N*-oxide	1.5	40	Camphor	-	Air	69
**2**	250	-	-	40	Camphor	-	Air	21
**3**	250	Pyridine-*N*-oxide	1.5	40	Camphor	-	Ar	52
**4**	250	-	-	40	Camphor	-	Ar	3
**5**	250	Pyridine-*N*-oxide	1.5	40	Camphor	-	O_2	68
**6**	250	NaI	1.5	40	Camphor	-	Air	43
**7**	250	Ph_3PO	1.5	40	Camphor	-	Air	21
**8**	250	4-NO_2 Pyridine-*N*-oxide	1.5	40	Camphor	-	Air	33
**9**	250	4-Ph Pyridine-*N*-oxide	1.5	40	Camphor	-	Air	55

Investigations with an *N*-Me THIQ substrate:

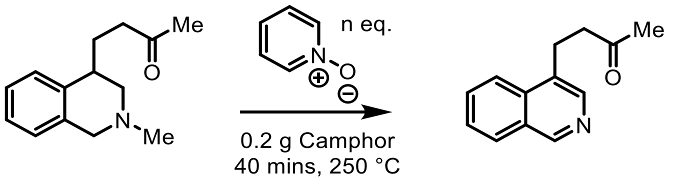

S6 **2**

#	°C	Reagent	Reagent eq.	Time (mins)	Solvent	Additive	Headspace	RP HPLC Yield (%)	Isolated Yield (%)
**1**	250	Pyridine-*N*-oxide	1.5	40	Camphor	-	Air	-	19
**2**	250	-	-	40	Camphor	-	Air	-	6
**3**	250	Pyridine-*N*-oxide	1.5	40	Camphor	-	Ar	-	10

Failed Substrates:

In all cases the failed substrates gave multicomponent mixtures with only traces of product; no starting material was recovered in any case.

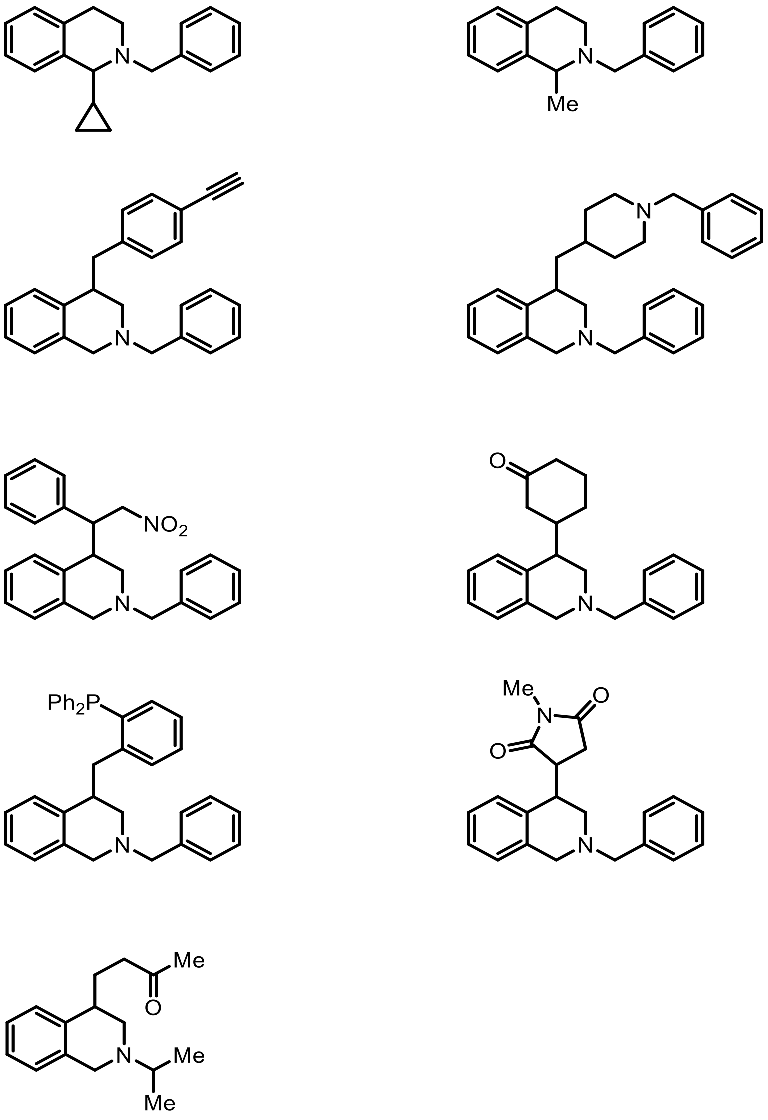

4. NMR Spectra

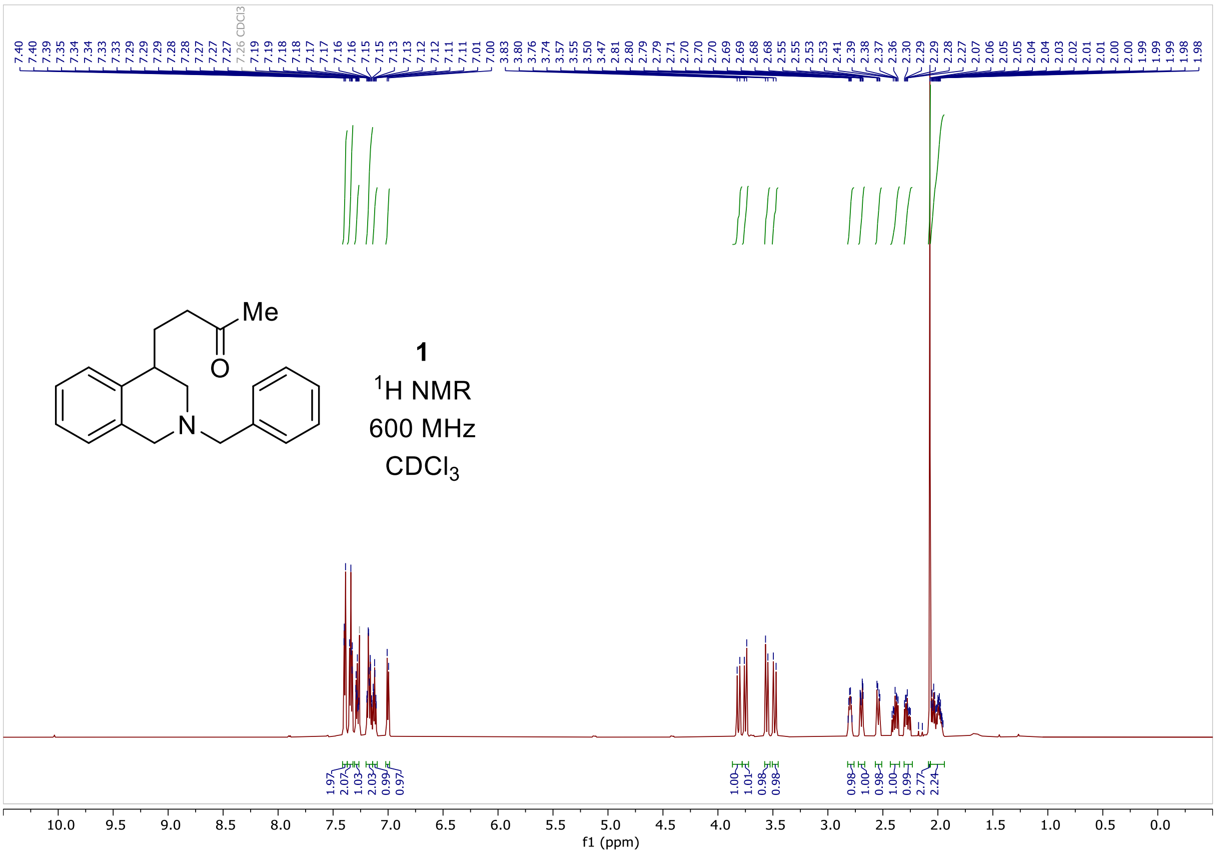

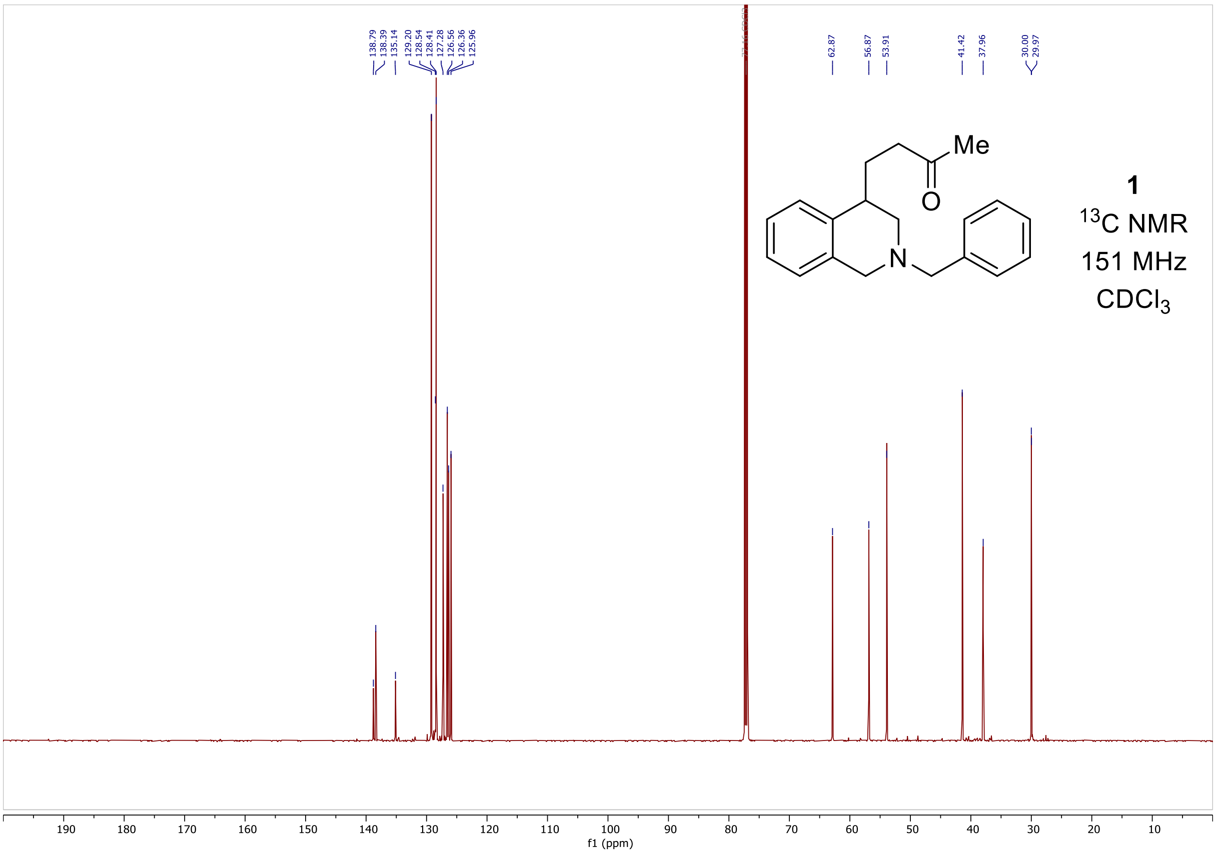

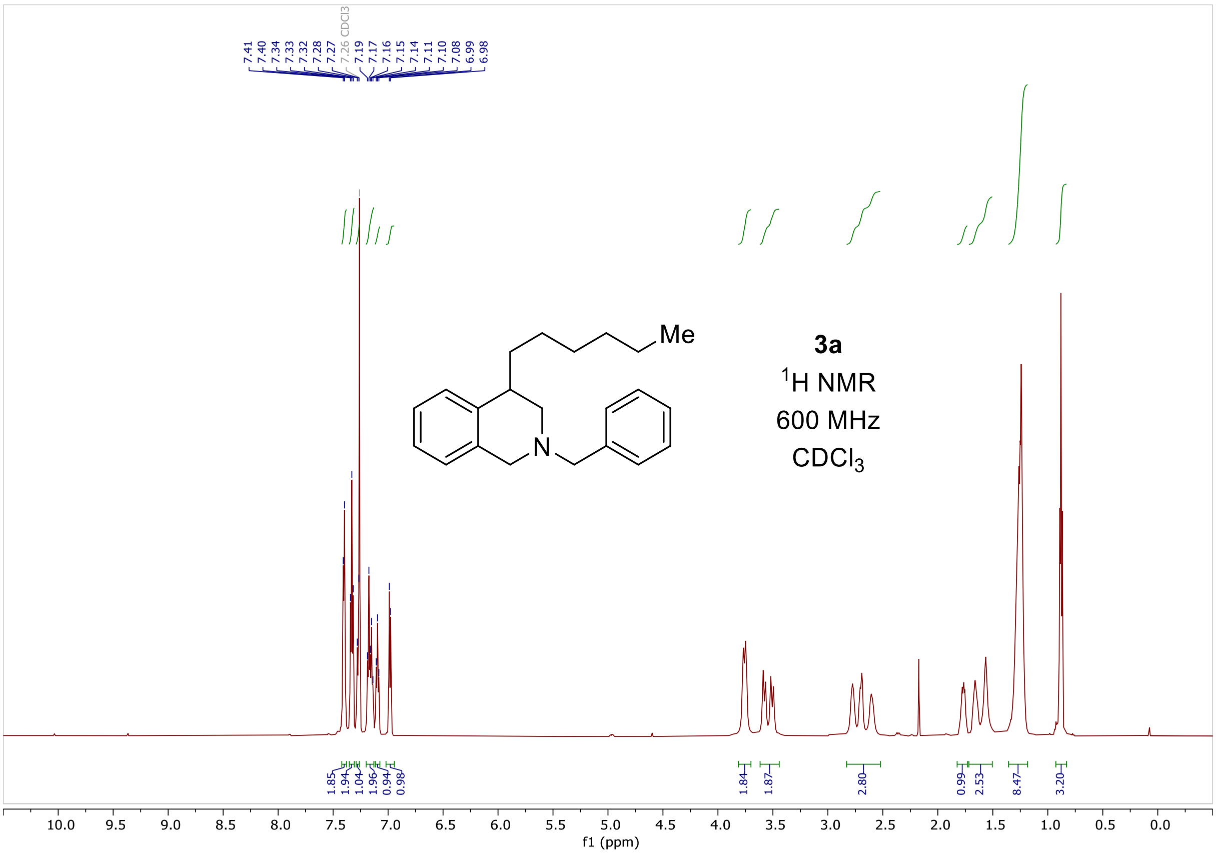
7.41
7.40
7.34
7.33
7.32
7.28
7.27
7.26 CDCl3
7.19
7.17
7.16
7.15
7.14
7.11
7.10
7.08
6.99
6.98
Me
N
3a
1H NMR
600 MHz
CDCl3
1.85
1.94
1.04
1.96
0.94
0.98
1.84
1.87
2.80
0.99
2.53
8.47
3.20
f1 (ppm)

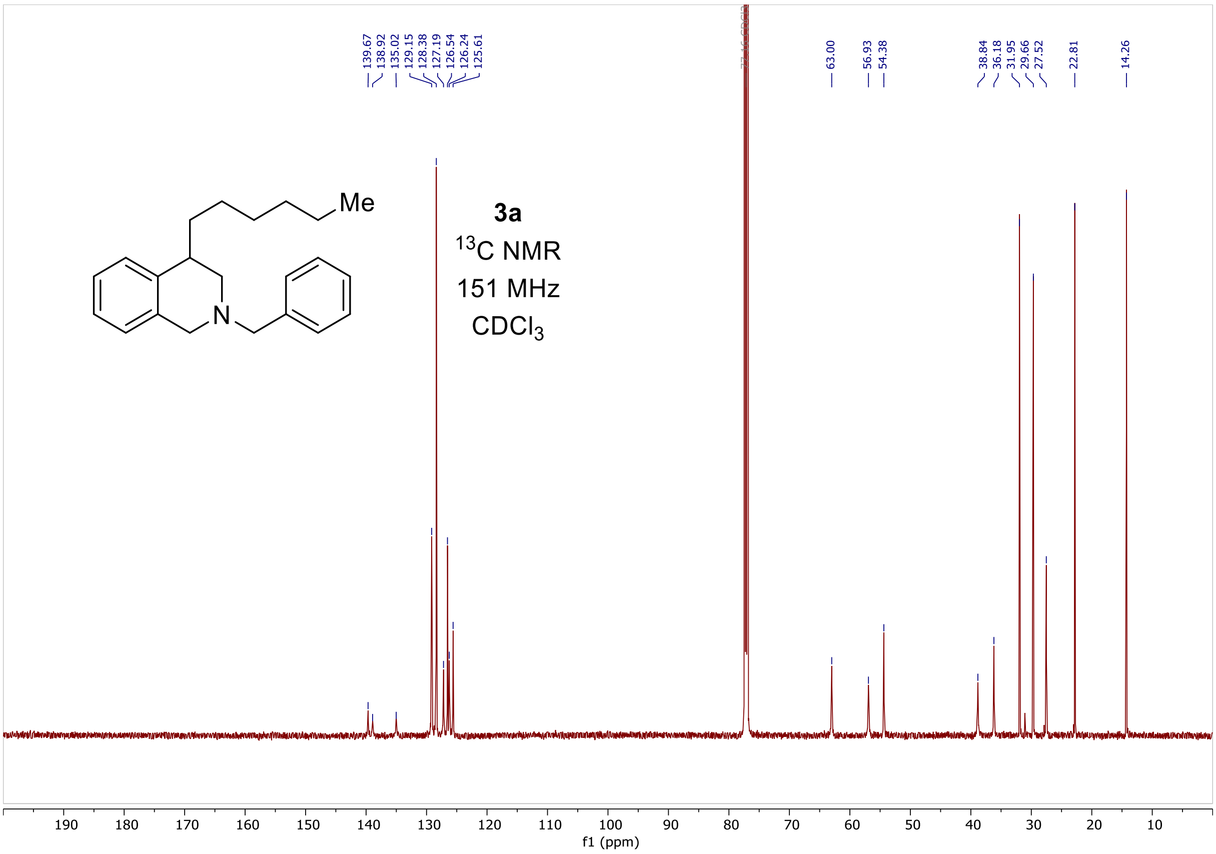
139.67
138.92
135.02
129.15
128.38
127.19
126.54
126.24
125.61
63.00
56.93
54.38
38.84
36.18
31.95
29.66
27.52
22.81
14.26
Me
N
3a
13C NMR
151 MHz
CDCl3
f1 (ppm)

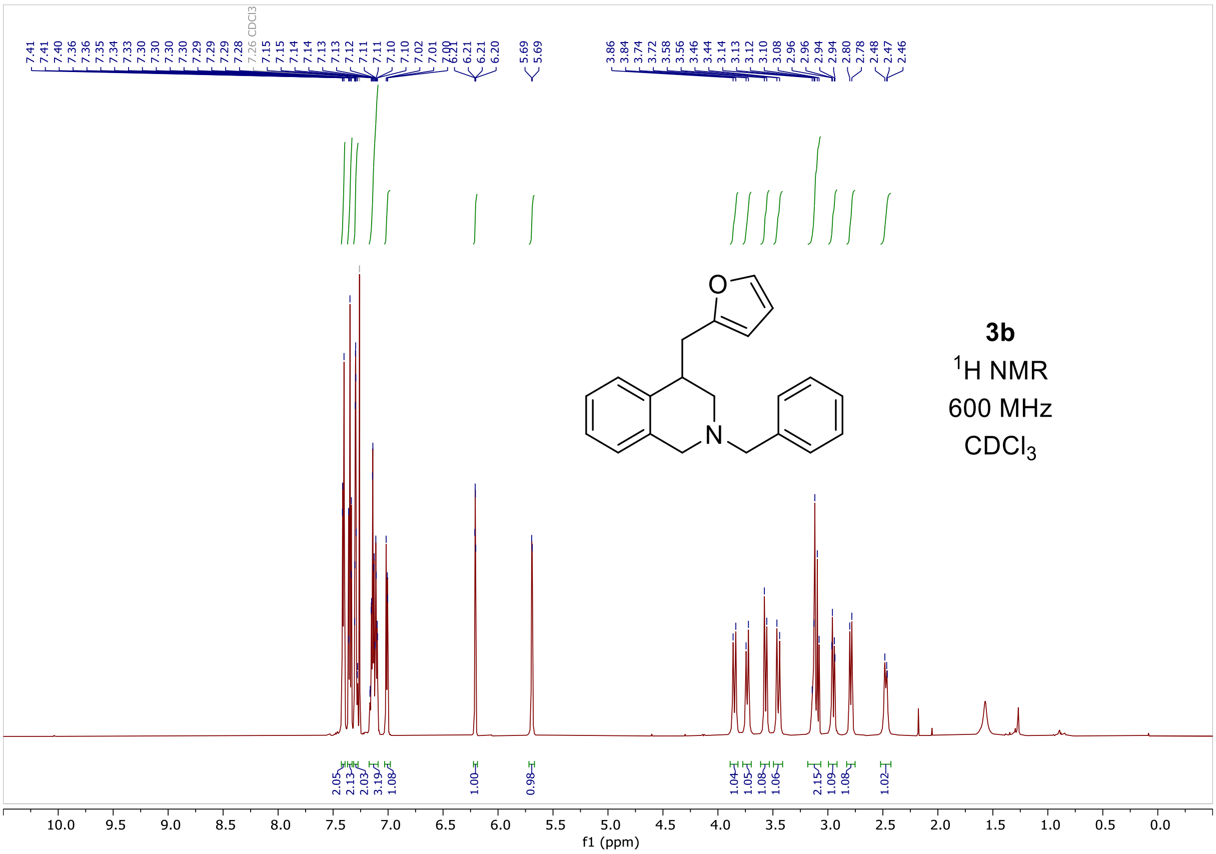
3b
^{1}H NMR
600 MHz
$CDCl_3$
f1 (ppm)

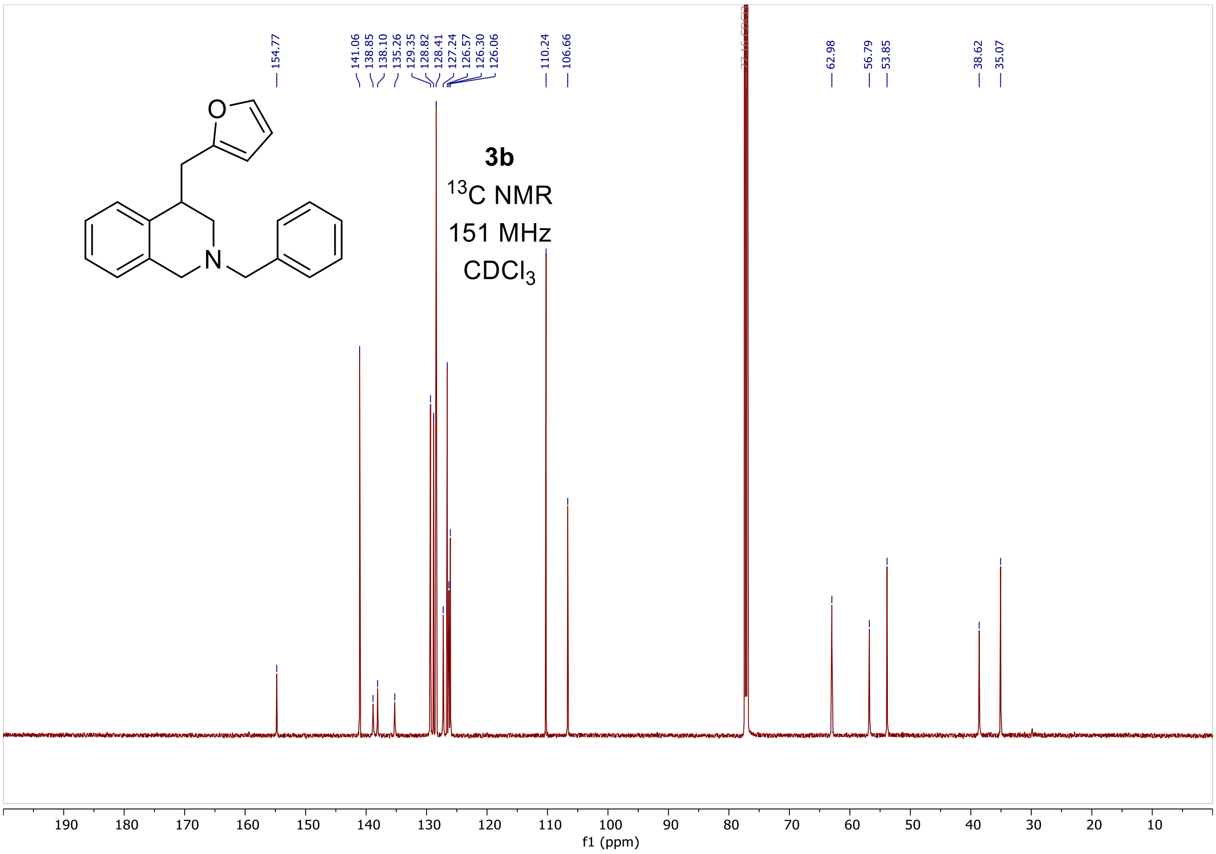
3b
^{13}C NMR
151 MHz
$CDCl_3$
f1 (ppm)

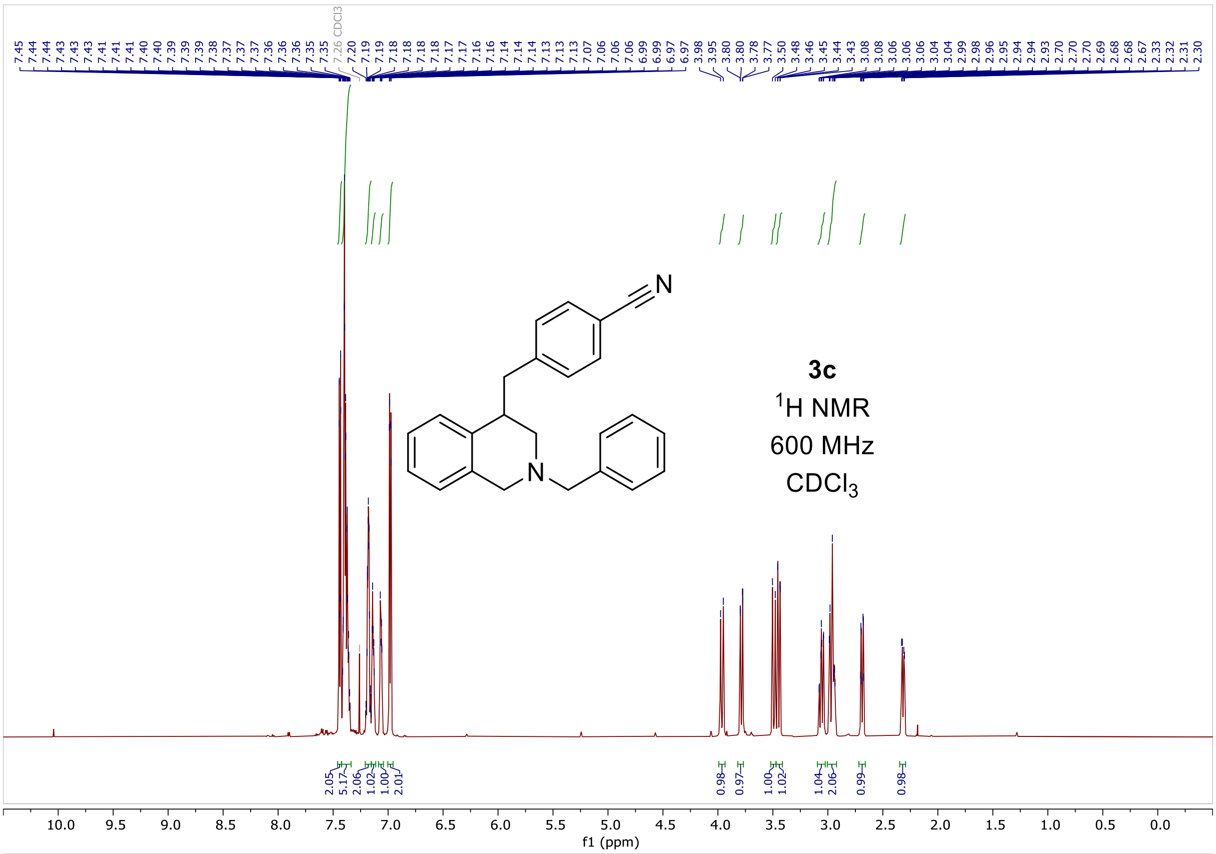
7.45 7.44 7.44 7.43 7.43 7.43 7.41 7.41 7.41 7.40 7.39 7.39 7.39 7.38 7.37 7.37 7.36 7.36 7.35 7.35 7.26 CDCl3 7.20 7.19 7.19 7.18 7.18 7.18 7.17 7.17 7.16 7.14 7.14 7.14 7.13 7.13 7.13 7.07 7.06 7.06 6.99 6.99 6.97 6.97 3.98 3.95 3.80 3.78 3.77 3.50 3.48 3.46 3.45 3.44 3.43 3.08 3.08 3.06 3.06 3.06 3.04 3.04 2.99 2.98 2.96 2.95 2.94 2.94 2.93 2.70 2.70 2.70 2.69 2.68 2.67 2.33 2.32 2.31 2.30
N
N
3c
^{1}H NMR
600 MHz
$CDCl_3$
2.05 5.17 2.06 1.02 1.00 2.01 0.98 0.97 1.00 1.02 1.04 2.06 0.99 0.98
10.0 9.5 9.0 8.5 8.0 7.5 7.0 6.5 6.0 5.5 5.0 4.5 4.0 3.5 3.0 2.5 2.0 1.5 1.0 0.5 0.0
f1 (ppm)

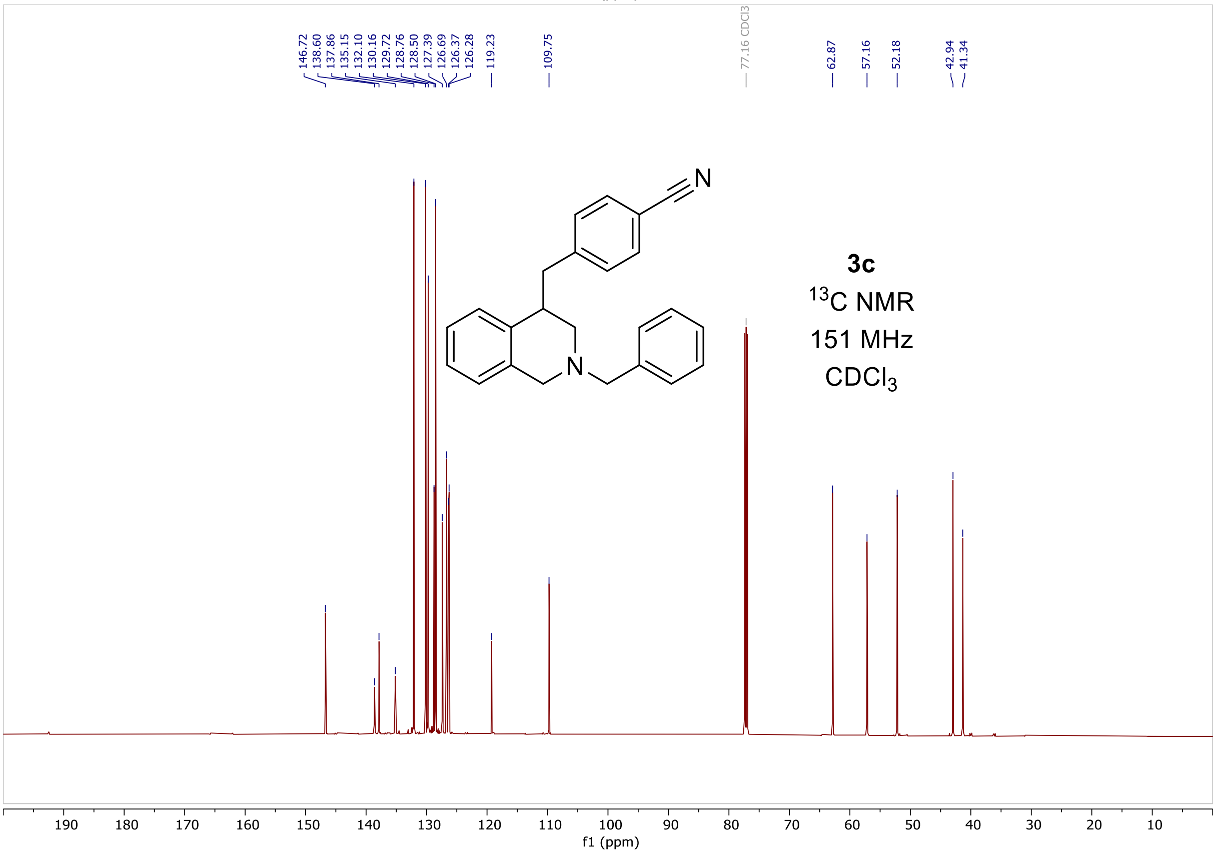
146.72 138.60 137.86 135.15 132.10 130.16 129.72 128.76 128.50 127.39 126.69 126.37 126.28 119.23 109.75 77.16 CDCl3 62.87 57.16 52.18 42.94 41.34
N
N
3c
^{13}C NMR
151 MHz
$CDCl_3$
190 180 170 160 150 140 130 120 110 100 90 80 70 60 50 40 30 20 10
f1 (ppm)

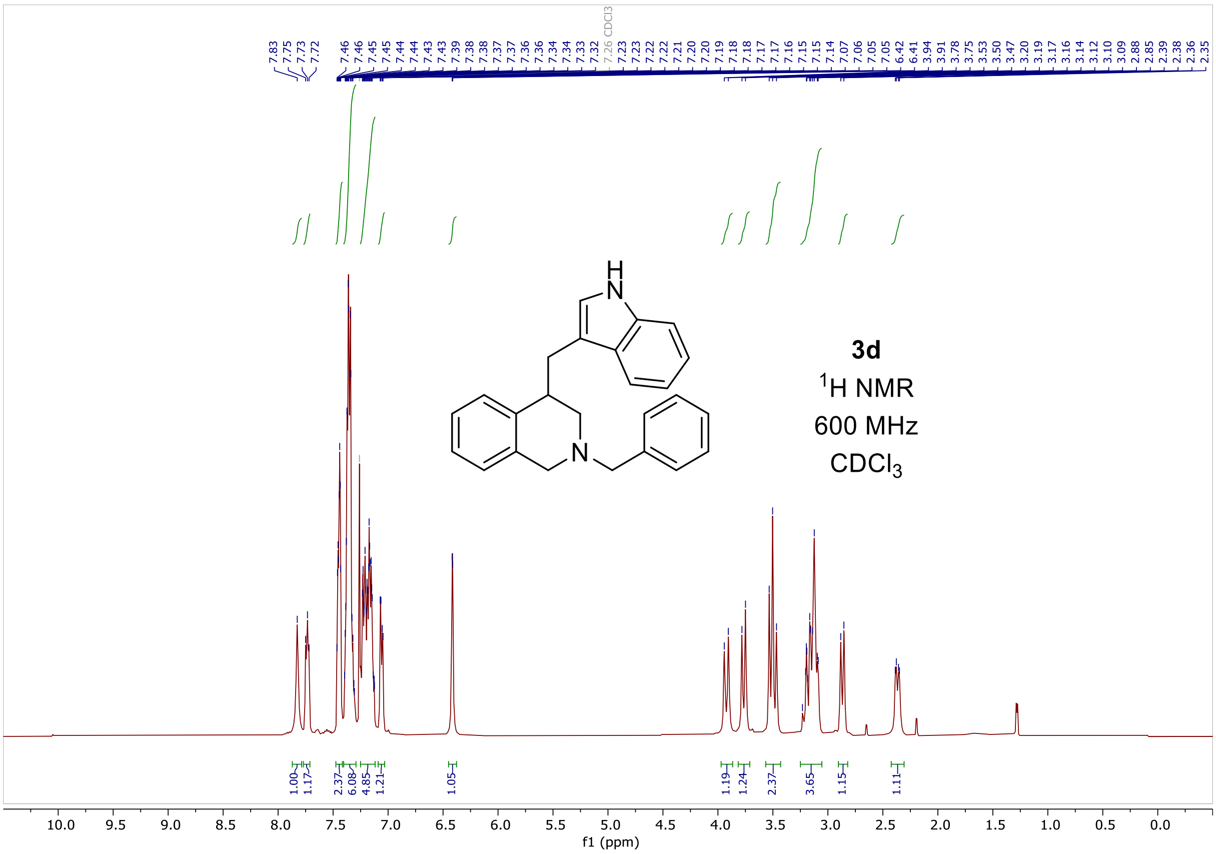
3d
^{1}H NMR
600 MHz
$CDCl_3$
f1 (ppm)

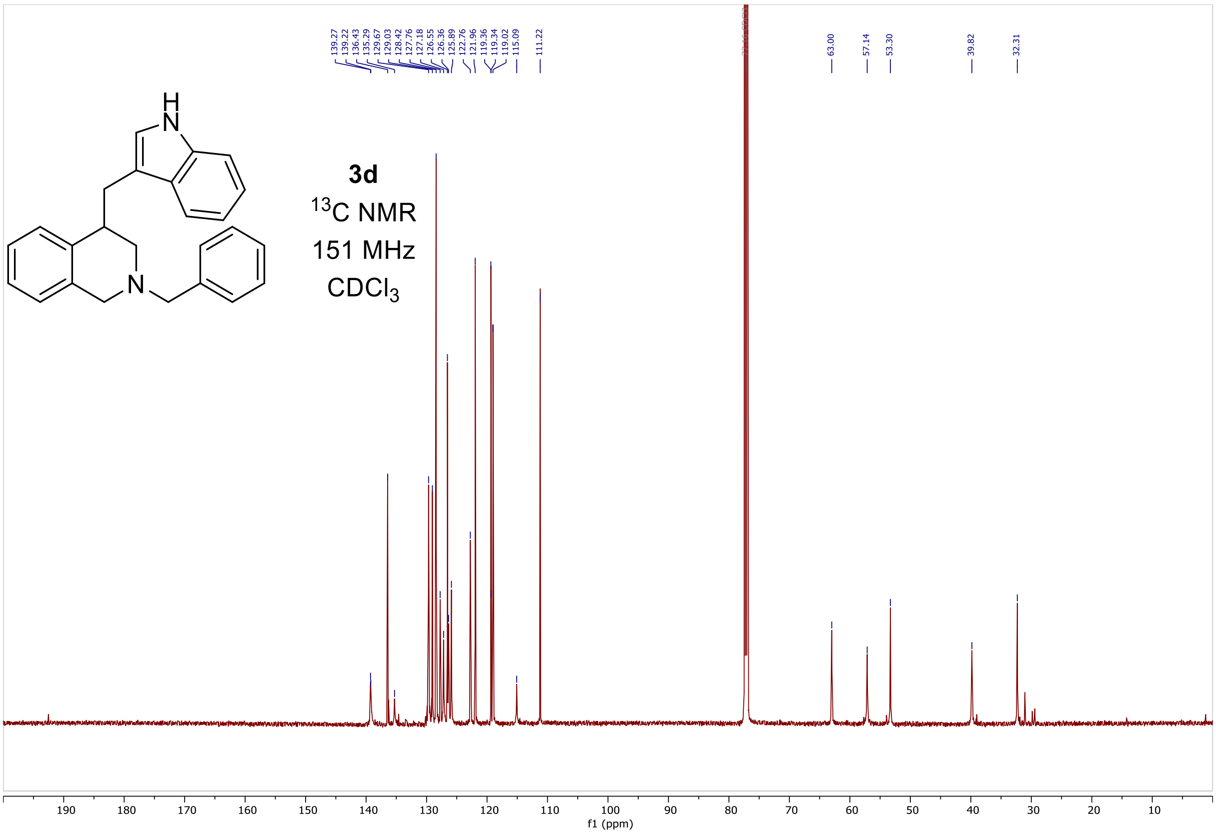
3d
^{13}C NMR
151 MHz
$CDCl_3$
f1 (ppm)

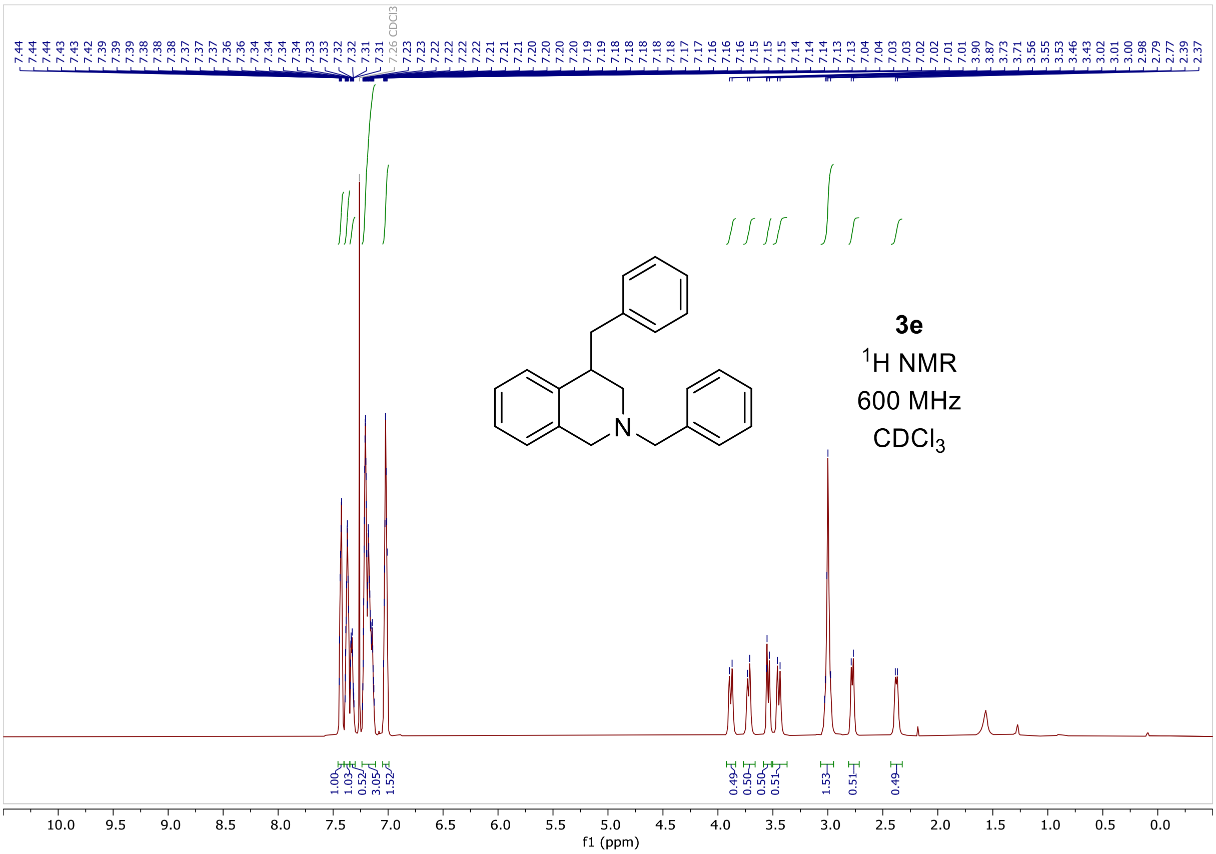
3e
^{1}H NMR
600 MHz
$CDCl_3$
f1 (ppm)

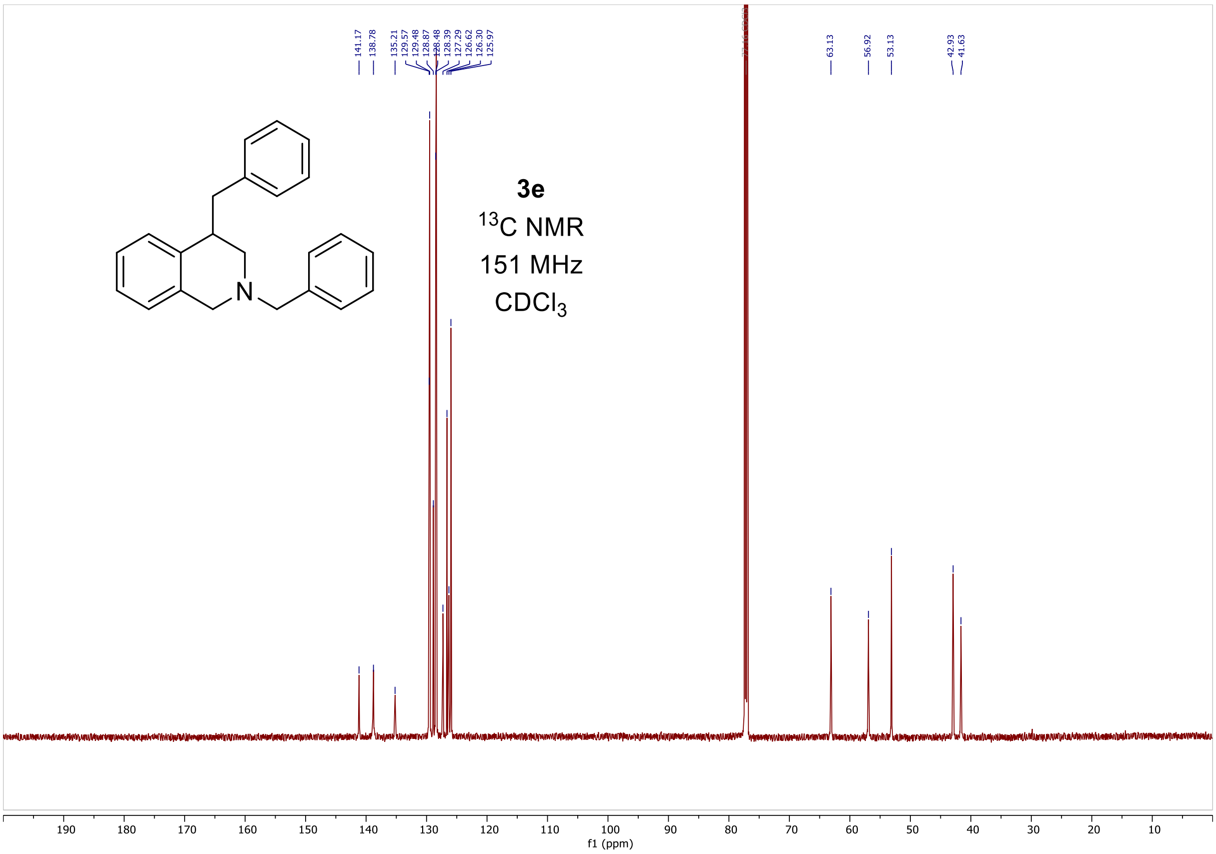
3e
^{13}C NMR
151 MHz
$CDCl_3$
141.17
138.78
135.21
129.57
129.48
128.87
128.48
128.39
127.29
126.62
126.30
125.97
63.13
56.92
53.13
42.93
41.63
f1 (ppm)

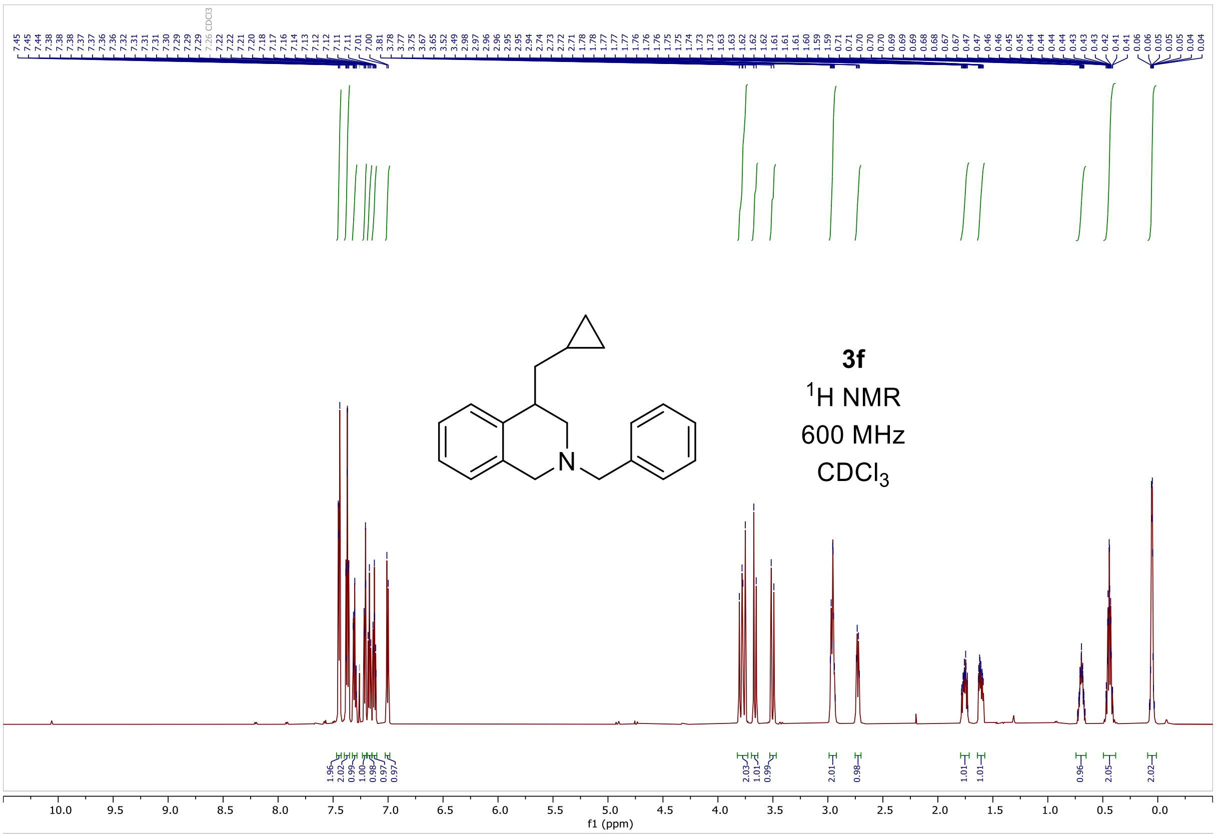
3f
1H NMR
600 MHz
CDCl3
f1 (ppm)

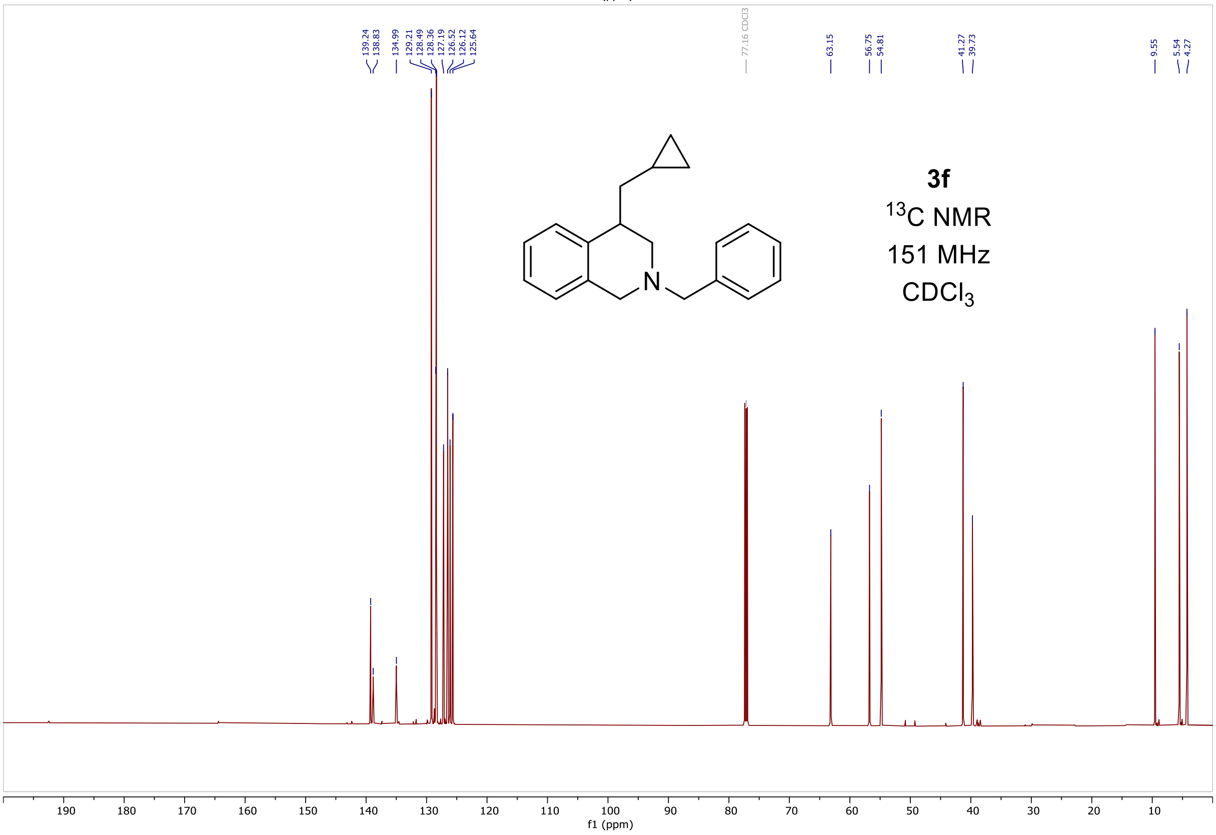
139.24
138.83
134.99
129.21
128.49
128.36
127.19
126.52
126.12
125.64
77.16 CDCl3
63.15
56.75
54.81
41.27
39.73
9.55
5.54
4.27
3f
13C NMR
151 MHz
CDCl3
f1 (ppm)

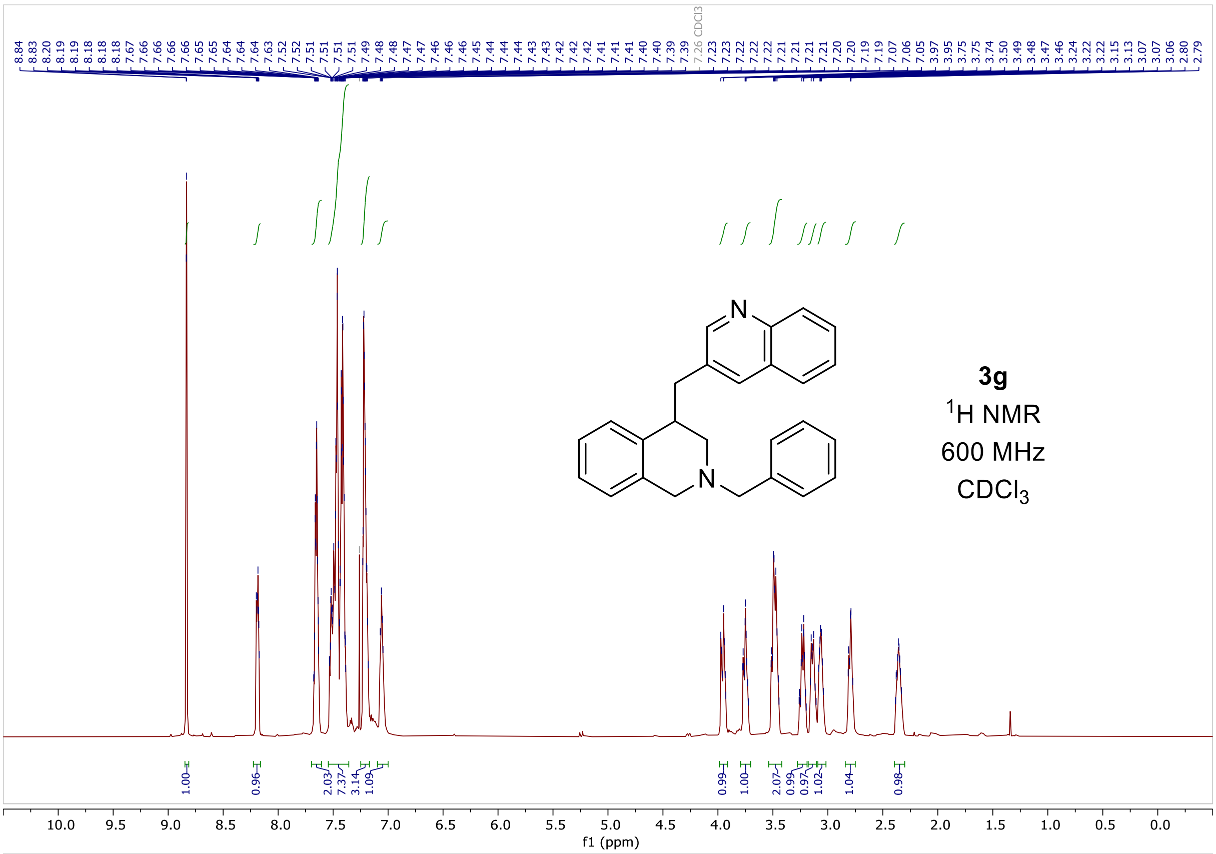
3g
^{1}H NMR
600 MHz
$CDCl_3$
f1 (ppm)

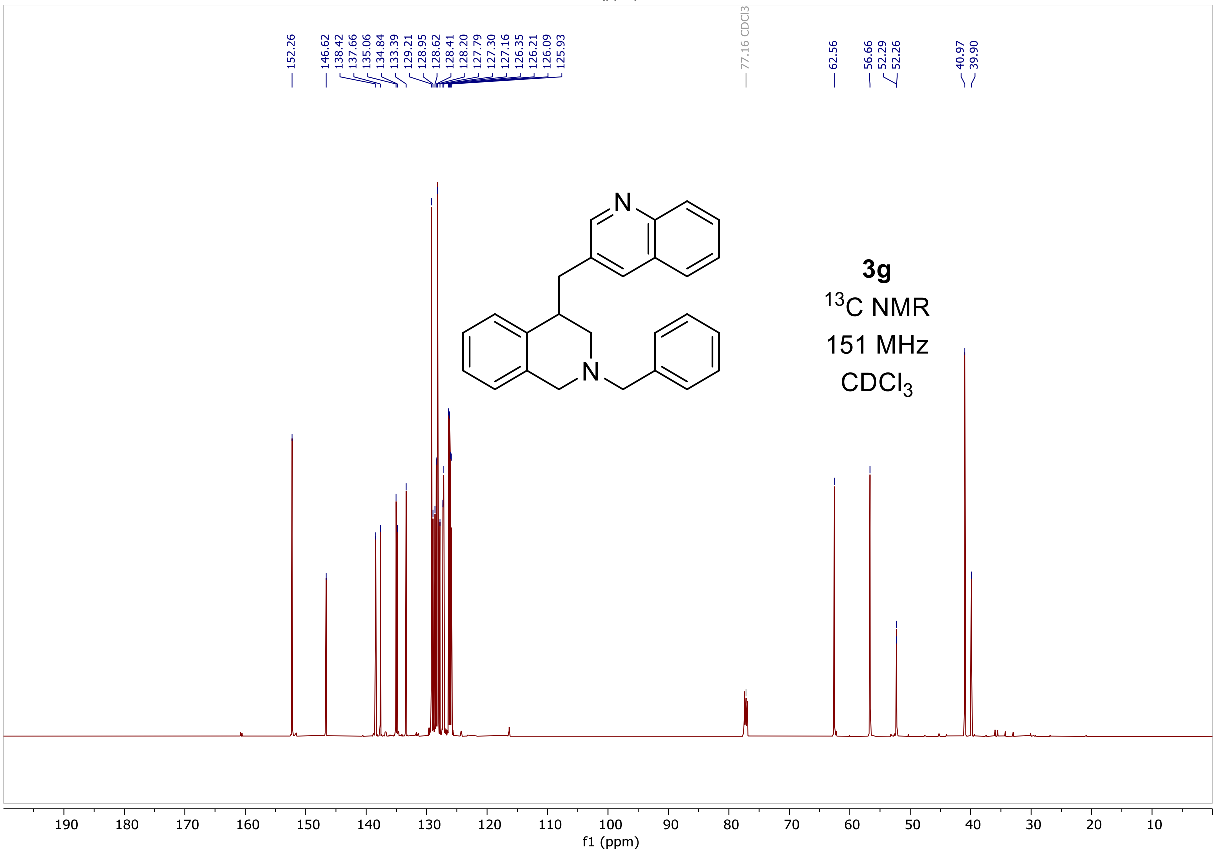
3g
^{13}C NMR
151 MHz
$CDCl_3$
152.26
146.62
138.42
137.66
135.06
134.84
133.39
129.21
128.95
128.62
128.41
128.20
127.79
127.30
127.16
126.35
126.21
126.09
125.93
77.16 CDCl3
62.56
56.66
52.29
52.26
40.97
39.90
f1 (ppm)

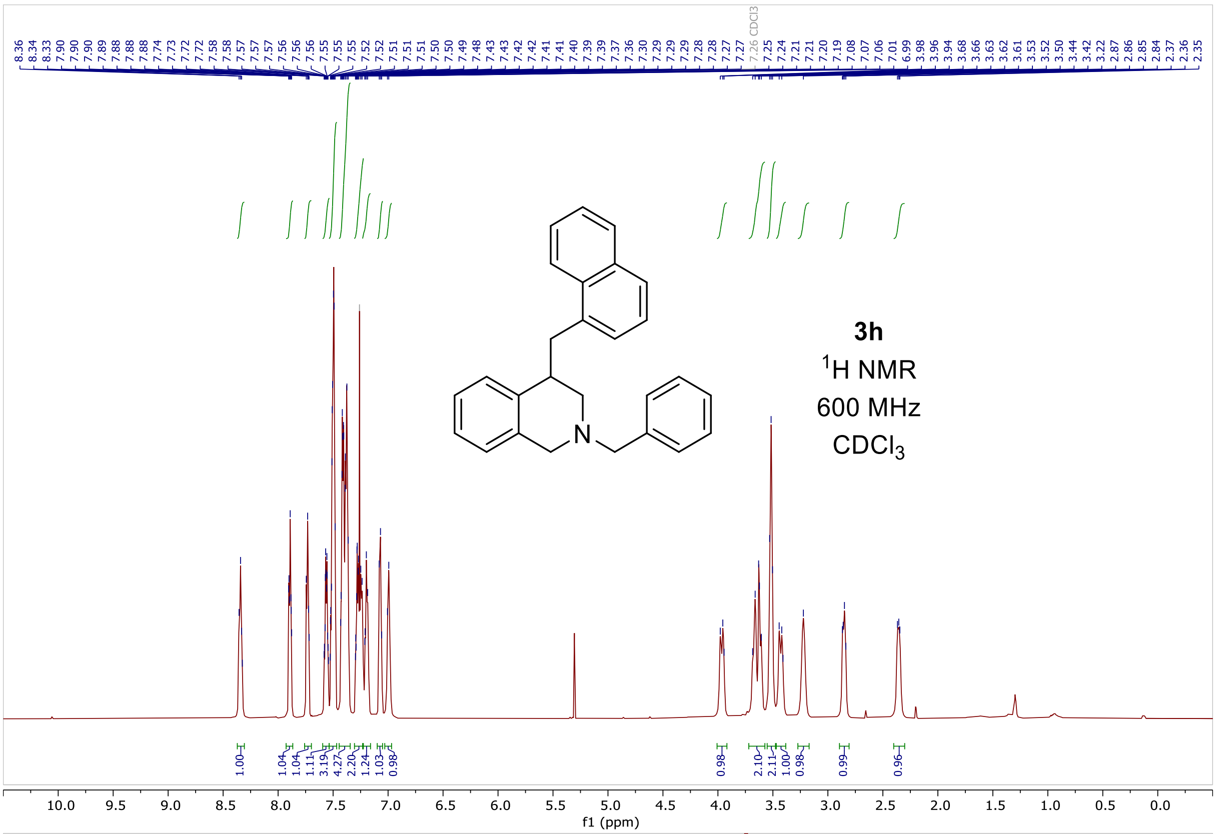
8.36
8.34
8.33
7.90
7.90
7.89
7.88
7.88
7.88
7.74
7.73
7.72
7.72
7.58
7.57
7.57
7.56
7.56
7.56
7.55
7.55
7.55
7.52
7.52
7.51
7.51
7.50
7.50
7.49
7.48
7.43
7.43
7.42
7.42
7.41
7.40
7.39
7.39
7.37
7.36
7.30
7.29
7.29
7.29
7.28
7.27
7.27
7.26 CDCl3
7.25
7.24
7.21
7.21
7.20
7.19
7.08
7.07
7.06
7.01
6.99
3.98
3.96
3.94
3.68
3.66
3.63
3.62
3.61
3.53
3.52
3.50
3.44
3.42
3.22
2.87
2.86
2.85
2.84
2.37
2.36
2.35
N
3h
1H NMR
600 MHz
CDCl3
1.00
1.04
1.04
1.11
3.19
4.27
2.20
1.24
1.03
0.98
0.98
2.10
2.11
1.00
0.98
0.99
0.96
10.0
9.5
9.0
8.5
8.0
7.5
7.0
6.5
6.0
5.5
5.0
4.5
4.0
3.5
3.0
2.5
2.0
1.5
1.0
0.5
0.0
f1 (ppm)

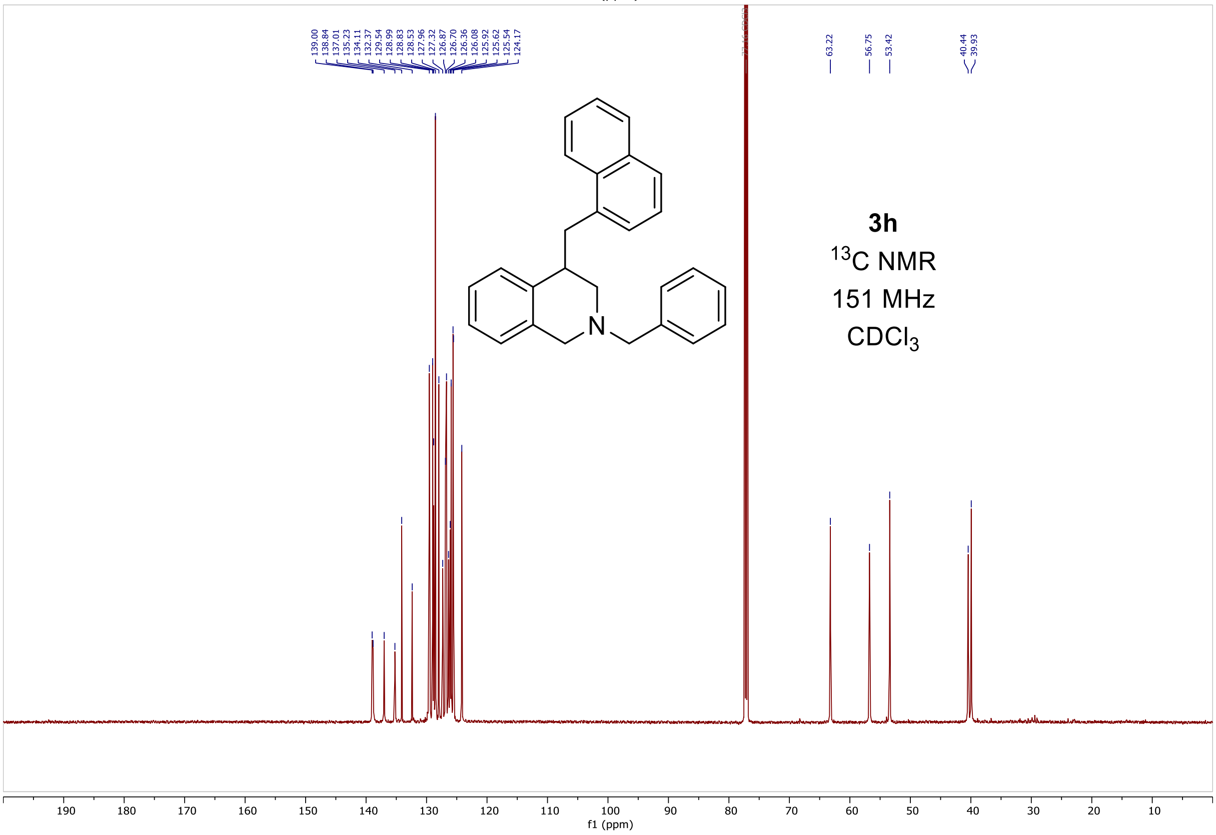
139.00
138.84
137.01
135.23
134.11
132.37
129.54
128.99
128.83
128.53
127.96
127.32
126.87
126.70
126.36
126.08
125.92
125.62
125.54
124.17
63.22
56.75
53.42
40.44
39.93
N
3h
13C NMR
151 MHz
CDCl3
190
180
170
160
150
140
130
120
110
100
90
80
70
60
50
40
30
20
10
f1 (ppm)

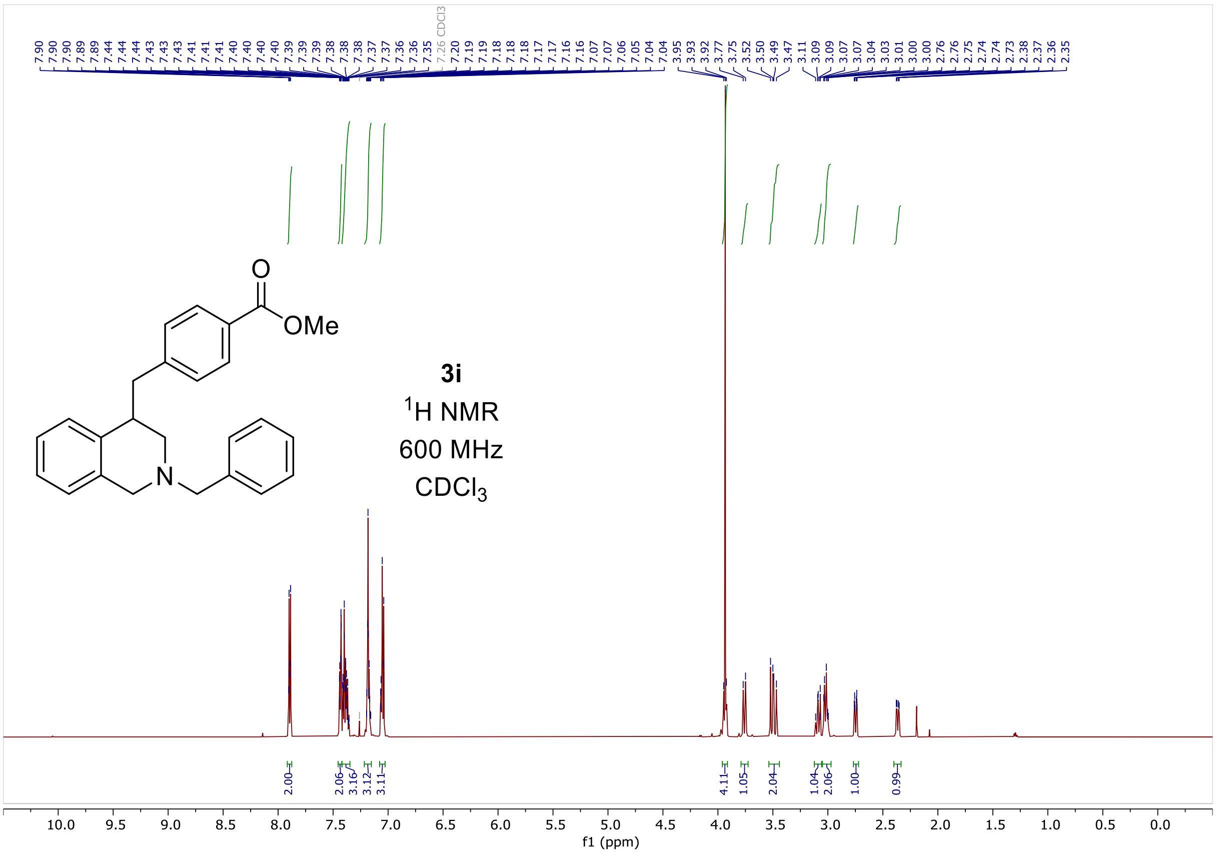
3i
1H NMR
600 MHz
CDCl3
OMe
N
O
f1 (ppm)

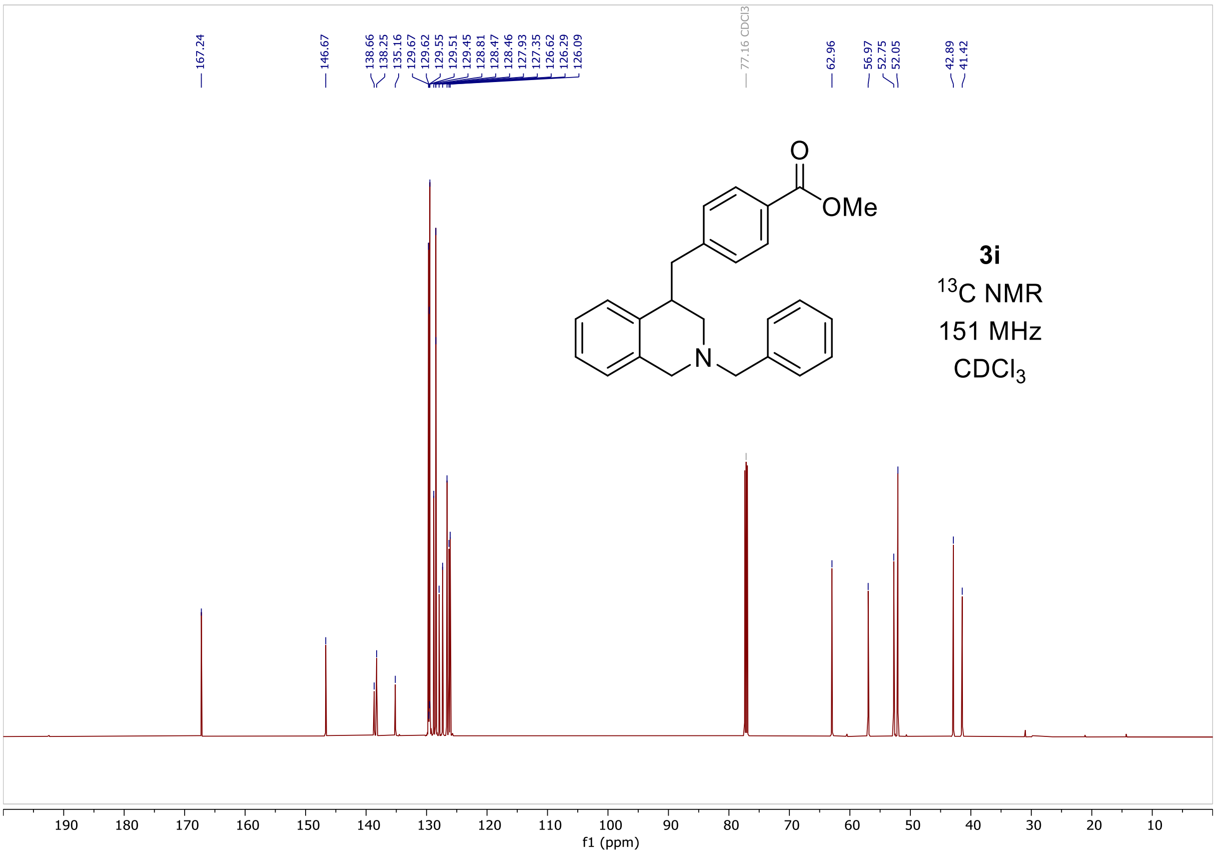
3i
13C NMR
151 MHz
CDCl3
OMe
N
O
f1 (ppm)

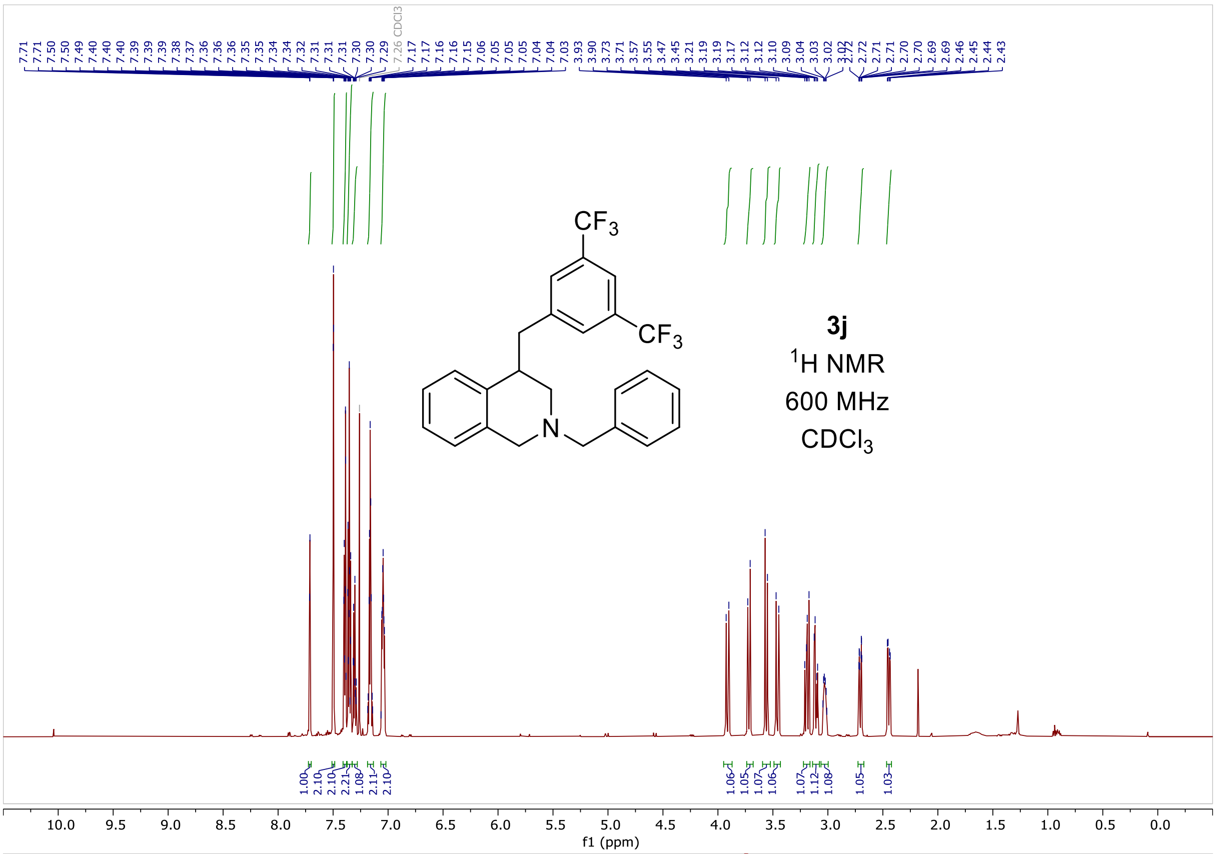
3j
^{1}H NMR
600 MHz
CDCl$_3$
f1 (ppm)

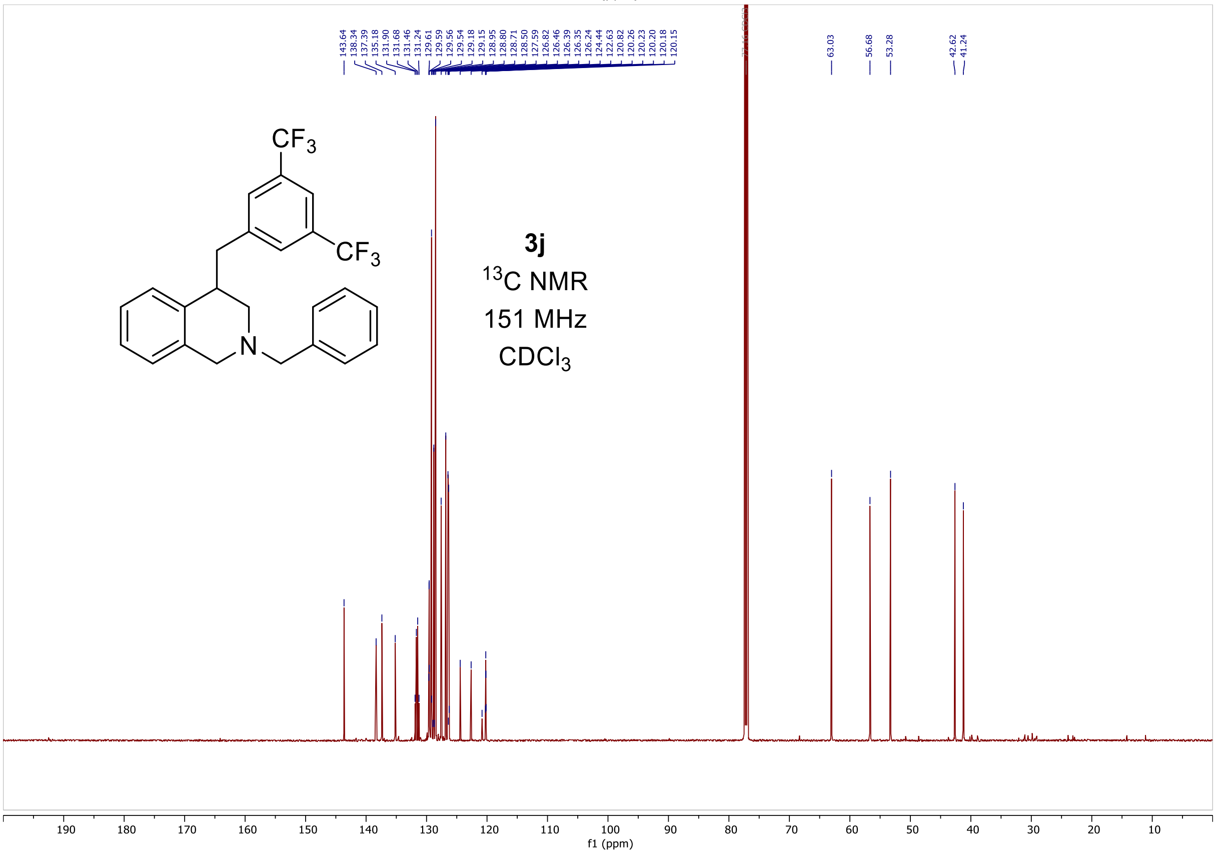
3j
^{13}C NMR
151 MHz
CDCl$_3$
f1 (ppm)

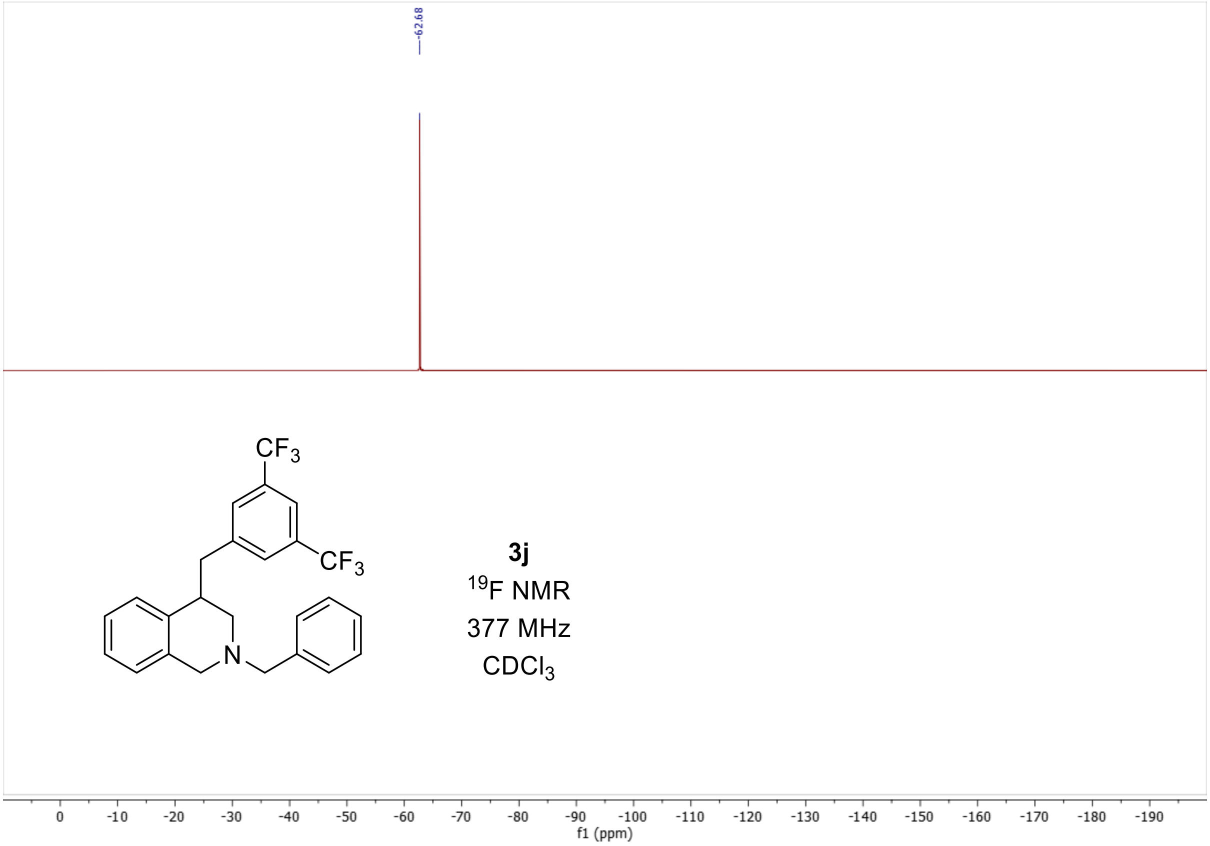

3j
^{19}F NMR
377 MHz
$CDCl_3$

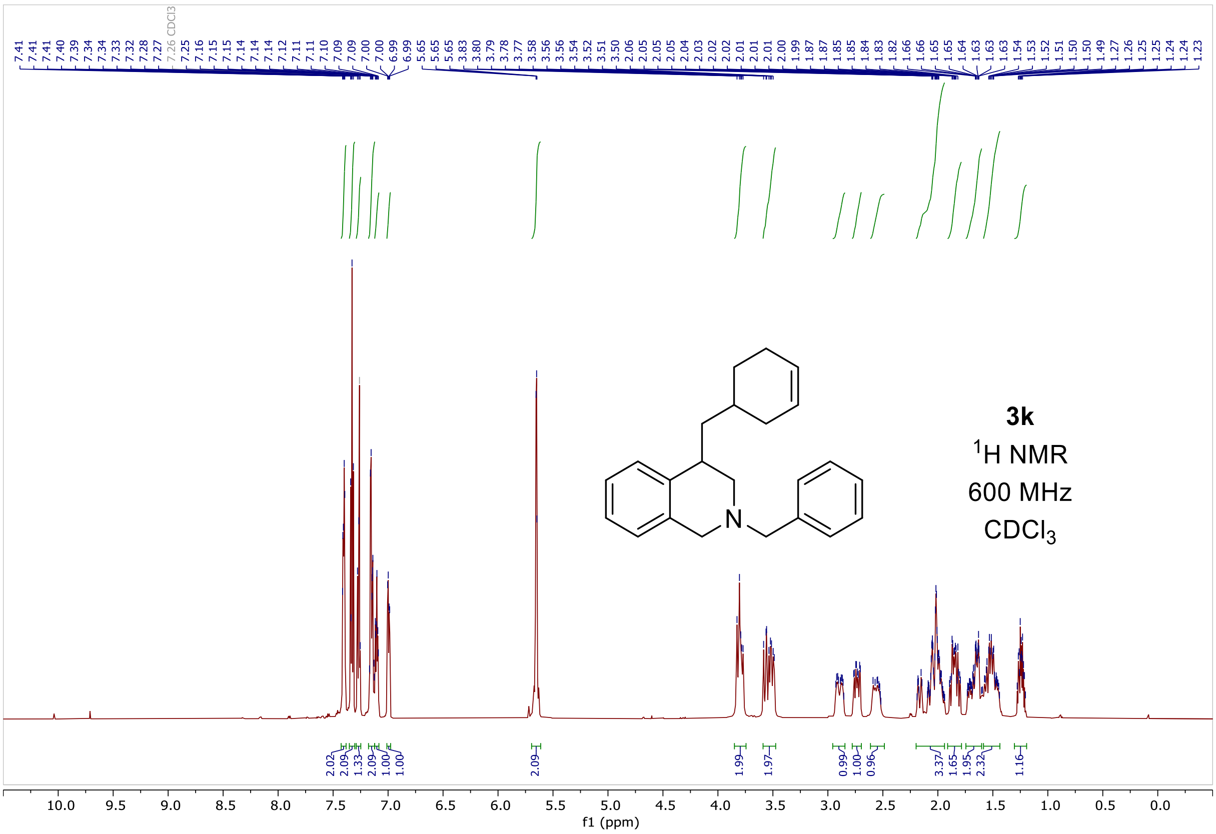
3k
^{1}H NMR
600 MHz
$CDCl_3$
f1 (ppm)

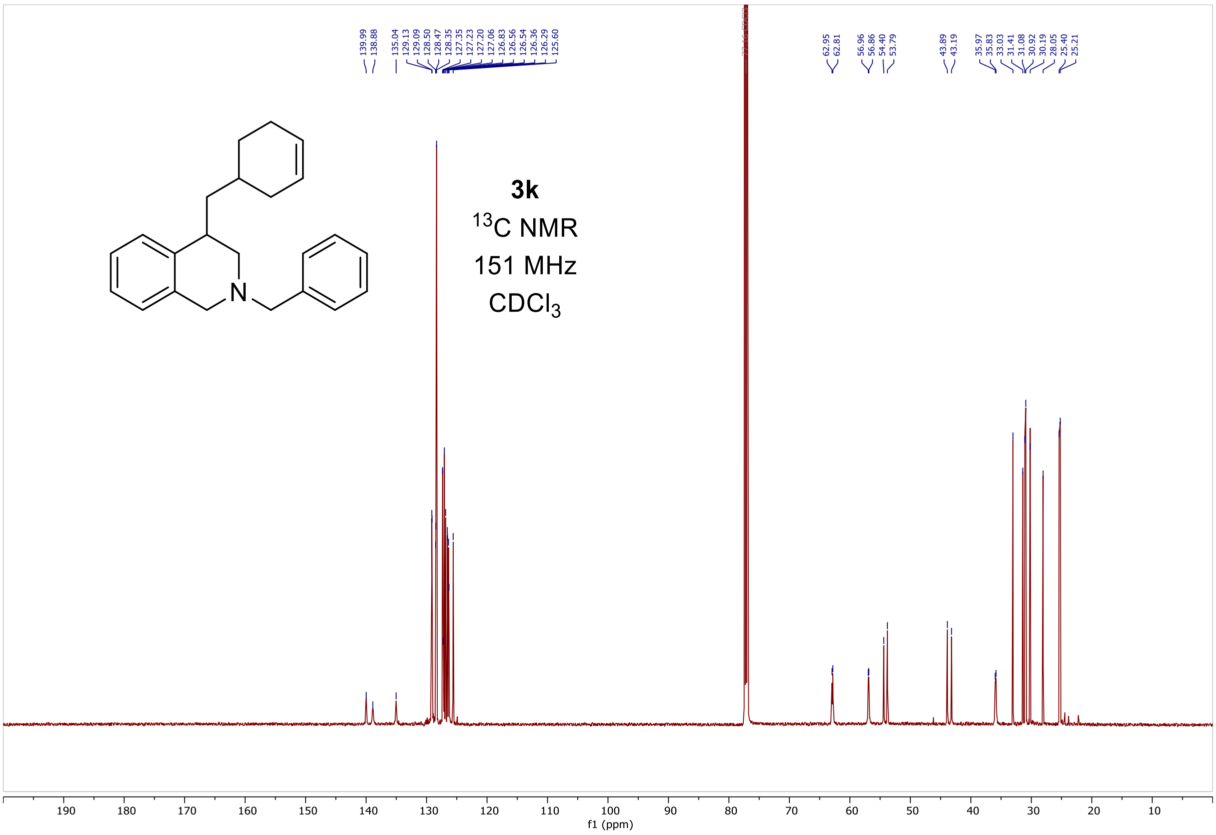
3k
^{13}C NMR
151 MHz
$CDCl_3$
f1 (ppm)

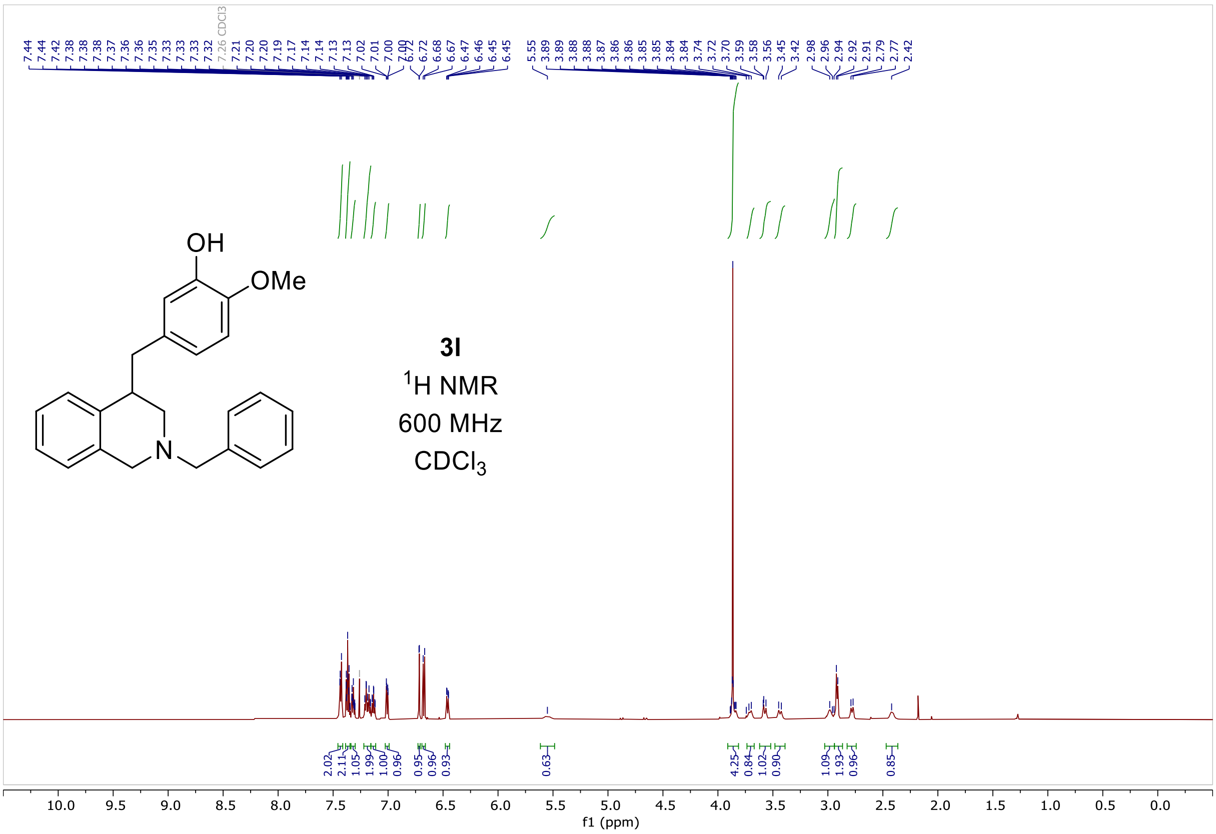
OH
OMe
N
3l
¹H NMR
600 MHz
CDCl3
f1 (ppm)

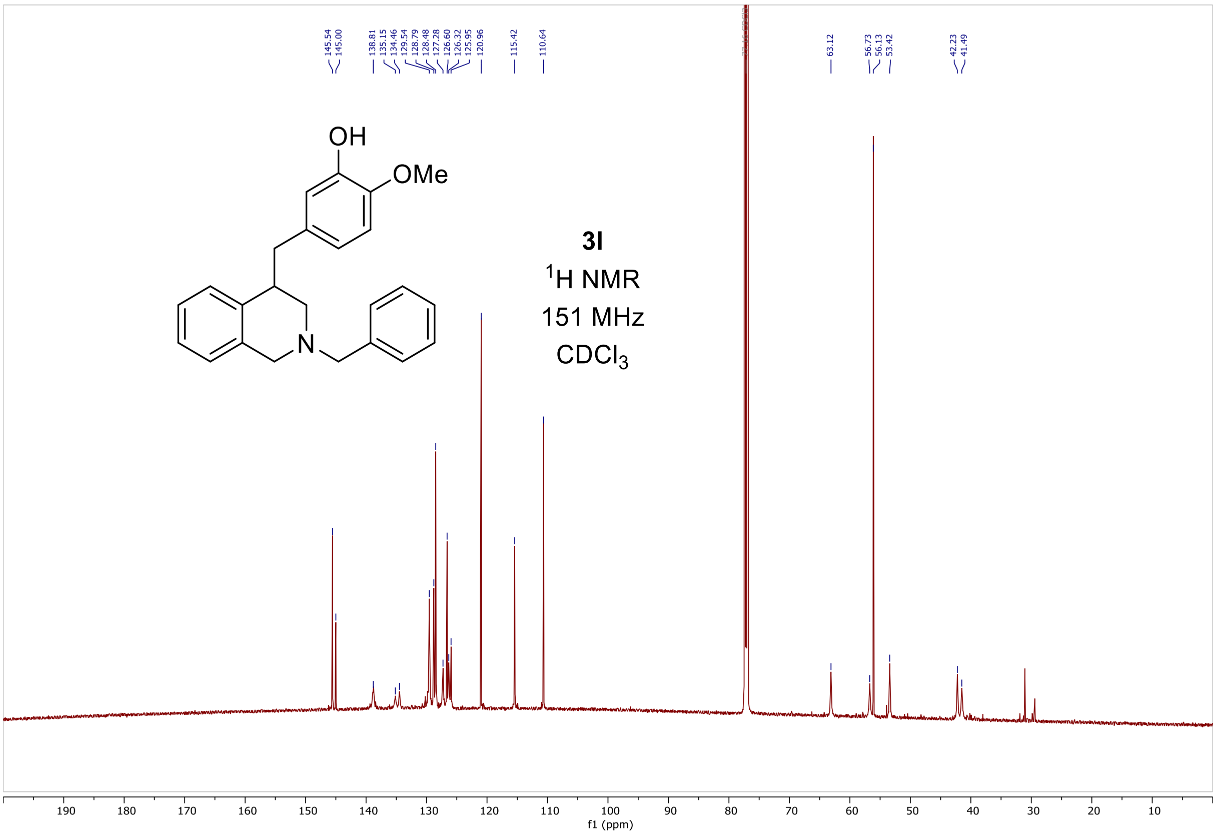
145.54
145.00
138.81
135.15
134.46
129.54
128.79
128.48
127.28
126.60
126.32
125.95
120.96
115.42
110.64
63.12
56.73
56.13
53.42
42.23
41.49
OH
OMe
N
3l
¹H NMR
151 MHz
CDCl3
f1 (ppm)

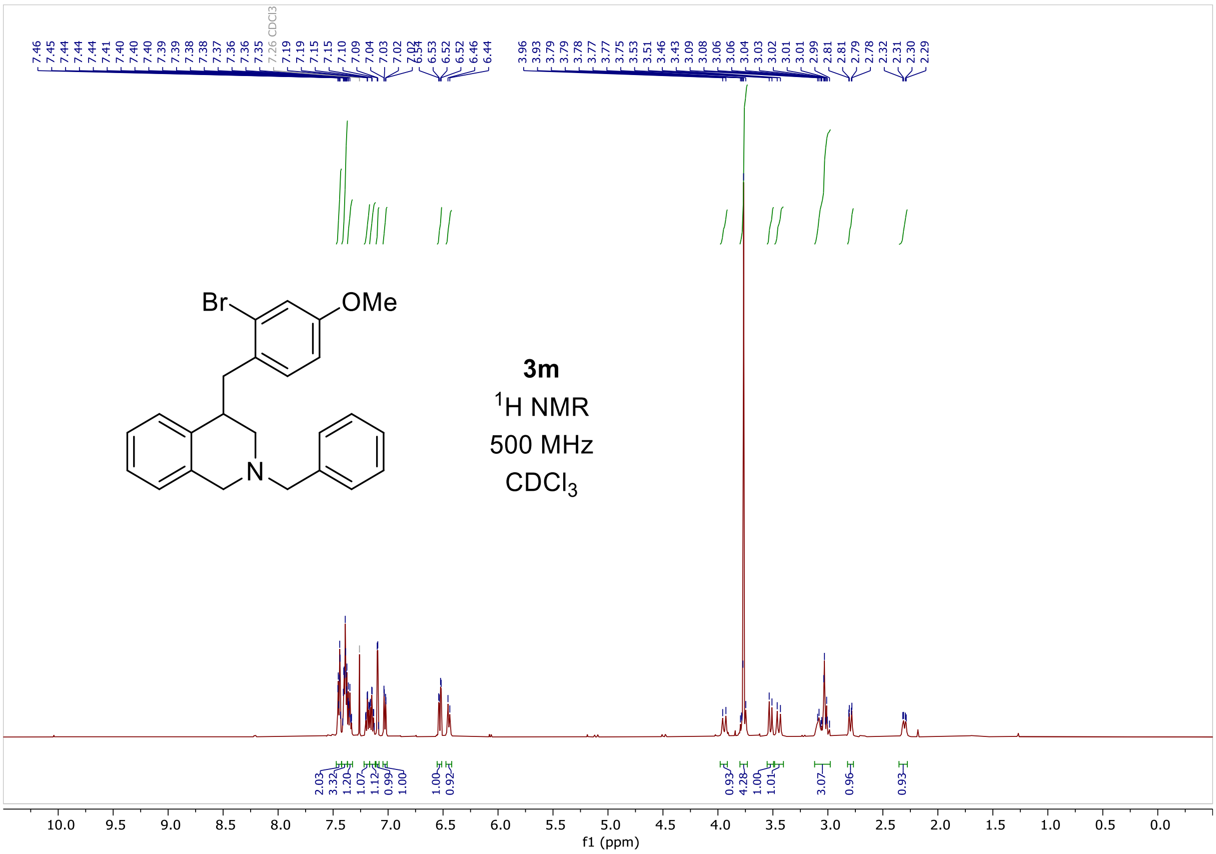
Br
OMe
N
3m
1H NMR
500 MHz
CDCl3
f1 (ppm)

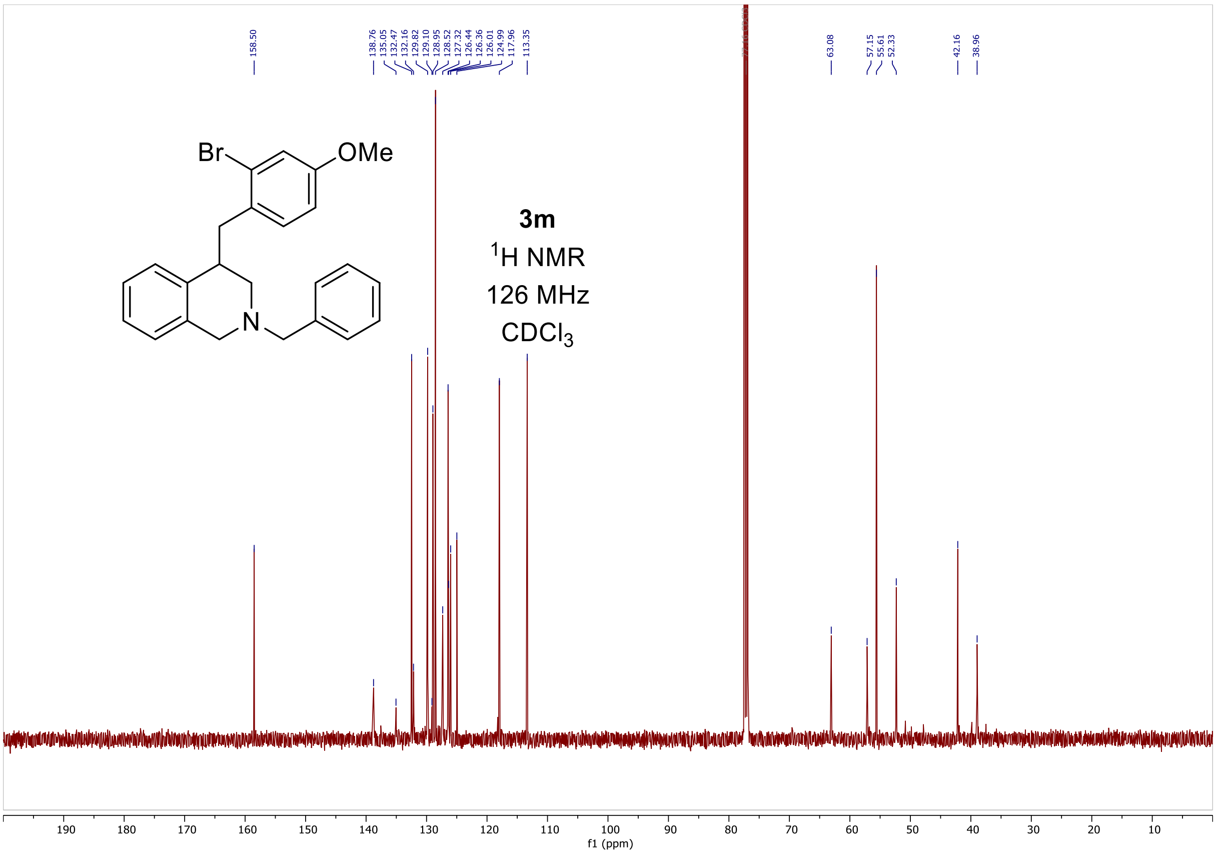
Br
OMe
N
3m
1H NMR
126 MHz
CDCl3
f1 (ppm)

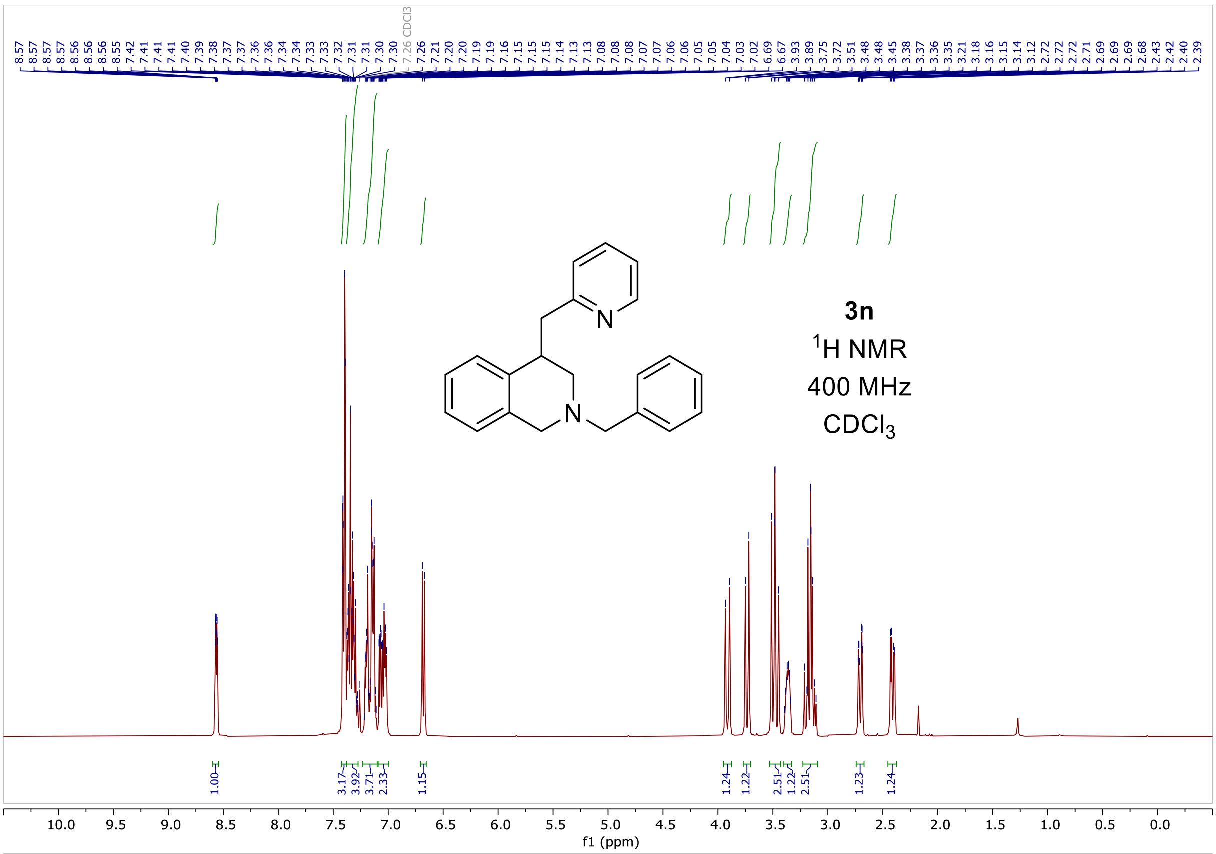
8.57 8.57 8.57 8.57 8.56 8.56 8.55 7.42 7.41 7.41 7.40 7.39 7.38 7.37 7.37 7.36 7.36 7.34 7.34 7.33 7.33 7.32 7.31 7.31 7.30 7.30 7.26 CDCl3 7.21 7.20 7.20 7.19 7.19 7.16 7.15 7.15 7.15 7.14 7.13 7.13 7.08 7.08 7.07 7.07 7.06 7.06 7.05 7.05 7.04 7.03 7.02 6.69 6.67 3.93 3.89 3.75 3.72 3.51 3.48 3.48 3.45 3.38 3.37 3.36 3.35 3.21 3.18 3.16 3.15 3.14 3.12 2.72 2.72 2.72 2.71 2.69 2.69 2.68 2.43 2.42 2.40 2.39
N
N
3n
1H NMR
400 MHz
CDCl3
1.00 3.17 3.92 3.71 2.33 1.15 1.24 1.22 2.51 1.22 2.51 1.23 1.24
10.0 9.5 9.0 8.5 8.0 7.5 7.0 6.5 6.0 5.5 5.0 4.5 4.0 3.5 3.0 2.5 2.0 1.5 1.0 0.5 0.0
f1 (ppm)

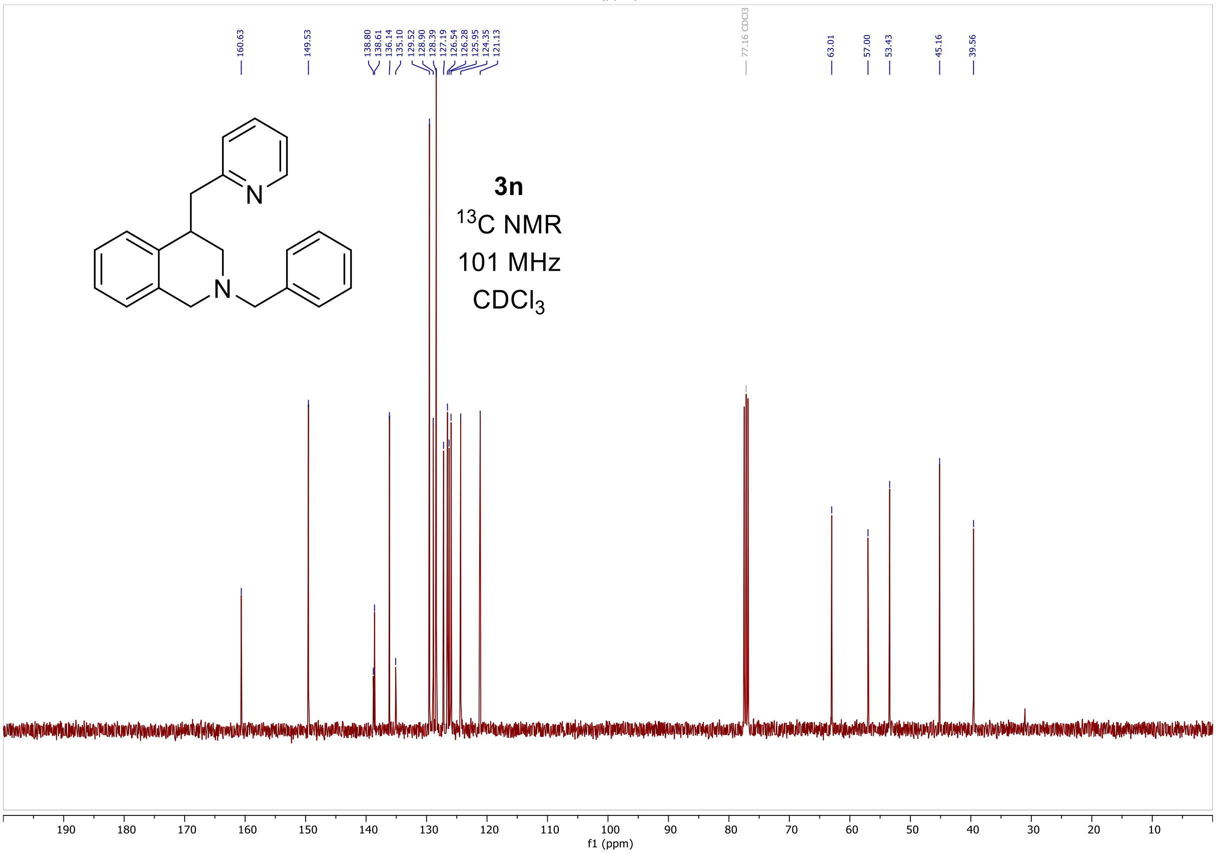
160.63 149.53 138.80 138.61 136.14 135.10 129.52 128.90 128.39 127.19 126.54 126.28 125.95 124.35 121.13 77.16 CDCl3 63.01 57.00 53.43 45.16 39.56
N
N
3n
13C NMR
101 MHz
CDCl3
190 180 170 160 150 140 130 120 110 100 90 80 70 60 50 40 30 20 10
f1 (ppm)

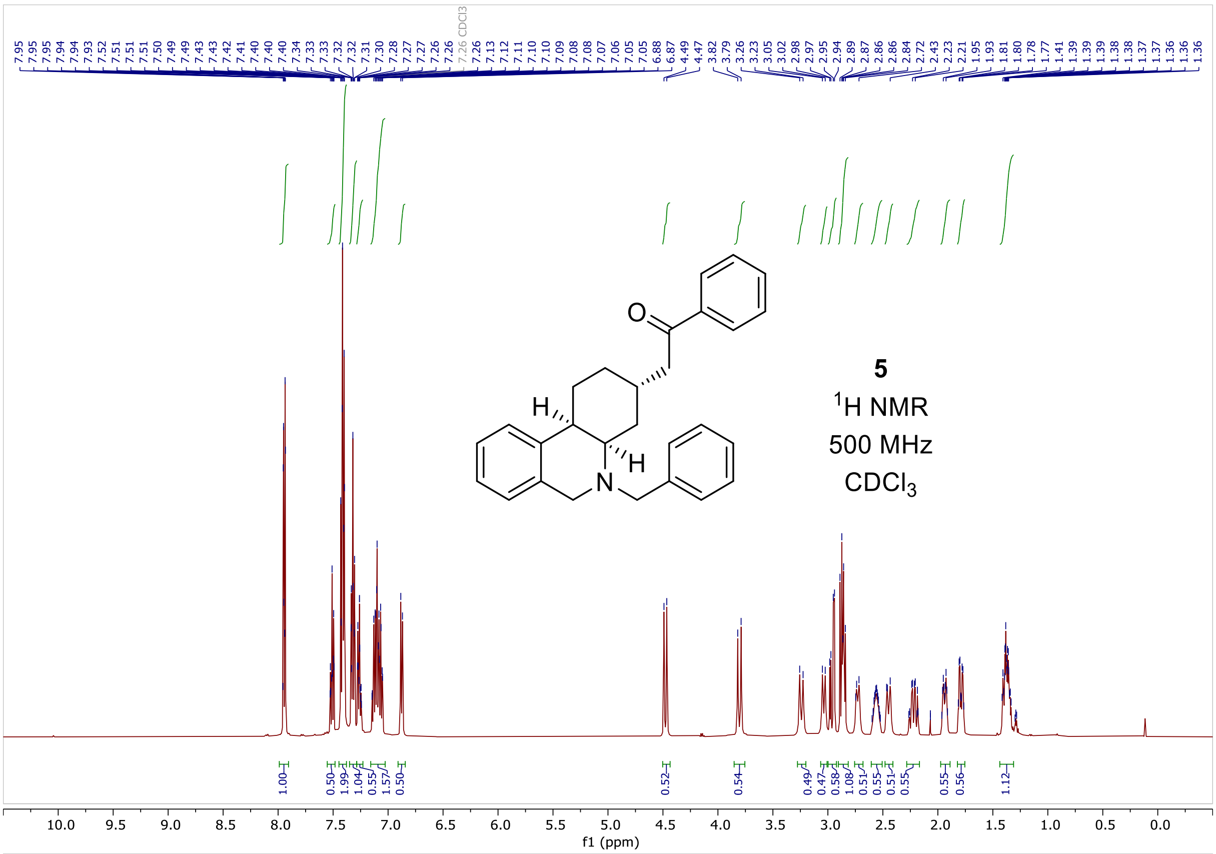
5
^{1}H NMR
500 MHz
CDCl$_3$
f1 (ppm)

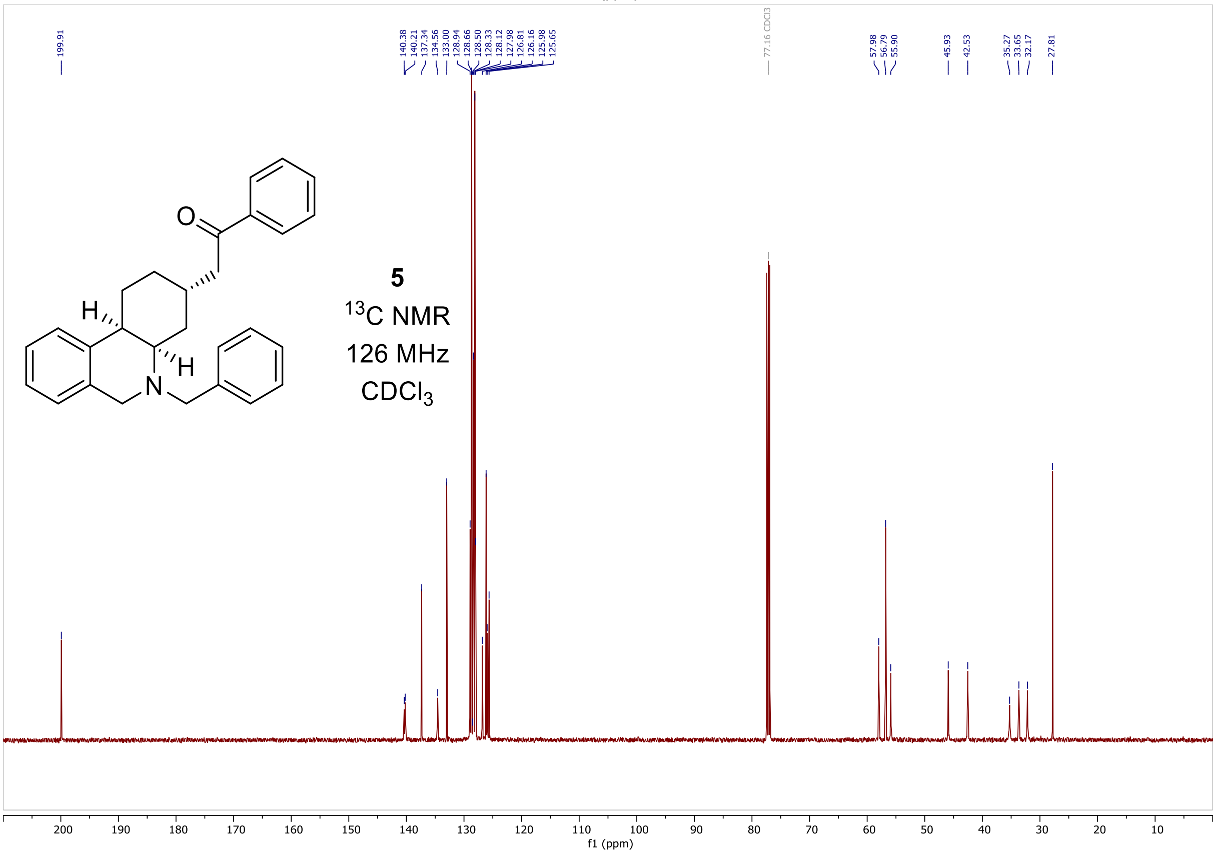
5
^{13}C NMR
126 MHz
CDCl$_3$
f1 (ppm)

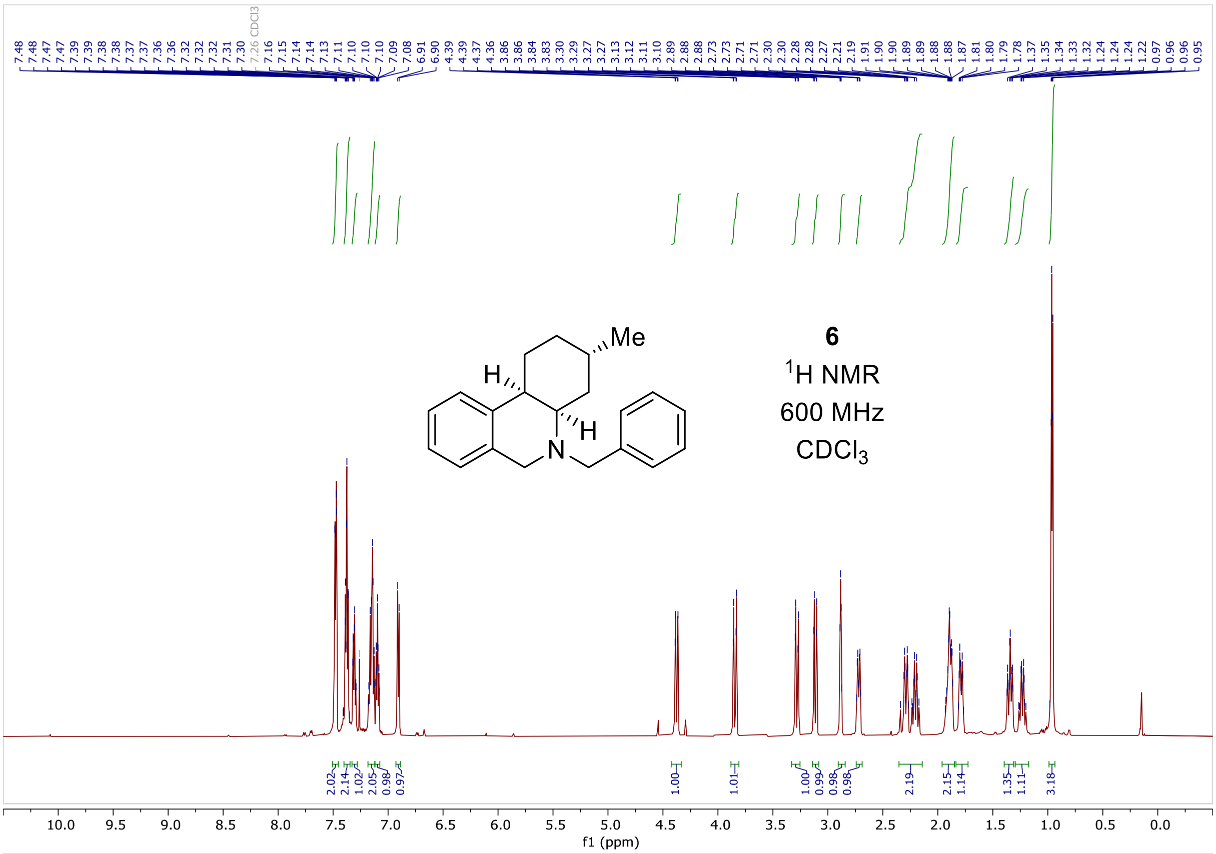
6
1H NMR
600 MHz
CDCl3
f1 (ppm)

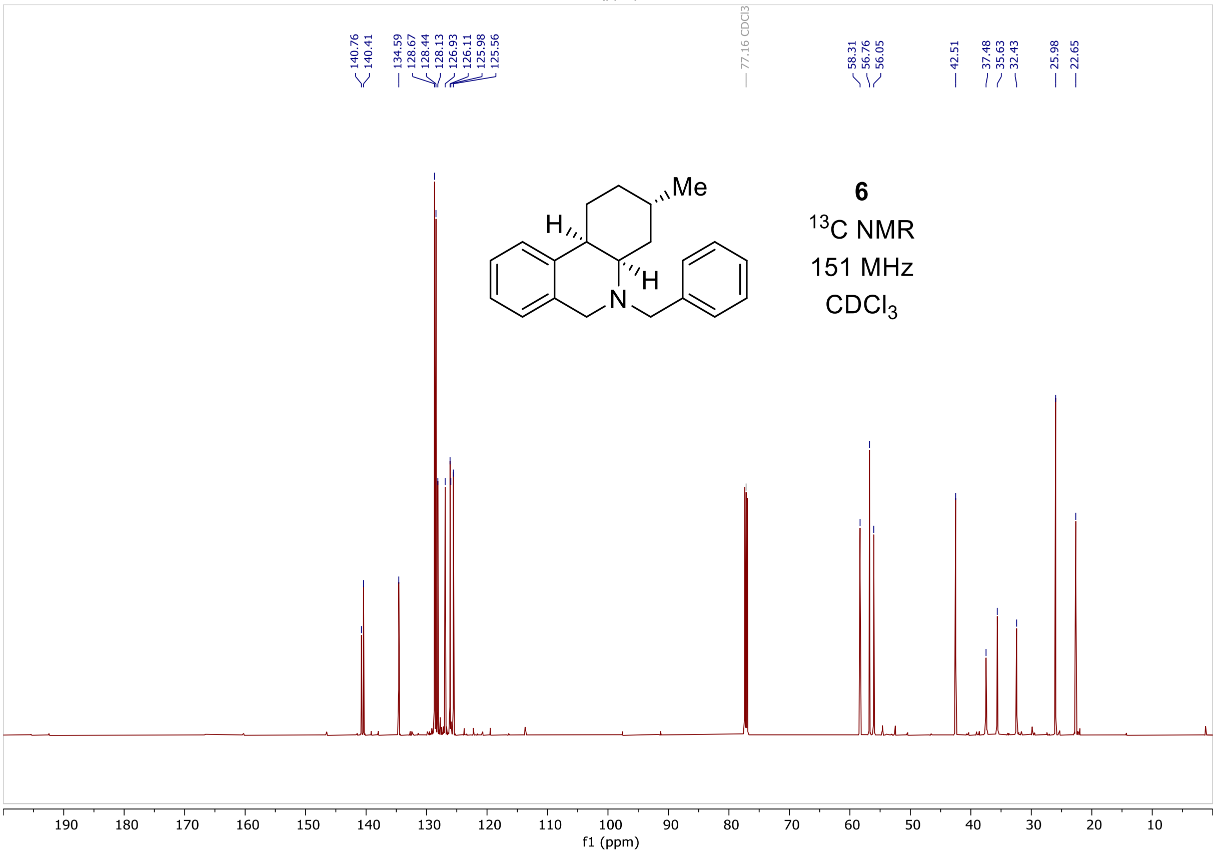
6
13C NMR
151 MHz
CDCl3
140.76
140.41
134.59
128.67
128.44
128.13
126.93
126.11
125.98
125.56
77.16 CDCl3
58.31
56.76
56.05
42.51
37.48
35.63
32.43
25.98
22.65
f1 (ppm)

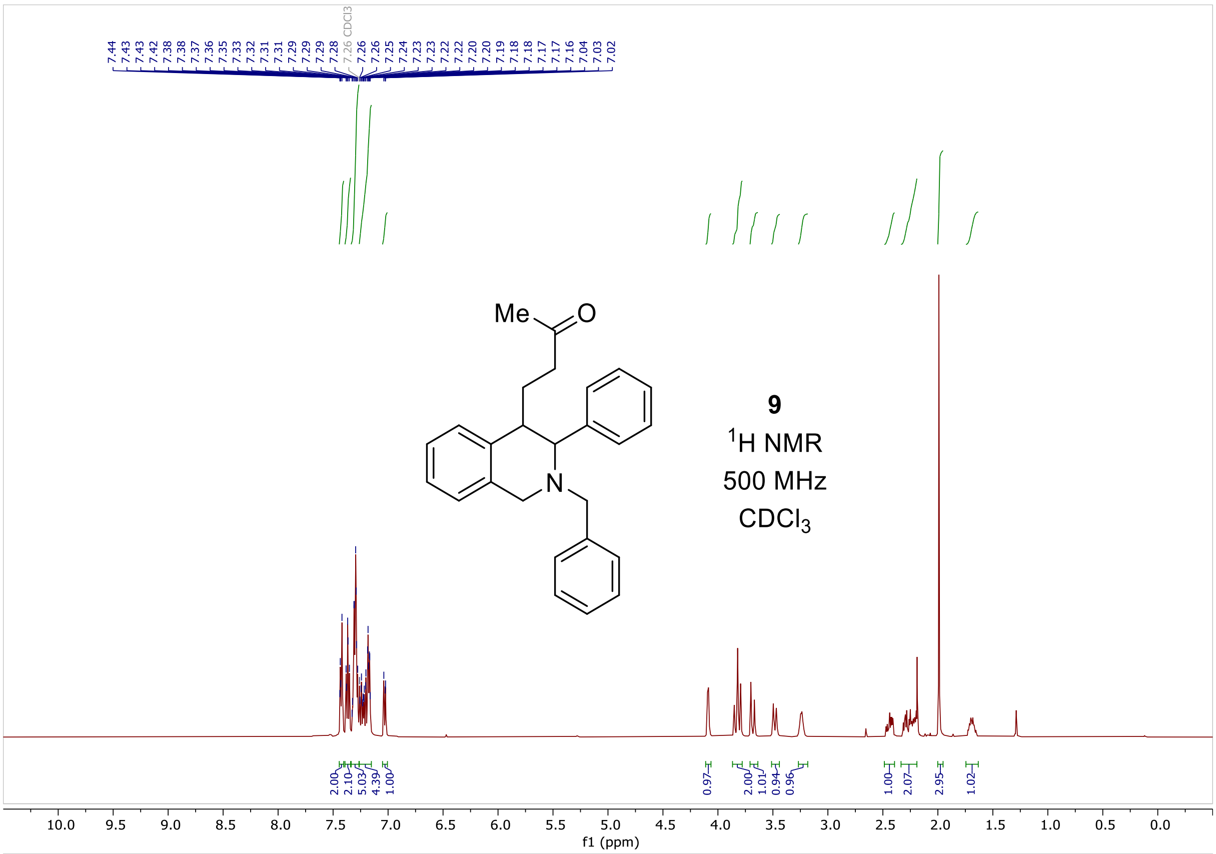
7.44 7.43 7.43 7.42 7.38 7.38 7.37 7.36 7.35 7.33 7.32 7.31 7.31 7.29 7.29 7.29 7.28 7.26 CDCl3 7.26 7.26 7.25 7.24 7.23 7.23 7.22 7.22 7.20 7.19 7.18 7.18 7.17 7.17 7.16 7.04 7.03 7.02
Me
O
N
9
¹H NMR
500 MHz
CDCl₃
2.00 2.10 5.03 4.39 1.00 0.97 2.00 1.01 0.94 0.96 1.00 2.07 2.95 1.02
f1 (ppm)

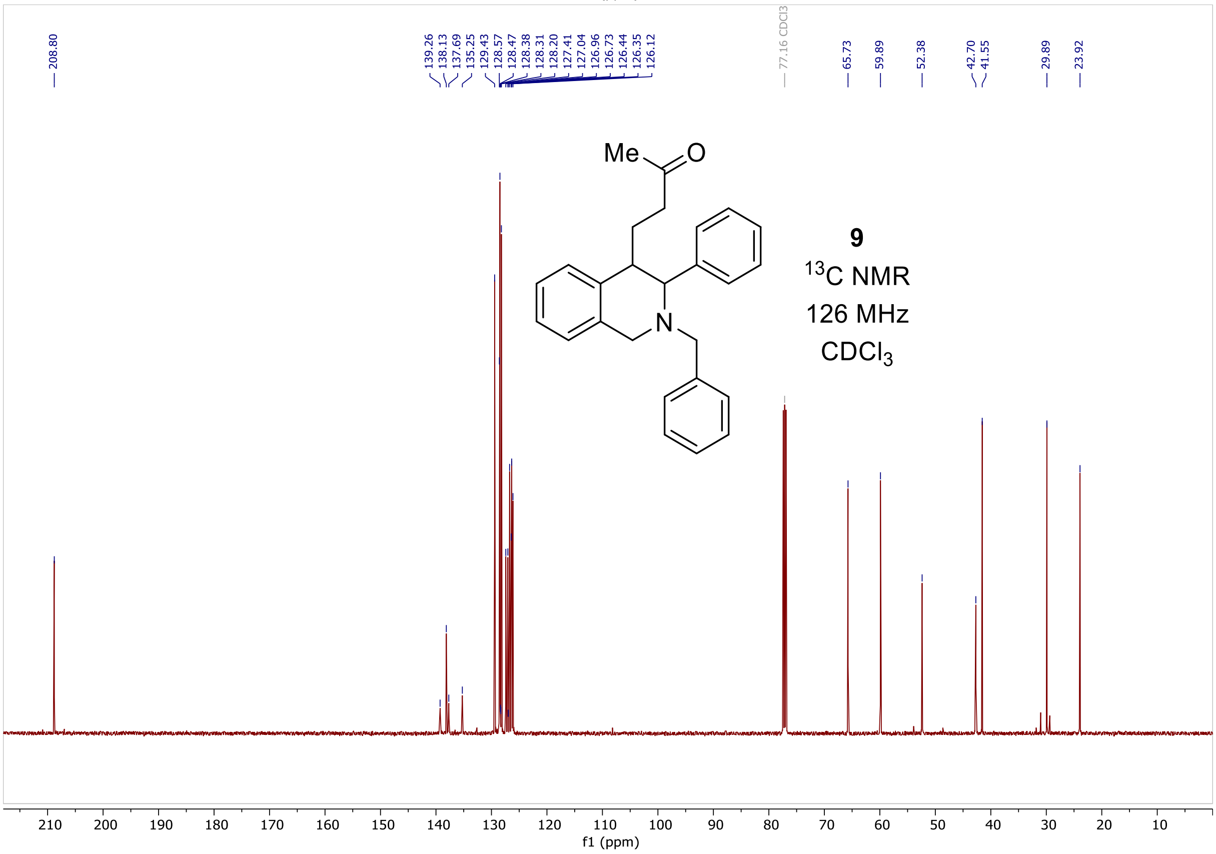
208.80
139.26 138.13 137.69 135.25 129.43 128.57 128.47 128.38 128.31 128.20 127.41 127.04 126.96 126.73 126.44 126.35 126.12
77.16 CDCl3
65.73
59.89
52.38
42.70 41.55
29.89
23.92
Me
O
N
9
¹³C NMR
126 MHz
CDCl₃
f1 (ppm)

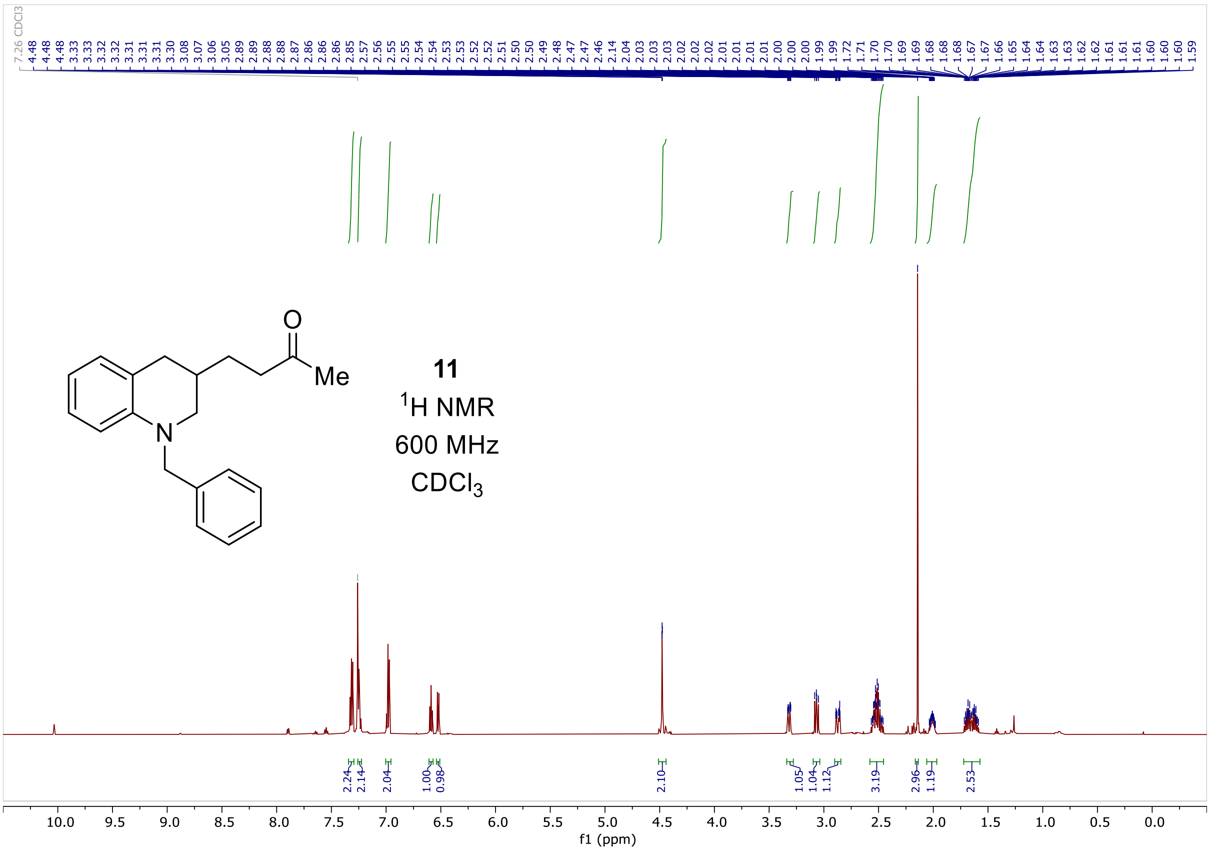
11
1H NMR
600 MHz
CDCl3
f1 (ppm)

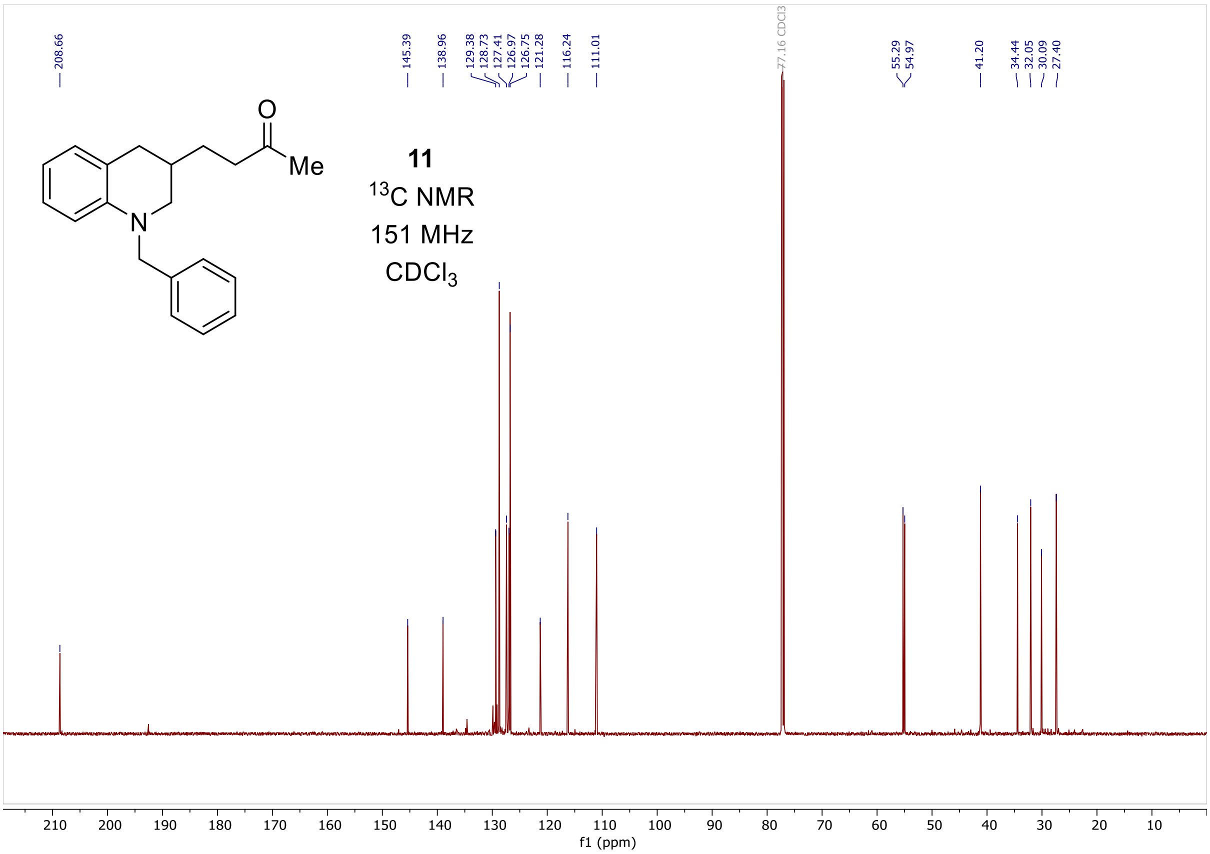
11
13C NMR
151 MHz
CDCl3
208.66
145.39
138.96
129.38
128.73
127.41
126.97
126.75
121.28
116.24
111.01
77.16 CDCl3
55.29
54.97
41.20
34.44
32.05
30.09
27.40
f1 (ppm)

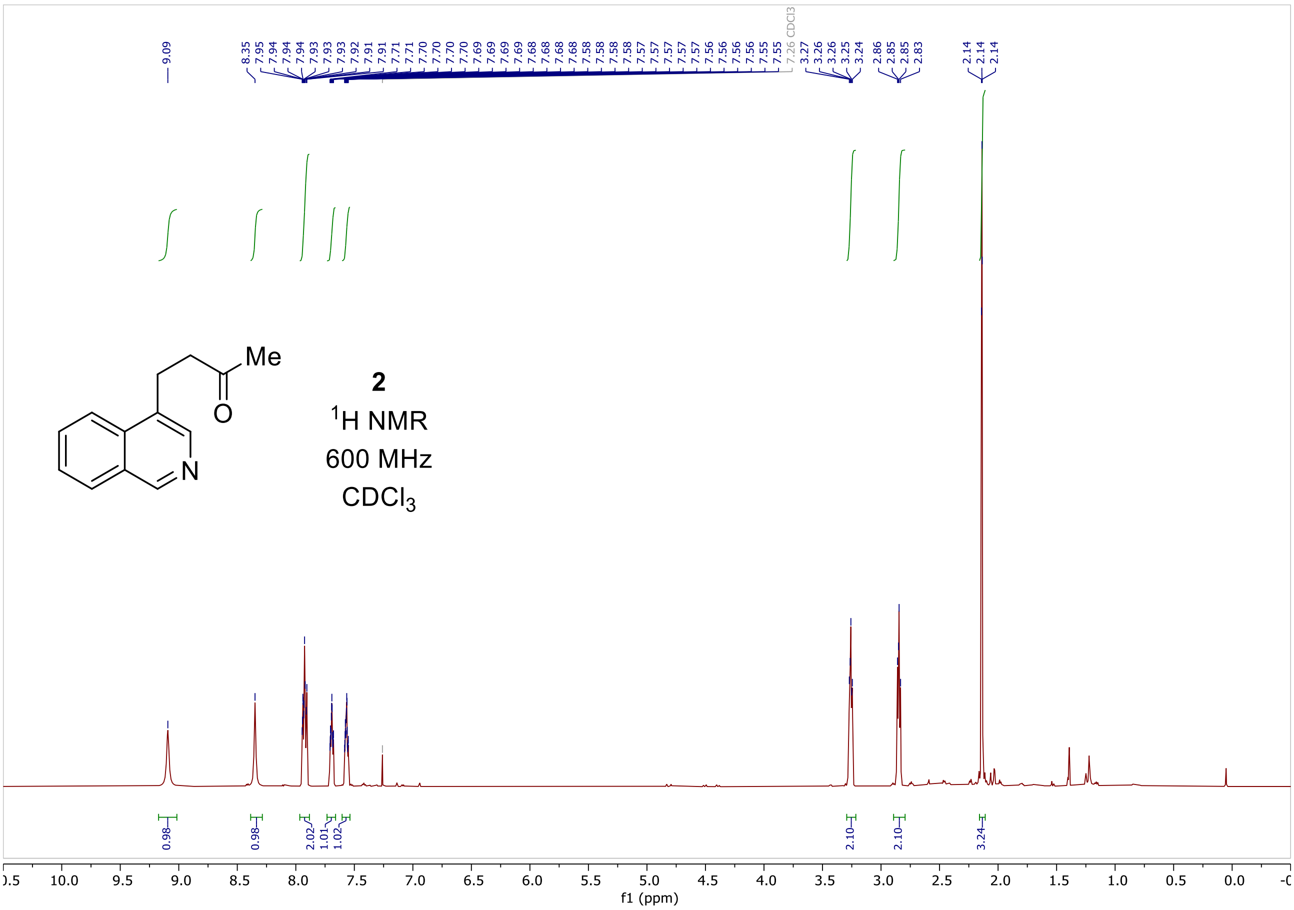
9.09
8.35 7.95 7.94 7.94 7.93 7.93 7.92 7.91 7.91 7.71 7.71 7.70 7.70 7.70 7.69 7.69 7.69 7.68 7.68 7.68 7.58 7.58 7.58 7.57 7.57 7.57 7.57 7.56 7.56 7.56 7.55 7.55
7.26 CDCl3
3.27 3.26 3.26 3.25 3.24
2.86 2.85 2.85 2.83
2.14 2.14 2.14
Me
O
N
2
1H NMR
600 MHz
CDCl3
0.98 0.98 2.02 1.01 1.02 2.10 2.10 3.24
f1 (ppm)

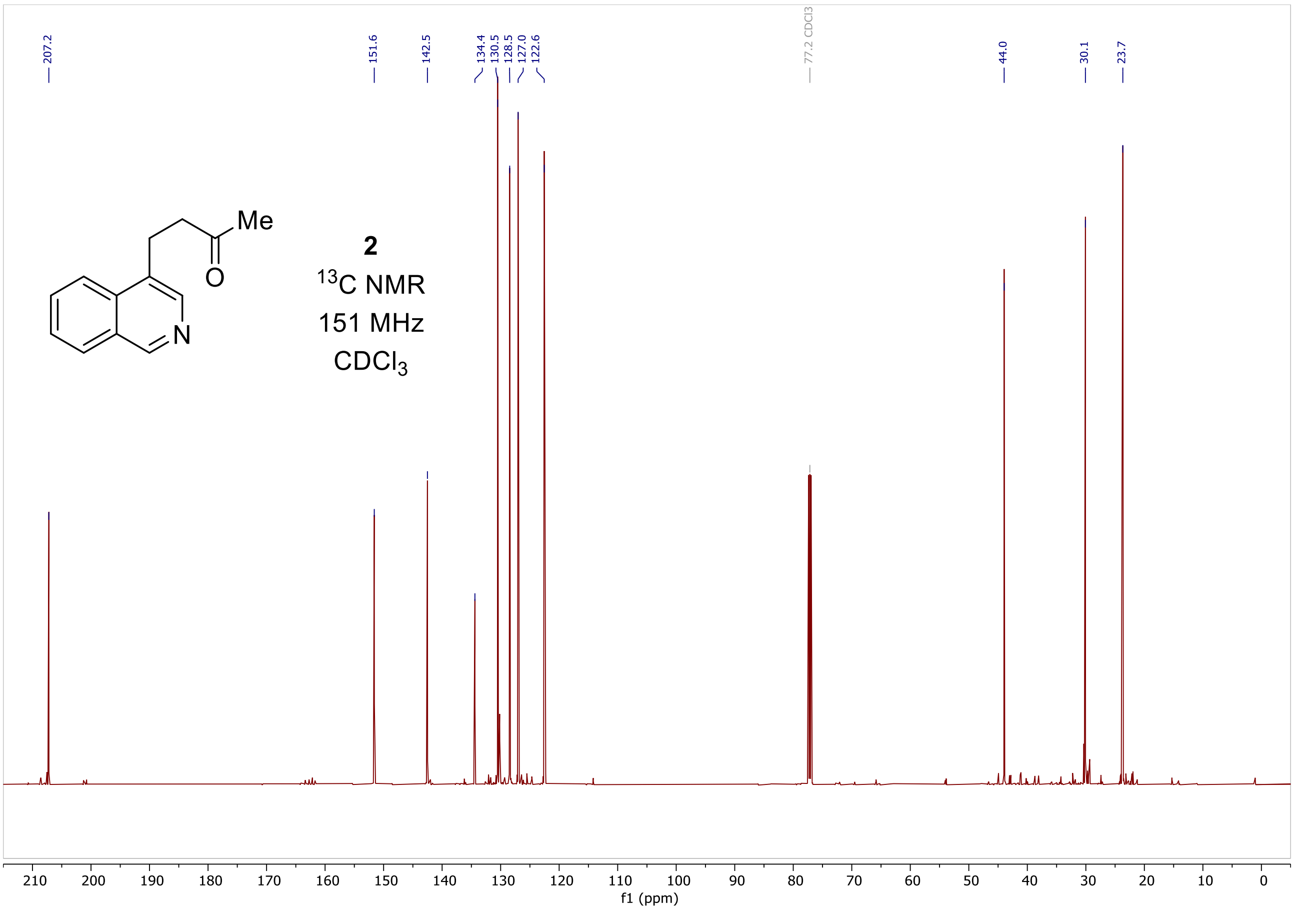
207.2
151.6
142.5
134.4 130.5 128.5 127.0 122.6
77.2 CDCl3
44.0
30.1
23.7
Me
O
N
2
13C NMR
151 MHz
CDCl3
f1 (ppm)

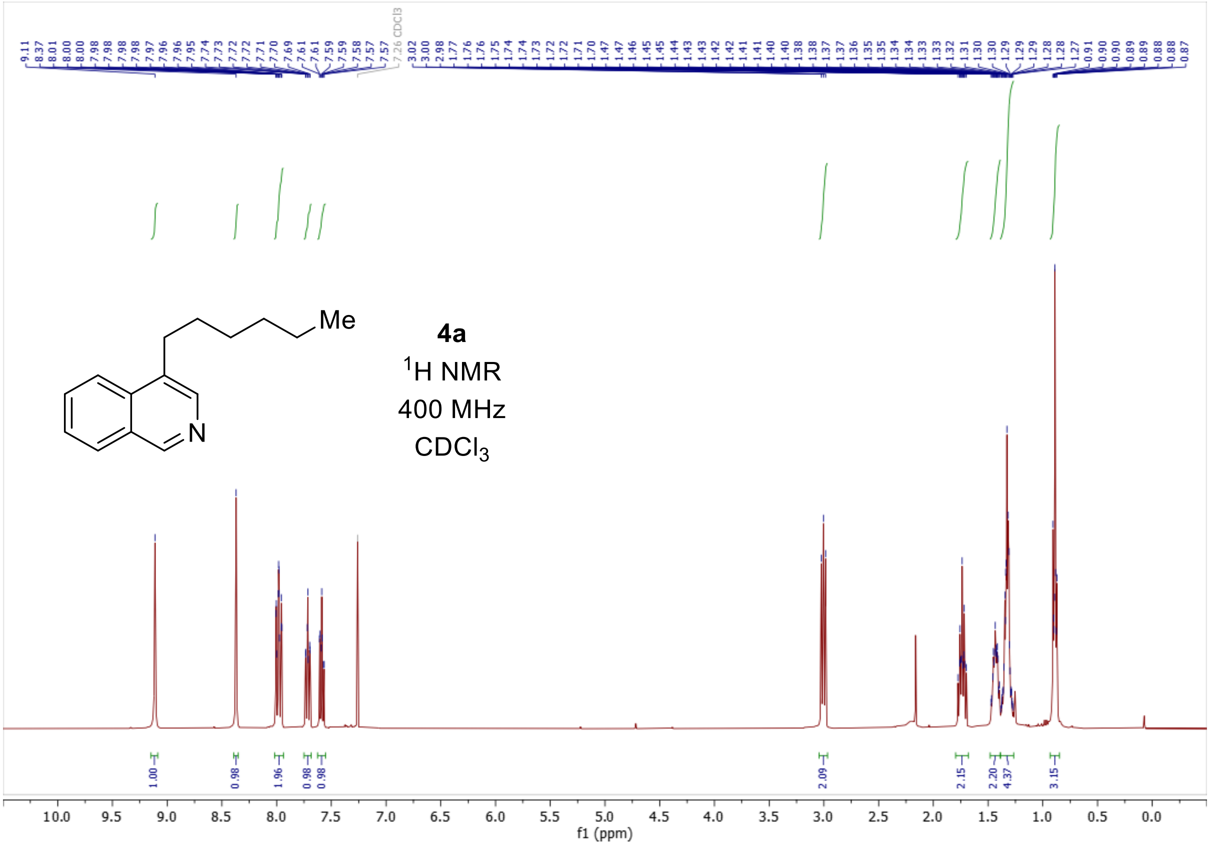
4a
1H NMR
400 MHz
CDCl3
Me
N
f1 (ppm)

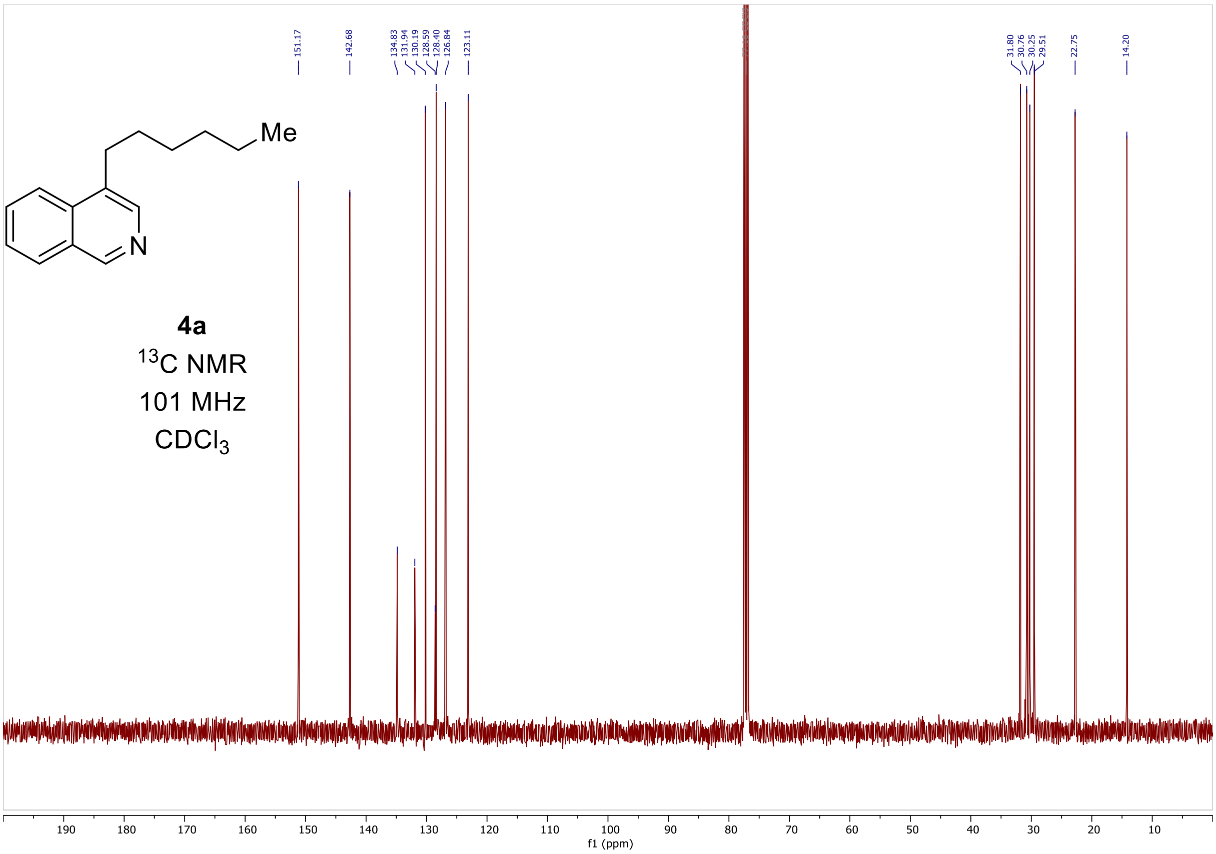
4a
13C NMR
101 MHz
CDCl3
Me
N
f1 (ppm)

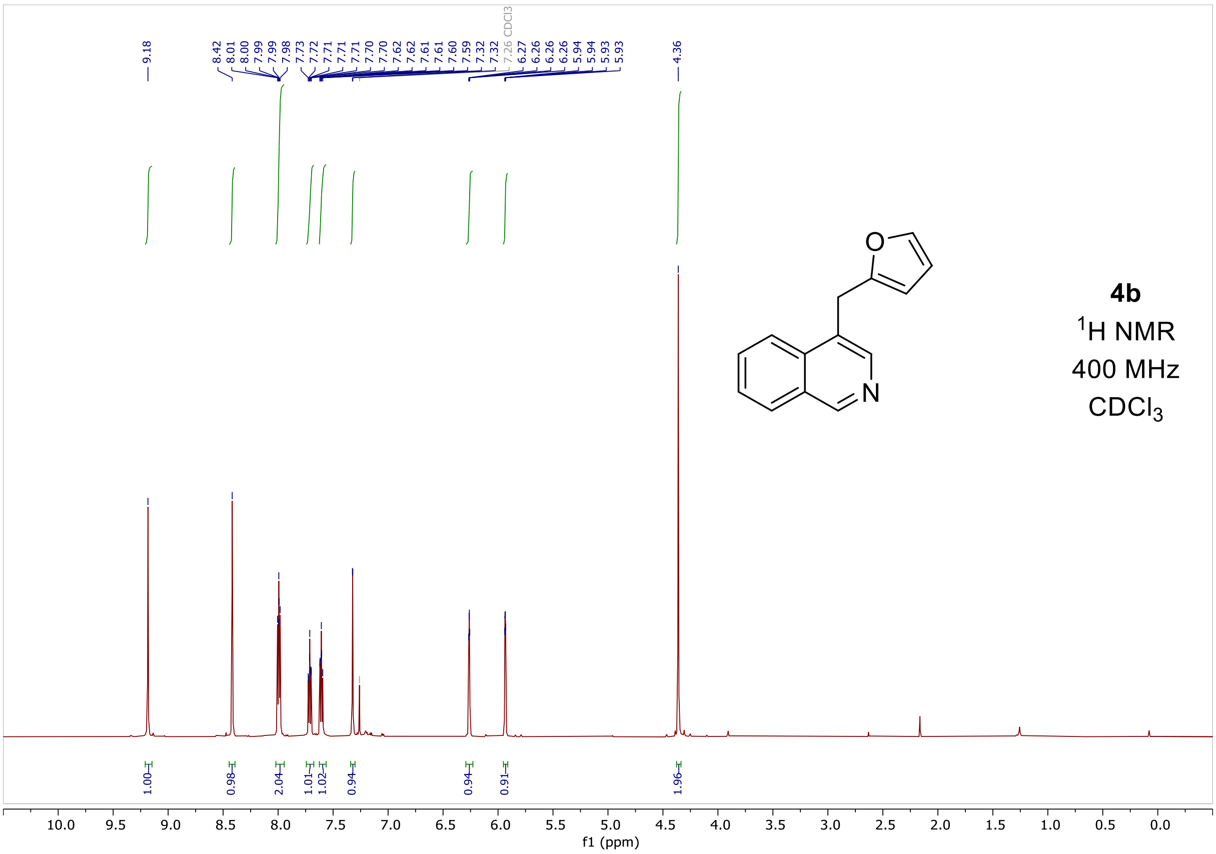
9.18
8.42
8.01
8.00
7.99
7.99
7.98
7.73
7.72
7.71
7.71
7.71
7.70
7.70
7.62
7.62
7.61
7.61
7.60
7.59
7.32
7.32
7.26 CDCl3
6.27
6.26
6.26
6.26
5.94
5.94
5.93
5.93
4.36
O
N
4b
1H NMR
400 MHz
CDCl3
1.00
0.98
2.04
1.01
1.02
0.94
0.94
0.91
1.96
10.0 9.5 9.0 8.5 8.0 7.5 7.0 6.5 6.0 5.5 5.0 4.5 4.0 3.5 3.0 2.5 2.0 1.5 1.0 0.5 0.0
f1 (ppm)

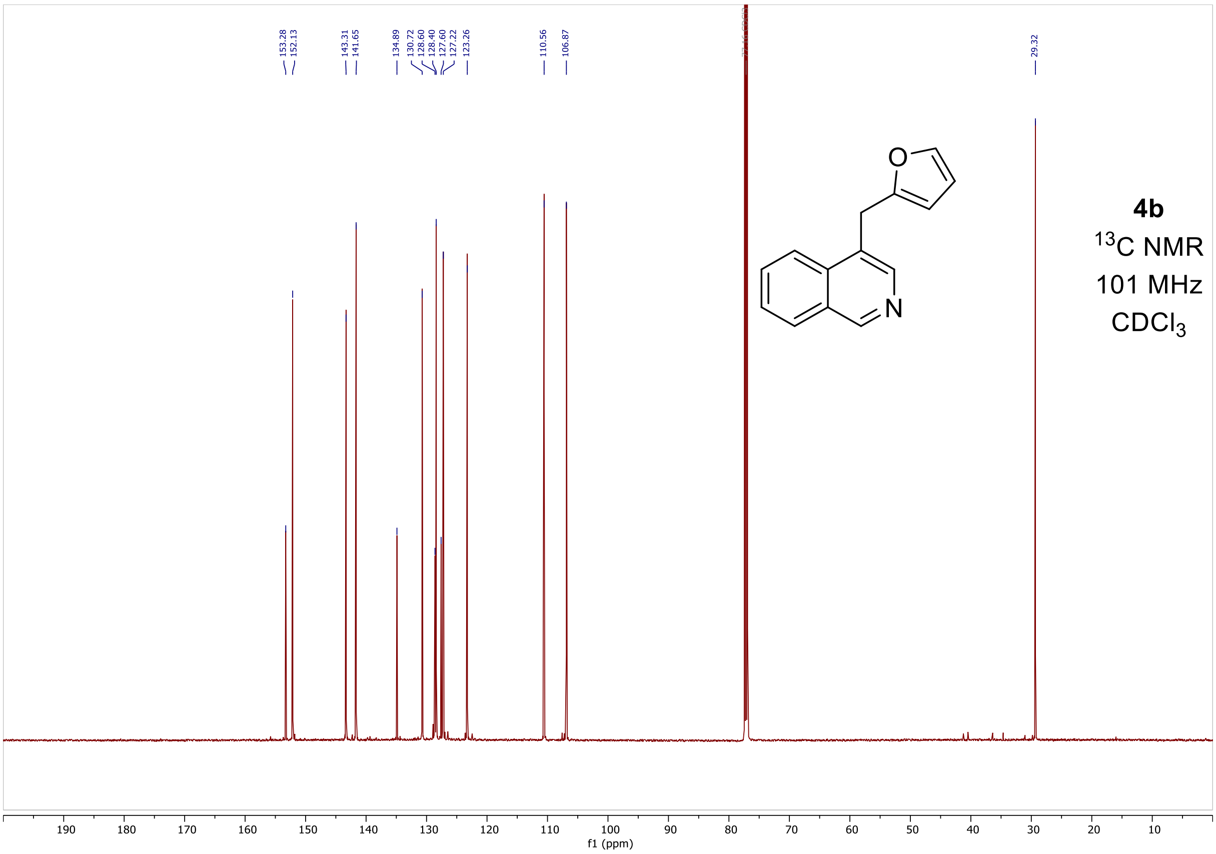
153.28
152.13
143.31
141.65
134.89
130.72
128.60
128.40
127.60
127.22
123.26
110.56
106.87
29.32
O
N
4b
13C NMR
101 MHz
CDCl3
190 180 170 160 150 140 130 120 110 100 90 80 70 60 50 40 30 20 10
f1 (ppm)

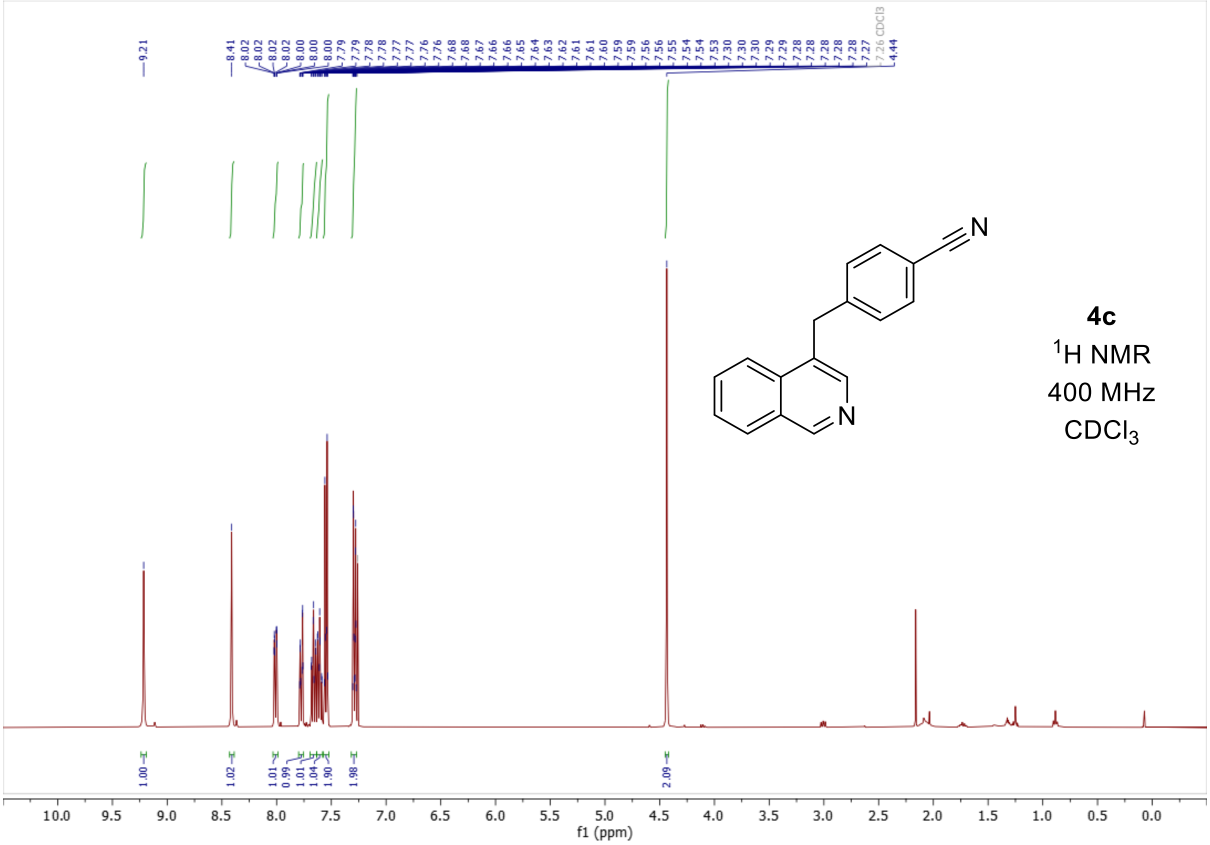
4c
1H NMR
400 MHz
CDCl3
N
N
f1 (ppm)

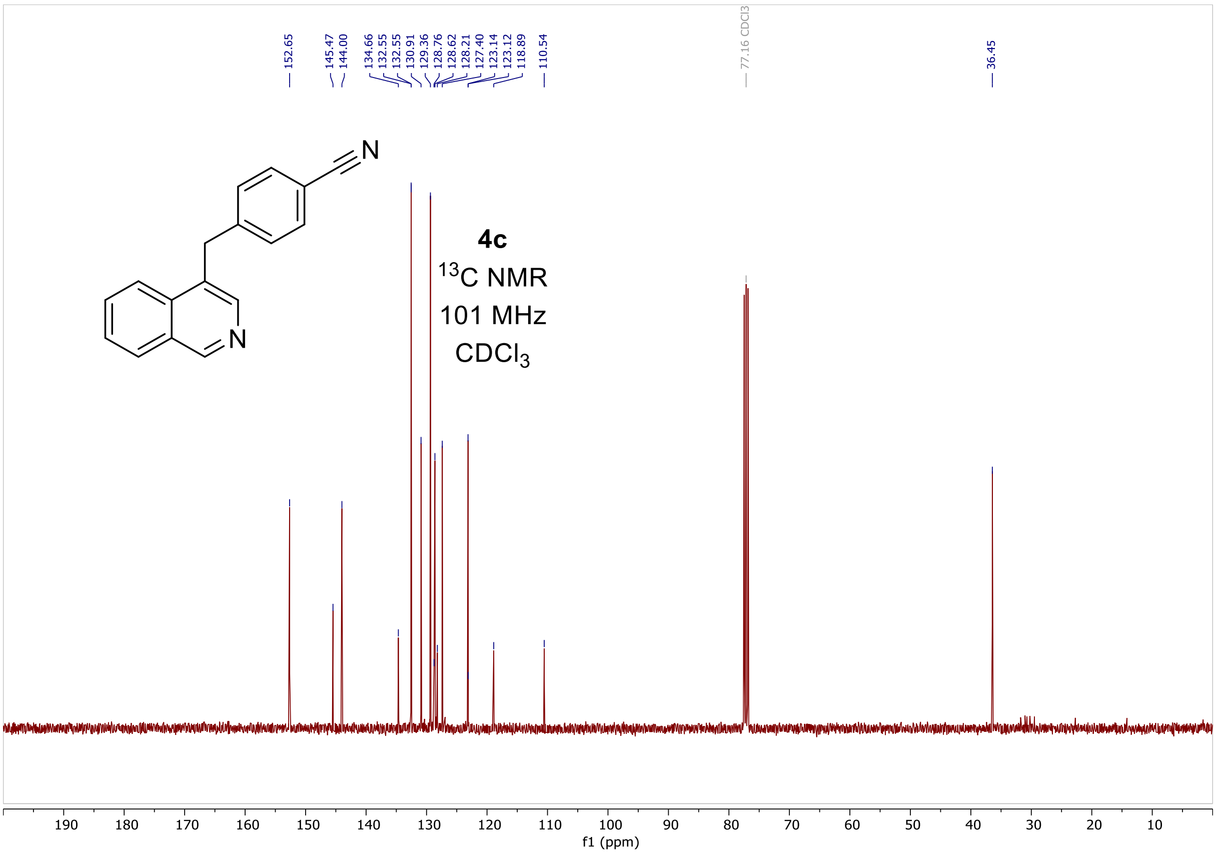
4c
13C NMR
101 MHz
CDCl3
N
N
f1 (ppm)

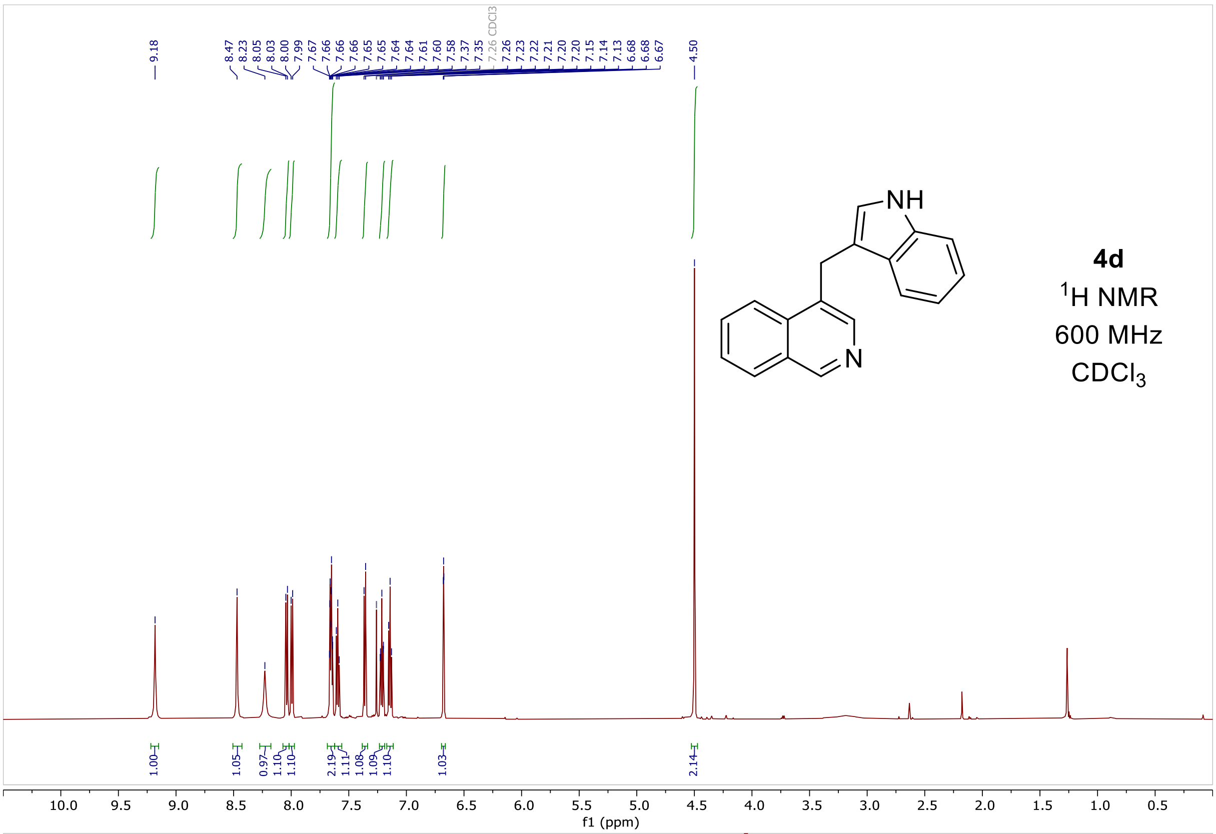
4d
^{1}H NMR
600 MHz
$CDCl_3$
NH
N
f1 (ppm)

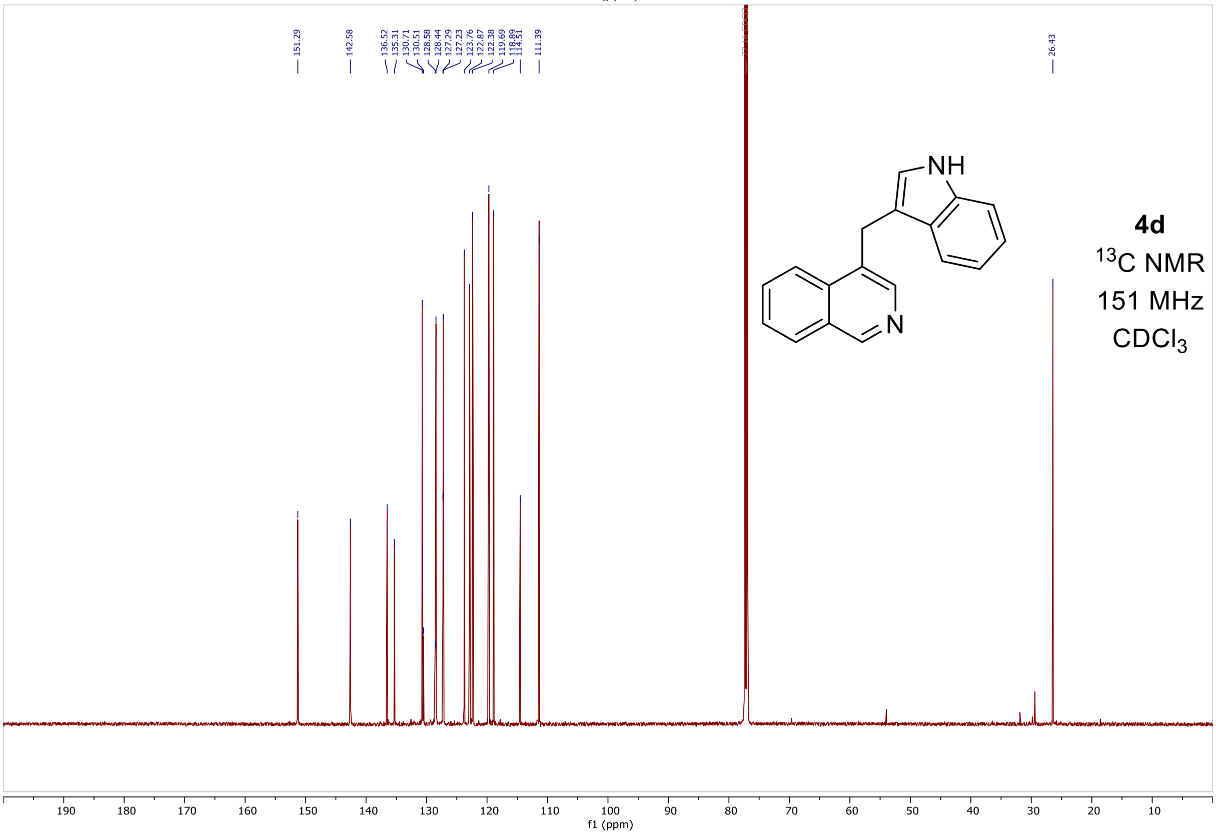
4d
^{13}C NMR
151 MHz
$CDCl_3$
NH
N
f1 (ppm)

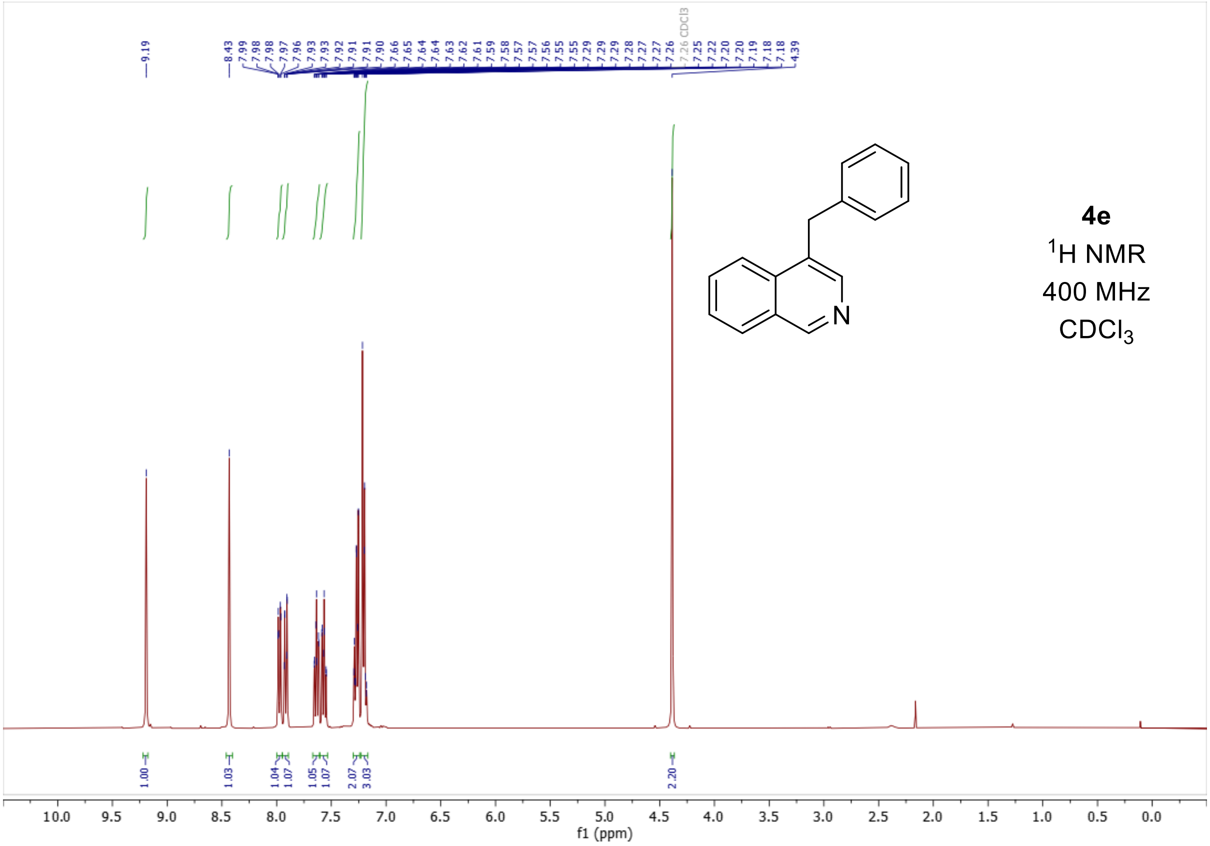
4e
^{1}H NMR
400 MHz
$CDCl_3$
N
f1 (ppm)

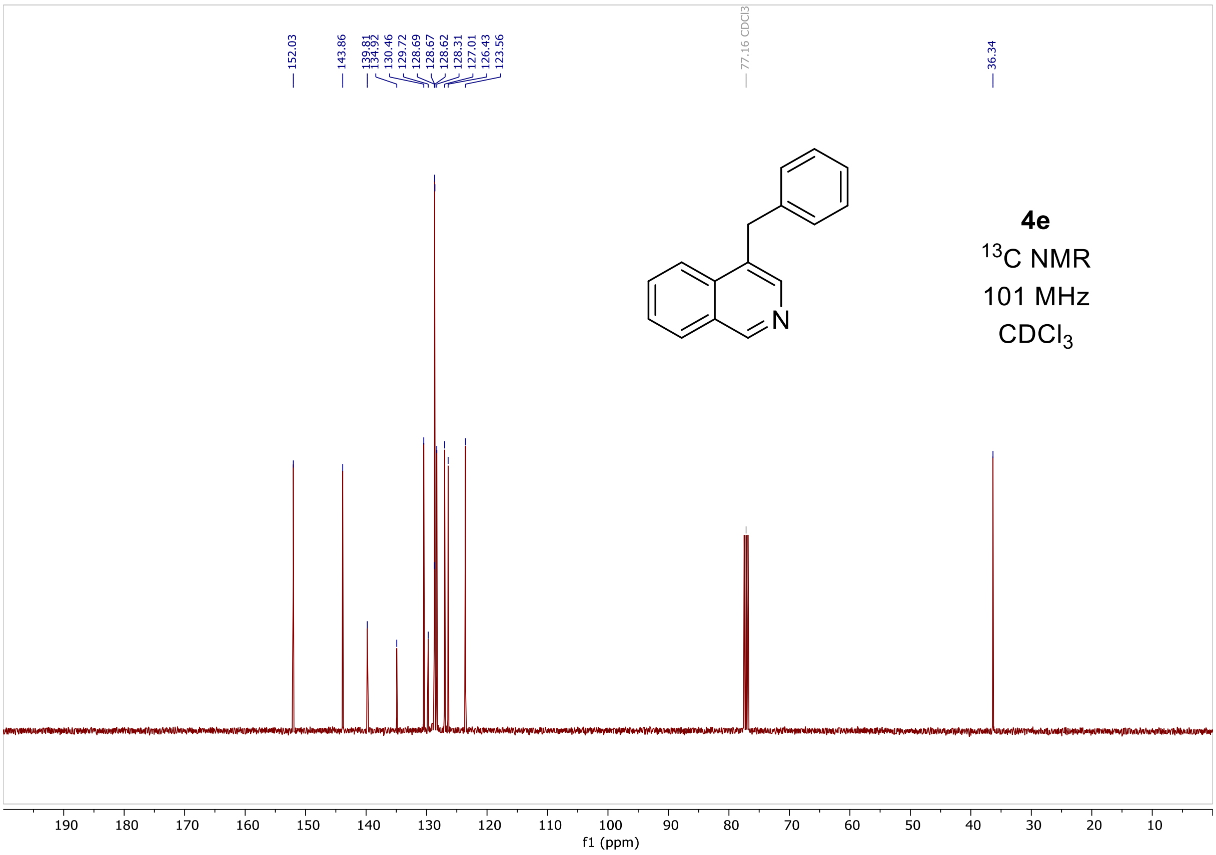
4e
^{13}C NMR
101 MHz
$CDCl_3$
N
f1 (ppm)

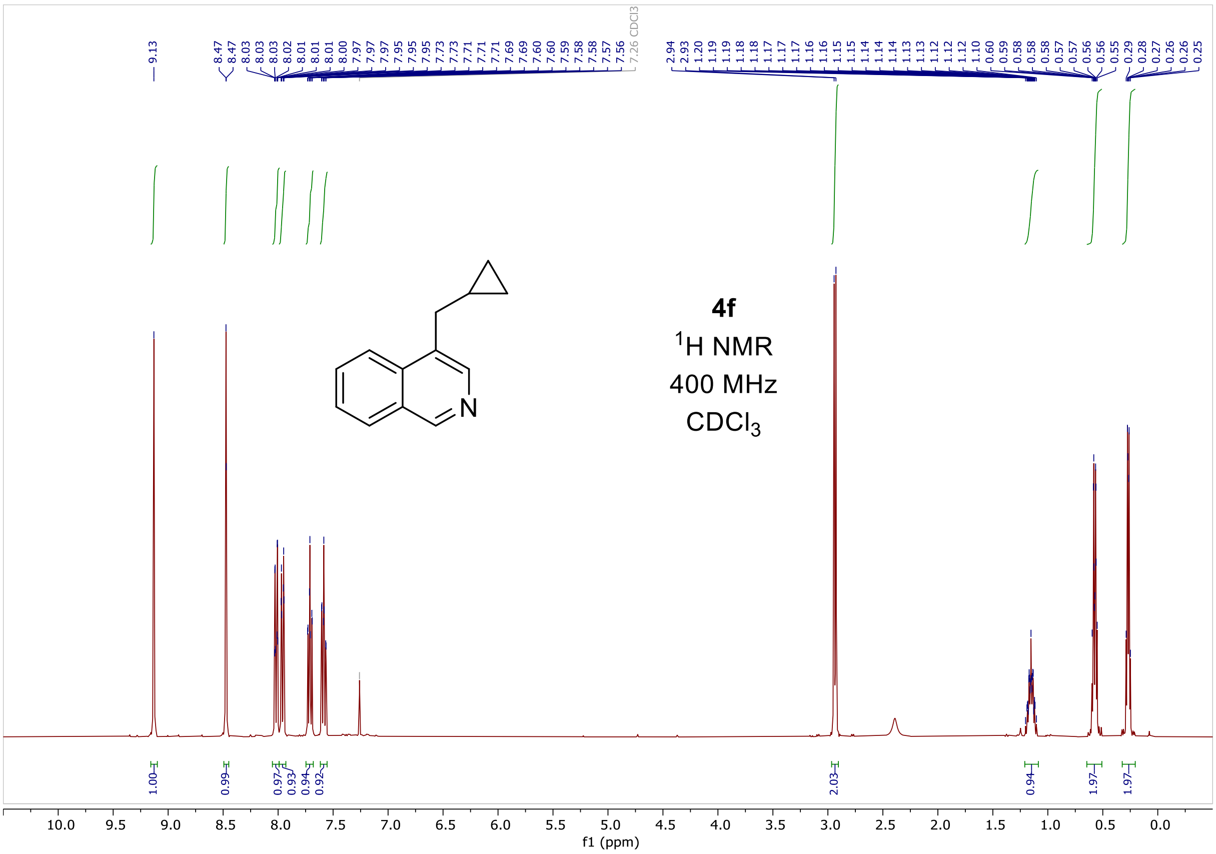
4f
1H NMR
400 MHz
CDCl3
f1 (ppm)

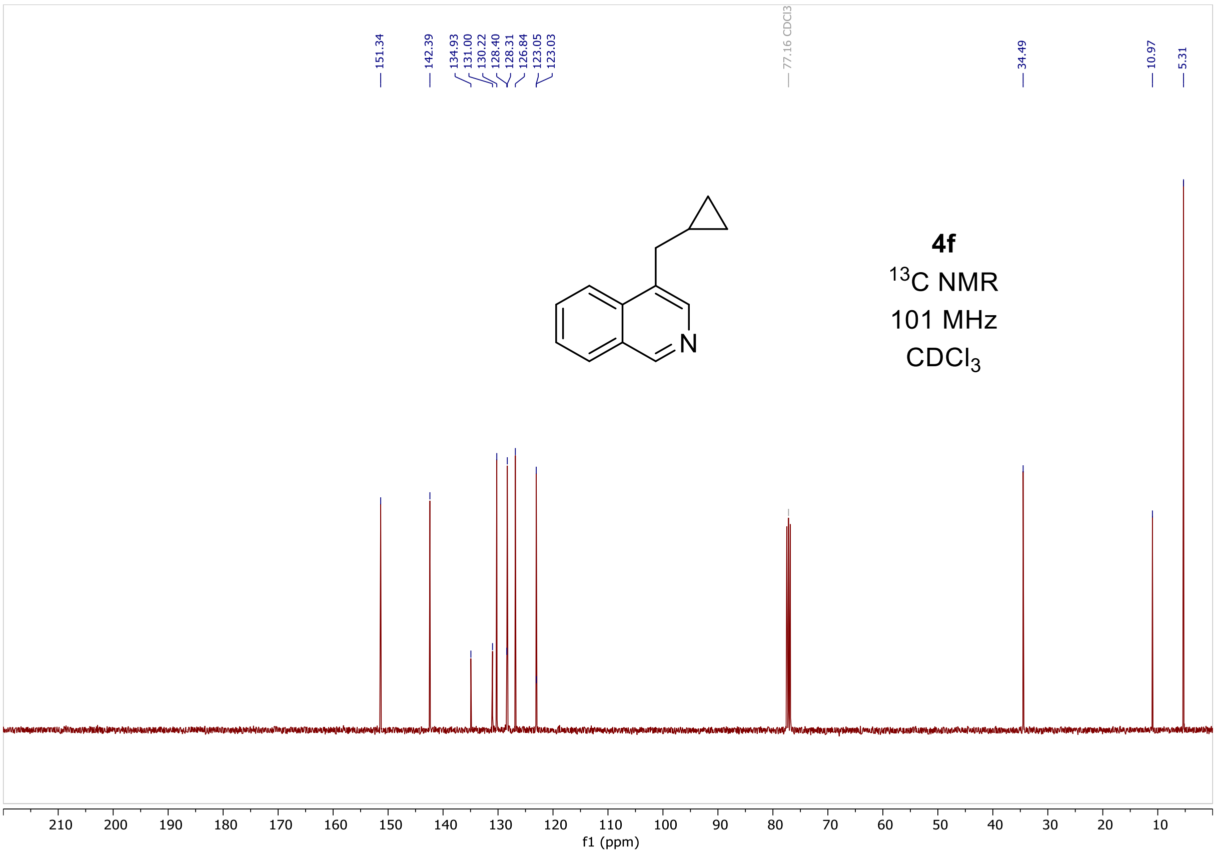
4f
13C NMR
101 MHz
CDCl3
f1 (ppm)

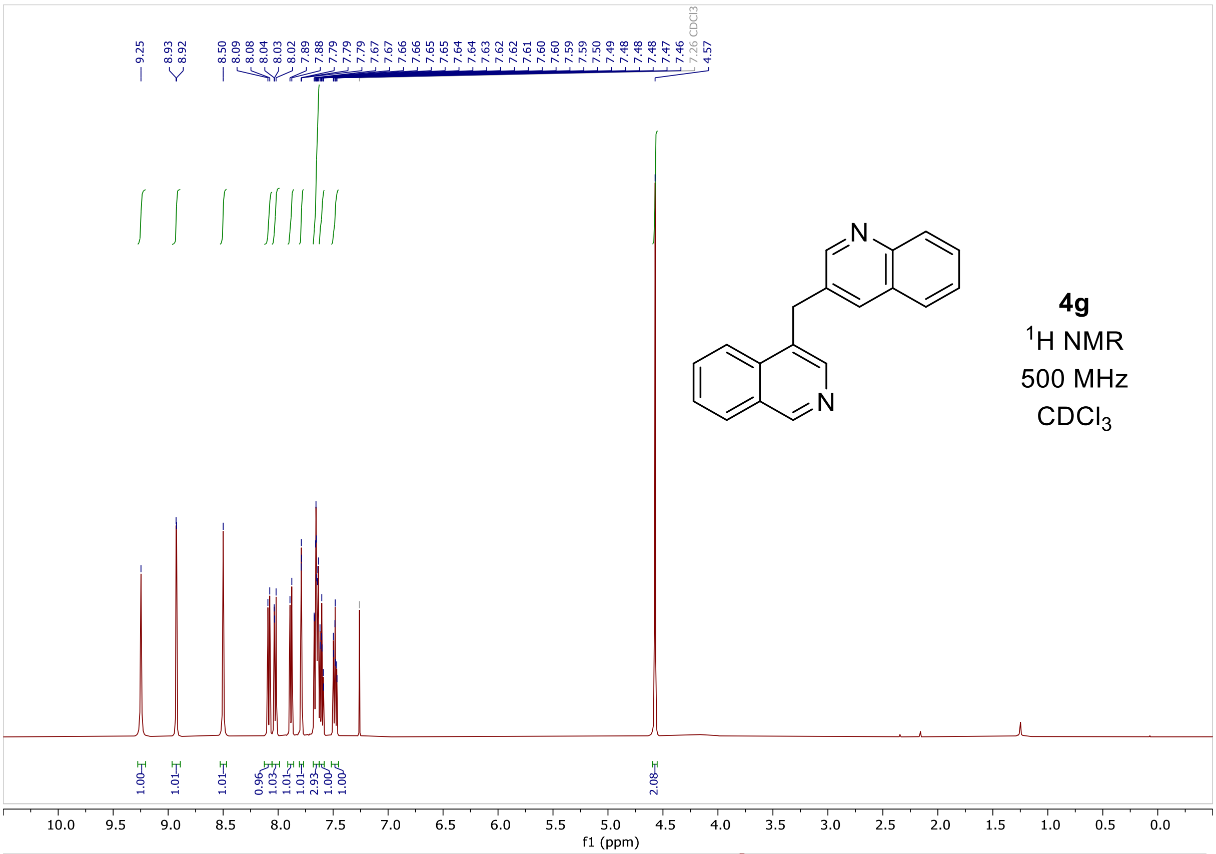
9.25
8.93
8.92
8.50
8.09
8.08
8.04
8.03
8.02
7.89
7.88
7.79
7.79
7.79
7.67
7.67
7.66
7.65
7.65
7.64
7.64
7.63
7.62
7.62
7.61
7.60
7.60
7.59
7.59
7.50
7.49
7.48
7.48
7.47
7.46
7.26 CDCl3
4.57
1.00
1.01
1.01
0.96
1.03
1.01
1.01
2.93
1.00
1.00
2.08
4g
1H NMR
500 MHz
CDCl3
10.0
9.5
9.0
8.5
8.0
7.5
7.0
6.5
6.0
5.5
5.0
4.5
4.0
3.5
3.0
2.5
2.0
1.5
1.0
0.5
0.0
f1 (ppm)

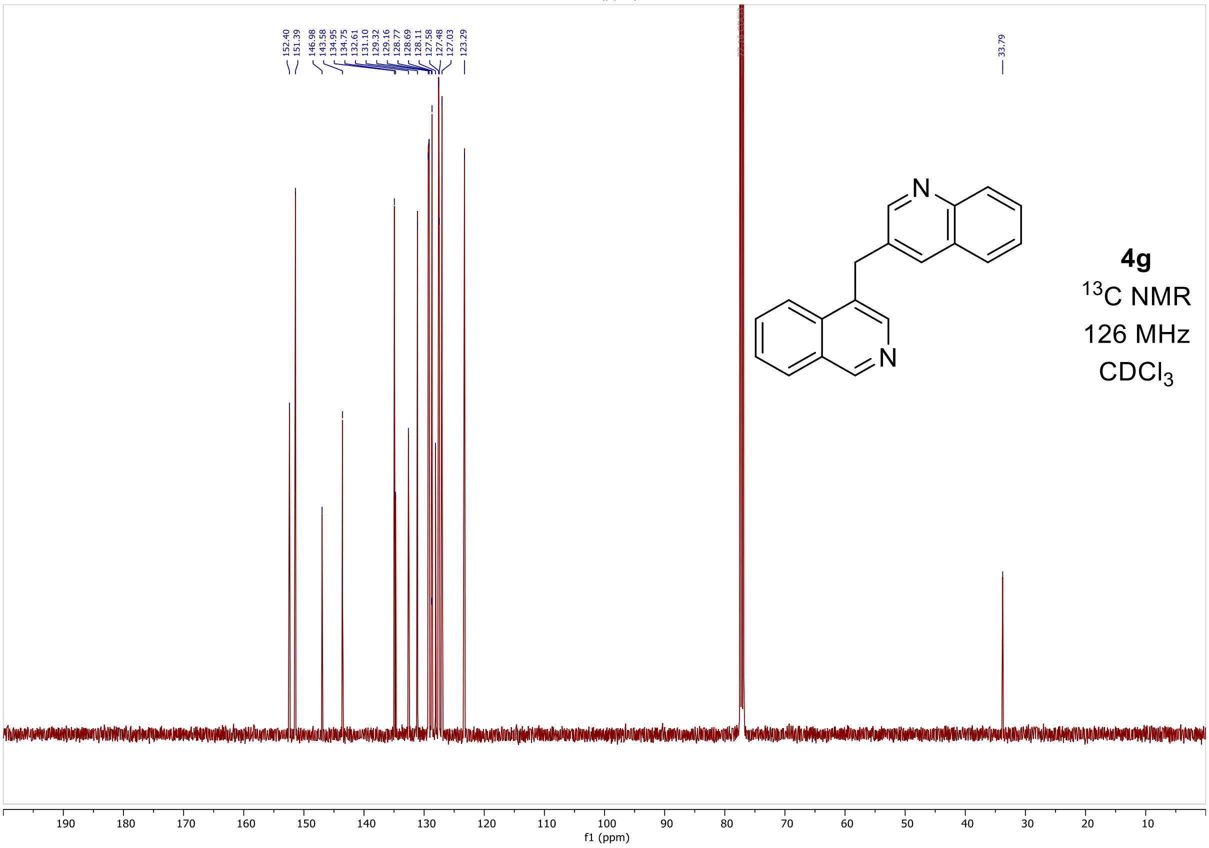
152.40
151.39
146.98
143.58
134.95
134.75
132.61
131.10
129.32
129.16
128.77
128.69
128.11
127.58
127.48
127.03
123.29
33.79
4g
13C NMR
126 MHz
CDCl3
190
180
170
160
150
140
130
120
110
100
90
80
70
60
50
40
30
20
10
f1 (ppm)

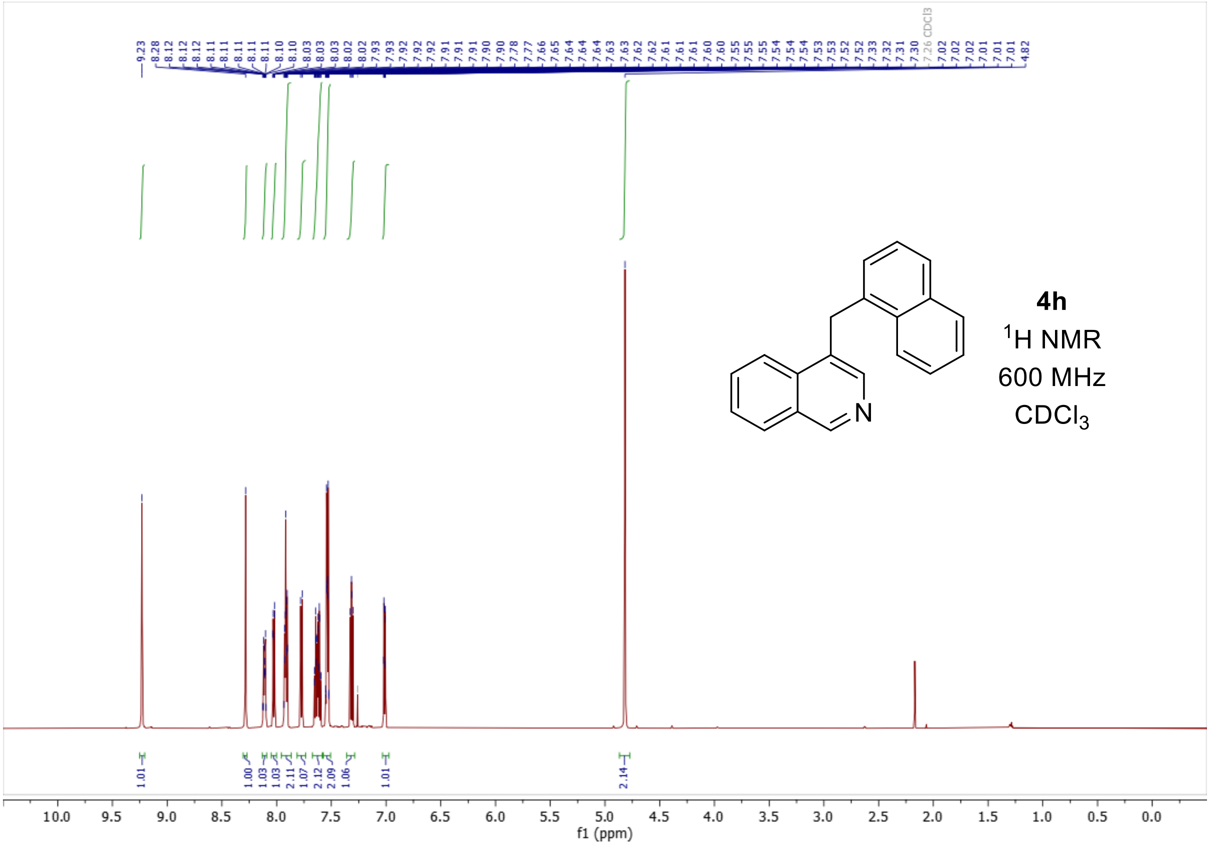
4h
1H NMR
600 MHz
CDCl3
f1 (ppm)

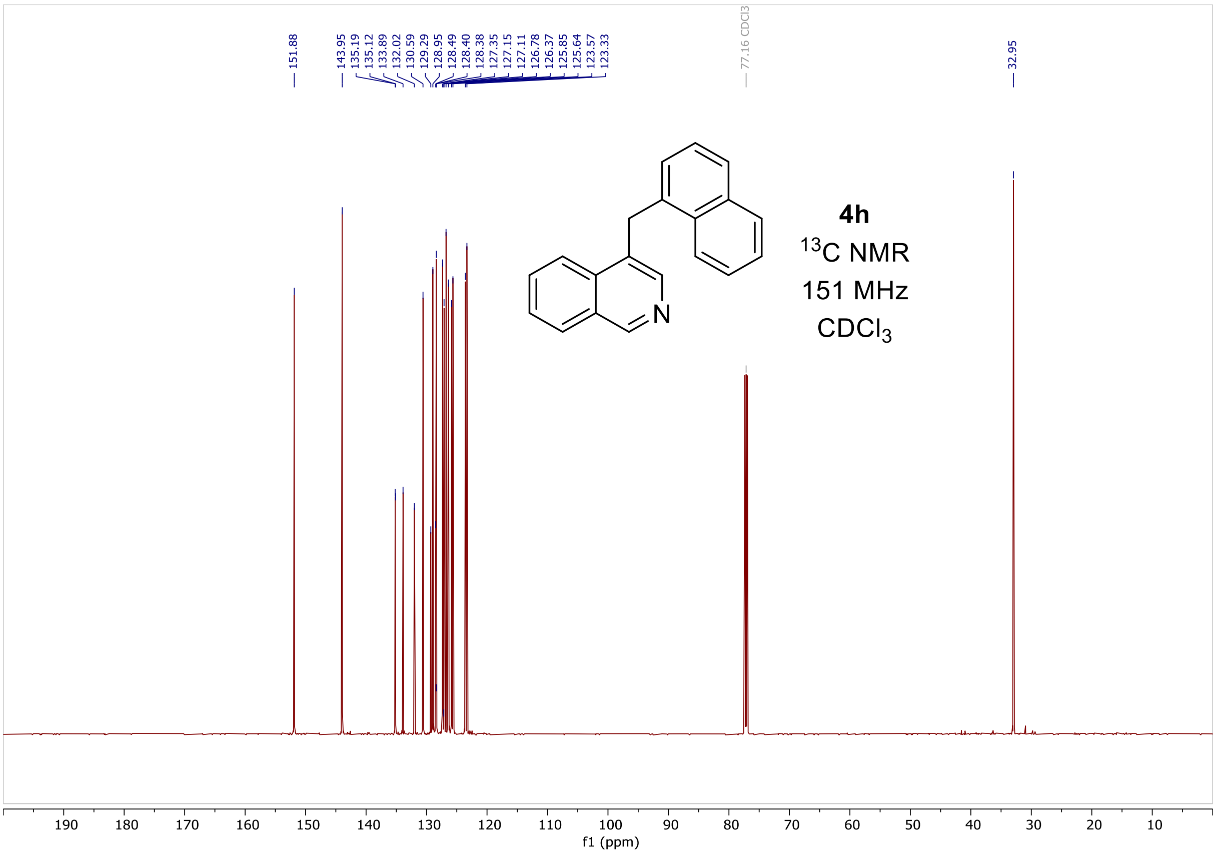
4h
13C NMR
151 MHz
CDCl3
f1 (ppm)

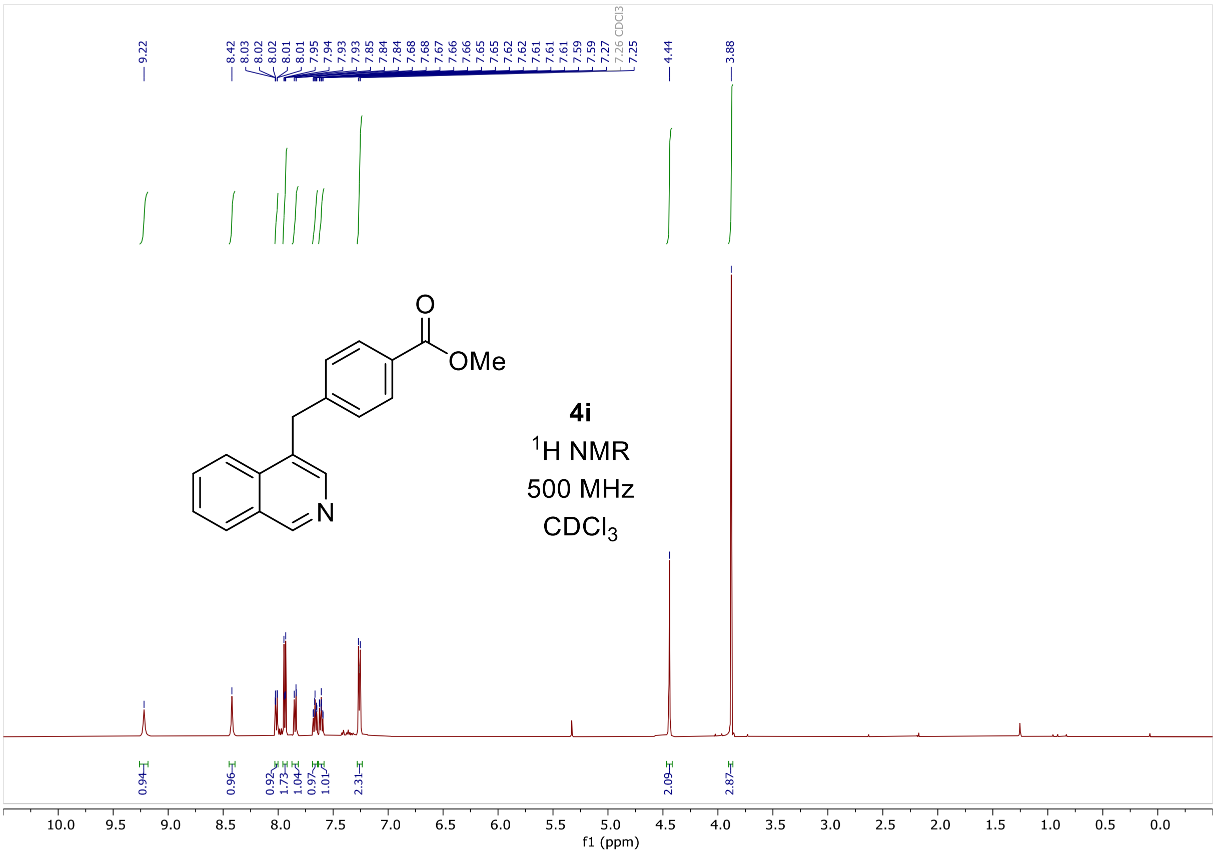
9.22
8.42
8.03
8.02
8.01
8.01
7.95
7.94
7.93
7.93
7.85
7.84
7.84
7.68
7.67
7.66
7.66
7.65
7.65
7.62
7.62
7.61
7.61
7.61
7.59
7.59
7.27
7.26 CDCl3
7.25
4.44
3.88
O
OMe
N
4i
^{1}H NMR
500 MHz
$CDCl_3$
0.94
0.96
0.92
1.73
1.04
0.97
1.01
2.31
2.09
2.87
10.0 9.5 9.0 8.5 8.0 7.5 7.0 6.5 6.0 5.5 5.0 4.5 4.0 3.5 3.0 2.5 2.0 1.5 1.0 0.5 0.0
f1 (ppm)

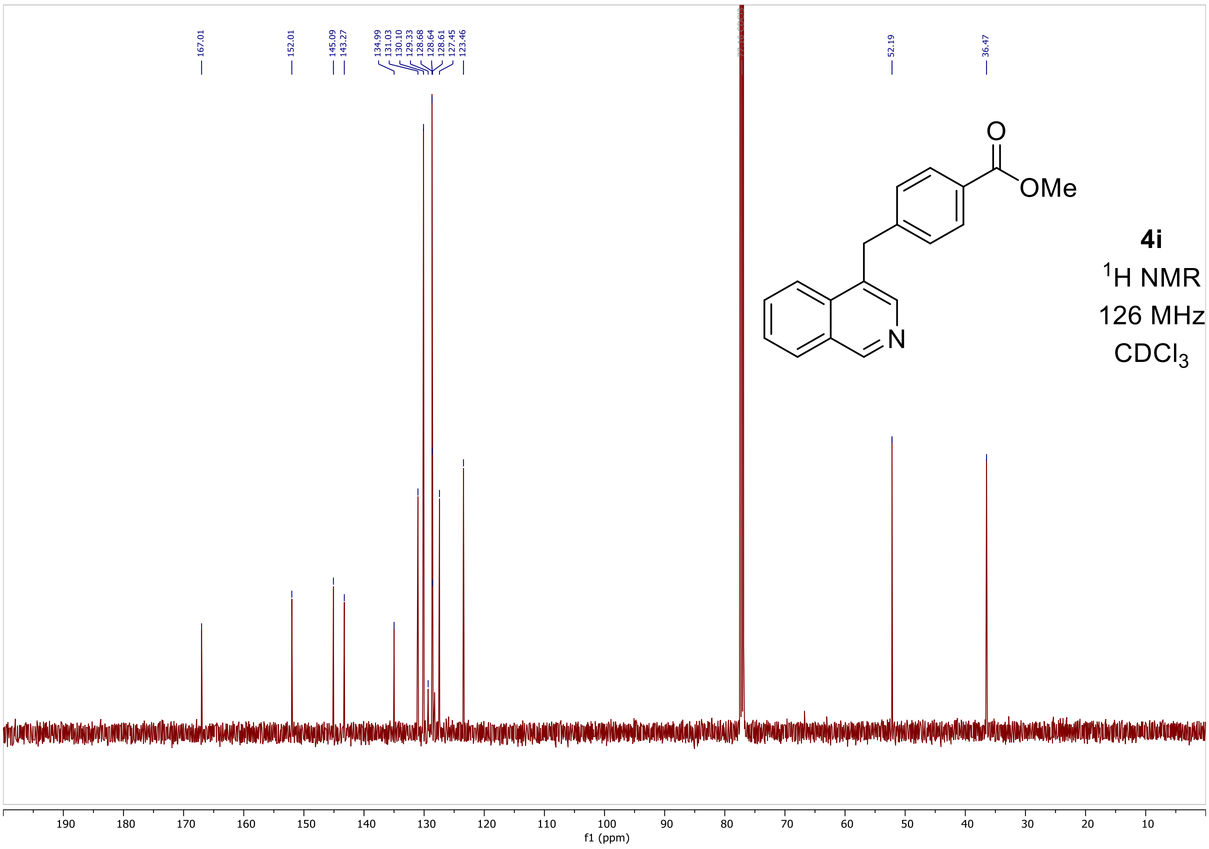
167.01
152.01
145.09
143.27
134.99
131.03
130.10
129.33
128.68
128.64
128.61
127.45
123.46
52.19
36.47
O
OMe
N
4i
^{1}H NMR
126 MHz
$CDCl_3$
190 180 170 160 150 140 130 120 110 100 90 80 70 60 50 40 30 20 10
f1 (ppm)

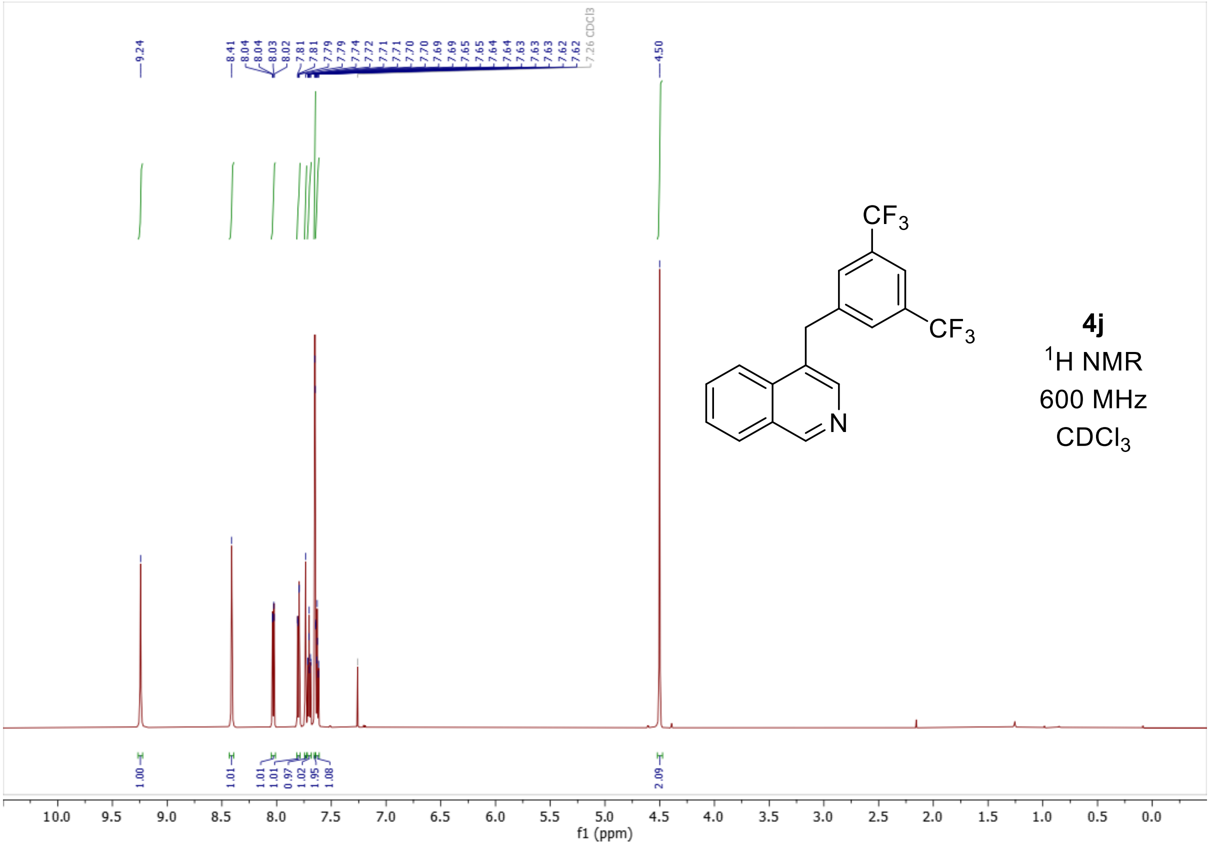
4j
1H NMR
600 MHz
CDCl3
f1 (ppm)

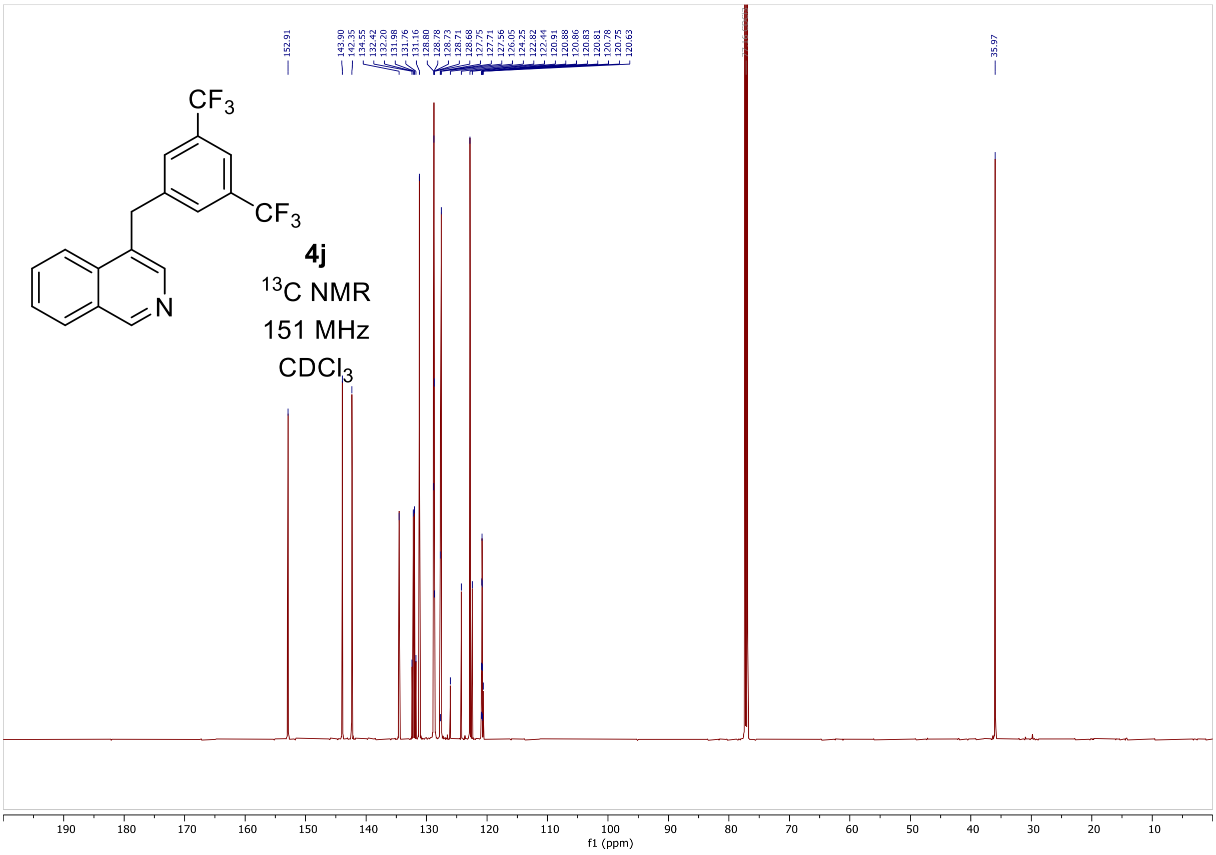
4j
13C NMR
151 MHz
CDCl3
f1 (ppm)

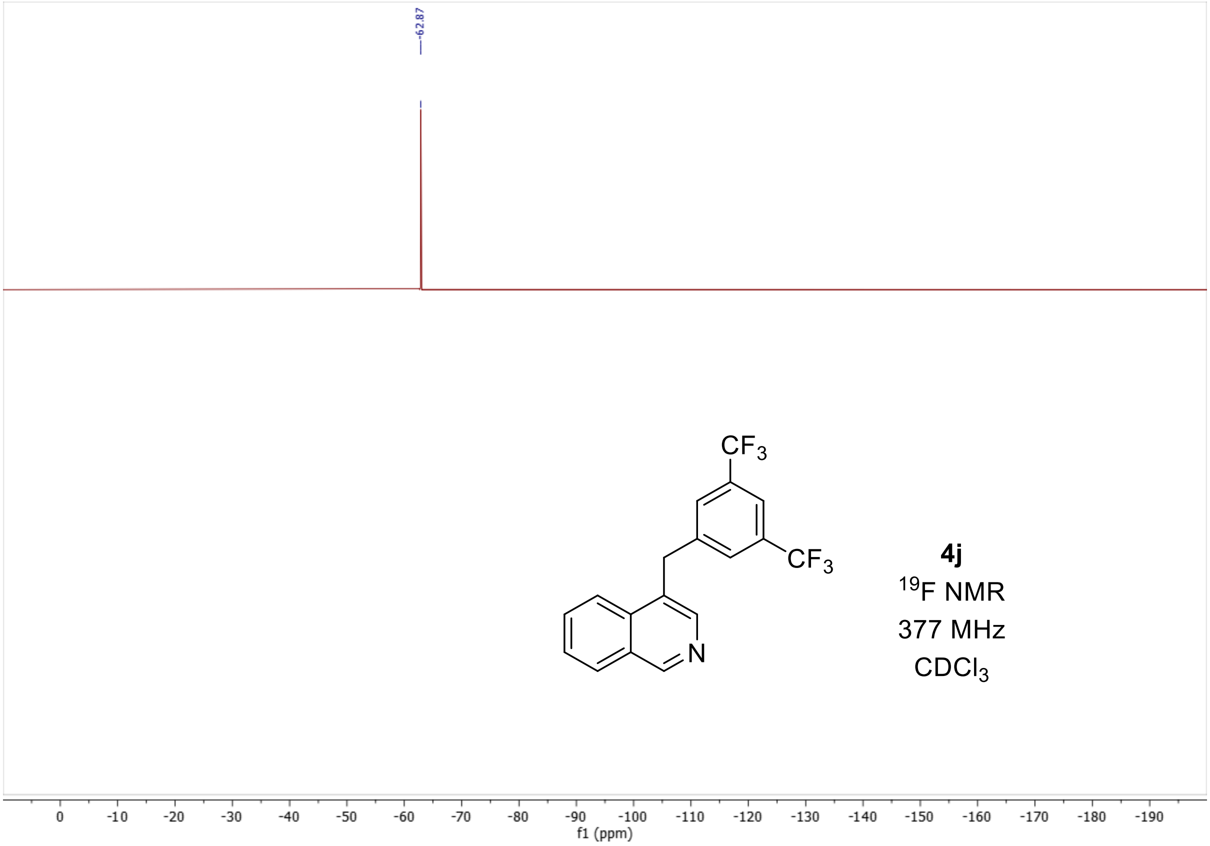

4j
^{19}F NMR
377 MHz
$CDCl_3$

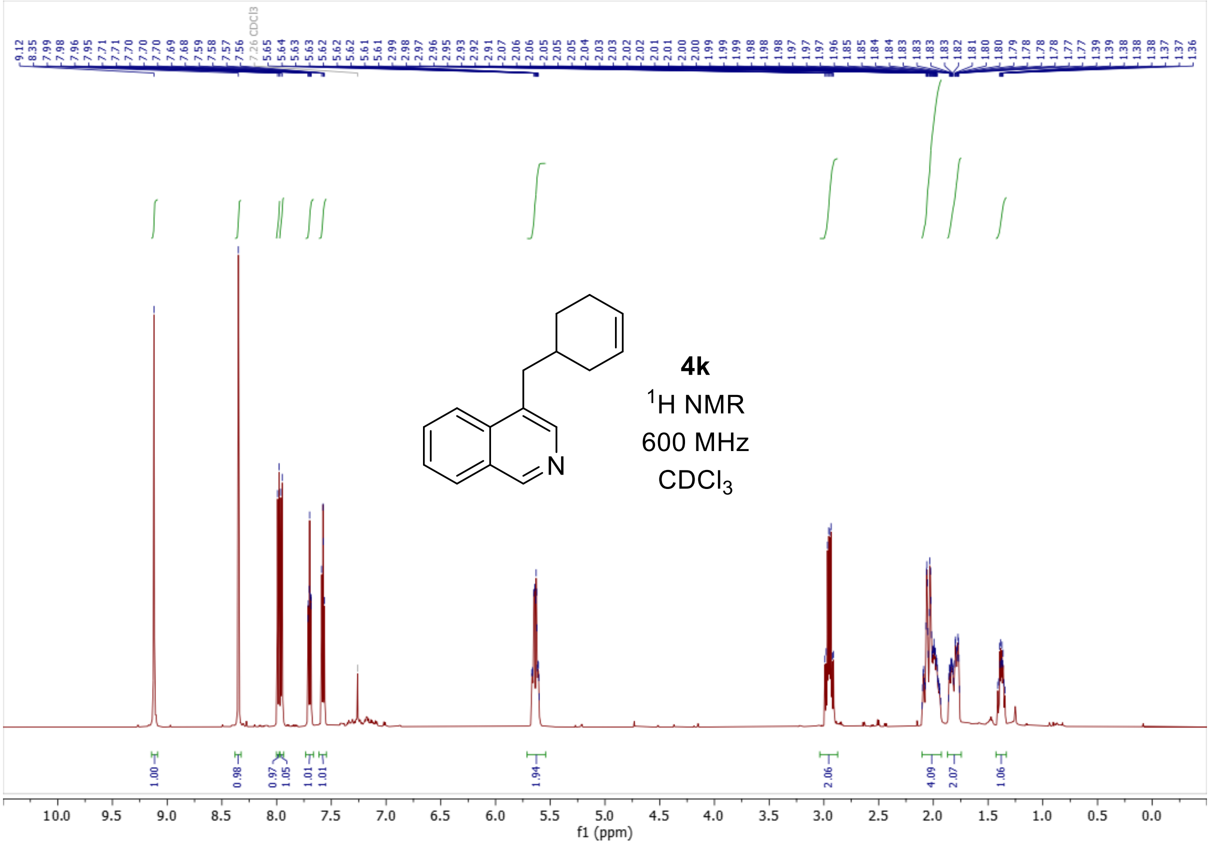
4k
^{1}H NMR
600 MHz
$CDCl_3$
f1 (ppm)

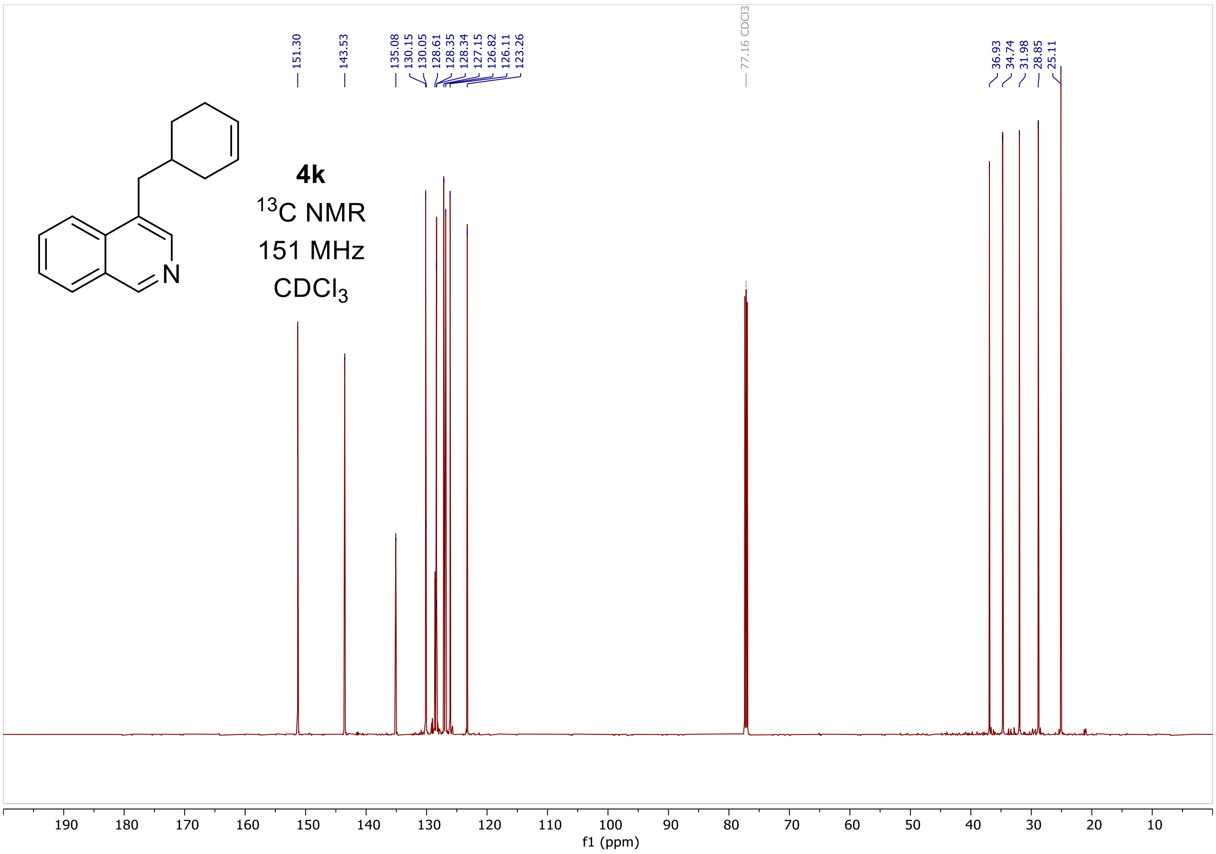
4k
^{13}C NMR
151 MHz
$CDCl_3$
151.30
143.53
135.08
130.15
130.05
128.61
128.35
128.34
127.15
126.82
126.11
123.26
77.16 CDCl3
36.93
34.74
31.98
28.85
25.11
f1 (ppm)

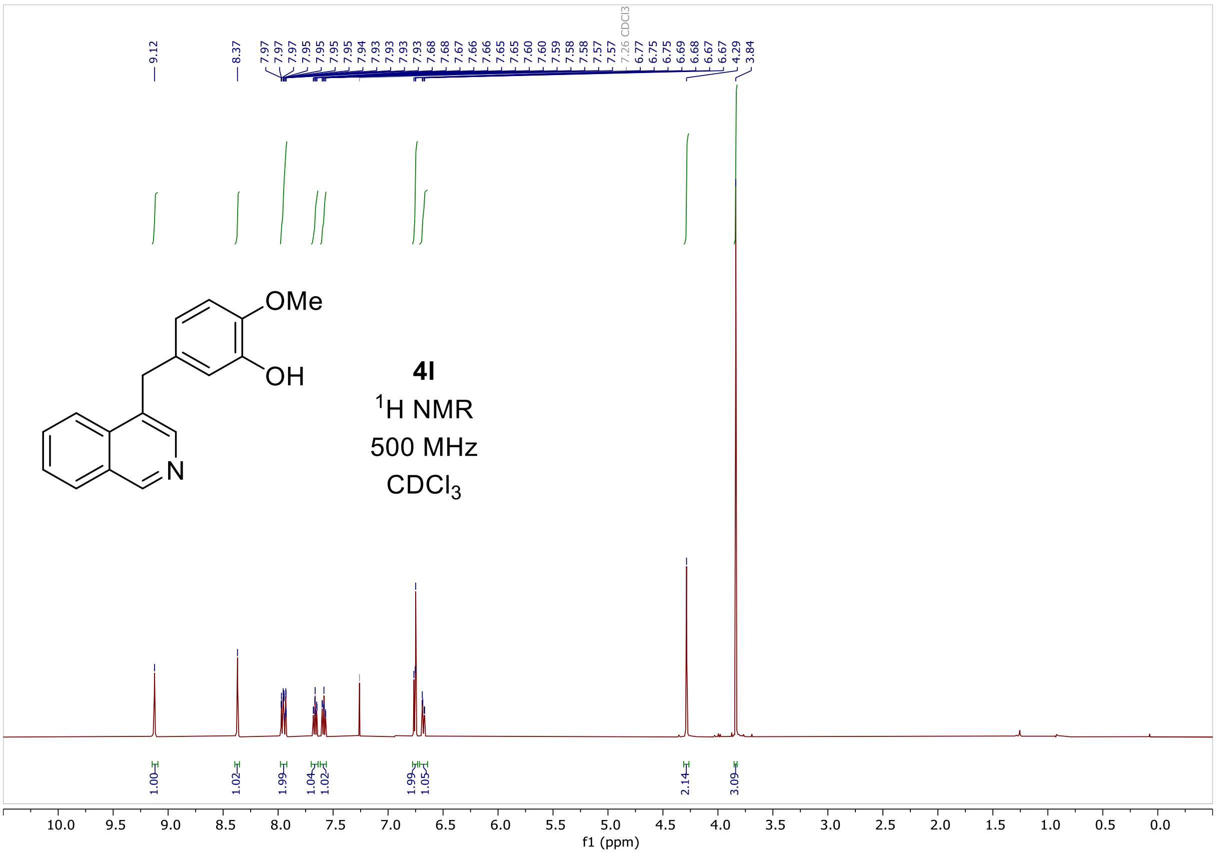
OMe
OH
N
4l
1H NMR
500 MHz
CDCl3
f1 (ppm)

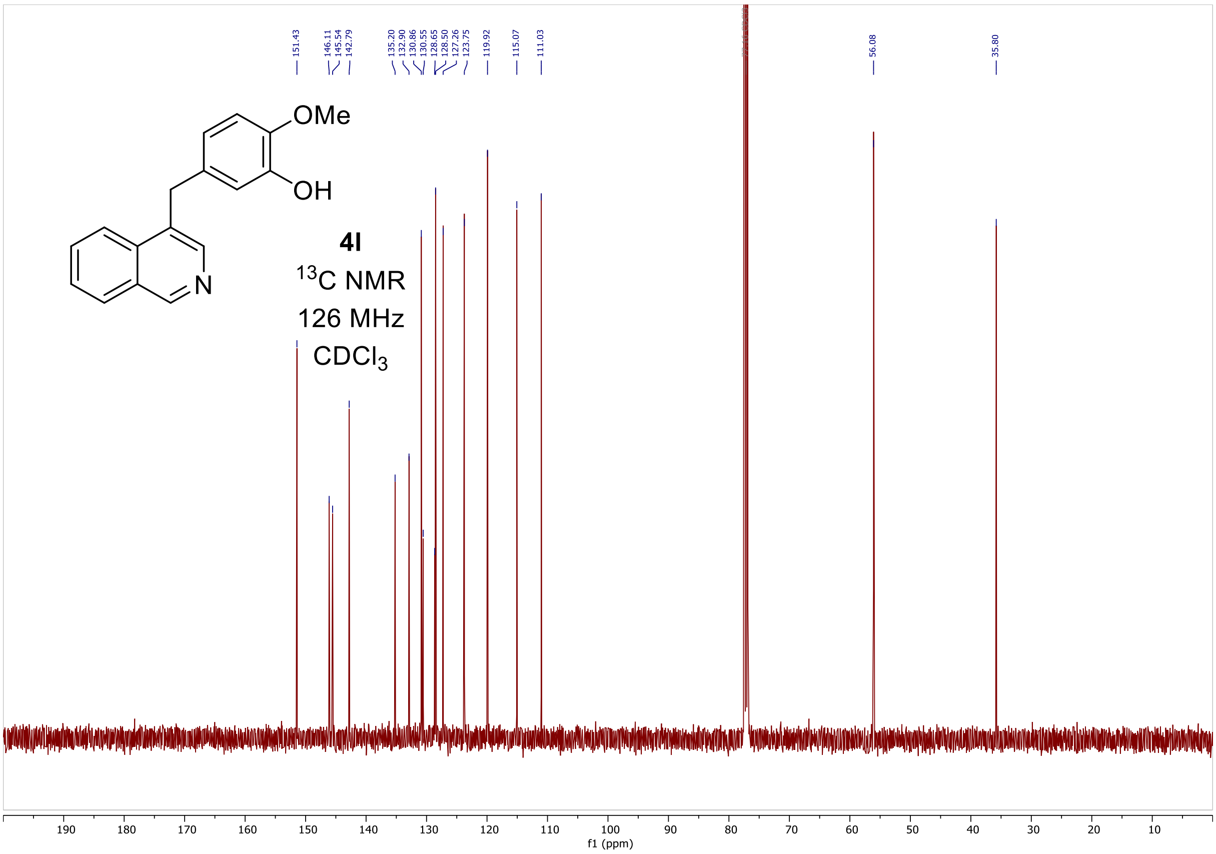
OMe
OH
N
4l
13C NMR
126 MHz
CDCl3
f1 (ppm)

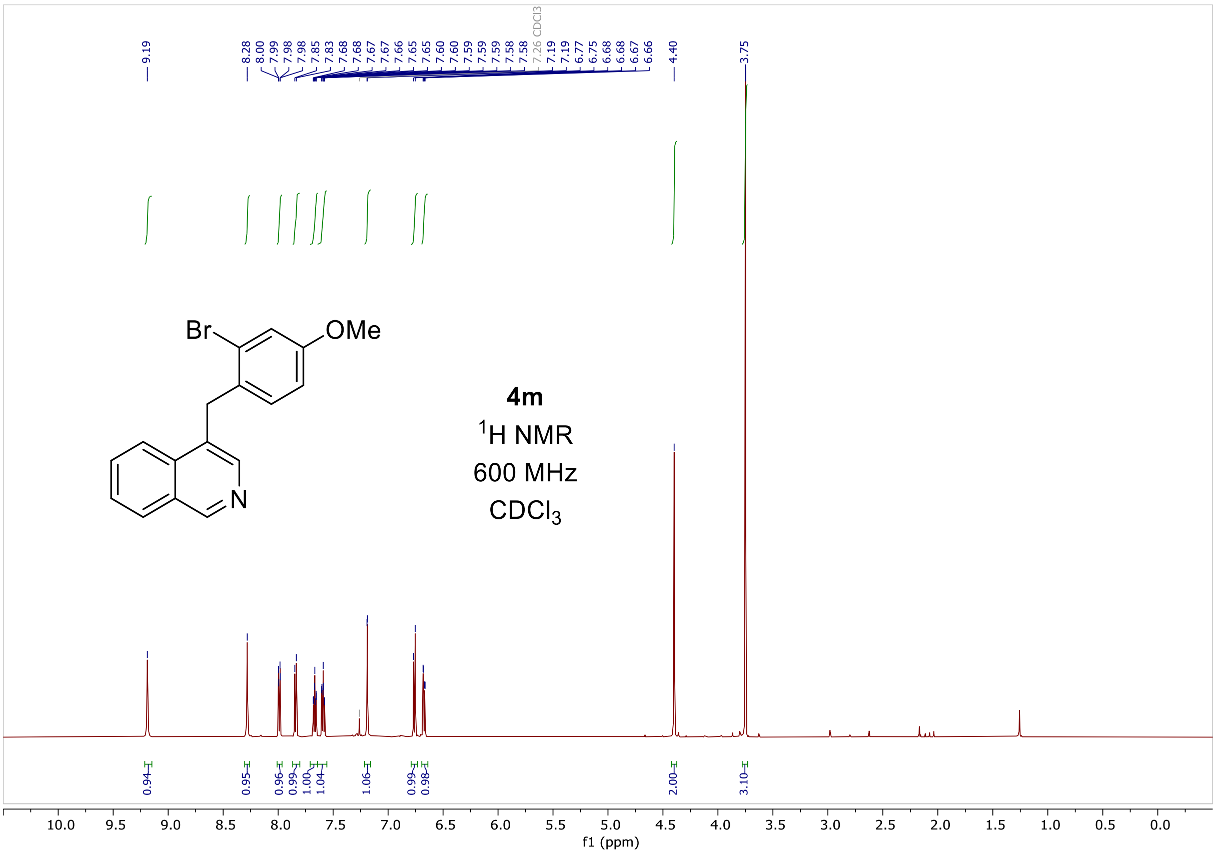
Br
OMe
N
4m
^{1}H NMR
600 MHz
$CDCl_3$
f1 (ppm)

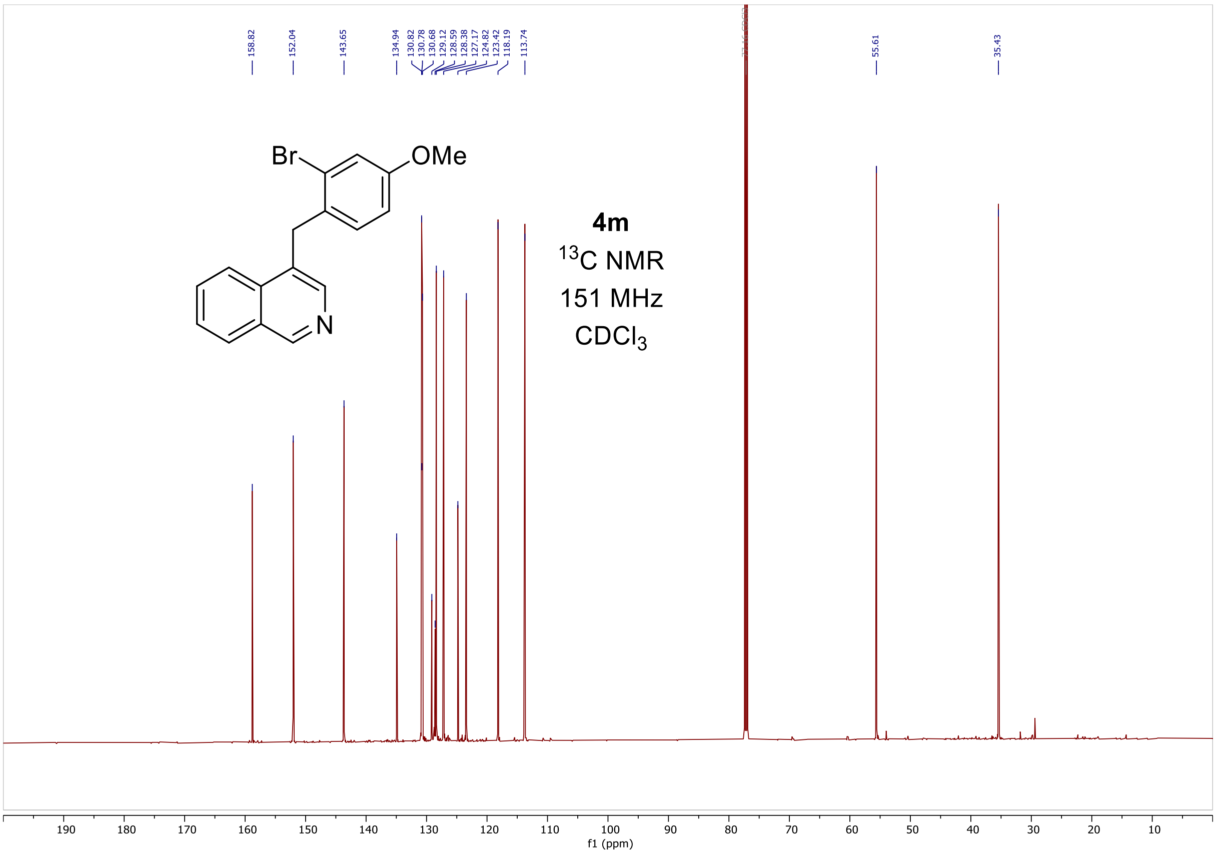
Br
OMe
N
4m
^{13}C NMR
151 MHz
$CDCl_3$
f1 (ppm)

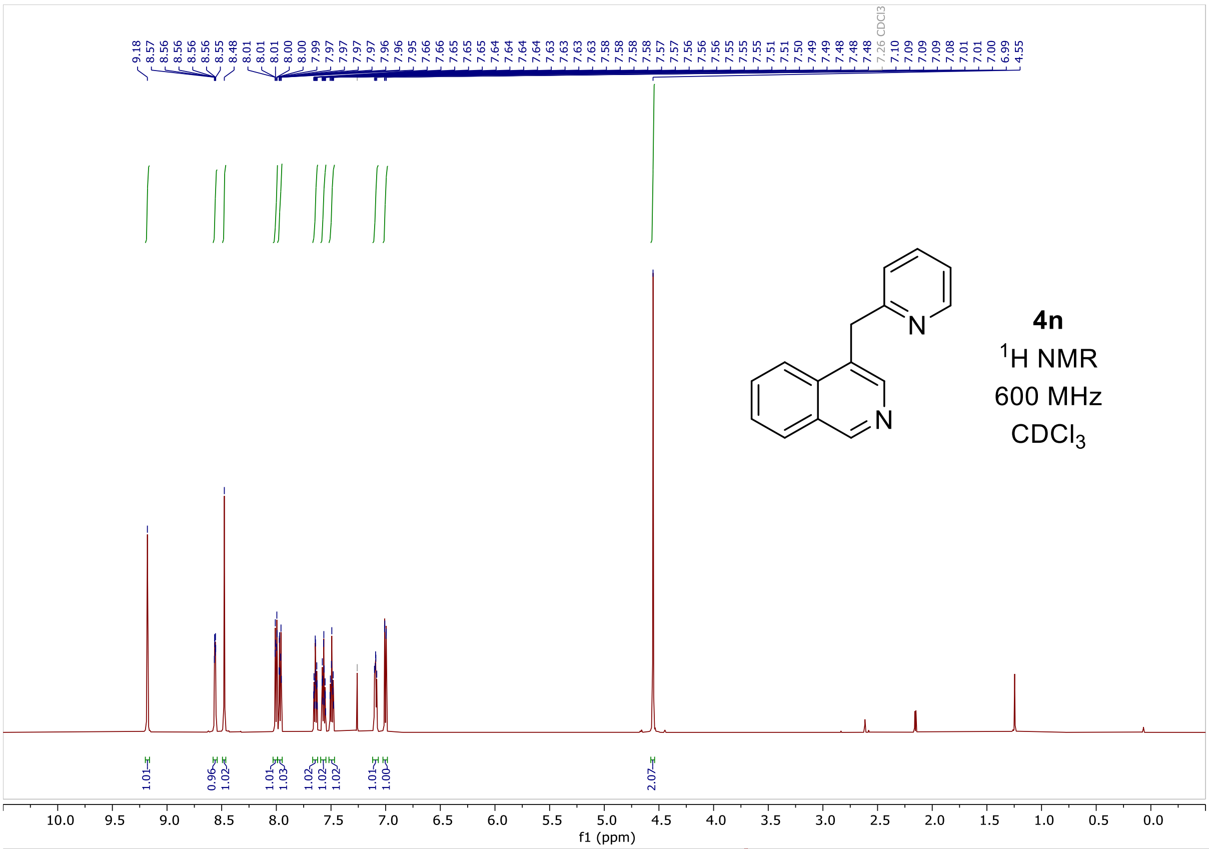
4n
^{1}H NMR
600 MHz
$CDCl_3$

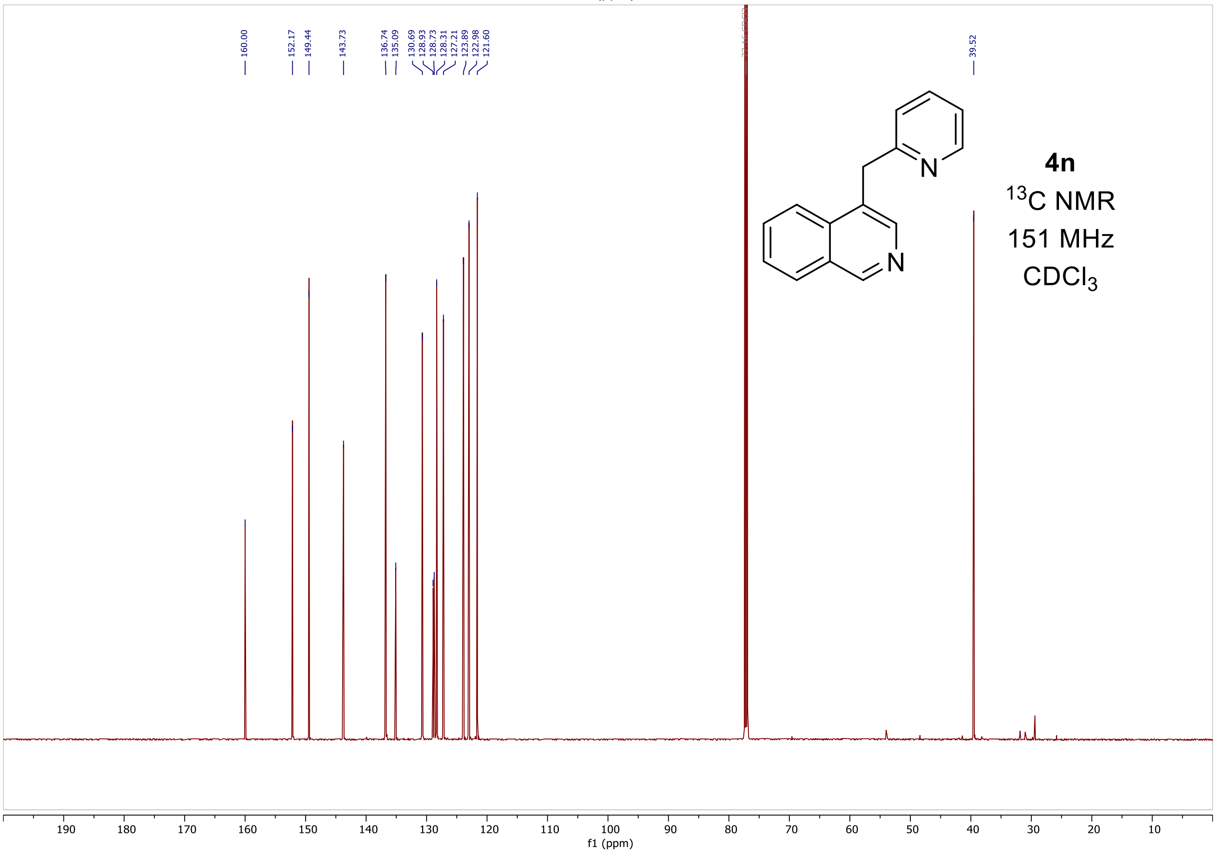
4n
^{13}C NMR
151 MHz
$CDCl_3$

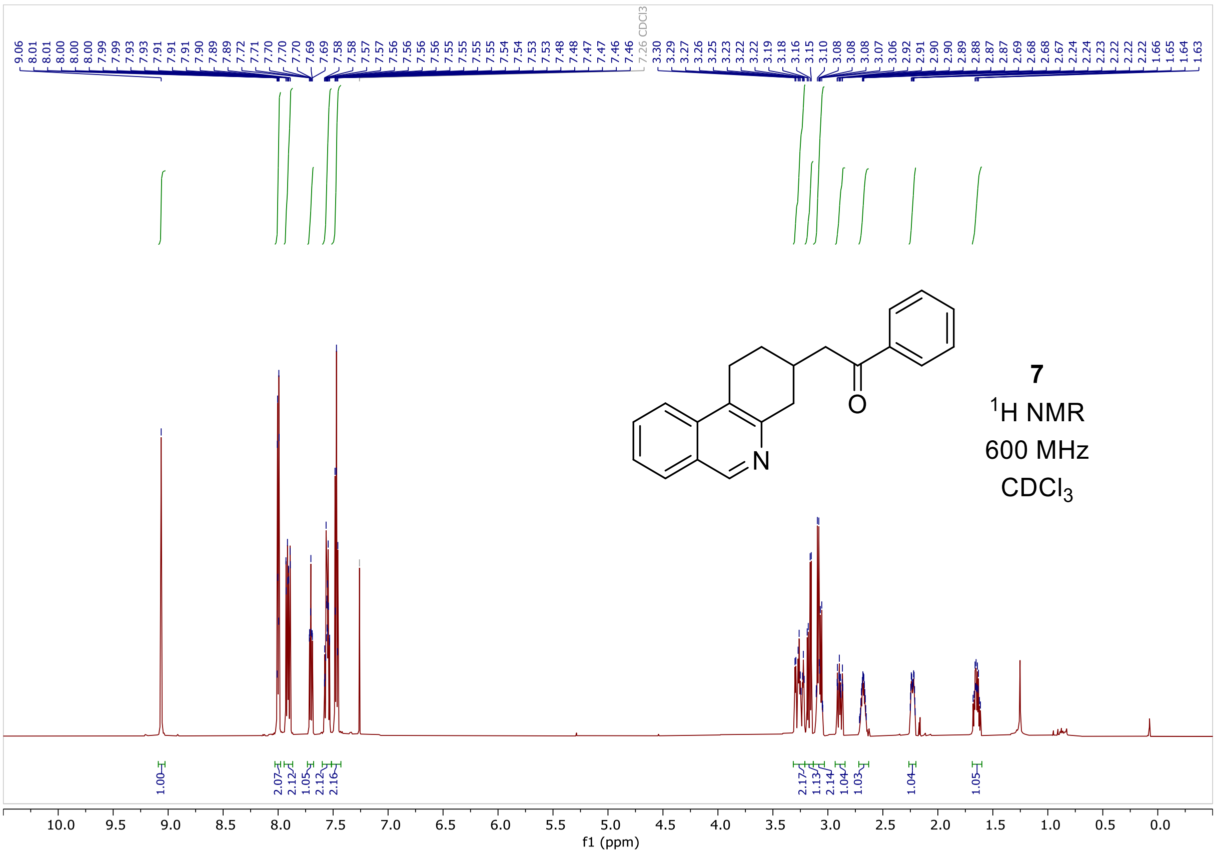
7
1H NMR
600 MHz
CDCl3
f1 (ppm)

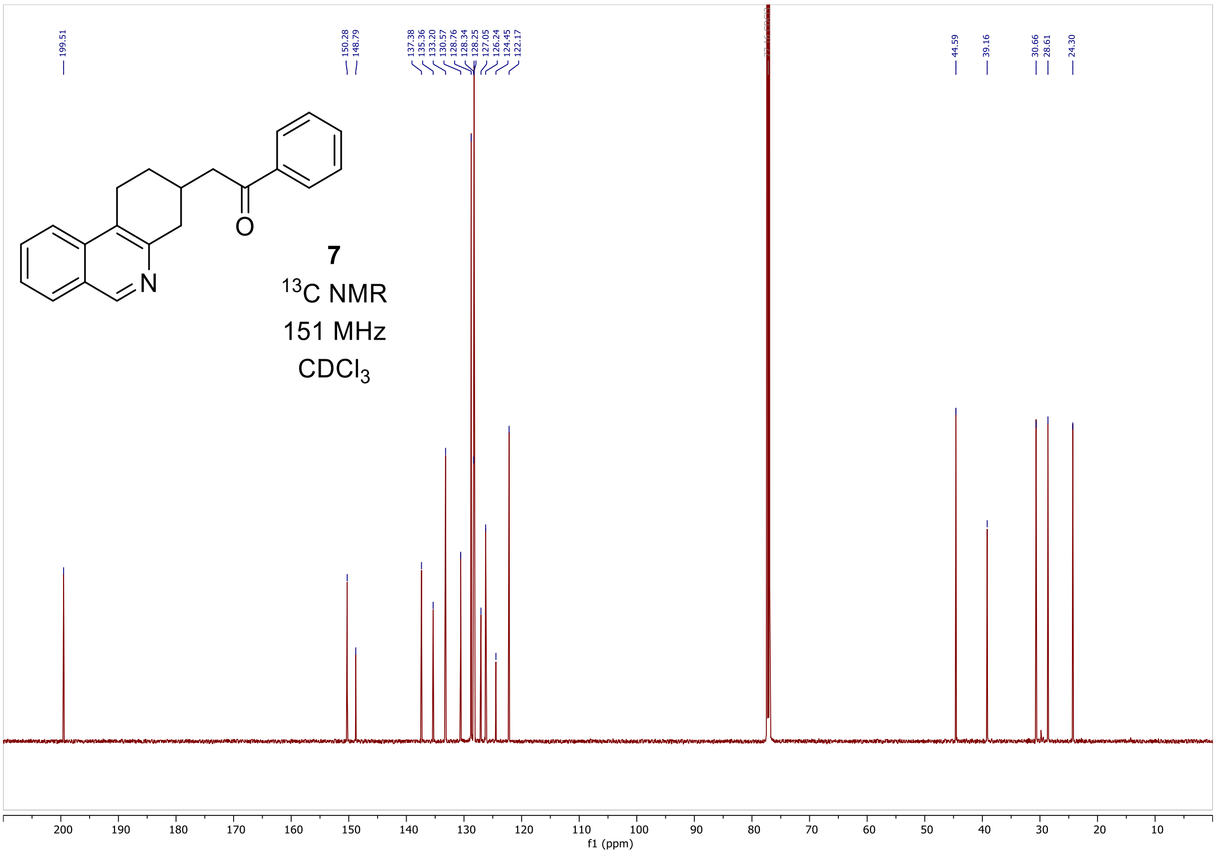
199.51
150.28
148.79
137.38
135.36
133.20
130.57
128.76
128.34
128.25
127.05
126.24
124.45
122.17
44.59
39.16
30.66
28.61
24.30
7
13C NMR
151 MHz
CDCl3
f1 (ppm)

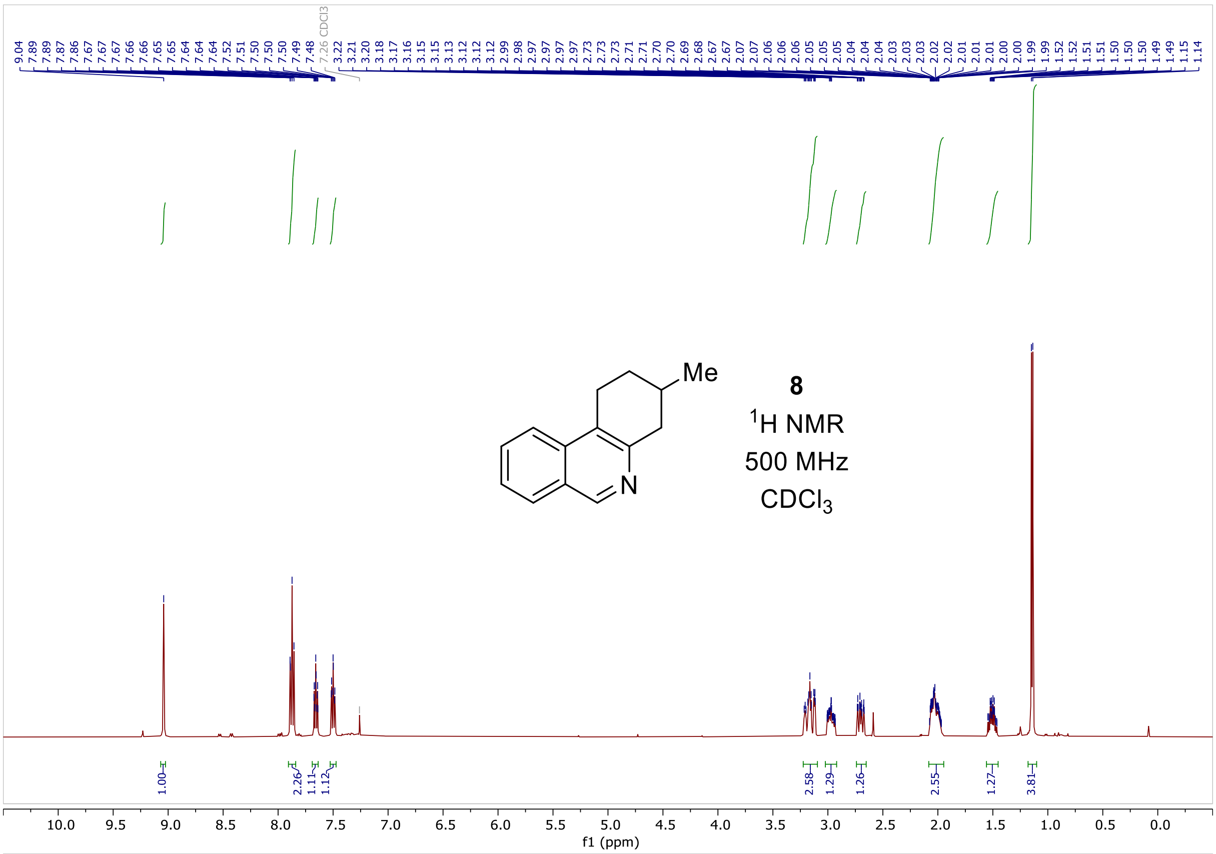

Me
N
8
^{1}H NMR
500 MHz
$CDCl_3$
7.26 CDCl3
1.00
2.26
1.11
1.12
2.58
1.29
1.26
2.55
1.27
3.81
f1 (ppm)

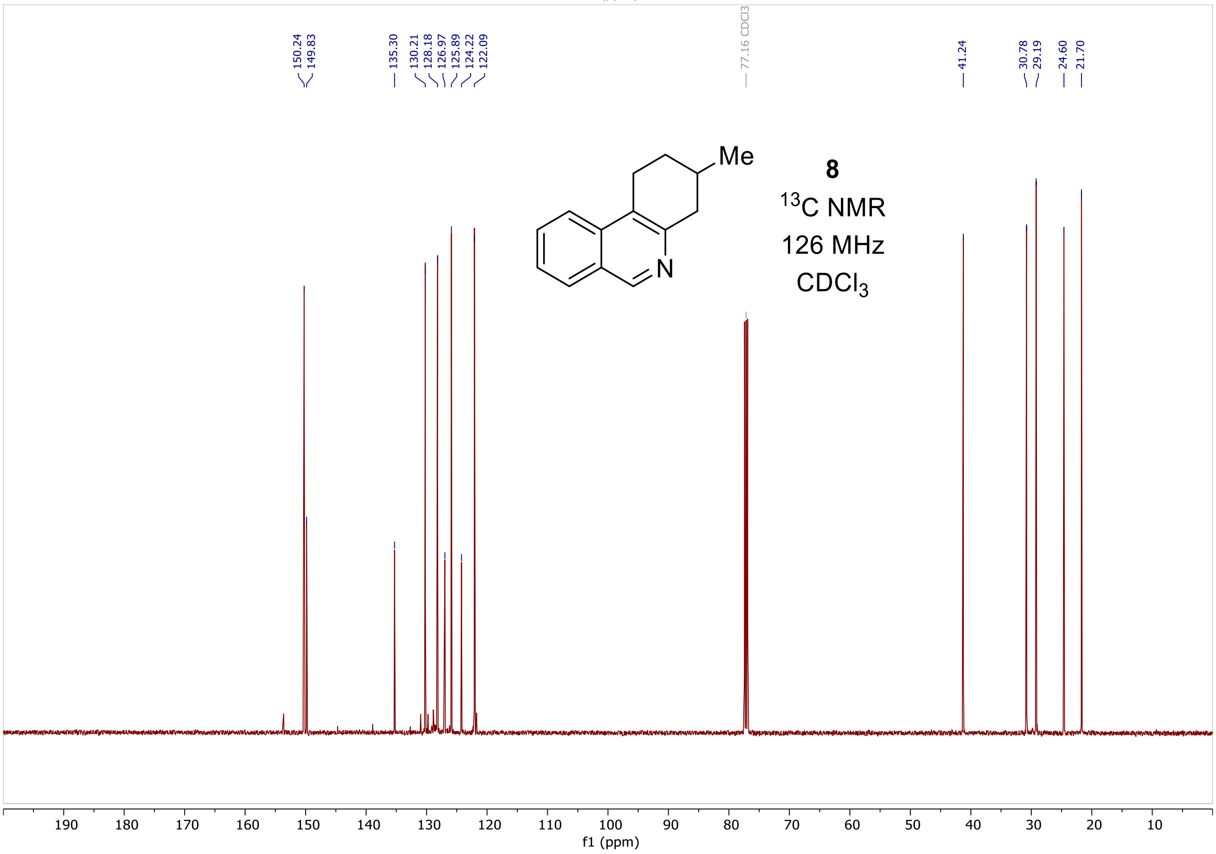

150.24
149.83
135.30
130.21
128.18
126.97
125.89
124.22
122.09
77.16 CDCl3
41.24
30.78
29.19
24.60
21.70
Me
N
8
^{13}C NMR
126 MHz
$CDCl_3$
f1 (ppm)

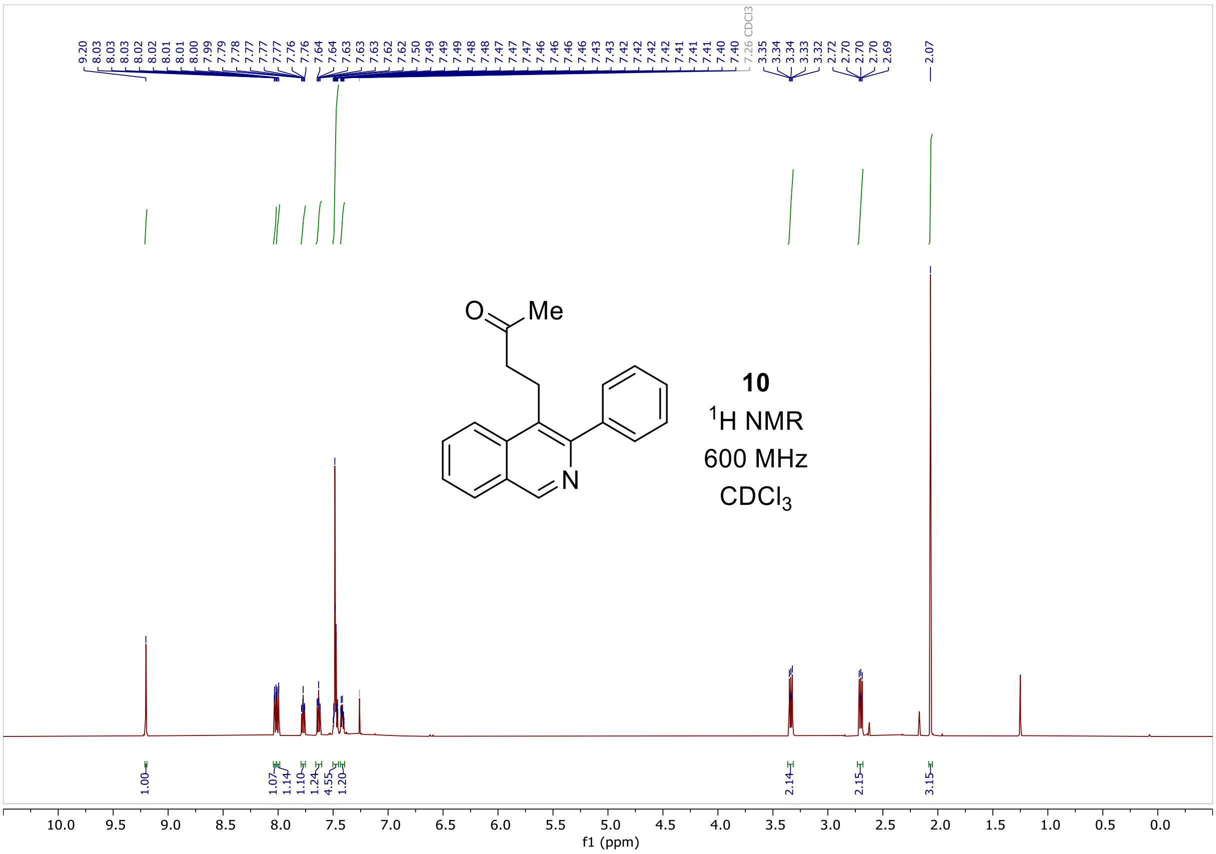
10
1H NMR
600 MHz
CDCl3
f1 (ppm)

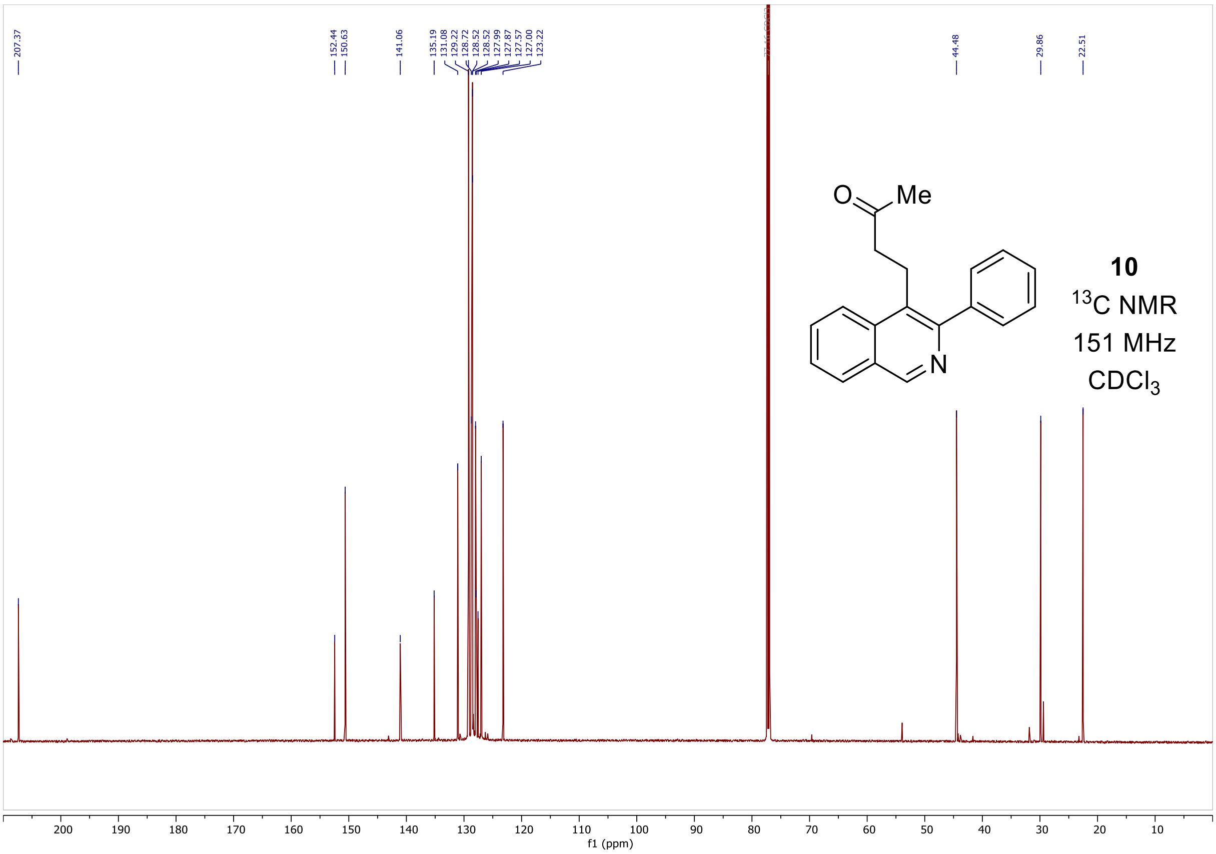
10
13C NMR
151 MHz
CDCl3
f1 (ppm)

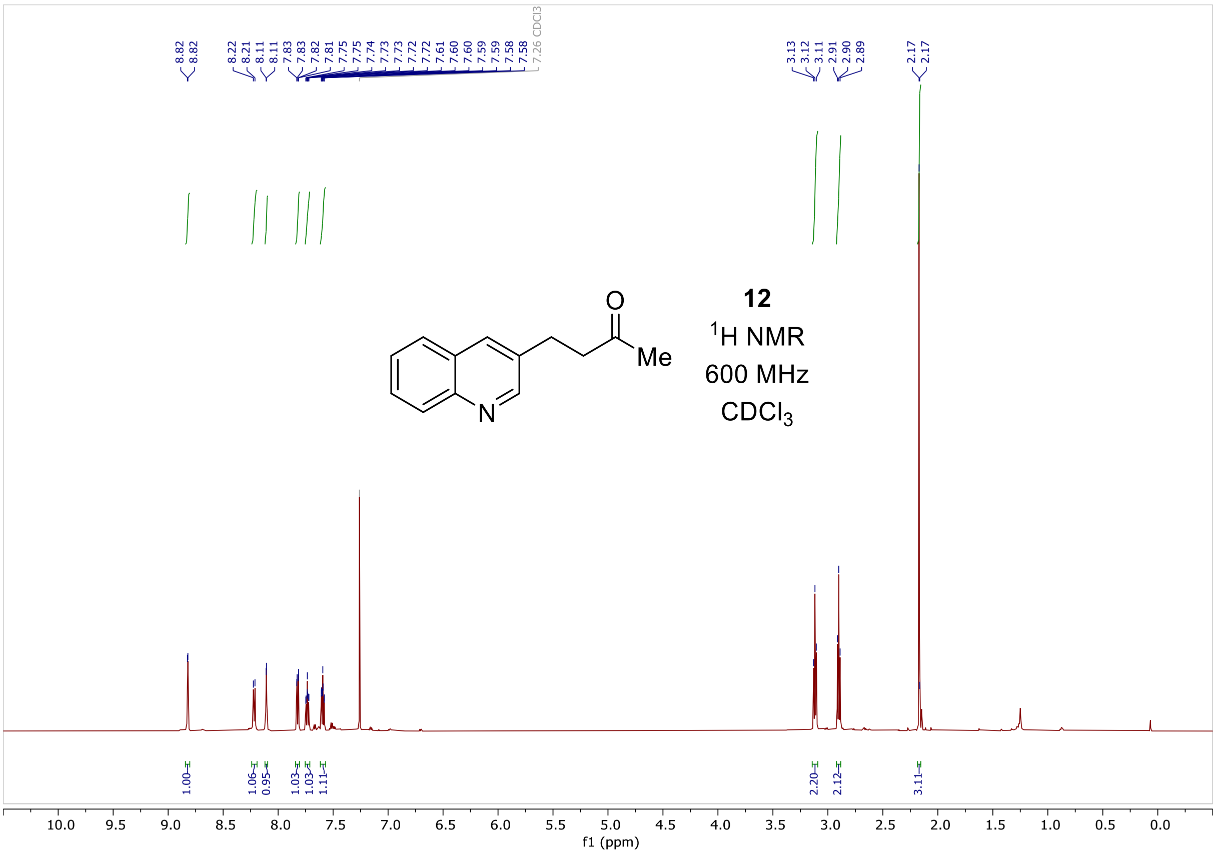
12
^{1}H NMR
600 MHz
$CDCl_3$
f1 (ppm)

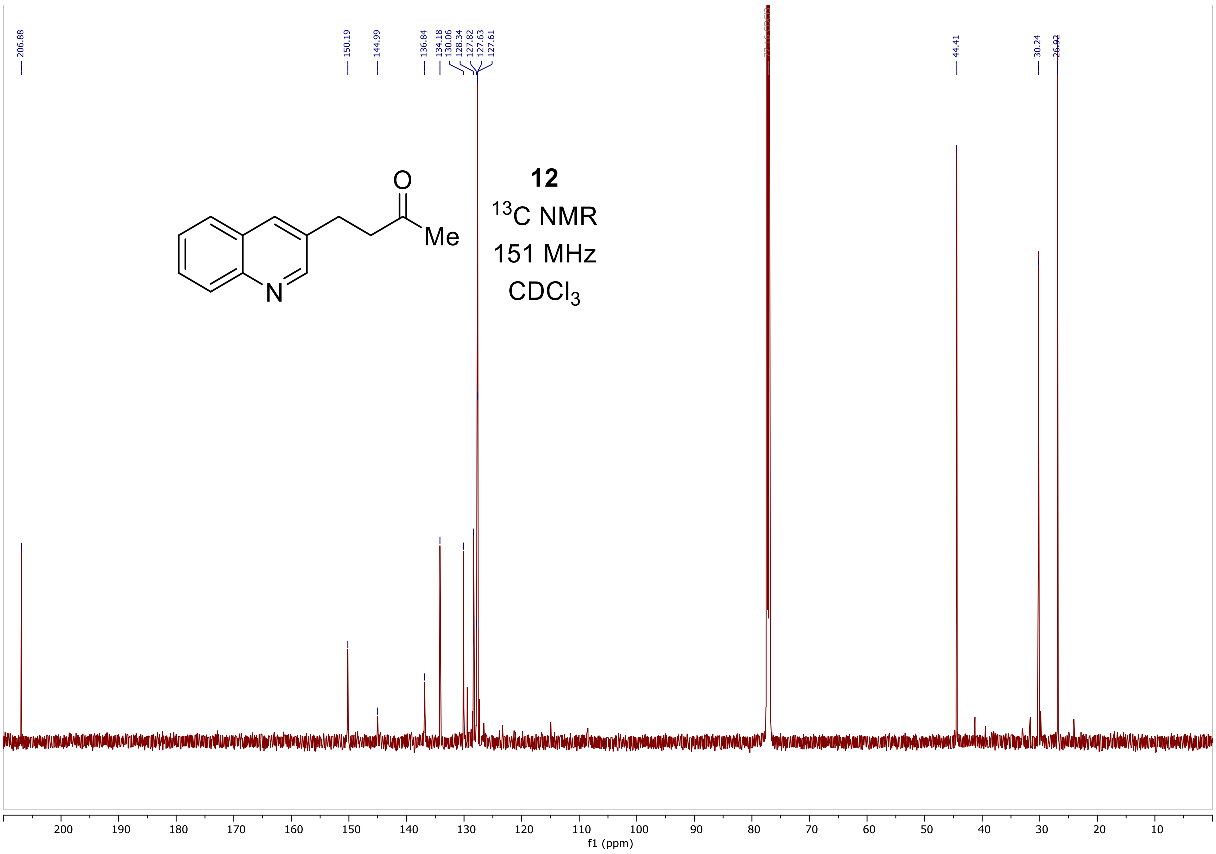
12
^{13}C NMR
151 MHz
$CDCl_3$
f1 (ppm)

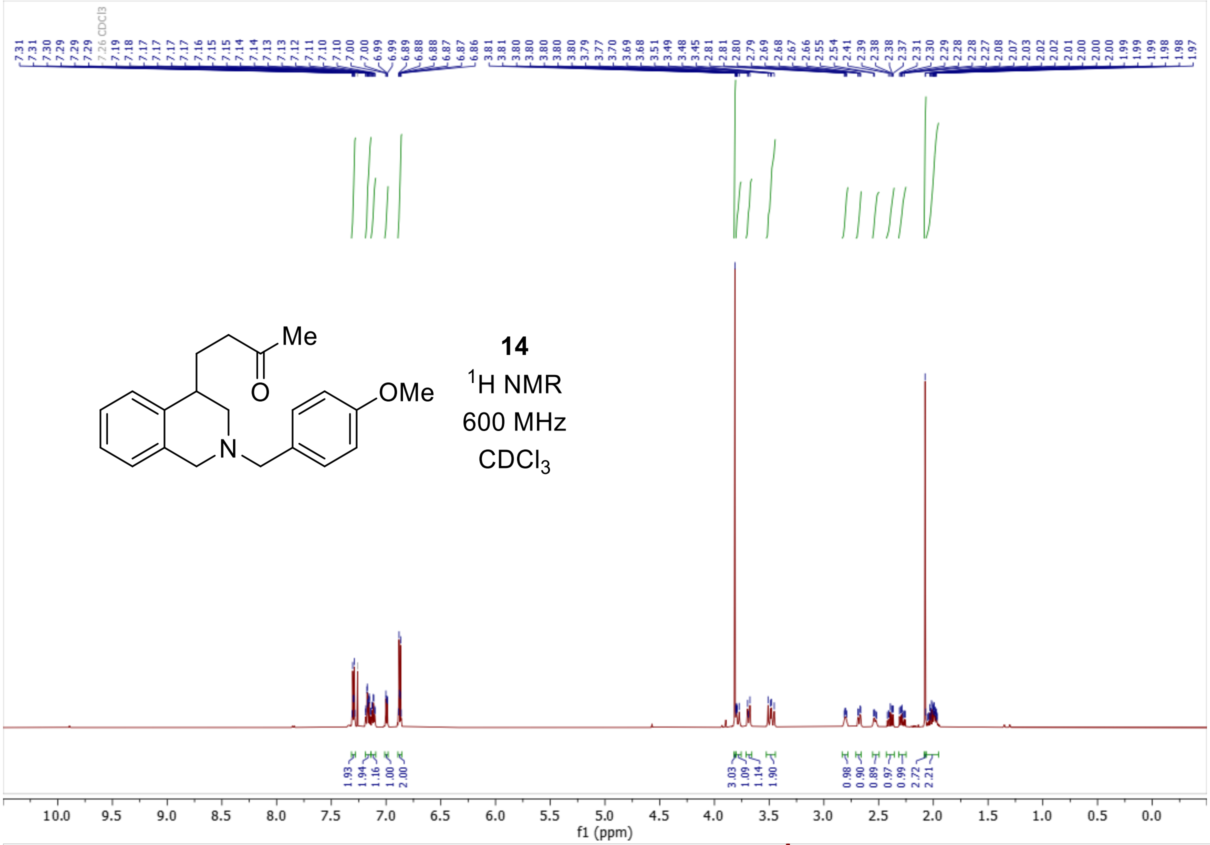
Me
O
OMe
N
14
^{1}H NMR
600 MHz
$CDCl_3$
7.26 CDCl3
f1 (ppm)

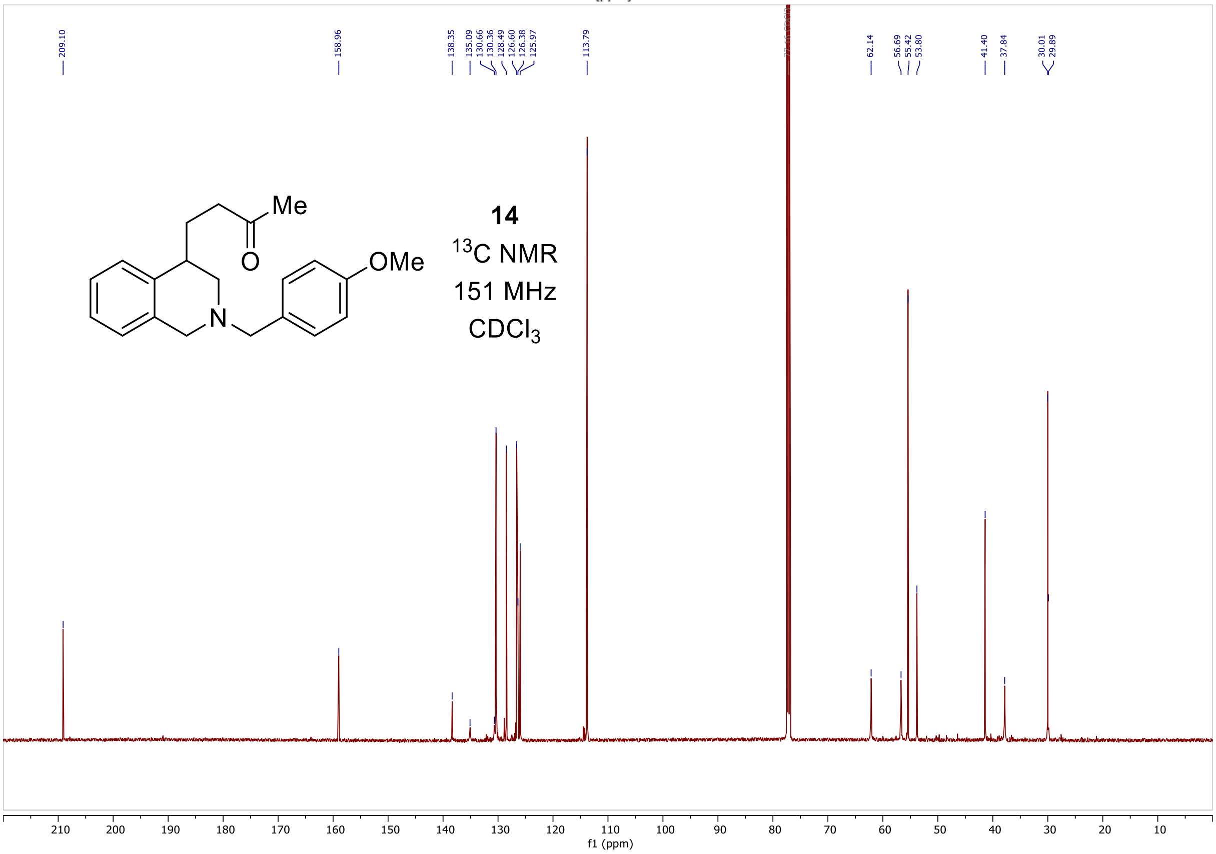
209.10
158.96
138.35
135.09
130.66
130.36
128.49
126.60
126.38
125.97
113.79
62.14
56.69
55.42
53.80
41.40
37.84
30.01
29.89
Me
O
OMe
N
14
^{13}C NMR
151 MHz
$CDCl_3$
f1 (ppm)

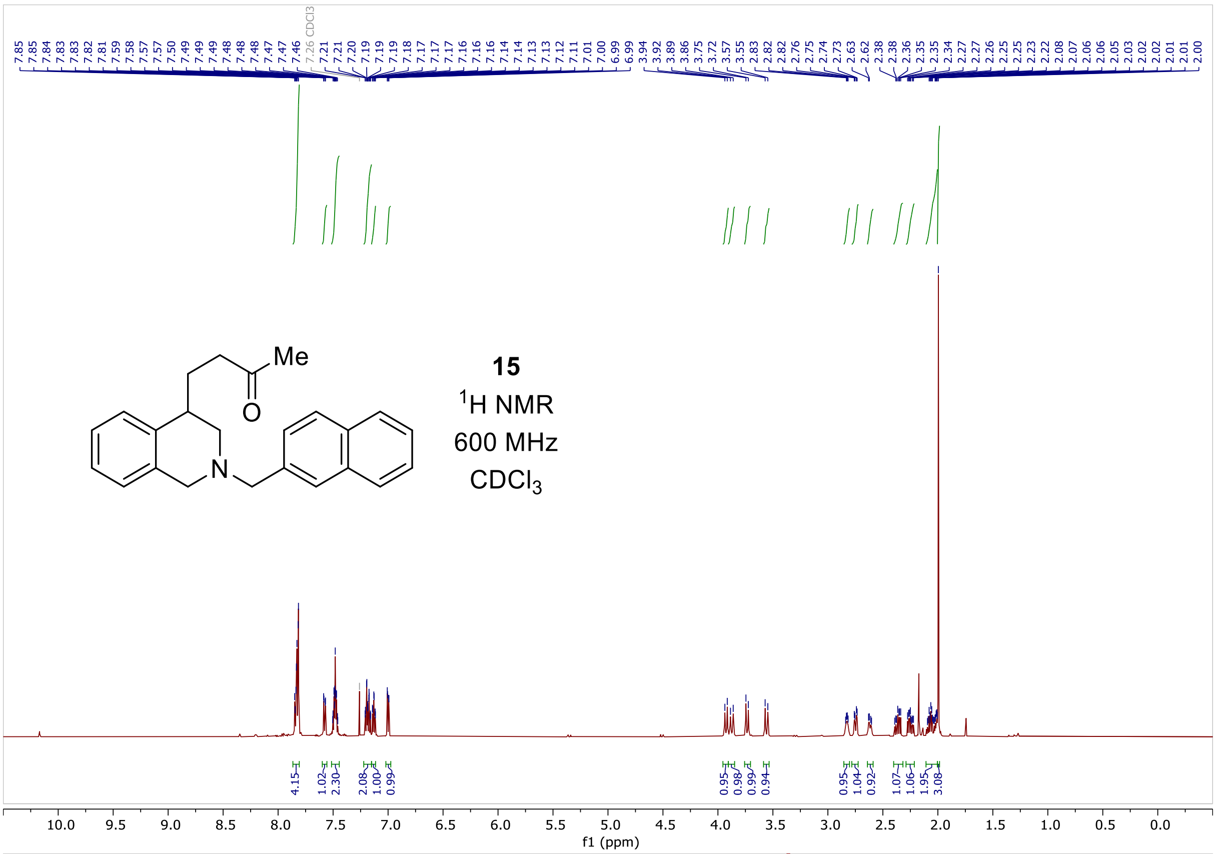

15
^{1}H NMR
600 MHz
$CDCl_3$

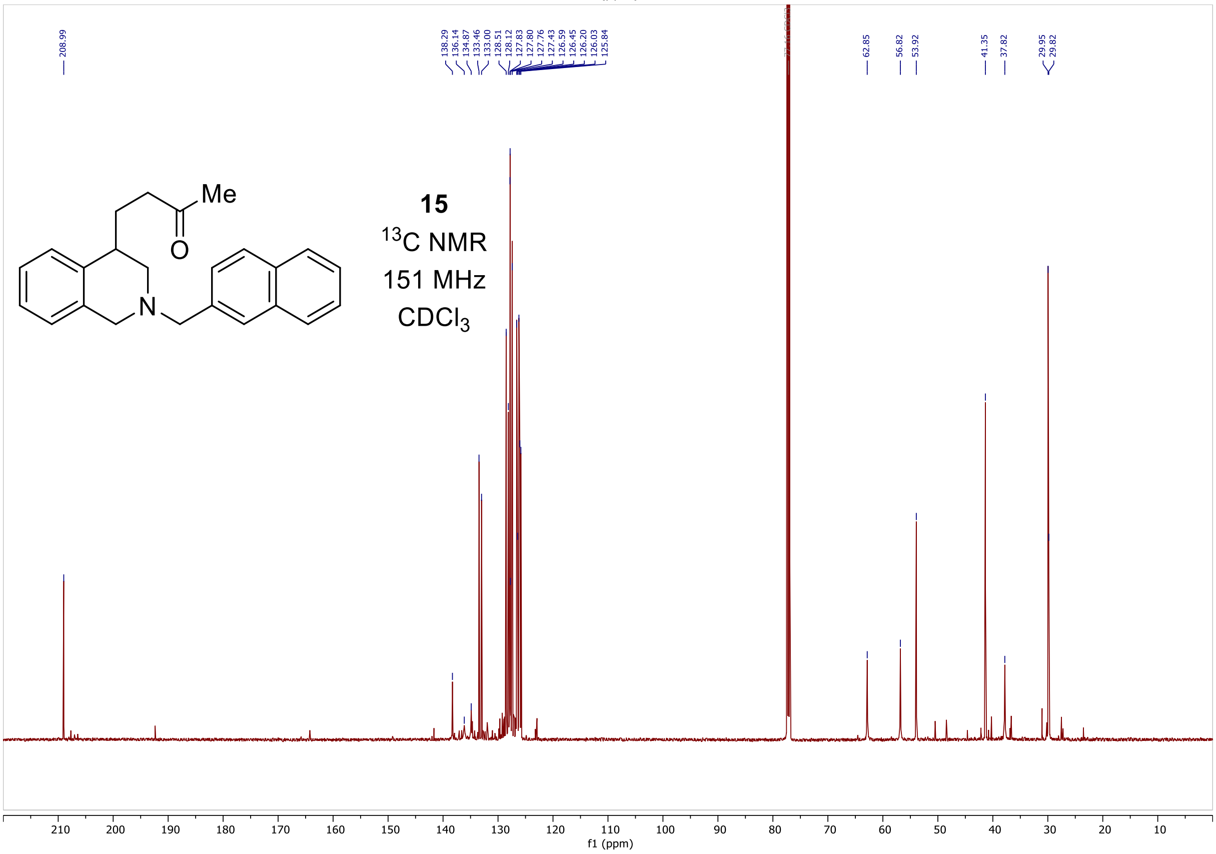

15
^{13}C NMR
151 MHz
$CDCl_3$

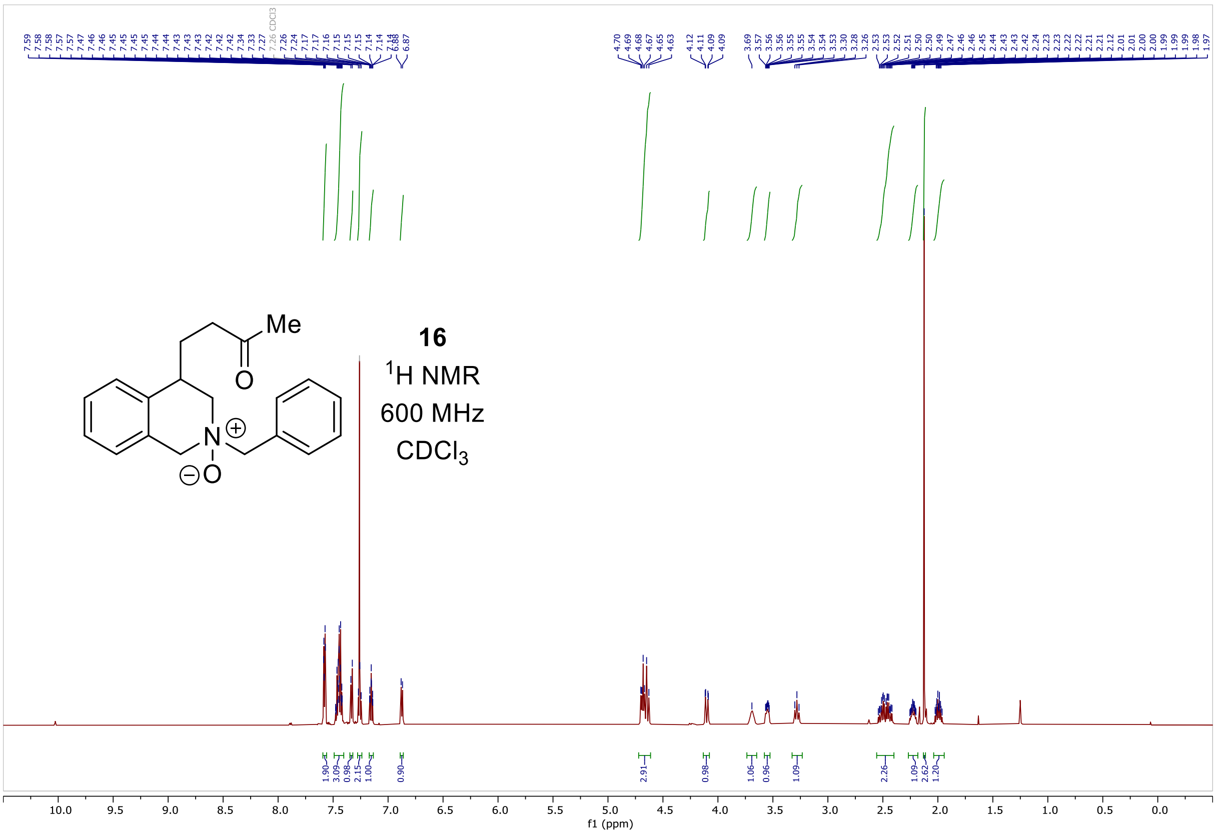
16
1H NMR
600 MHz
CDCl3
Me
O
N
O
f1 (ppm)

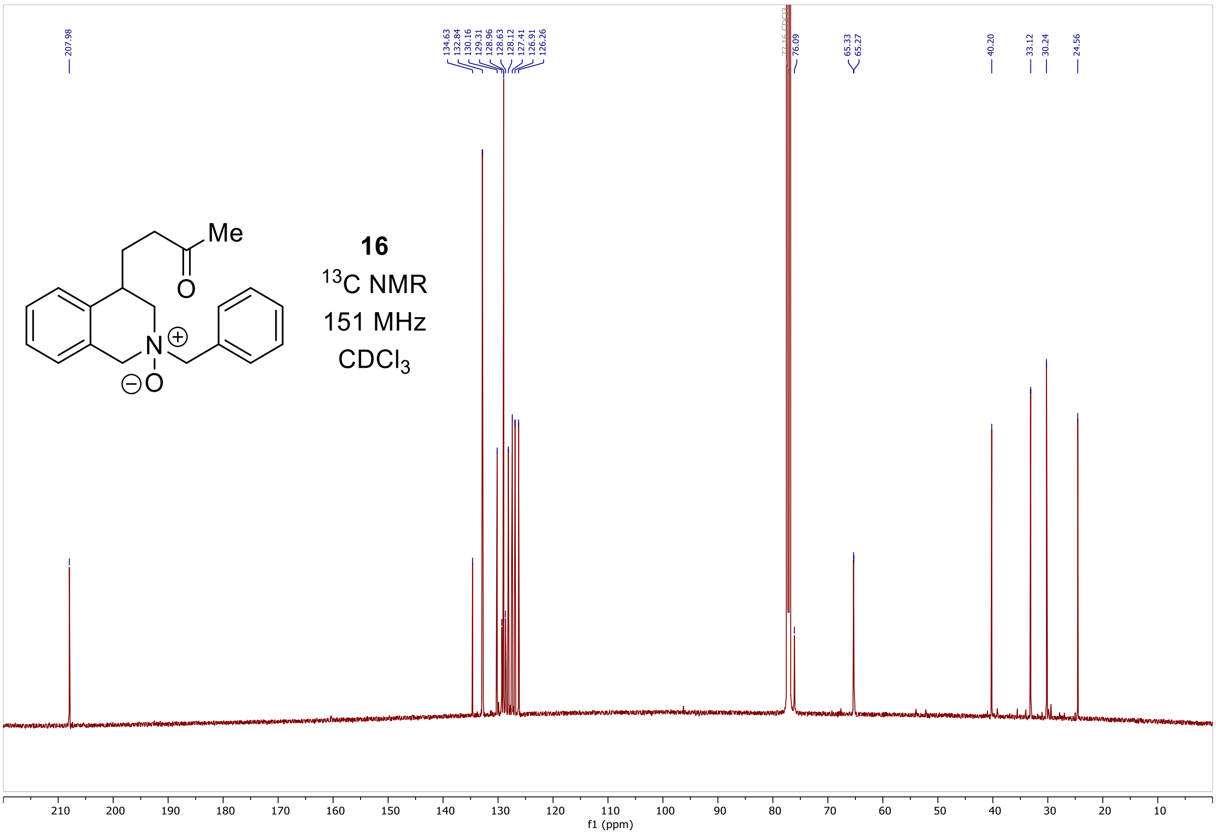
16
13C NMR
151 MHz
CDCl3
Me
O
N
O
207.98
134.63
132.84
130.16
129.31
128.96
128.63
128.12
127.41
126.91
126.26
76.09
65.33
65.27
40.20
33.12
30.24
24.56
f1 (ppm)

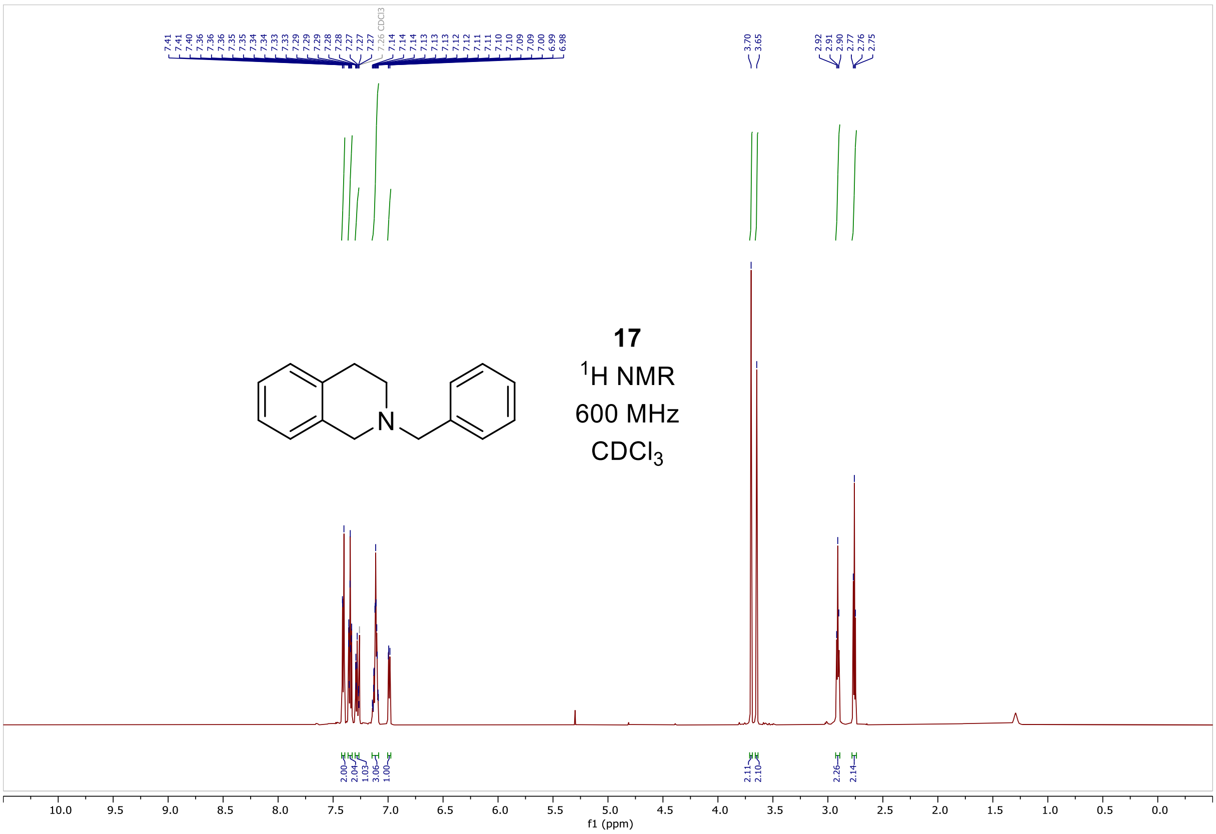
7.41 7.41 7.40 7.36 7.36 7.35 7.35 7.34 7.34 7.33 7.33 7.29 7.29 7.29 7.28 7.28 7.27 7.27 7.26 CDCl3 7.14 7.14 7.14 7.13 7.13 7.13 7.12 7.12 7.11 7.11 7.10 7.10 7.09 7.09 7.00 6.99 6.98
3.70 3.65
2.92 2.91 2.90 2.77 2.76 2.75
N
17
^{1}H NMR
600 MHz
$CDCl_3$
2.00 2.04 1.03 3.06 1.00
2.11 2.10
2.26 2.14
10.0 9.5 9.0 8.5 8.0 7.5 7.0 6.5 6.0 5.5 5.0 4.5 4.0 3.5 3.0 2.5 2.0 1.5 1.0 0.5 0.0
f1 (ppm)

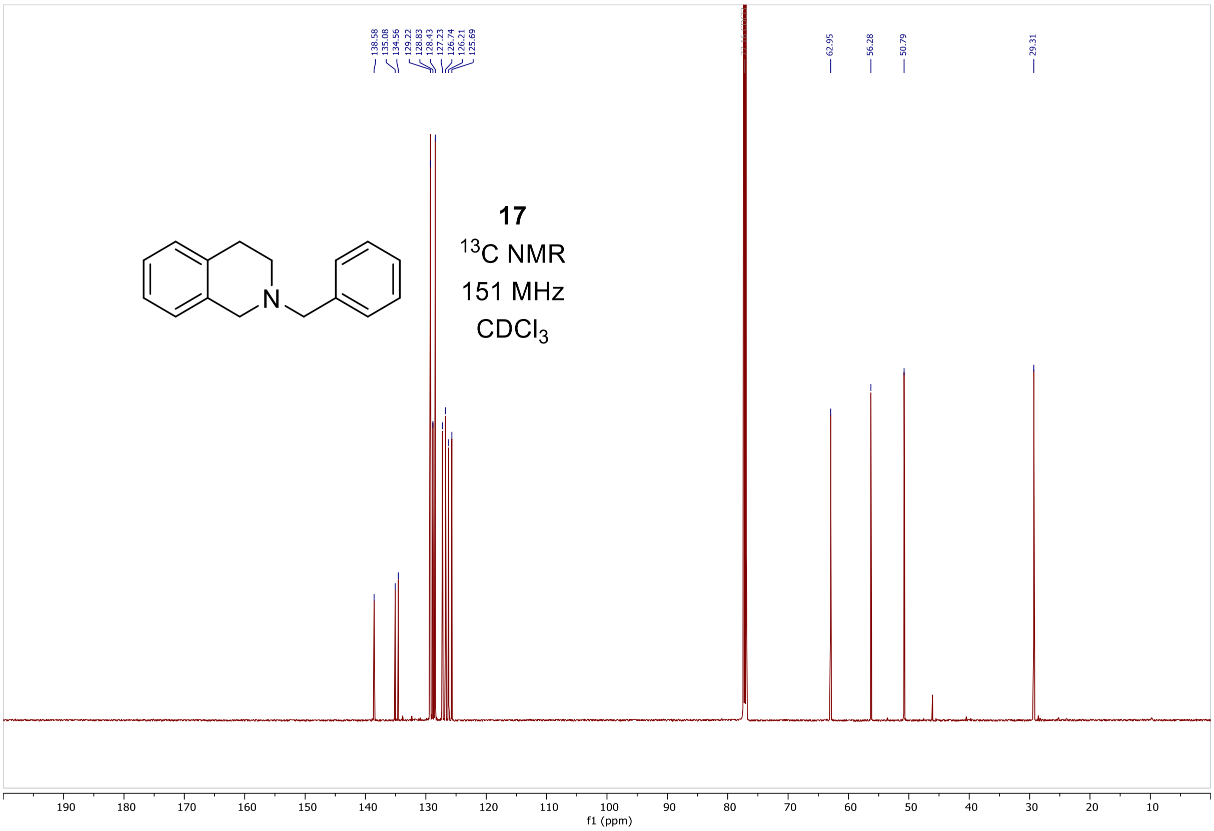
138.58 135.08 134.56 129.22 128.83 128.43 127.23 126.74 126.21 125.69
62.95
56.28
50.79
29.31
N
17
^{13}C NMR
151 MHz
$CDCl_3$
190 180 170 160 150 140 130 120 110 100 90 80 70 60 50 40 30 20 10
f1 (ppm)

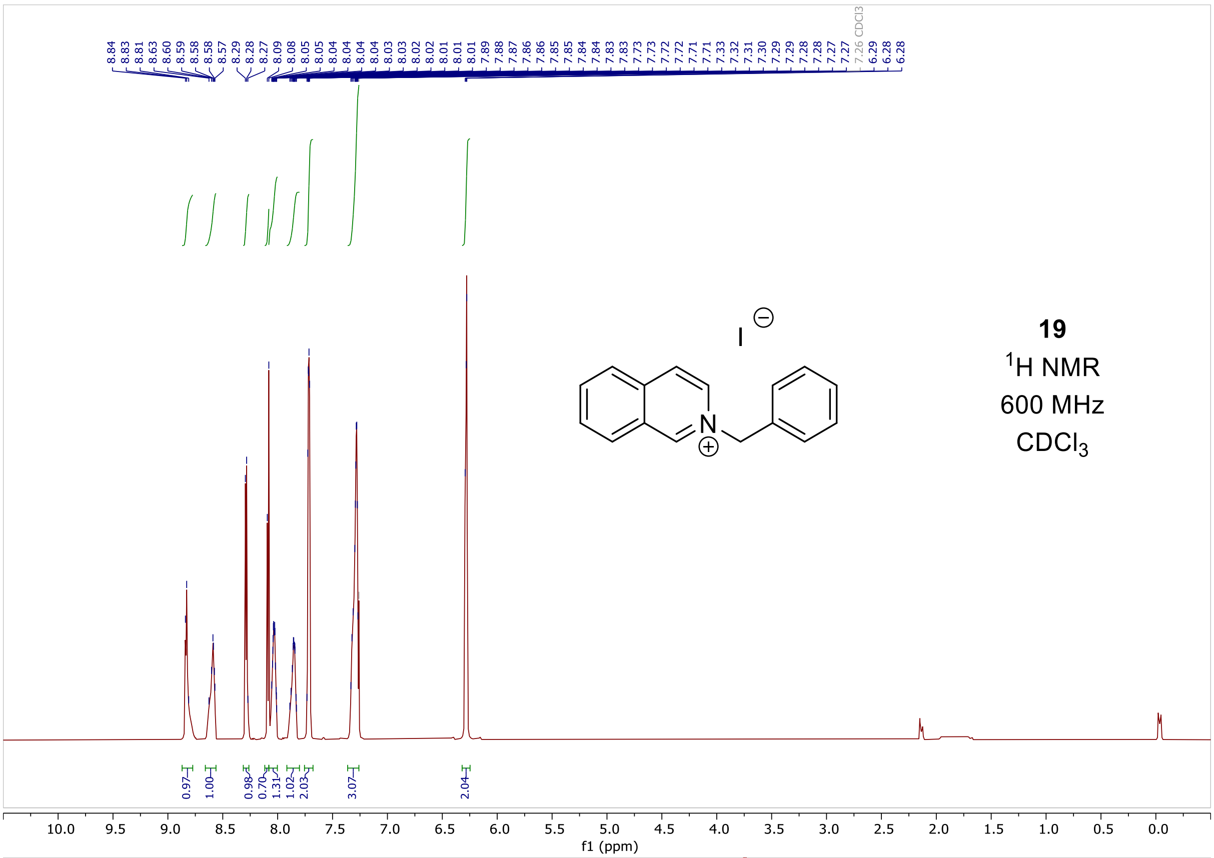
8.84 8.83 8.81 8.63 8.60 8.59 8.58 8.58 8.57 8.29 8.28 8.27 8.09 8.08 8.05 8.05 8.04 8.04 8.04 8.04 8.03 8.03 8.02 8.02 8.01 8.01 8.01 7.89 7.88 7.87 7.86 7.86 7.85 7.85 7.84 7.84 7.83 7.83 7.73 7.73 7.72 7.72 7.71 7.71 7.33 7.32 7.31 7.30 7.29 7.29 7.28 7.28 7.27 7.27 7.26 CDCl3 6.29 6.28 6.28
0.97 1.00 0.98 0.70 1.31 1.02 2.03 3.07 2.04
I
N
19
^{1}H NMR
600 MHz
$CDCl_3$
10.0 9.5 9.0 8.5 8.0 7.5 7.0 6.5 6.0 5.5 5.0 4.5 4.0 3.5 3.0 2.5 2.0 1.5 1.0 0.5 0.0
f1 (ppm)

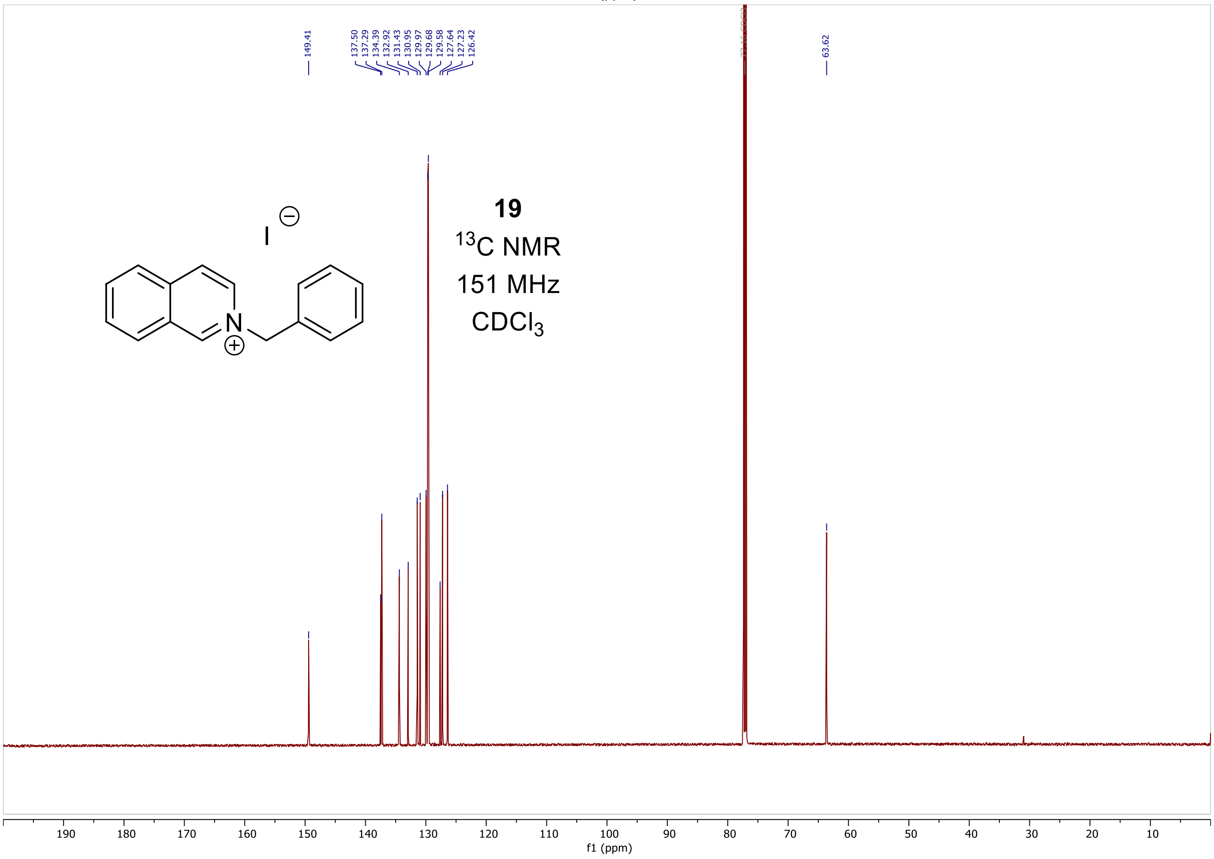
149.41
137.50 137.29 134.39 132.92 131.43 130.95 129.97 129.68 129.58 127.64 127.23 126.42
63.62
I
N
19
^{13}C NMR
151 MHz
$CDCl_3$
190 180 170 160 150 140 130 120 110 100 90 80 70 60 50 40 30 20 10
f1 (ppm)

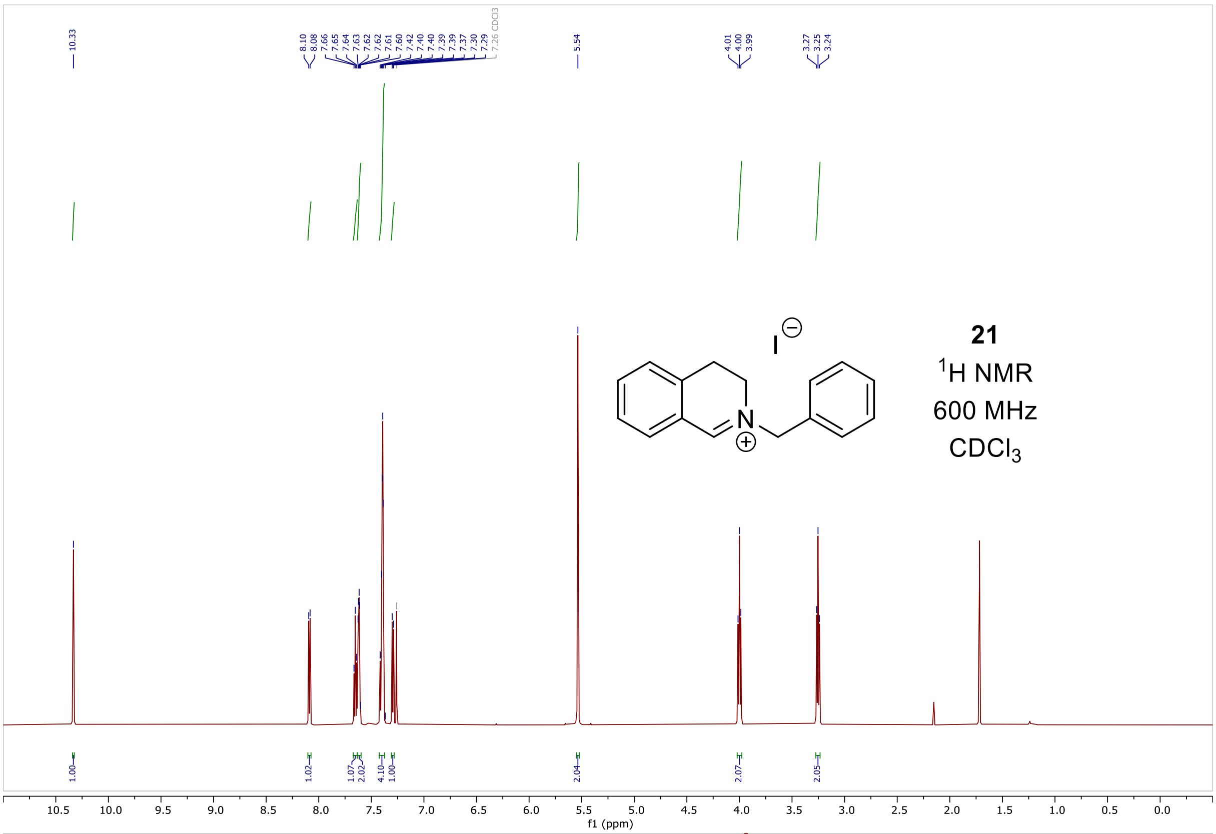

10.33
8.10
8.08
7.66
7.65
7.64
7.63
7.62
7.62
7.61
7.60
7.42
7.40
7.40
7.39
7.39
7.37
7.30
7.29
7.26 CDCl3
5.54
4.01
4.00
3.99
3.27
3.25
3.24
21
^{1}H NMR
600 MHz
$CDCl_3$
1.00
1.02
1.07
2.02
4.10
1.00
2.04
2.07
2.05
f1 (ppm)

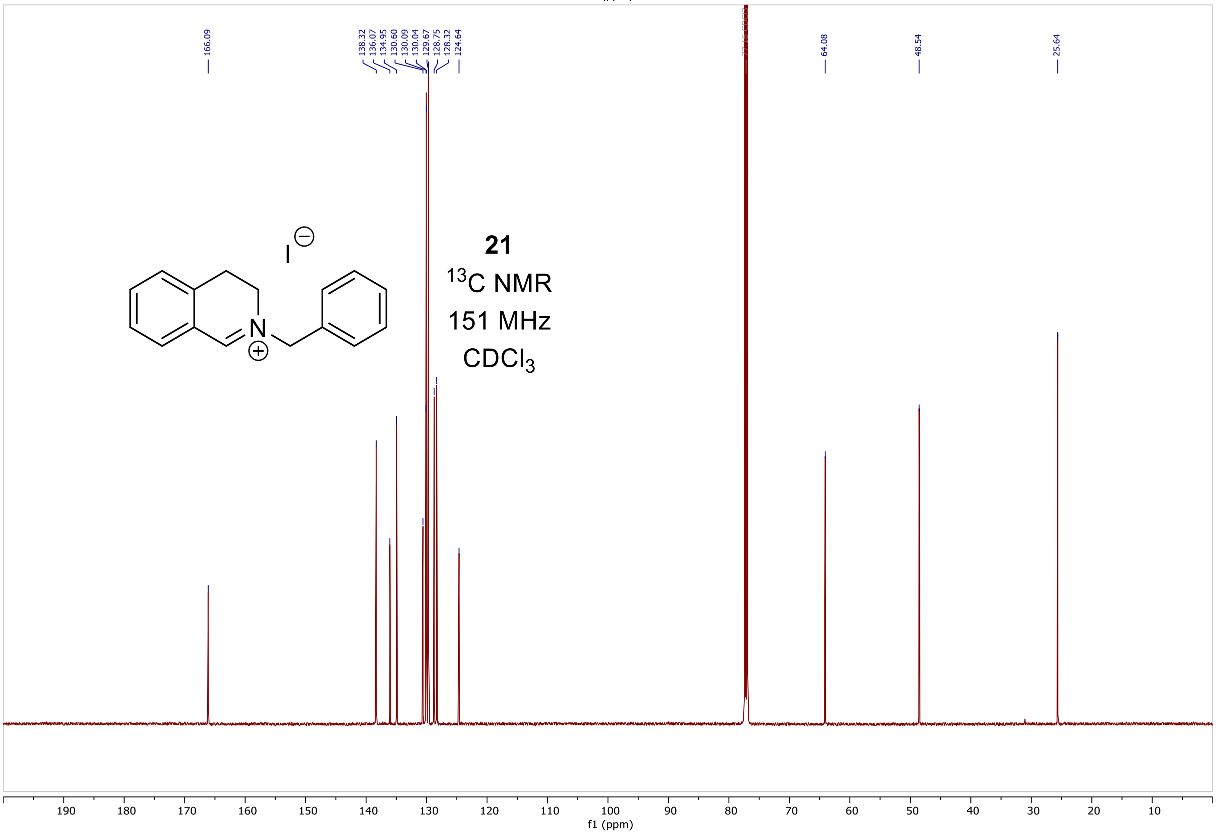

166.09
138.32
136.07
134.95
130.60
130.09
130.04
129.67
128.75
128.32
124.64
64.08
48.54
25.64
21
^{13}C NMR
151 MHz
$CDCl_3$
f1 (ppm)

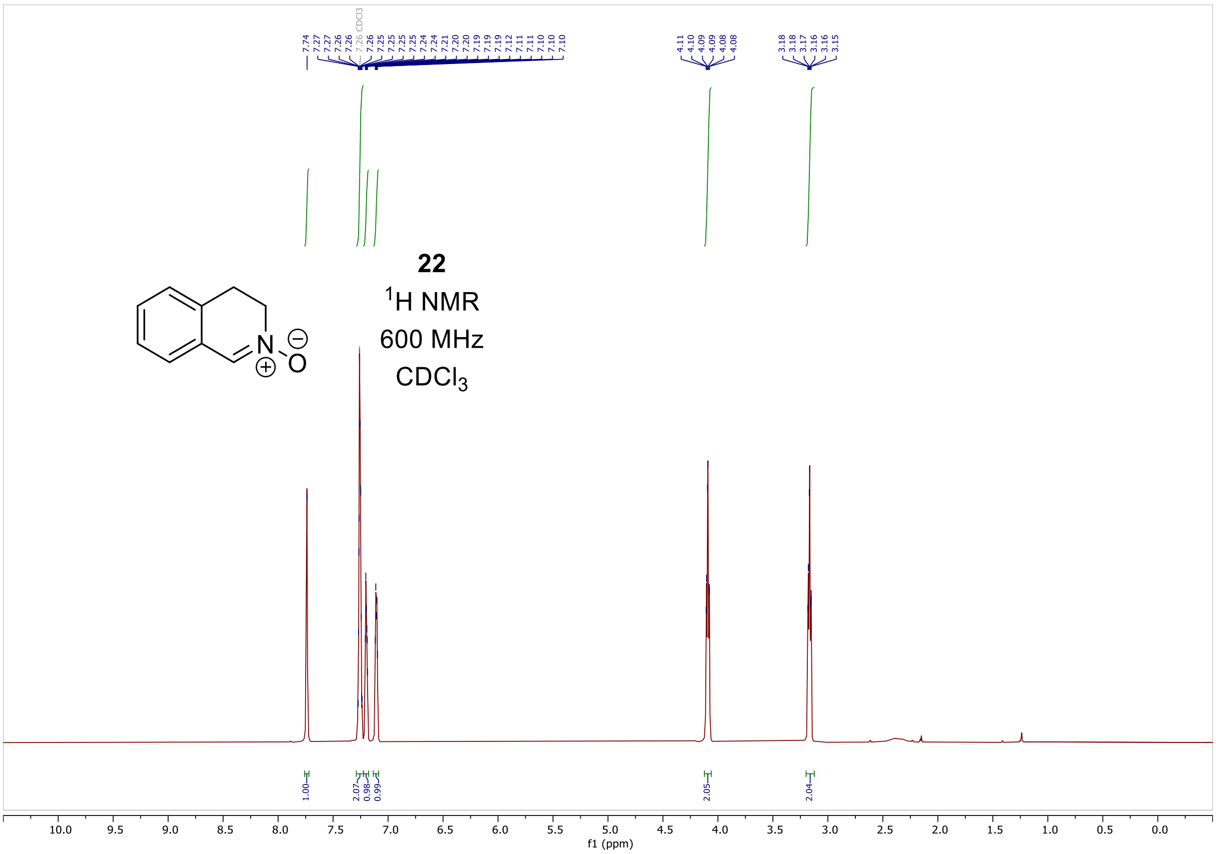
7.74
7.27
7.27
7.26
7.26
7.26 CDCl3
7.26
7.25
7.25
7.25
7.25
7.24
7.24
7.21
7.20
7.20
7.19
7.19
7.19
7.12
7.11
7.11
7.10
7.10
7.10
4.11
4.10
4.09
4.09
4.08
4.08
3.18
3.18
3.17
3.16
3.16
3.15
22
^{1}H NMR
600 MHz
CDCl$_3$
1.00
2.07
0.98
0.99
2.05
2.04
10.0
9.5
9.0
8.5
8.0
7.5
7.0
6.5
6.0
5.5
5.0
4.5
4.0
3.5
3.0
2.5
2.0
1.5
1.0
0.5
0.0
f1 (ppm)

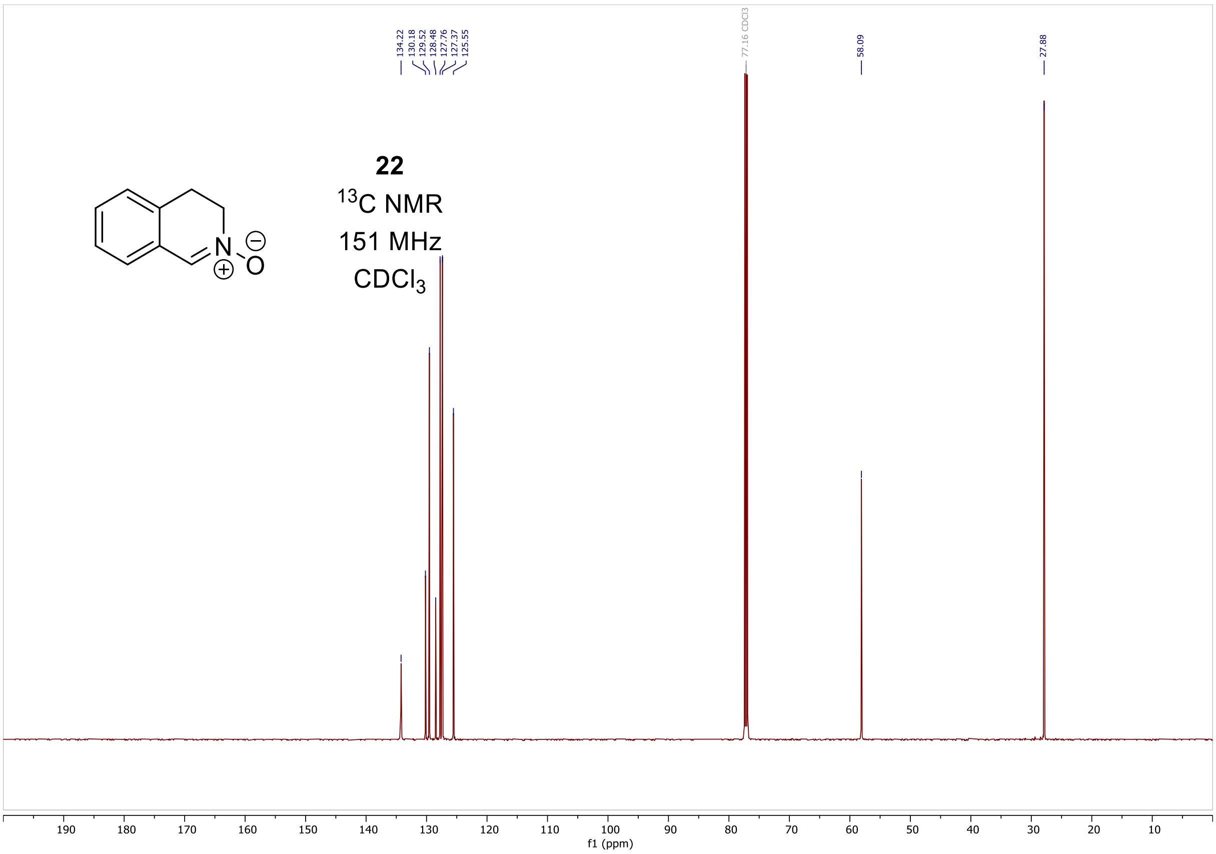
134.22
130.18
129.52
128.48
127.76
127.27
125.55
77.16 CDCl3
58.09
27.88
22
^{13}C NMR
151 MHz
CDCl$_3$
190
180
170
160
150
140
130
120
110
100
90
80
70
60
50
40
30
20
10
f1 (ppm)

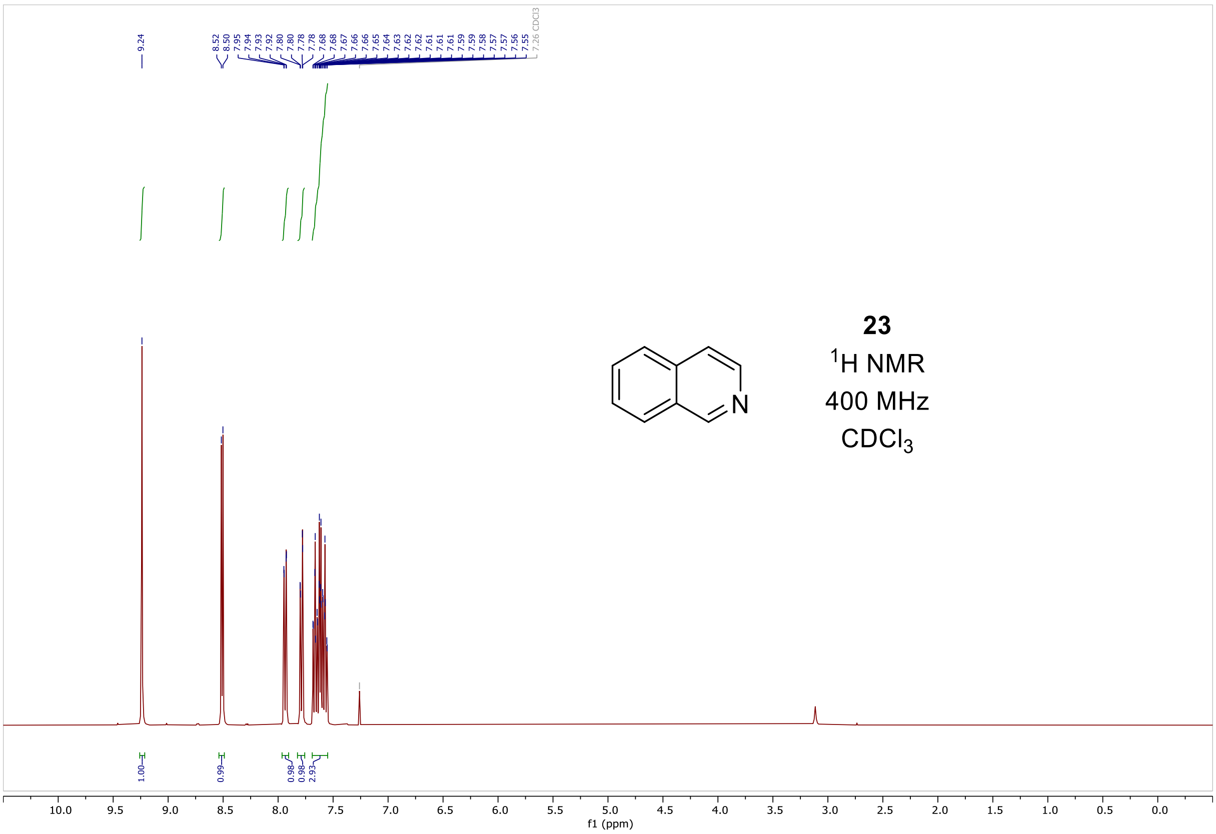
23
1H NMR
400 MHz
CDCl3
f1 (ppm)

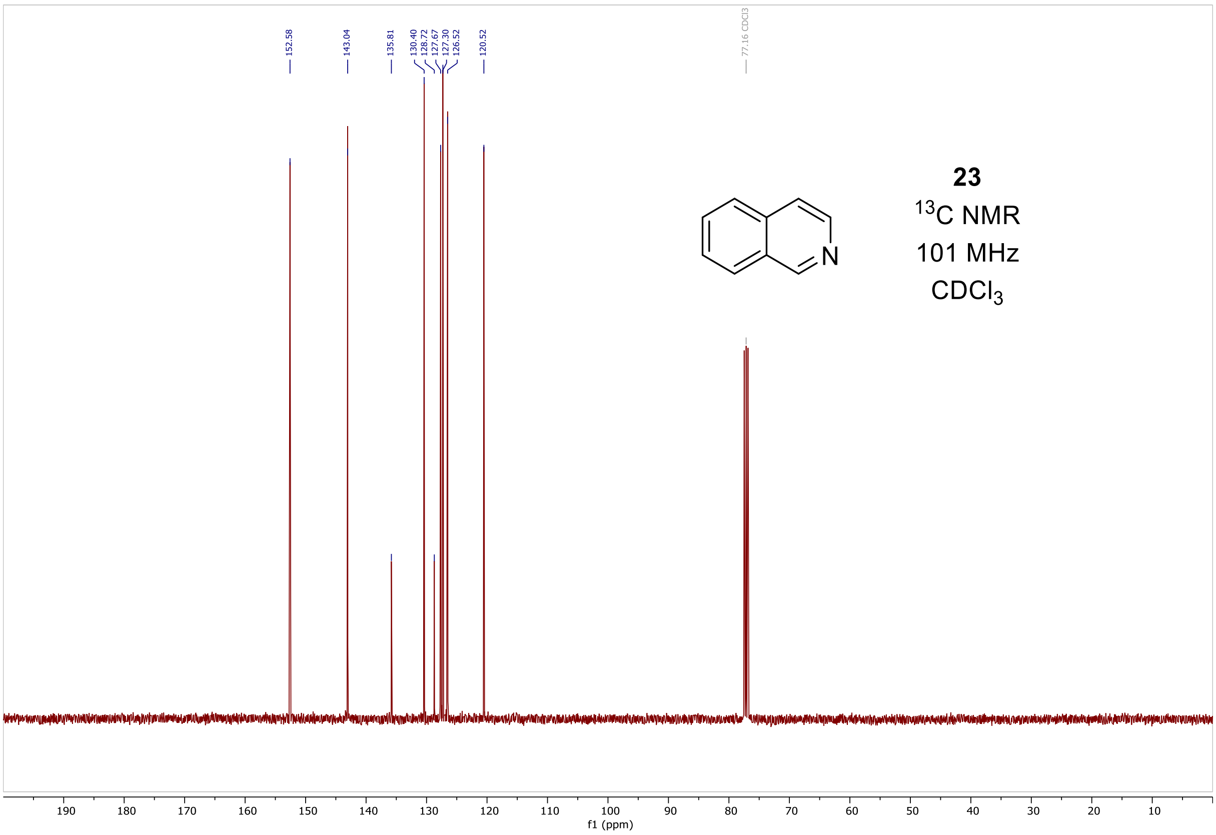
23
13C NMR
101 MHz
CDCl3
f1 (ppm)

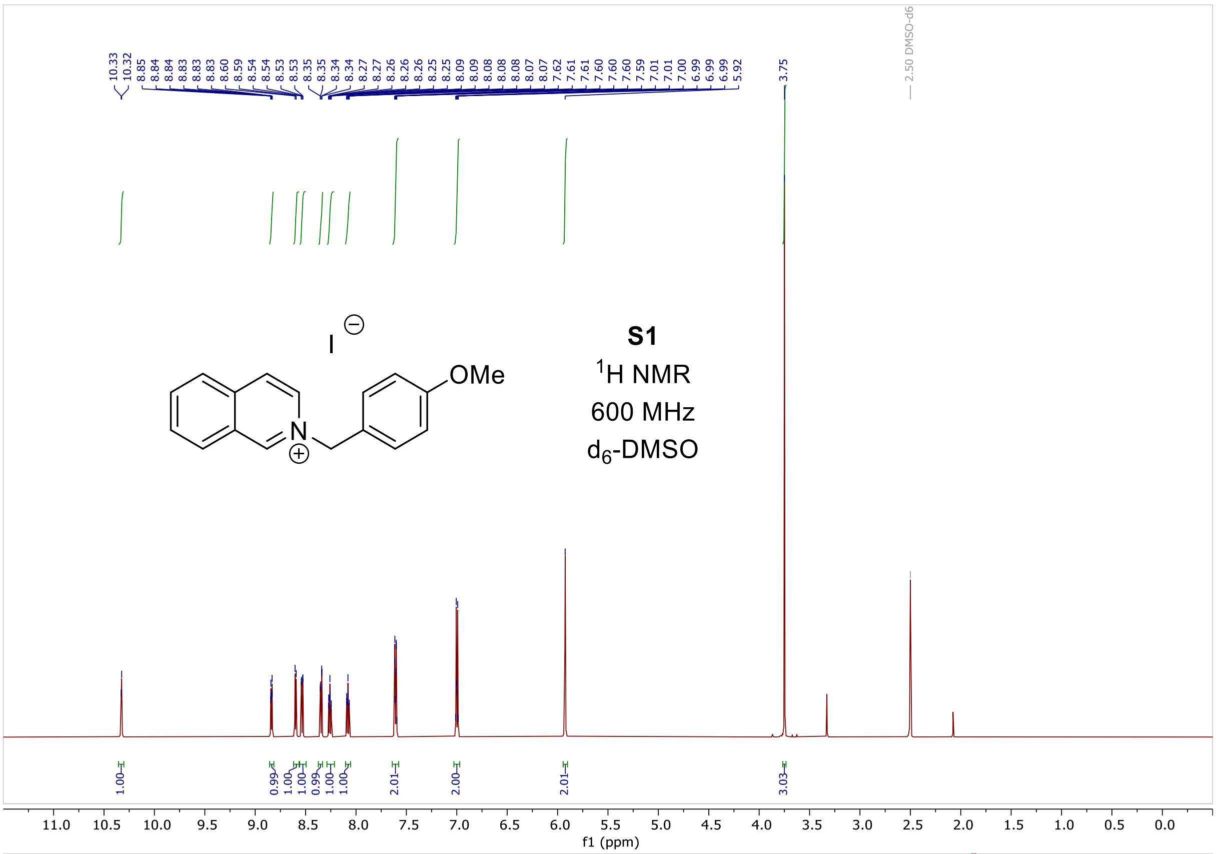
10.33
10.32
8.85
8.84
8.84
8.83
8.83
8.83
8.60
8.59
8.54
8.54
8.53
8.53
8.35
8.35
8.34
8.34
8.27
8.27
8.26
8.26
8.26
8.25
8.09
8.09
8.08
8.08
8.08
8.07
8.07
7.62
7.61
7.61
7.60
7.60
7.59
7.01
7.01
7.00
6.99
6.99
6.99
5.92
3.75
2.50 DMSO-d6
I
OMe
N
S1
1H NMR
600 MHz
d6-DMSO
1.00
0.99
1.00
1.00
0.99
1.00
1.00
2.01
2.00
2.01
3.03
f1 (ppm)

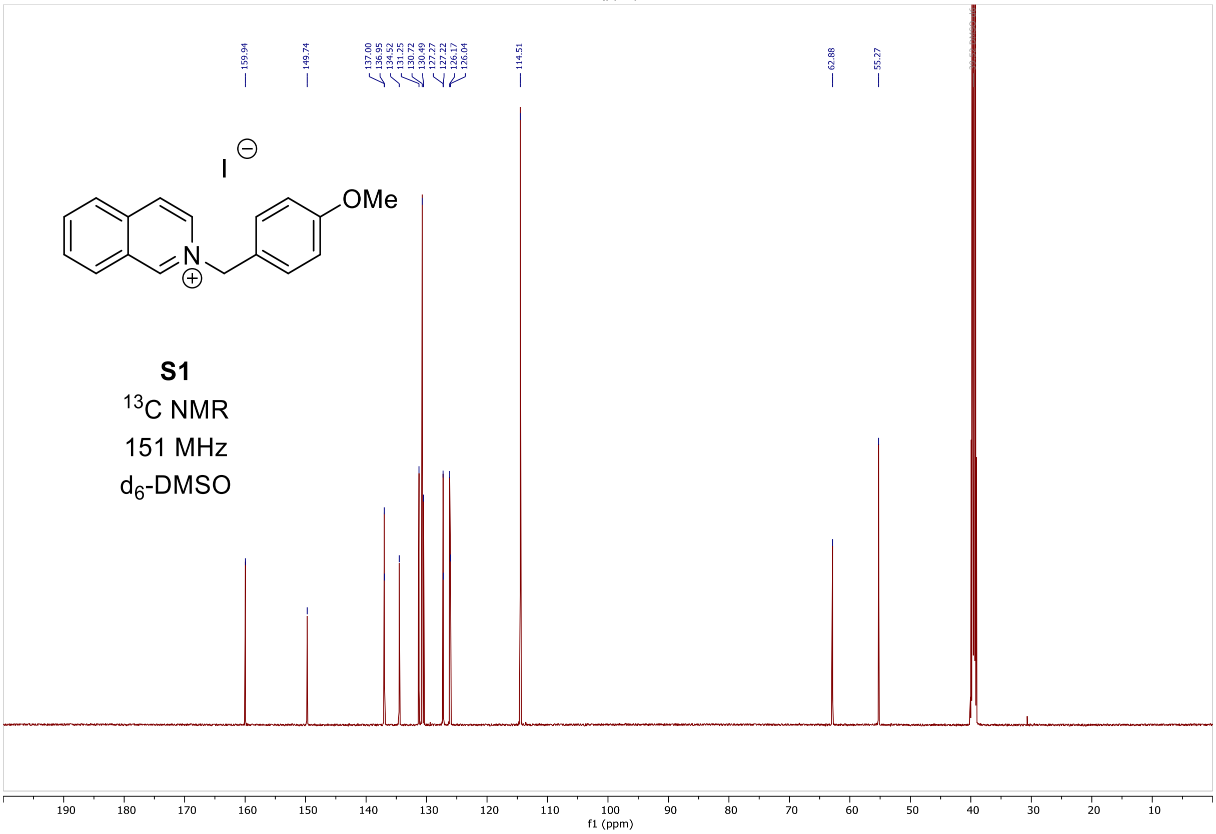
159.94
149.74
137.00
136.95
134.52
131.25
130.72
130.49
127.27
127.22
126.17
126.04
114.51
62.88
55.27
I
OMe
N
S1
13C NMR
151 MHz
d6-DMSO
f1 (ppm)

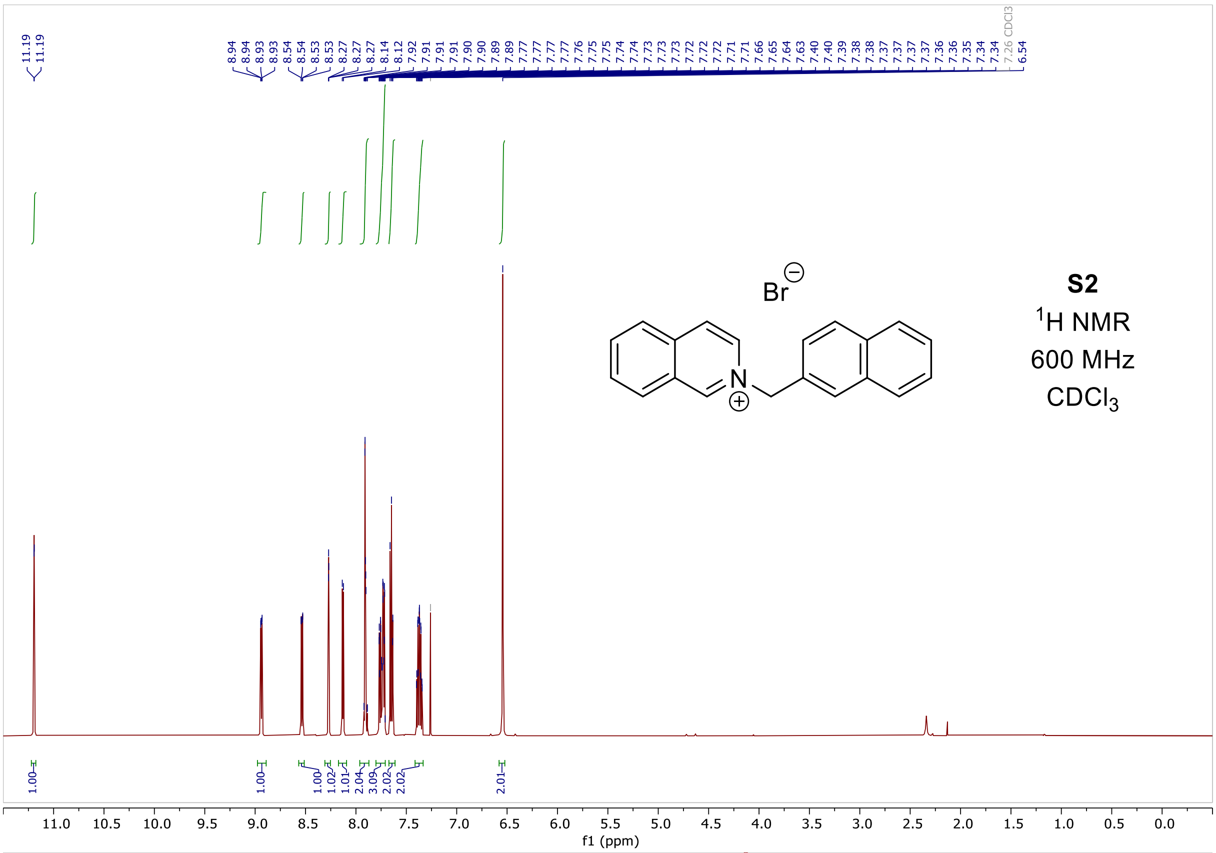
S2
^{1}H NMR
600 MHz
$CDCl_3$
f1 (ppm)

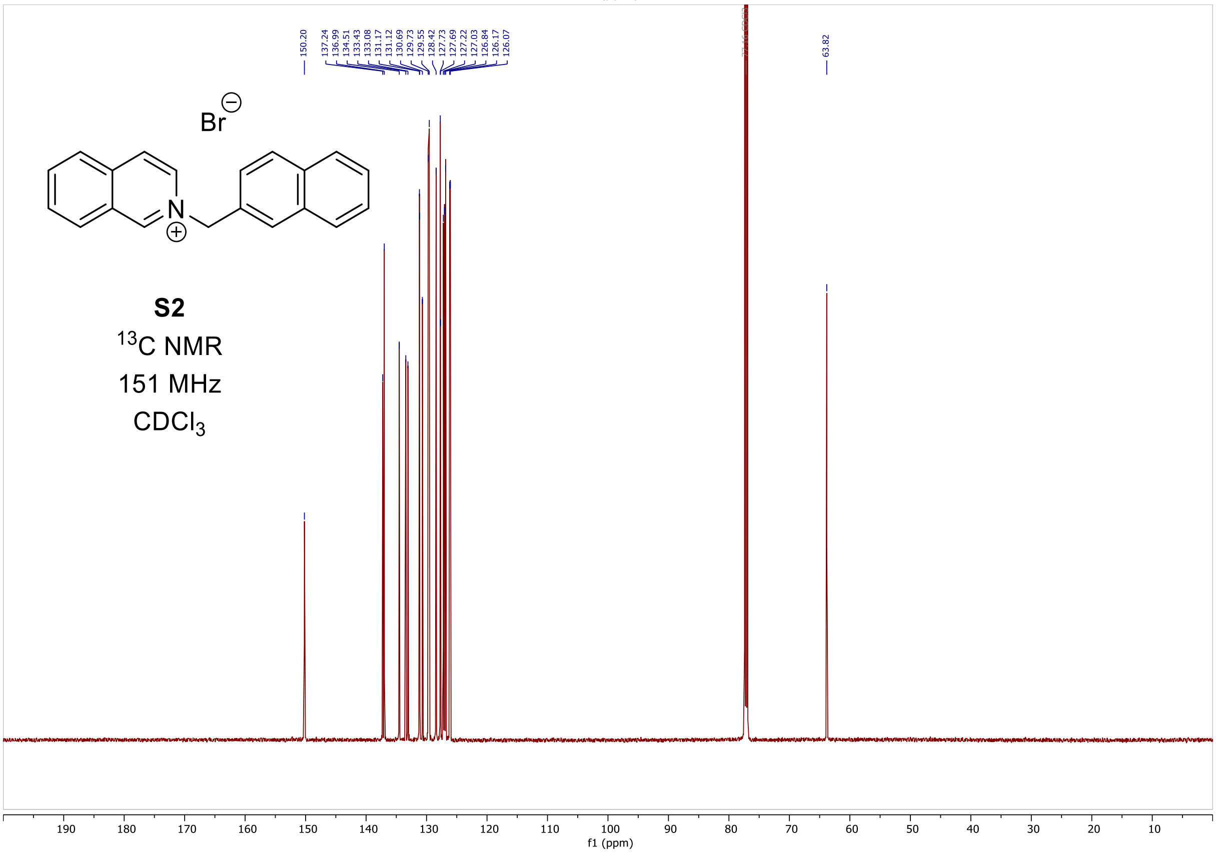
S2
^{13}C NMR
151 MHz
$CDCl_3$
f1 (ppm)

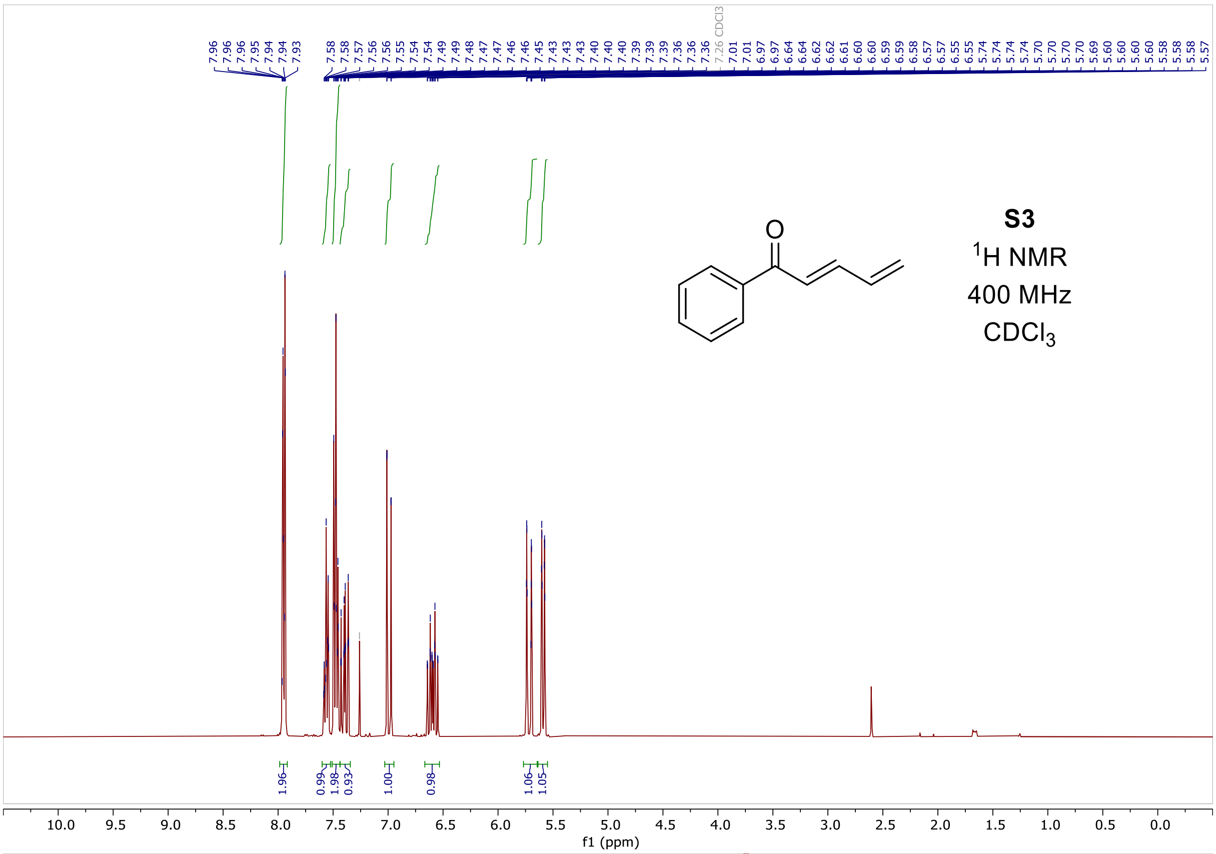

S3
1H NMR
400 MHz
CDCl3
O
1.96
0.99
1.98
0.93
1.00
0.98
1.06
1.05
f1 (ppm)

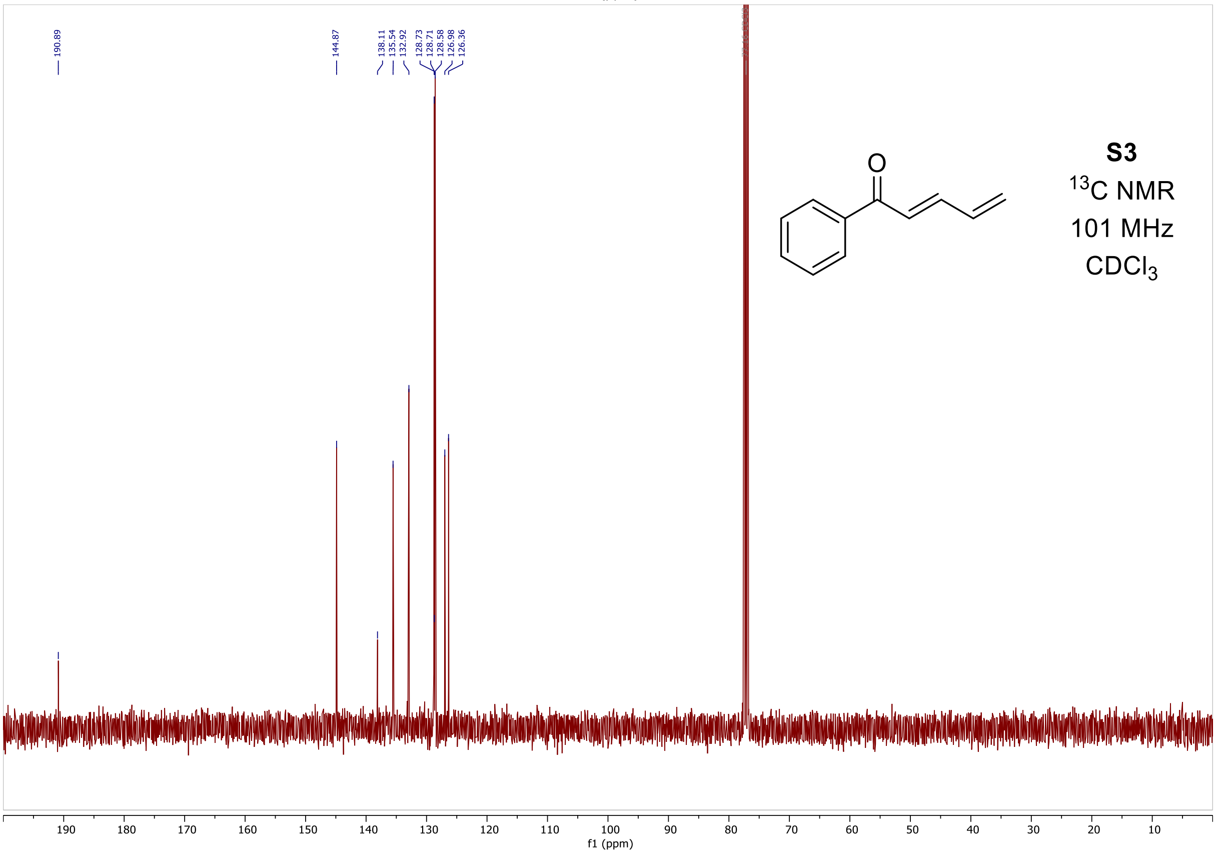

190.89
144.87
138.11
135.54
132.92
128.73
128.71
128.58
126.98
126.36
S3
13C NMR
101 MHz
CDCl3
O
f1 (ppm)

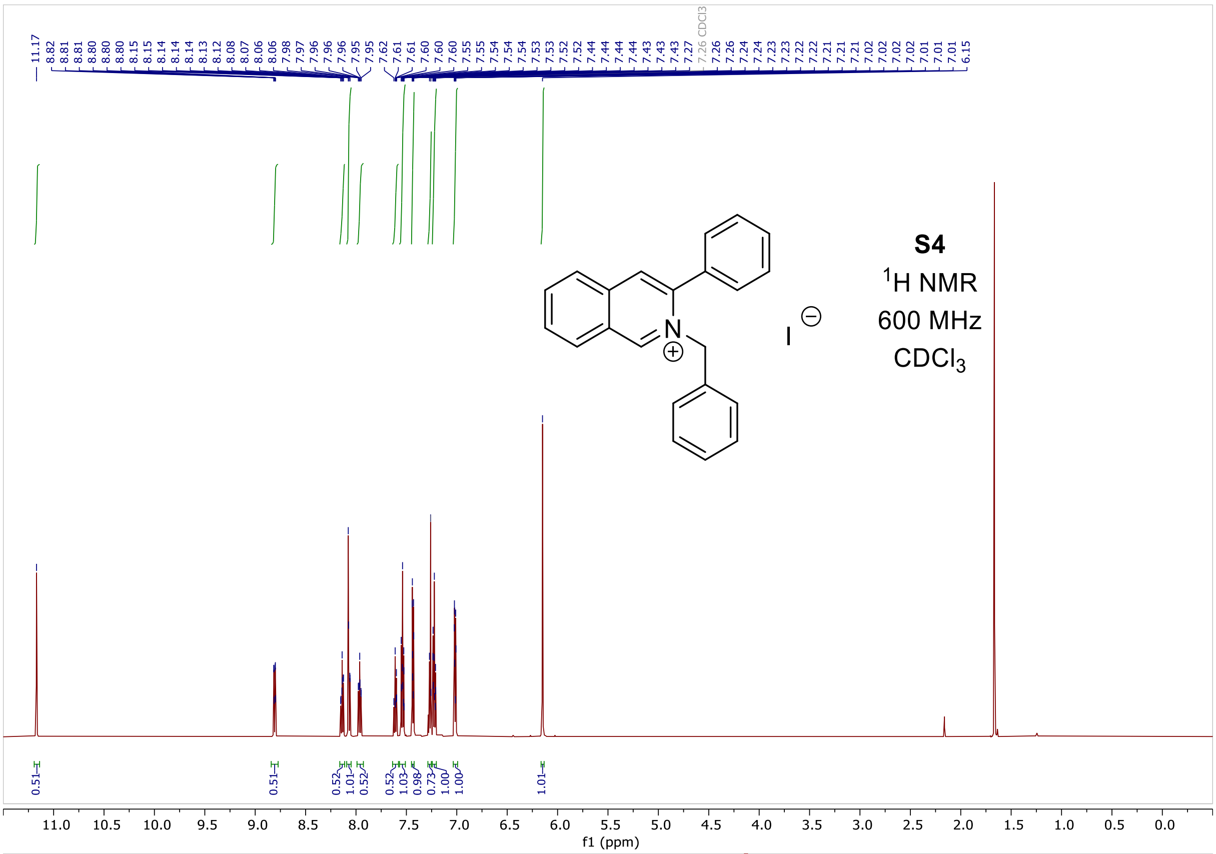
S4
^{1}H NMR
600 MHz
$CDCl_3$
f1 (ppm)

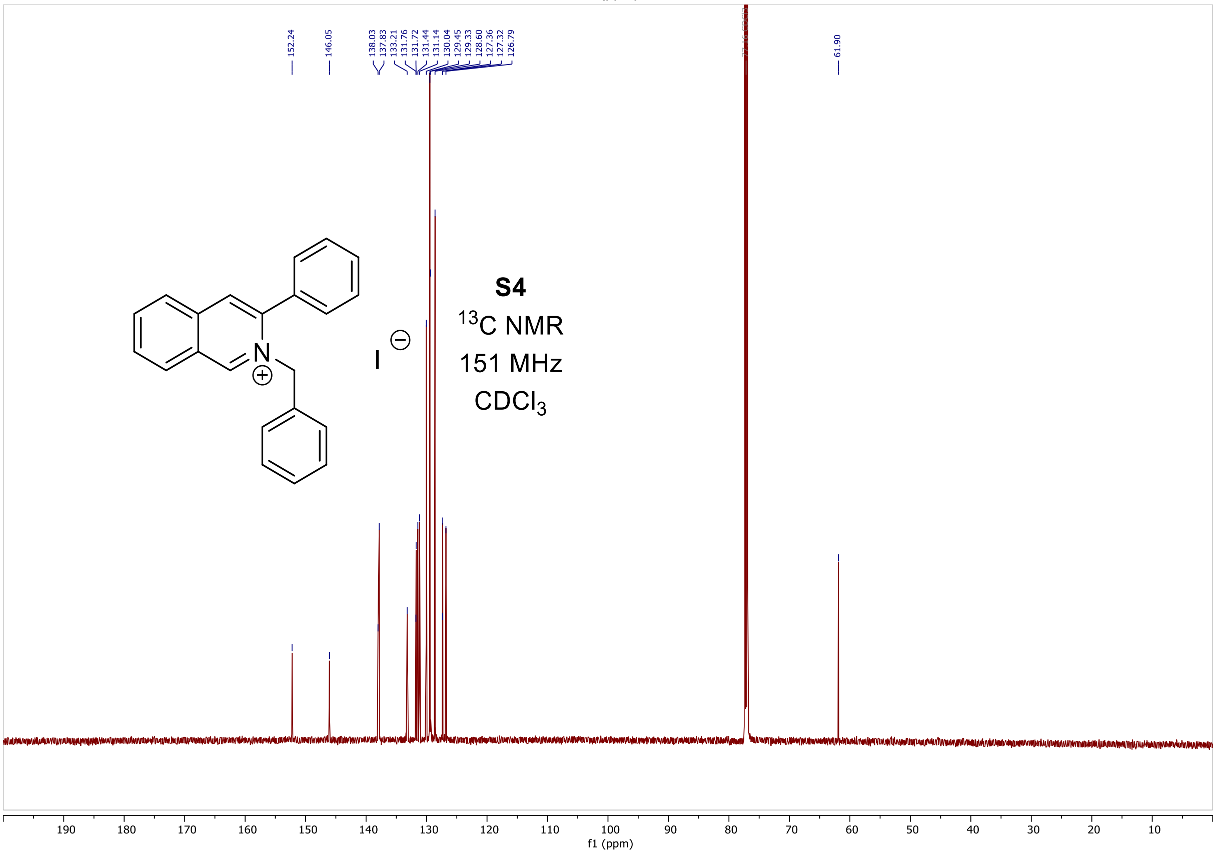
S4
^{13}C NMR
151 MHz
$CDCl_3$
f1 (ppm)

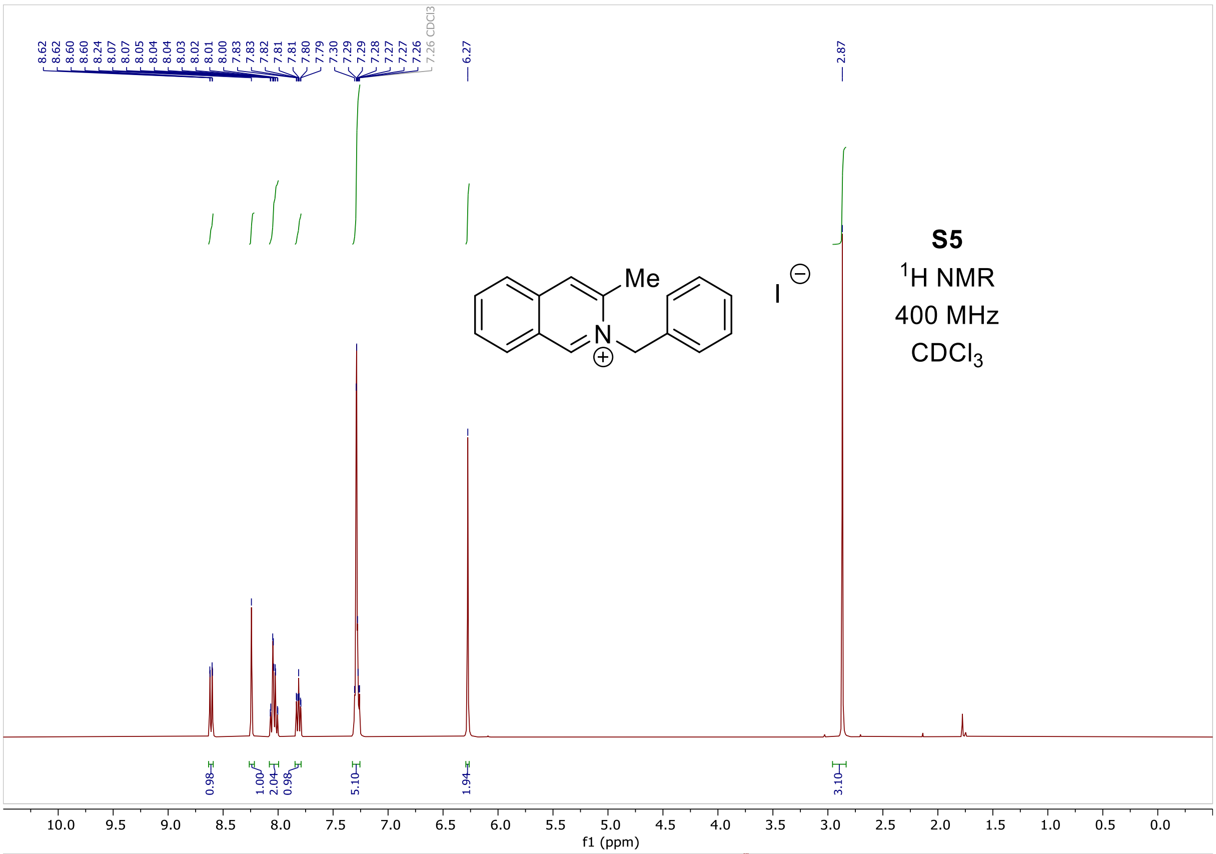
8.62
8.62
8.60
8.60
8.24
8.07
8.07
8.05
8.05
8.04
8.04
8.03
8.02
8.01
8.00
7.83
7.83
7.82
7.81
7.81
7.80
7.79
7.30
7.29
7.29
7.28
7.27
7.27
7.26
7.26 CDCl3
6.27
2.87
Me
I
N
S5
1H NMR
400 MHz
CDCl3
0.98
1.00
2.04
0.98
5.10
1.94
3.10
f1 (ppm)

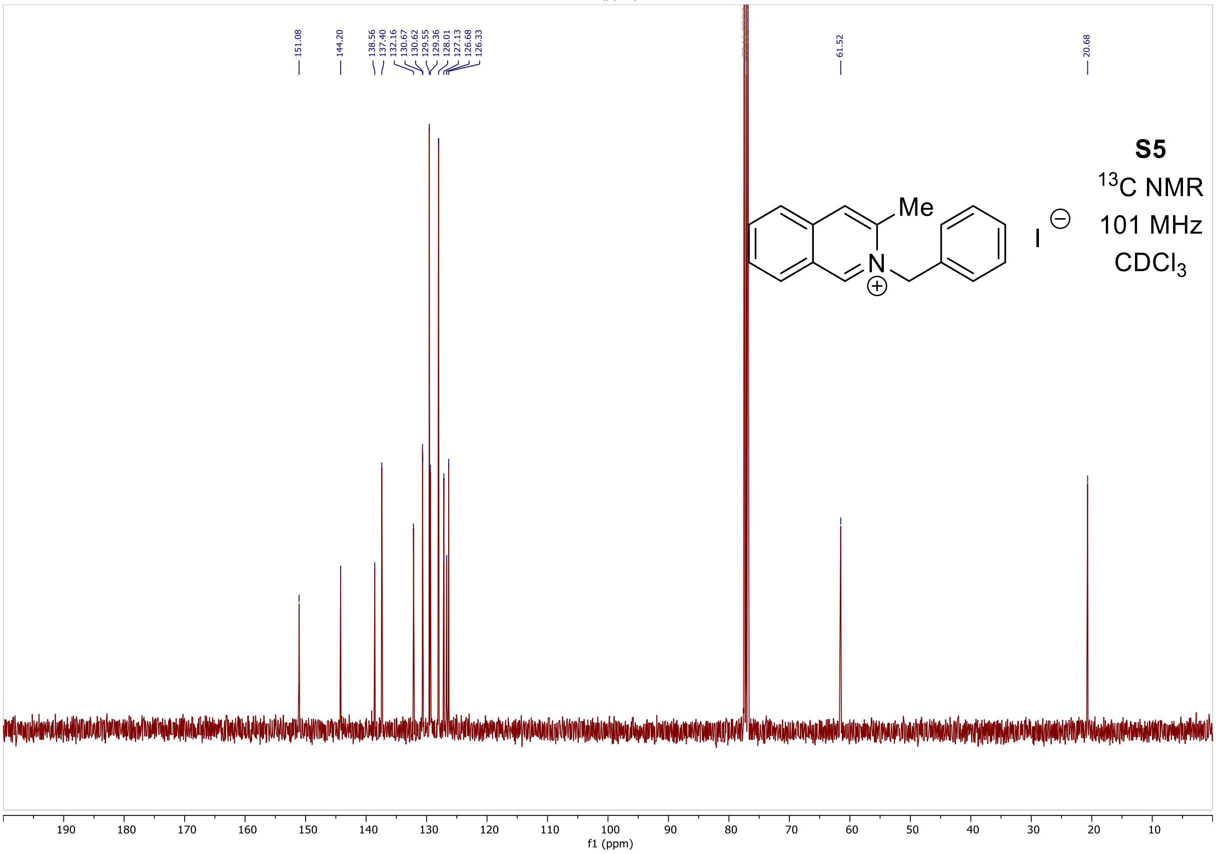
151.08
144.20
138.56
137.40
132.16
130.67
130.62
129.55
129.36
128.01
127.13
126.68
126.33
61.52
20.68
Me
I
N
S5
13C NMR
101 MHz
CDCl3
f1 (ppm)

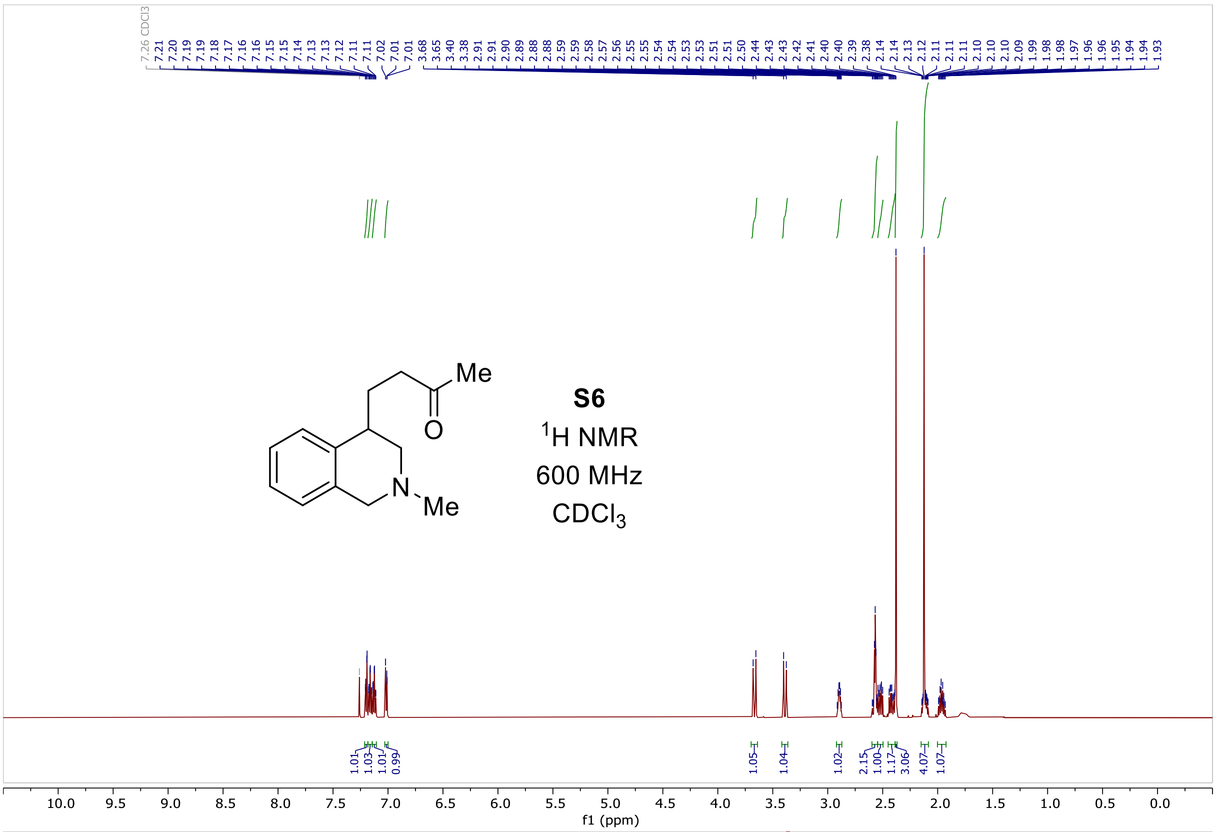
S6
¹H NMR
600 MHz
CDCl₃
Me
O
N
Me
f1 (ppm)

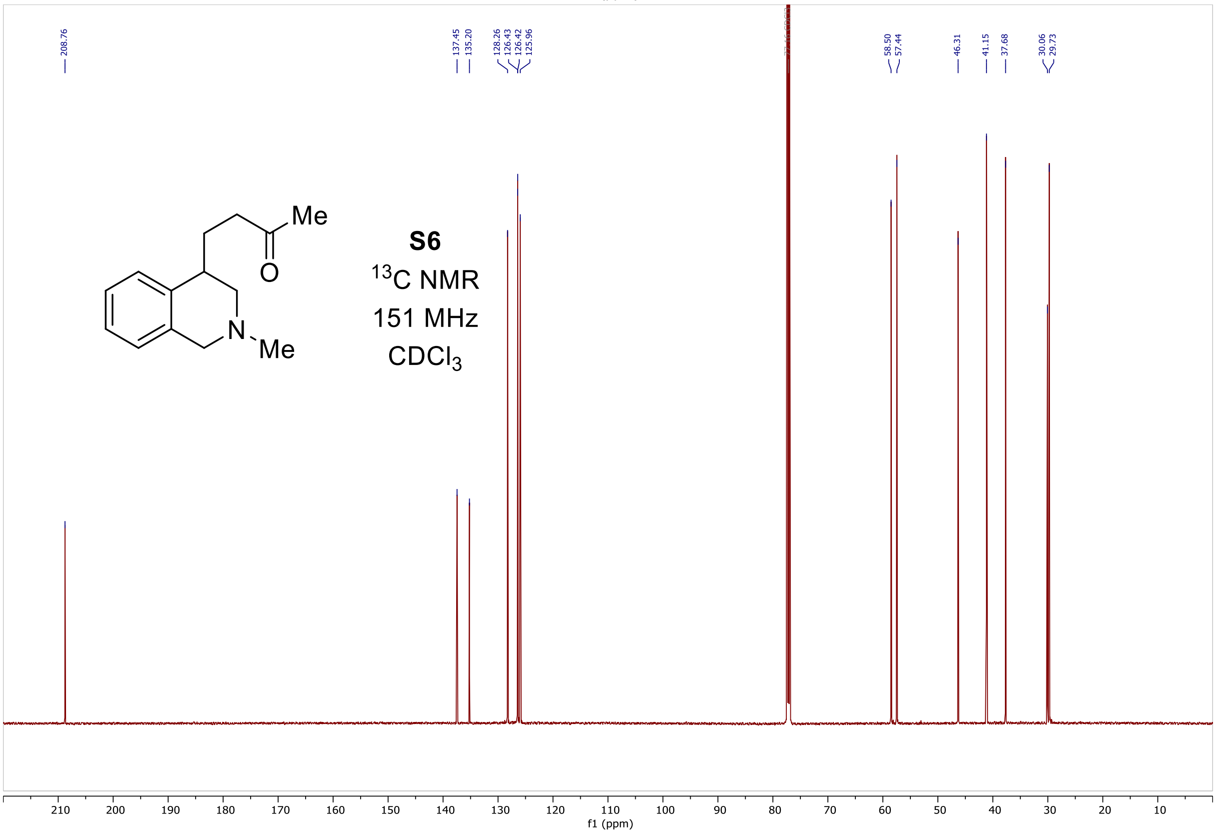
S6
¹³C NMR
151 MHz
CDCl₃
Me
O
N
Me
208.76
137.45
135.20
128.26
126.43
126.42
125.96
58.50
57.44
46.31
41.15
37.68
30.06
29.73
f1 (ppm)

5. Single Crystal X-ray Data

2,4-Dibenzyl-1,2,3,4-tetrahydroisoquinoline (3e):

Recrystallised from acetone by slow evaporation. 3e (CCDC number 2347994) is a previously reported structure, see- sp3 C–H Bond Activation with Ruthenium(II) Catalysts and C(3)-Alkylation of Cyclic Amines, Sundararaju, B.; Achard, M.; Sharma, G. V. M.; Bruneau, C. *J. Am. Chem. Soc*. **2011**, *133*, 10340-10343.

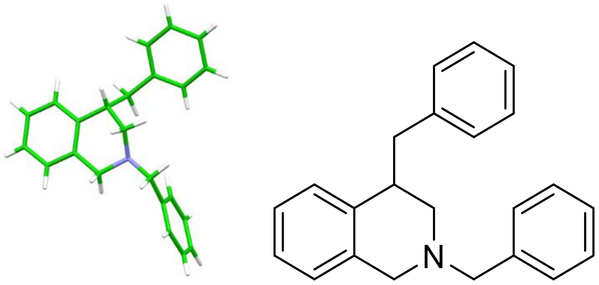

Formula C23 H23 N1

Crystal Class	orthorhombic	Space Group	P c c n
a	24.1315(3)	alpha	90
b	20.9517(2)	beta	90
c	6.92850(10)	gamma	90
Volume	3503.02(7)	Z	8
Radiation type	Cu K\a	Wavelength	1.541840
Dx	1.19	Mr	313.44
Mu	0.515	Temperature (K)	150
Size	0.20x 0.20x 0.20		
Colour	clear pale colourless	Shape	prism
Cell from	18114 Reflections	Theta range	4 to 76
Diffractometer type	multi-scan	Scan type	OMEGA
Absorption type	multi-scan	Transmission range	0.83 0.90
Reflections measured	36816	Independent reflections	3669
Rint	0.0004	Theta max	76.34
Hmin, Hmax	-30 30		
Kmin, Kmax	-25 18		
Lmin, Lmax	-8 8		
Refinement on Fsqd			
R-factor	0.041	Weighted R-factor	0.100
Max shift/su	0.0127		
Delta Rho min	-0.22	Delta Rho max	0.26

Reflections used	3668	sigma(I) limit	-3.00
Number of parameters	309	Goodness of fit	0.962

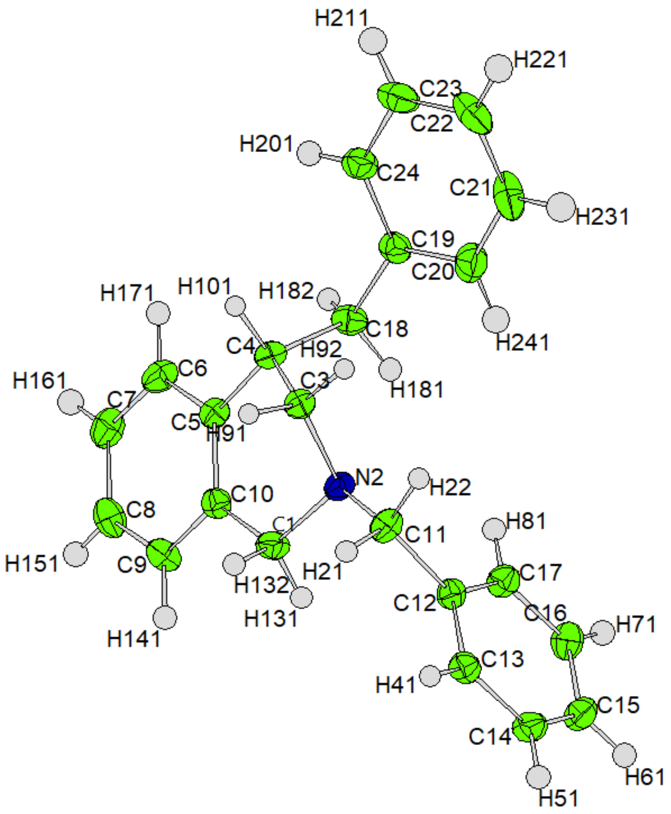

Thermal ellipsoids are drawn at the 50% probability level.

4-Benzylisoquinoline (4e):

Recrystallised from acetone by slow evaporation

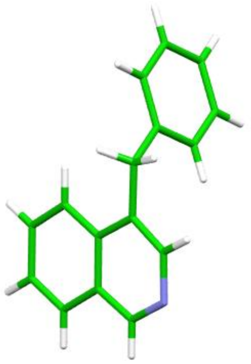

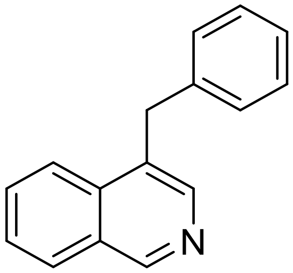

Formula C16 H13 N1

Crystal Class monoclinic Space Group P 21/n

a 6.4852(2) alpha 90

b 8.1394(2) beta 92.424(3)

c 21.9972(7) gamma 90

Volume 1160.10(6) Z 4

Radiation type Cu K\a Wavelength 1.541840

Dx 1.26 Mr 219.29

Mu 0.559 Temperature (K) 150

Size 0.01x 0.20x 0.30

Colour clear light colourless Shape plate

Cell from 3514 Reflections Theta range 4 to 76

Diffractometer type multi-scan Scan type OMEGA

Absorption type multi-scan Transmission range 0.92 0.99

Reflections measured 5629 Independent reflections 2389

Rint 0.0002 Theta max 76.10

Hmin, Hmax -6 8

Kmin, Kmax -10 9

Lmin, Lmax -27 27

Refinement on Fsqd

R-factor 0.042 Weighted R-factor 0.104

Max shift/su 0.0104

Delta Rho min -0.26 Delta Rho max 0.21

Reflections used 2389 sigma(I) limit -3.00

Number of parameters 154 Goodness of fit 0.974

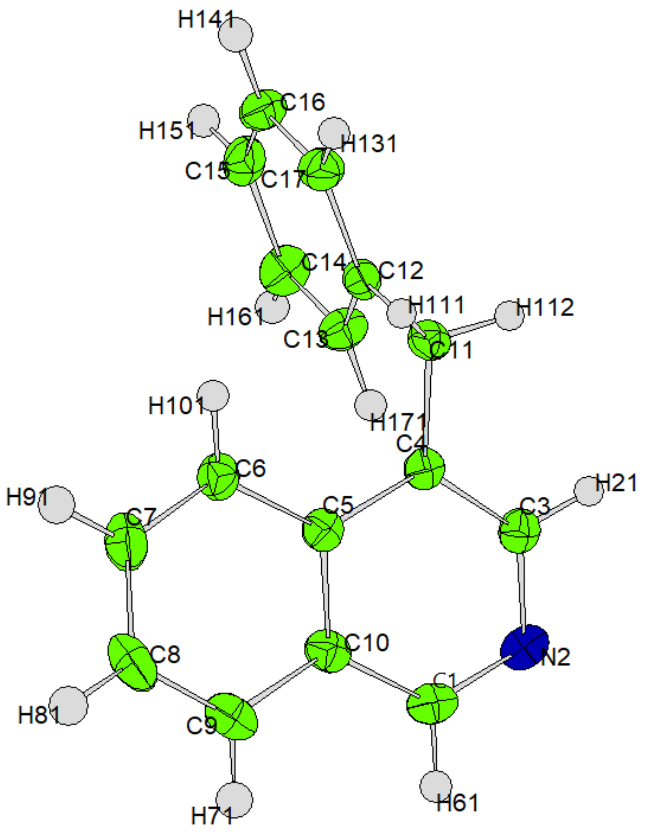

Thermal ellipsoids are drawn at the 50% probability level.

4-(Naphthalen-1-ylmethyl)isoquinoline (4h):

Recrystallised from acetone by slow evaporation

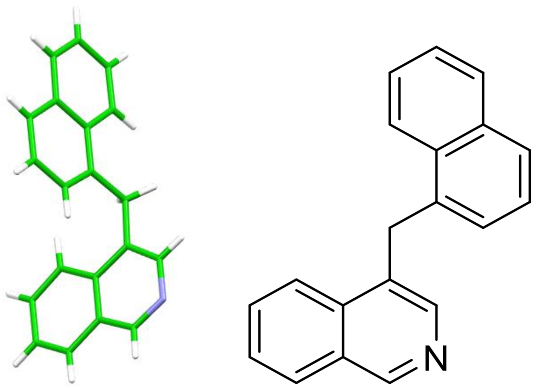

Formula C20 H15 N1			
Crystal Class	monoclinic	Space Group	P 21/n
a	15.7726(2)	alpha	90
b	8.89510(10)	beta	109.5290(11)
c	21.2757(2)	gamma	90
Volume	2813.24(6)	Z	8
Radiation type	Cu K\a	Wavelength	1.541840
Dx	1.27	Mr	538.69
Mu	0.563	Temperature (K)	150
Size	0.20x 0.20x 0.20		
Colour	clear colourless	Shape	shapeless
Cell from	32408 Reflections	Theta range	4 to 76
Reflections measured	71442	Independent reflections	5872
Rint	0.0004	Theta max	76.30
Hmin, Hmax	-17 19		
Kmin, Kmax	-11 11		
Lmin, Lmax	-26 26		
Refinement on Fsqd			
R-factor	0.038	Weighted R-factor	0.095
Max shift/su	0.0008		
Delta Rho min	-0.17	Delta Rho max	0.25
Reflections used	5872	sigma(I) limit	-3.00
Number of parameters	379	Goodness of fit	0.970

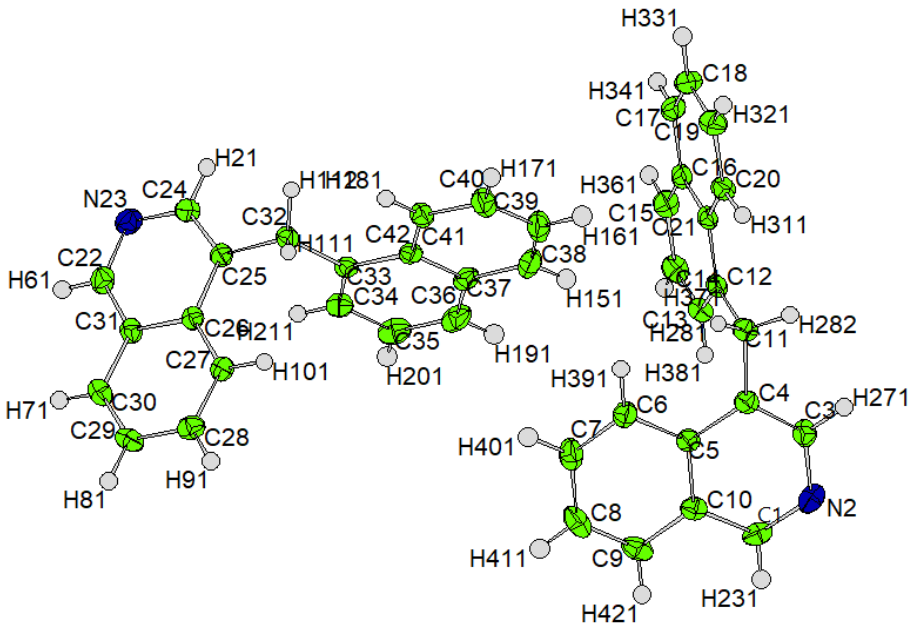

Thermal ellipsoids are drawn at the 50% probability level.

4-(Pyridin-2-ylmethyl)isoquinoline (4n):

Recrystallised from acetone by slow evaporation

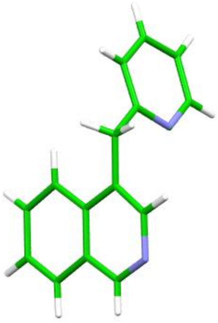

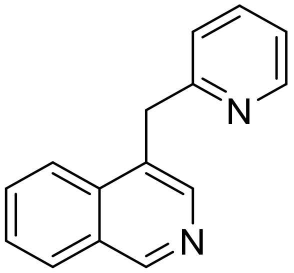

Formula C15 H12 N2

Crystal Class	monoclinic	Space Group	P 21/n
a	6.4010(2)	alpha	90
b	8.1364(2)	beta	91.842(3)
c	21.7622(7)	gamma	90
Volume	1132.81(6)	Z	4
Radiation type	Cu K\a	Wavelength	1.541840
Dx	1.29	Mr	220.27
Mu	0.601	Temperature (K)	150
Size	0.10x 0.10x 0.20		
Colour	translucent light bronze	Shape	block
Cell from	3969 Reflections	Theta range	4 to 76
Diffractometer type	multi-scan	Scan type	OMEGA
Absorption type	multi-scan	Transmission range	0.85 0.94
Reflections measured	9273	Independent reflections	2338
Rint	0.0003	Theta max	76.25
Hmin, Hmax	-7 7		
Kmin, Kmax	-7 10		
Lmin, Lmax	-27 27		
Refinement on Fsqd			
R-factor	0.048	Weighted R-factor	0.106
Max shift/su	0.0003		
Delta Rho min	-0.22	Delta Rho max	0.23
Reflections used	2338	sigma(I) limit	-3.00
Number of parameters	155	Goodness of fit	0.994

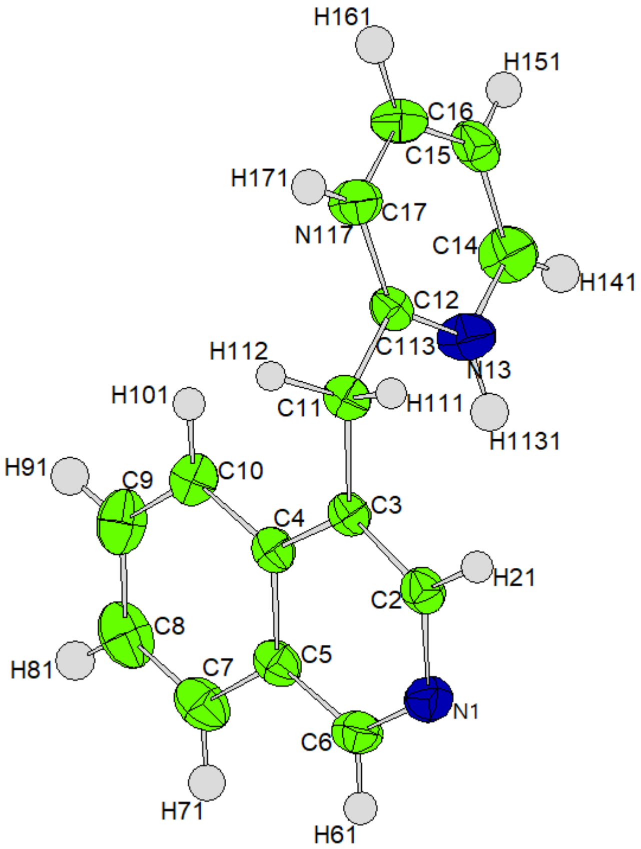

Thermal ellipsoids are drawn at the 50% probability level.

6. References

[1] A Nitrogen-Gas-Stream Cryostat for General X-ray Diffraction Studies, Cosier, J. Glazer, A. M. *J. Appl. Cryst.* **1986**, *19*, 105-107.

[2] SUPERFLIP – a computer program for the solution of crystal structures by charge flipping in arbitrary dimensions, Palatinus, L. Chapuis, G. *J. Appl. Cryst*. **2007**, *40,* 786-790.

[3] Crystal structures of increasingly large molecules: meeting the challenges with CRYSTALS software, Parois, P. Cooper, R. I. Thompson, A. L. *Chem. Cent. J.* **2015**, *9,* 30.

[4] CRYSTALS Enhancements: Dealing with Hydrogen Atoms in Refinement, Cooper, R. I. Thompson, A. L. Watkin, D. J. *J. Appl. Cryst.* **2010**, *43*, 1100-1107.

[5] (a) Evolution of the Dearomative Functionalization of Activated Quinolines and Isoquinolines: Expansion of the Electrophile Scope, Kischkewitz, M. Marinic, B. Kratena, N. Lai, Y. Hepburn, H. B. Dow, M. Christensen, K. E. Donohoe, T. J. *Angew. Chem. Int. Ed.* **2022**, *61*, e202204682; (b) sp3 C–H Bond Activation with Ruthenium(II) Catalysts and C(3)-Alkylation of Cyclic Amines, Sundararaju, B.; Achard, M.; Sharma, G. V. M.; Bruneau, C. *J. Am. Chem. Soc*. **2011**, *133,* 10340-10343.

[6] The Heck Reaction of Allylic Alcohols Catalysed by an Air-Stable Phosphinito Complex of Palladium(II), Sauza, A. Morales-Serna, J. A. García-Molina, M. Gavino, R. Cárdenas, J. *Synthesis*, **2012**, *2*, 272-282.

[7] Studies on Quinoline and Isoquinoline Derivatives. VIII. Hydration and Hydrogenation of Ethynyl Substituents attached to the Pyridine Moiety of Quinoline and Isoquinoline Rings, Konno, S. Shiraiwa, M. Yamanaka, H. *Chem. Pharm. Bull.* **1981**, *29,* 3554-3560.

[8] A new synthesis of 4-substituted isoquinolines, Minter, D. Re, M. A. *J. Org. Chem.* **1988**, *53*, 2653-2655.

[9] Reactivity of Isocoumarins. III. Reaction of 1-Ethoxychroman with Benzylamines, Yamato, M. Ishikawa, T. Kobayashi, T. *Chem. Pharm. Bull.* **1981**, *29*, 720-725.

[10] Microwave-Assisted Synthesis of Heterocycles by Rhodium(III)-Catalysed Annulation of *N*-Methoxyamides with α-Chlroraldehydes, Huang, J. R. Bolm, C. *Angew. Chem. Int. Ed.* **2017**, *56*, 15921-15925.

[11] Microwave-assisted palladium-catalysed reductive cyclization/ring-opening/aromatization cascade of oxazolidines to isoquinolines, Xu, X. Feng, H. Van der Eycken, E. V. *Org. Lett.* **2021**, *23*, 6578-6582.

[12] Heterocyclic monomers via reissert chemistry, Gibson, H. W. Rasco, M. L. Niu, Z. *Polymer Chemistry* **2011**, *49*, 3842-3851.

[13] Highly Chemoselective Deoxygenation of *N*-Heterocyclic *N*-Oxides Using Hantzsch Esters as Mild Reducing Agents, An, J. H. Kim, K. D. Lee, J. H. *J. Org. Chem.* **2021**, *86*, 2876-2894

[14] *N*-aryltetrahydroisoquinoline derivatives as HA-CD44 interaction inhibitors: Design, synthesis, computational studies and antitumor effect, Espejo-Román, J. M. Rubio-Ruiz, B. Chayah-Ghaddab, M. Vega-Gutierrez, C. García-García, G. Muguruza-Montero, A. Domene, C. Sánchez-Martín, R. M. Cruz-López, O. Conejo-García, A. *European Journal of Medicinal Chemistry*, **2023**, *258*, 115570

[15] Metal-Free Solvent Promoted Oxidation of Benzylic Secondary Amines to Nitrones with H_2O_2, Granato, A. S. Amarante, G. W. Adrio, J. *J. Org. Chem.* **2021**, *86*, 13817-13823

[16] A New Focused Microwave Approach to the Synthesis of Amino-Substituted Pyrroloisoquinolines and Pyrroloquinolines *via* a Sequential Multi-Component Coupling Process, Hopkin, M. D. Baxendale, I. R. Ley, S. V *Synthesis*, **2008**, *11*, 1688-1702.

[17] Synthesis of 4-Iodoisoquinolin-1(2H)-ones by a Dirhodium(II)-Catalysed 1,4-Bisfunctionalization of Isoquinolinium Iodide Salts, Fang, Z. Wang, Y. Wang, Y. *Org. Lett.* **2019**, *21*, 434-438.

[18] Construction of tropane derivatives by the organocatalytic asymmetric dearomatization of isoquinolines, Xu, J. H. Zheng, S. C. Zhang, J. W. Liu, X. Y. Tan, B. *Angew. Chem. Int. Ed.* **2016**, *55*, 11834-11839.

[19] Organocatalytic asymmetric 1,6-additions of *β*-ketoesters and glycine imine, Bernardi, L. López-Cantarero, J. Niess, B. Jørgensen, K. A. *J. Am. Chem. Soc.* **2007**, *129*, 5772-5778.
